# Stereoselective Synthesis and Antiproliferative Activity of Steviol-Based Diterpen Aminodiols

**DOI:** 10.3390/ijms21010184

**Published:** 2019-12-26

**Authors:** Dániel Ozsvár, Viktória Nagy, István Zupkó, Zsolt Szakonyi

**Affiliations:** 1 Institute of Pharmaceutical Chemistry, University of Szeged, Interdisciplinary Excellent Center, H-6720 Szeged, Hungary; daniel.ozsvar@pharm.u-szeged.hu; 2 Department of Pharmacodynamics and Biopharmacy, University of Szeged, H-6720 Szeged, Hungary; nagy.viktoria@pharm.u-szeged.hu (V.N.); zupko@pharm.u-szeged.hu (I.Z.); 3 Interdisciplinary Centre of Natural Products, University of Szeged, H-6720 Szeged, Hungary

**Keywords:** aminodiol, steviol, diterpene, triazole, click reaction, chiral catalysts, antiproliferative activity

## Abstract

A library of steviol-based trifunctional chiral ligands was developed from commercially available natural stevisoide and applied as chiral catalysts in the addition of diethylzinc to benzaldehyde. The key intermediate steviol methyl ester was prepared according to literature procedure. Depending on the epoxidation process, both *cis*- and *trans*-epoxyalcohols were obtained. Subsequent oxirane ring opening with primary and secondary amines afforded 3-amino-1,2-diols. The ring opening with sodium azide followed by a “click” reaction with alkynes resulted in dihydroxytriazoles. The regioselective ring closure of *N*-substituted aminodiols with formaldehyde was also investigated. The resulting steviol-type aminodiols were tested against a panel of human adherent cancer cell lines (A2780, SiHa, HeLa, and MDA-MB-231). It was consistently found that the *N*-benzyl substituent is an essential part within the molecule and the ring closure towards *N*-benzyl substituted oxazolidine ring system increased the antiproliferative activity to a level comparable with that of cisplatine. In addition, structure–activity relationships were examined by assessing substituent effects on the aminodiol systems.

## 1. Introduction

In recent years, aminodiols and their *N*-heterocyclic analogues have proven to be important building blocks of new chiral catalysts, or even complex bioactive molecules with significant biological activities [1,2]. Several aminodiol-based nucleoside analogues prepared recently have been shown to possess cardiovascular, cytostatic, and antiviral effects [3,4,5]. The Abbott aminodiol, found to be a useful building block for the synthesis of the potent renin inhibitors Zankiren^®^ and Enalkiren^®^, was introduced into the therapy of hypertension [6,7]. Aminodiols can also exert antidepressive activity. For example, (*S*,*S*)-reboxetine, a selective norepinephrine reuptake inhibitor, was approved in many countries for the treatment of unipolar depression [8]. Other aminodiols may serve as starting materials for the synthesis of biologically active natural compounds. For example, cytoxazone, a microbial metabolite isolated from *Streptomyces* species, is a selective modulator of the secretion of T_H_2 cytokine [2,9].

Besides their biological interest, aminodiols have also been applied as starting materials in asymmetric syntheses or as chiral auxiliaries and ligands in enantioselective transformations [1]. To develop new, efficient, and commercially available chiral catalysts, chiral natural products, including (+)- and (−)-*α*-pinene [10,11], (−)-nopinone [12], (+)-carene [13,14], (+)-sabinol [15], (−)-pulegone [16], or menthone [17], can serve as important starting materials for the synthesis of aminodiols. Monoterpene-based aminodiols have been demonstrated to be excellent chiral auxiliaries in a wide range of stereoselective transformations, including Grignard addition [18,19], intramolecular [2 + 2] photocycloaddition [20], and intramolecular radical cyclization [21].

**Figure 1:**
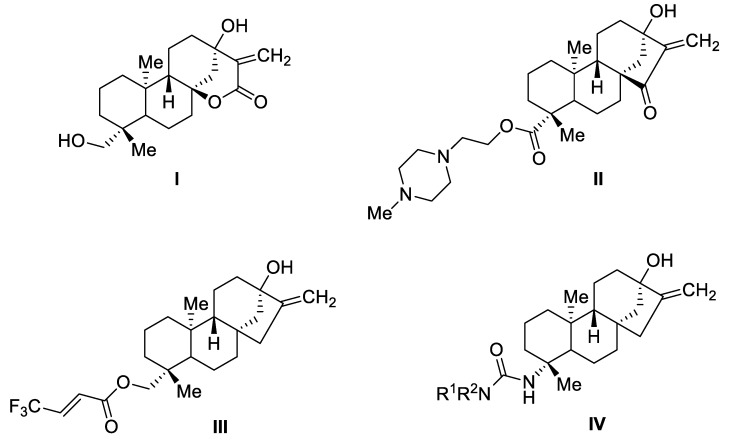
Examples for steviol derivatives with remarkable biological activities.

Although monoterpene-based 3-amino-1,2-diols are well-known compounds, their sesquiterpene or diterpene analogues are still not known in the literature. This is despite the fact that dihydroxy-substituted diterpene alkaloids as formal aminodiols with diverse pharmacological activity, owing to both basic nitrogen incorporated into their ring system and separated hydroxyl groups, have been thoroughly studied [22,23]. From this point of view, steviol, the aglycon of natural artificial sweetener stevisoide (isolated from the extract of the Paraguayan shrub *Stevia rebaudiana* L.) [24], has proved to be excellent starting material for the synthesis of *ent*-kaurane diterpenoids [25], with a wide range of biological activity (Figure 1), such as cytotoxic and apopthosis (**I**-**III**) [26,27,28,29], or glutathione *S*-transferase-inducing activity [30].

Some steviol derivatives that have substituted the carboxylic function with an amino group at the C-4 position possess inhibitory effects against Hepatitis B virus (Figure 1, **IV**) [31]. In recent years, several reviews have disclosed thorough discussions about the syntheses of steviol-based polyols [28,32], or even more complex structures [25,33,34]. It is important to note that, in most cases, acid sensitivity and the rapid rearrangement of the *ent*-kaurane ring system were mentioned as the limitation of transformations.

In the present contribution, we report the preparation of a new library of steviol-based chiral trifunctional synthons, such as aminodiols, azidodiols, and dihydroxytriazols, starting from commercially available natural stevioside. Our study also involves the evaluation of the resulting ligands as catalysts in the asymmetric addition of Et_2_Zn to benzaldehyde and their antiproliferative activity on multiple human cancer cell lines.

## 2. Results

### 2.1. Synthesis of Steviol-Based Epoxyalcohol Key Intermediates **4** and **5**

**Scheme 1:**
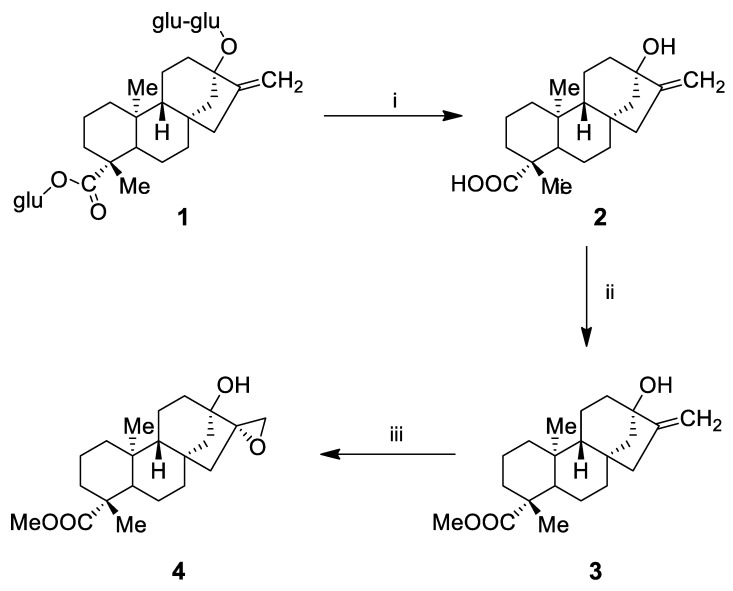
(**i**) 1. NaIO_4_, H_2_O, 25 °C, 16 h, 2. KOH, H_2_O, 100 °C, 2 h, 60%; (**ii**) CH_2_N_2_, Et_2_O, 25 °C, 5 min, 72%;.(**iii**) VO(acac)_2_, 70% *t*-BuOOH (1.5 equ.), dry toluene, 25 °C, 12 h, 65%

Steviol (**2**) was prepared starting from commercially available natural stevioside (**1**), the glycoside of steviol, in two steps, according to literature methods [26,35]. The esterification of **2** was accomplished with diazomethane, resulting in steviol methyl ester (**3**) [26]. The epoxidation of **3** with *t*-BuOOH in the presence of vanadyl acetylacetonate (VO(acac)_2_) as the catalyst furnished *cis*-epoxyalcohol **4** in a stereospecific reaction with known stereochemistry (Scheme 1) [32,36].

**Scheme 2:**
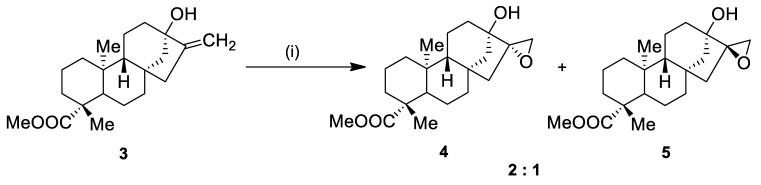
(**i**) Dimethyldioxirane (DMDO), acetone/H_2_O, 25 °C, 12 h, 64% overall yield.

The synthesis of diastereoisomeric *trans*-epoxyalcohol **5** was also attempted by several epoxidation methods, but in most cases **4** was isolated as a single product. However, when applying in situ-prepared dimethyldioxirane (DMDO) as a mild epoxydation reagent, diastereoisomer **5** could be obtained as minor component (**4**:**5** = 2:1 ratio, separated by preparative column chromatography, Scheme 2) [37].

### 2.2. Synthesis of Steviol-Based Dihydroxytriazoles via Azidodiol **6**

**Scheme 3:**
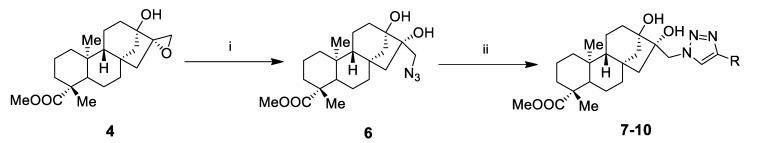
(**i**) NaN_3_, NH_4_Cl, EtOH/H_2_O, 70–80 °C, 6 h, 57%; (**ii**) Alkyne (2 equ.), 2 mol% CuSO_4*_5H_2_O, 10 mol% sodium ascorbate, DCM, 25 °C, 16 h, 74–91%.

The reaction of *cis*-epoxyalcohol **4** with sodium azide in the presence of ammonium chloride in a mixture of EtOH/water gave azidodiol **6**, from which triazoles **7**–**10** and **18** were generated by a “click” reaction with various substituted alkynes (Scheme 3) [38,39].

### 2.3. Synthesis of Steviol-Based Aminodiol Derivatives

**Scheme 4:**
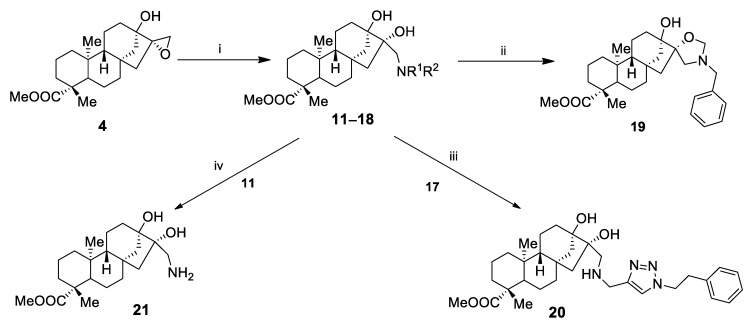
(**i**) R^1^R^2^NH (2 equ.), LiClO_4_ (2 equ.), MeCN, 25 °C, 3 d, 32–77%; (**ii**) 35% HCHO, Et_2_O, 25 °C, 2 h, 88–91% (see Table 1); (**iii**) 2-(Azidoethyl)benzene (2 equ.), 2 mol% CuSO_4*_5H_2_O, 10 mol% sodium ascorbate, DCM, 25 °C, 16 h, 62%; (**iv**) 5% Pd/C, H_2_ (1 atm), MeOH, 25 °C, 12 h, 57%.

The nucleophilic addition of amines to epoxyalcohols has proved to be an efficient method for the preparation of a highly diverse library of 3-amino-1,2-diols [14,40,41]. The opening of the oxirane ring of **4** was accomplished with different primary and secondary amines, resulting in a library of tridentate aminodiol derivatives (**11**–**18**) presented in Table 1. When aminodiol **11** was treated with formaldehyde at room temperature, spiro-oxazolidine **19** was obtained in a highly regioselective ring closure. Furthermore, an alternative triazol-type tridentate product **20** was synhesized with a click reaction, starting from *N*-propargyl-substituted aminodiol **17** and 2-phenylethyl azide, while the debenzylation of **11** by hydrogenolysis over Pd/C resulted in primary aminodiol **21** with moderate yield (Scheme 4).

**Scheme 5:**
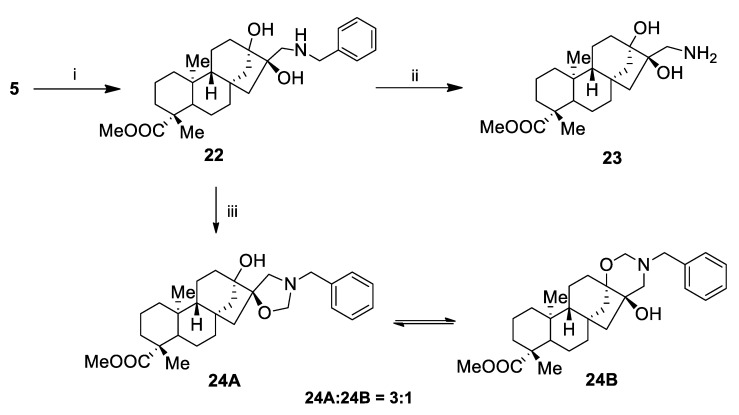
(**i**) BnNH_2_ (2 equ.), LiClO_4_ (2 equ.), MeCN, 25 °C, 3 d, 77%; (**ii**) 5% Pd/C, H_2_ (1 atm), MeOH, 25 °C, 12 h, 58%; (**iii**) 35% HCHO, Et_2_O, 25 °C, 2 h, 91%.

Diasteroisomeric primary aminodiol **23** was prepared by ring opening of *trans*-epoxyalcohol **5** with benzylamine, followed by hydrogenolysis of **22** over Pd/C. In contrast to the *cis* counterpart, the treatment of **22** with formaldehyde at room temperature gave an inseparable mixture of spiro-oxazolidine **24A** and 1,3-oxazine **24B** in a ratio of **24A**:**24B** = 3:1 (Scheme 5) [40].

### 2.4. Application of Aminodiol Derivatives as Chiral Ligands for Catalytic Addition of Diethylzinc to Benzaldehyde

**Scheme 6:**
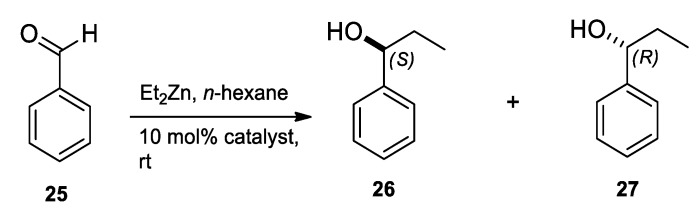
Model reaction for enantioselective catalysis.

Aminodiol derivatives **7**–**24** were applied as chiral catalysts in the enantioselective addition of diethylzinc to benzaldehyde **25** to study their catalytic effect on the formation of (*S*)- or (*R*)-1-phenyl-1-propanol **26** and **27** (Scheme 6).

The enantiomeric purity of the major product 1-phenyl-1-propanol enantiomer (**26** or **27**) was determined by GC analysis on a Chirasil-DEX CB column using the literature methods [10,42]. Low to moderate enantioselectivities were observed. The results obtained clearly show that all aminodiol derivatives favoured the formation of the (*R*)-enantiomer, whereas triazol-type compounds had only a weak catalytic effect on the transformation. Table 2 shows the best selected results. Aminodiol **12** afforded the best, but still moderate, *ee* value (*ee* = 52%) with an (*R*)-selectivity. In contrast to our earlier results, ring closure of **11** and **22** towards oxazolidines **19** and **24**, or decreasing the temperature (0 °C) or changing solvent (from *n*-hexane to toluene), did not increase catalytic activity. All other compounds were also examined as chiral ligands, but their selectivity was less than 10%.

### 2.5. Antiproliferative Activity of Aminodiols

The antiproliferative properties of the prepared diterpene aminodiol analogs determined on our cancer cell panel covered a wide range. Namely, a few of them elicited no relevant effect, while the activities of others were comparable to those of the reference agent cisplatin (Table 3) [43]. Based on these data, some conclusions concerning structure–activity relationships could be obtained. Since **5**, **6**, and **21** were ineffective, it seems that the presence of the aromatic ring—even in the case of triazol, oxazol, or benzyl derivatives—is a requirement of the cell-growth inhibiting effect of the compounds. Among 4-substituted triazoles (**7**–**10**), only 4-ferrocenyl derivative **8** exerted substantial action, especially against cervical SiHa cells. Testing the set of aminodiol analogs with secondary or tertiary amino function (**11**–**18**), it was consistently found that the *N*-benzyl substituent is an essential part within the molecule. Comparing **11** and **12**, it seemed to be evident that a secondary amino function is favoured over a tertiary group. Analogs containing α-methylbenzyl (**13** and **14**) or *bis*-(trifuoromethyl)methylbenzyl groups (**18**) exerted reasonable antiproliferative actions, i.e., their approximate IC_50_ values were 4–8 μM. It is interesting to note that in contrast to our earlier observation on aminodiols, *N*-benzyl-substituted oxazolidine **19** proved to be the most potent member of the prepared set. Namely, its potency determined on ovarian cancer cell line (A2780) was close to that of cisplatin, while it was substantially more effective than the reference agent on the other three cell lines (SiHa, HeLa, and MDA-MB-231). 1-Phenylethyl-substituted triazol **20** also exerted a modest action with IC_50_ values of 10–15 μM.

Diastereoisomeric *N*-benzyl **22** has somehow a lower cytotoxic effect than **11**. Meanwhile, the activity of the inseparable mixture of its oxazine-oxazolidine analog (**24A-B**) is comparable to that of **19**. Interestingly, **23** exhibited some minor action against MDA-MB-231 cells at 30 μM.

## 3. Discussion

Starting from commercially available stevioside, a new family of diterpene-based chiral aminodiols and triazolodiols was prepared through chiral epoxyalcohols as key intermediates via stereoselective transformations.

The resulting aminodiols exerted remarkable antiproliferative action on a panel of human cancer cell lines. The in vitro pharmacological studies clearly showed that the *N*-benzyl substituent on the amino function is essential.

Since some of the prepared molecules proved to be more potent than anticancer agent cisplatin used clinically, the design, synthesis, and investigation of further analogs—in particular, oxazolidine derivatives—are suggested. Concerning the outstanding action of the most potent member (**19**) of the current set of molecules, a mechanistic study to explore a probable mechanism of the action is considered to be an additional rational opportunity.

In addition, aminodiol and aminotriol derivatives were applied as chiral catalysts in the enantioselective addition of diethylzinc to benzaldehyde, observing moderate enantioselectivity but significant (*R*) selectivity.

## 4. Materials and Methods

### 4.1. General Methods

Commercially available compounds were used as obtained from suppliers (Molar Chemicals Ltd., Halásztelek, Hungary; Merck Ltd., Budapest, Hungary and VWR International Ltd., Debrecen, Hungary), while applied solvents were dried according to standard procedures. Optical rotations were measured in MeOH at 20 °C with a PerkinElmer 341 polarimeter (PerkinElmer Inc., Shelton, CT, USA). Chromatographic separations and the monitoring of reactions were carried out on a Merck Kieselgel 60 (Merck Ltd., Budapest, Hungary). Elemental analyses for all prepared compounds were performed on a PerkinElmer 2400 Elemental Analyzer (PerkinElmer Inc., Waltham, MA, USA). GC measurements for the separation of *O*-acetyl derivatives of enantiomers were performed on a Chirasil-DEX CB column (2500 × 0.25 mm I.D.) on a PerkinElmer Autosystem XL GC consisting of a flame ionization detector (PerkinElmer Corporation, Norwalk, CT, USA) and a Turbochrom Workstation data system (Perkin-Elmer Corp., Norwalk, CT, USA). Melting points were determined on a Kofler apparatus (Nagema, Dresden, Germany) and were uncorrected. ^1^H- and ^13^C-NMR spectra were recorded on a Brucker Avance DRX 500 spectrometer (500 MHz (^1^H) and 125 MHz (^13^C), δ = 0 (TMS)). Chemical shifts are expressed in ppm (δ) relative to TMS as the internal reference. *J* values are given by Hz. All ^1^H/^13^C NMR, NOESY, 2D-HMBC, and 2D-HMQC spectra are found in the Supplementary materials.

### 4.2. Starting Materials

Steviol methyl ester **3** was prepared in a two-step synthesis according to literature methods, starting from stevioside **1**, which is available commercially from Alfa Aesar Co [26,35].

### 4.3. Epoxydation of Steviol Methyl Ester **3**

Method A: A solution of briefly-dried *t*-BuOOH (extracted from its 70% H_2_O solution with toluene, 0.60 g, 4.6 mmol; sicc. Na_2_CO_3_) in dry toluene (10 mL) was added dropwise to a stirred pale-red solution of **3** (1.00 g, 3.0 mmol) and VO(acac)_2_ (3.2 mg) in dry toluene (15 mL) at room temperature. The red color of the solution darkened during the addition and then faded to brownish yellow. Stirring was continued for 12 h, and then a solution of KOH (0.25 g, 4.5 mmol) in brine (25 mL) was added. The mixture was extracted with toluene (3 × 50 mL), and the organic layer was washed with brine before it was dried (Na_2_SO_4_), filtered, and concentrated. The resulting crude product **4** (diastereomeric purity was checked by means ^1^H-NMR) was purified by flash column chromatography on silica gel with *n*-hexane/EtOAc 4:1 to obtain pure **4**.

Method B: **3** (1.00 g, 3.0 mmol) was dissolved in the mixture of acetone (110 mL) and water (140 mL). Then, oxone (44.14 g, 0.29 mol) and sodium bicarbonate (96.00 g, 1.14 mol) in three to four portions were added to the mixture with stirring at room temperature, and the mixture was stirred for 12 h and then filtered off. The aqueous acetone filtrate was extracted with Et_2_O (3 × 150 mL) and the organic phase was dried (Na_2_SO_4_) and evaporated to dryness. The resulting mixture of **4** and **5** was purified by column chromatography on silica gel with *n*-hexane/EtOAc 4:1 to obtain pure **4** and **5**.

#### 4.3.1. (2’*S*,4*R*,6a*S*,9*S*,11a*R*,11b*S*)-Methyl 9-hydroxy-4,11b-dimethyldodecahydro-1H-spiro[6a,9-methanocyclohepta[a]naphthalene-8,2’-oxirane]-4-carboxylate (**4**)

Yield: 0.68 g (65%, Method A), 0.44 g (43%, Method B), white crystals, m.p.: 144–146 °C. [α]${}_{D}^{20}$ = −83.0 (c 0.190, CHCl_3_). All spectroscopic data of **4** were consistent with those reported in literature [32,36].

#### 4.3.2. (2’*R*,4*R*,6a*S*,9*S*,11a*R*,11b*S*)-Methyl9-hydroxy-4,11b-dimethyldodecahydro-1H-spiro[6a,9-methanocyclohepta[a]naphthalene-8,2’-oxirane]-4-carboxylate (**5**) 

Yield: 0.22 g (21%, Method B), white crystals, m.p.: 149–151 °C, [α]${}_{D}^{20}$ = –48.0 (c 0.240, CHCl_3_). ^1^H NMR (500 MHz, CDCl_3_): δ 0.81–0.89 (4H, m), 0.97–1.08 (3H, m), 1.17 (3H, s), 1.43–1.97 (14H, m), 2.17 (2H, t, *J* = 24.4 Hz), 2.67 (1H, d, *J* = 5.4 Hz), 3.09 (1H, d, *J* = 5.3 Hz), 3.65 (3H, s). ^13^C NMR (125 MHz, CDCl_3_): δ 15.5, 19.1, 19.5, 21.5, 28.7, 35.5, 38.0, 39.3, 40.7, 41.4, 41.7, 43.8, 45.1, 46.9, 51.2, 52.8, 53.9, 56.8, 65.6, 76.3, 178.0. Anal. calcd. for C_21_H_32_O_4_: C, 72.38; H, 9.26. Found: C, 72.51; H, 8.98. 

### 4.4. (4R,6aS,8S,9S,11aR,11bS)-Methyl8-(azidomethyl)-8,9-dihydroxy-4,11b-dimethyltetradecahydro-6a,9-methanocyclohepta[a]naphthalene-4-carboxylate (**6**)

NaN_3_ (0.11 g, 1.7 mmol) and NH_4_Cl (0.09 g, 1.7 mmol) were added to a stirred solution of **4** (0.30 g, 0.86 mmol) in an EtOH and water 8:1 mixture (10 mL). The solution was reflux for 6 h, then water (20 mL) was added and the mixture was extracted with Et_2_O (3×20 mL). The organic phase was dried (Na_2_SO_4_) and evaporated to dryness. The crude product was purified by column chromatography on silica gel with EtOAc/*n*-hexane 9:1.

Yield: 0.19 g (57%), white crystals, m.p.: 157–160 °C, [α]${}_{D}^{20}$ = –19.0 (c 0.210, CHCl_3_). ^1^H NMR (500 MHz, CDCl_3_): δ 0.77–0.82 (4H, m), 0.90 (1H, d, *J* = 8.7 Hz), 0.96–1.04 (2H, m), 1.16 (3H, s), 1.36–1.57 (5H, m), 1.61–1.67 (3H, m), 1.72–1.88 (7H, m), 2.18 (1H, d, *J* = 13.3 Hz), 3.10 (1H, s), 3.45–3.51 (2H, m), 3.63 (3H, s). ^13^C NMR (125 MHz, CDCl_3_): δ 15.3, 19.0, 20.0, 22.0, 28.6, 33.6, 38.0, 39.3, 40.7, 41.6, 41.9, 42.9, 43.8, 51.1, 52.1, 54.6, 56.4, 56.7, 77.4, 80.6, 177.8. Anal. calcd. for C_21_H_33_N_3_O_4_: C, 64.42; H, 8.50; N, 10.73. Found: C, 64.65; H, 8.74; N, 10.59.

### 4.5. General Procedure for the “Click” Reaction of 6 for the Preparation of **7**–**10**

CuSO_4*_5H_2_O (2 mol%; 1.3 mg) catalyst, 10 mol% sodium ascorbate (5.2 mg), and alkyne (0.5 mmol) in DCM (10 mL) were added at 25 °C to a stirred solution of **6** (0.10 g, 0.3 mmol). Stirring was continued for 16 h at room temperature, then water (20 mL) was added and the mixture was extracted with DCM (3 × 20 mL). The organic phase was dried (Na_2_SO_4_) and evaporated to dryness. Compounds **7**–**10** were isolated after flash column chromatography on silica gel column with CHCl_3_/MeOH 9:1.

#### 4.5.1. (4*R*,6a*S*,8*S*,9*S*,11a*R*,11b*S*)-Methyl 8,9-dihydroxy-4,11b-dimethyl-8-((4-phenyl-1H-1,2,3-triazol-1-yl) methyl) tetradecahydro-6a,9-methanocyclohepta[a]naphthalene-4-carboxylate (**7**)

The reaction was accomplished with ethynylbenzene according to the general procedure. Yield: 0.11 g (83%), white crystals, m.p.: 190–193 °C, [α]${}_{D}^{20}$ = –26.0 (c 0.210, CHCl_3_). ^1^H NMR (500 MHz, DMSO): δ 0.78–0.80 (3H, m), 0.83–0.94 (2H, m), 0.98–1.07 (2H, m), 1.10 (3H, s), 1.16 (1H, d, *J* = 14.8 Hz), 1.28–1.45 (3H, m), 1.58–1.83 (11H, m), 2.04 (1H, d, *J* = 13.2 Hz), 3.57 (3H, s), 4.21 (1H, s), 4.28 (1H, d, *J* = 13.9 Hz), 4.51 (1H, d, *J* = 13.9 Hz), 4.80 (1H, s), 7.30–7.34 (1H, m), 7.44 (2H, t, *J* = 7.9 Hz), 7.83 (2H, d, *J* = 7.9, 8.2 Hz), 8.48 (1H, s).^13^C NMR (125 MHz, DMSO): δ 15.5, 19.1, 19.9, 22.2, 28.6, 33.6, 37.9, 39.2, 39.5, 40.4, 41.2, 42.0, 43.6, 51.3, 51.5, 54.3, 54.5, 56.2, 77.1, 79.5, 123.2, 125.5, 128.1, 129.4, 131.5, 146.1, 177.5. Anal. calcd. for C_29_H_39_N_3_O_4_: C, 70.56; H, 7.96; N, 8.51. Found: C, 70.34; H, 8.08; N, 8.11.

#### 4.5.2. Cyclopenta-2,4-dien-1-yl(2-(1-(((4*R*,6a*S*,8*S*,9*S*,11a*R*,11b*S*)-8,9-dihydroxy-4-(methoxycarbonyl)-4,11b-dimethyltetradecahydro-6a,9-methanocyclohepta[a]naphthalen-8-yl)methyl)-1H-1,2,3-triazol-4-yl)cyclopenta-2,4-dien-1-yl)iron (**8**)

The reaction was accomplished with ethynylferrocene according to the general procedure. Yield: 0.14 g (91%), yellow crystals, m.p.: 138–142 °C, [α]${}_{D}^{20}$ = –15.0 (c 0.275, CHCl_3_). ^1^H NMR (500 MHz, DMSO): δ 0.78–0.92 (5H, m), 0.98–1.16 (6H, m), 1.25–1.30 (1H, m), 1.37–1.46 (2H, s), 1.57–1.82 (11H, m), 2.04 (1H, d, *J* = 12.6 Hz), 3.57 (3H, s), 4.03 (5H, s), 4.19 (1H, s), 4.23 (1H, d, *J* = 13.9 Hz), 4.29 (2H, s), 4.47 (1H, d, *J* = 13.9 Hz), 4.70 (2H, s), 4.77 (1H, s), 8.1 (1H, s). ^13^C NMR (125 MHz, DMSO): δ 15.5, 19.1, 19.9, 22.2, 28.6, 33.6, 37.9, 39.2, 39.6, 41.1, 41.9, 43.6, 51.3, 51.5, 54.1, 54.5, 56.2, 66.7, 66.8, 68.6, 69.7, 76.8, 77.2, 79.5, 122.5, 144.9, 177.5. Anal. calcd. for C_33_H_43_FeN_3_O_4_: C, 65.89; H, 7.20; Fe, 9.28; N, 6.99. Found: C, 66.03; H, 7.35; Fe, 9.02; N, 6.79.

#### 4.5.3. (4*R*,6a*S*,8*S*,9*S*,11a*R*,11b*S*)-Methyl 8,9-dihydroxy-4,11b-dimethyl-8-((4-(pyridin-2-yl)-1H-1,2,3-triazol-1-yl) methyl) tetradecahydro-6a,9-methanocyclohepta[a]naphthalene-4-carboxylate (**9**)

Yield: 0.11 g (89%), white crystals, m.p.: 218–220 °C, [α]${}_{D}^{20}$ = –9.0 (c 0.195, CHCl_3_). ^1^H NMR (500 MHz, CDCl_3_): δ 0.82–0.86 (4H, m), 0.99–1.05 (3H, m), 1.16 (3H, s), 1.35–1.47 (2H, m), 1.59–1.96 (12H, m), 2.10–2.12 (1H, m), 2.19 (1H, d, *J* = 13.7 Hz), 3.64 (3H, s), 4.53 (2H, dd, *J* = 14.0, 26.2 Hz), 7.36–7.38 (1H, m), 8.05 (1H, s), 8.19 (1H, d, *J* = 7.6 Hz), 8.55 (1H, d, *J* = 2.8 Hz), 9.00 (1H, s). ^13^C NMR (125 MHz, CDCl_3_): δ 15.3, 19.0, 19.8, 22.0, 28.6, 33.5, 38.0, 39.3, 40.7, 41.4, 41.8, 43.4, 43.8, 51.1, 52.1, 54.5, 54.5, 56.7, 77.1, 80.3, 122.7, 123.9, 126.9, 133.2, 144.2, 146.7, 148.8, 177.8. Anal. calcd. for C_28_H_38_N_4_O_4_: C, 67.99; H, 7.74; N, 11.33. Found: C, 68.32; H, 7.56; N, 11.05.

#### 4.5.4. (4*R*,6a*S*,8*S*,9*S*,11a*R*,11b*S*)-Methyl8-((4-cyclopropyl-1H-1,2,3-triazol-1-yl)methyl)-8,9-dihydroxy-4,11b-dimethyltetradecahydro-6a,9-methanocyclohepta[a]naphthalene-4-carboxylate (**10**)

Yield: 0.09 g (74%), colourless oil, [α]${}_{D}^{20}$ = +5.0 (c 0.240, CDCl_3_). ^1^H NMR (500 MHz, CHCl_3_): δ 0.80–0.84 (6H, m), 0.94–1.04 (5H, m), 1.16 (3H, s), 1.33–1.46 (2H, m), 1.58–1.95 (13H, m), 2.09–2.11 (1H, m), 2.17 (1H, d, *J* = 13.5 Hz), 3.63 (3H, s), 4.32 (1H, d, *J* = 13.8 Hz), 4.45 (1H, d, *J* = 13.8 Hz), 7.40 (1H, s). ^13^C NMR (125 MHz, CDCl_3_): δ 6.6, 7.9, 7.9, 15.3, 19.0, 19.8, 22.0, 28.6, 33.4, 38.0, 39.3, 40.6, 41.2, 41.8, 43.2, 43.7, 51.2, 52.1, 54.2, 54.5, 56.7, 77.1, 80.2, 122.7, 149.8, 177.9. Anal. calcd. for C_26_H_39_N_3_O_4_: C, 68.24; H, 8.59; N, 9.18. Found: C, 68.63; H, 8.52; N, 8.86.

### 4.6. General Procedure for the Preparation of **11**–**18**

LiClO_4_ (91.5 mg, 0.9 mmol) and the appropriate amine (0.9 mmol) were added to a solution of **4** (150.0 mg, 0.4 mmol) in MeCN (10 mL). The solution was stirred for three days at room temperature, then water (20 mL) was added and the mixture was extracted with Et_2_O (3 × 15 mL). The organic phase was dried (Na_2_SO_4_) and evaporated to dryness. The crude product was purified by column chromatography on silica gel with MeOH/CHCl_3_ 1:9.

#### 4.6.1. (4*R*,6a*S*,8*S*,9*S*,11a*R*,11b*S*)-Methyl 8-((benzylamino)methyl)-8,9-dihydroxy-4,11b-dimethyltetradecahydro-6a,9-methanocyclohepta[a]naphthalene-4-carboxylate (**11**)

The reaction was accomplished with benzylamine according to the general procedure. Yield: 0.13 g (67%), white crystals, m.p.: 91–93 °C, [α]${}_{D}^{20}$ = –15.0 (c 0.250, CDCl_3_). ^1^H NMR (500 MHz, CHCl_3_): δ 0.74–0.79 (1H, m), 0.81 (3H, s), 0.85 (1H, d, *J* = 8.5 Hz), 0.94–1.02 (2H, m), 1.15 (3H, s), 1.32–1.56 (5H, m), 1.60–1.74 (6H, m), 1.78–1.86 (4H, m), 2.16 (1H, d, *J* = 13.3 Hz), 2.77–2.82 (2H, m), 3.62 (3H, s), 3.81 (2H, dd, *J* = 13.2, 31.0 Hz), 7.25–7.34 (5H, m). ^13^C NMR (125 MHz, CDCl_3_): δ 15.3, 19.0, 19.8, 22.0, 28.6, 33.2, 38.1, 39.3, 40.7, 41.6, 42.0, 43.4, 43.8, 51.1, 52.3, 53.3, 53.9, 54.7, 56.8, 76.4, 80.6, 127.4, 128.3, 128.6, 138.9, 177.9. Anal. calcd. for C_28_H_41_NO_4_: C, 73.81; H, 9.07; N, 3.07. Found: C, 74.01; H, 9.25; N, 3.02.

#### 4.6.2. (4*R*,6a*S*,8*S*,9*S*,11a*R*,11b*S*)-Methyl 8-((benzyl(methyl)amino)methyl)-8,9-dihydroxy-4,11b-dimethyltetradecahydro-6a,9-methanocyclohepta[a]naphthalene-4-carboxylate (**12**)

The reaction was accomplished with *N*-benzylmethylamine according to the general procedure. Yield: 0.15 g (75%), white crystals, m.p.: 96–98 °C, [α]${}_{D}^{20}$ = –26.0 (c 0.240, CHCl_3_). ^1^H NMR (500 MHz, CDCl_3_): δ 0.76–0.88 (5H, m), 0.92–1.03 (2H, m), 1.15 (3H, s), 1.33–1.93 (15H, m), 2,17 (1H, d, *J* = 13.0 Hz), 2.23 (3H, s), 2.44 (1H, d, *J* = 12.5 Hz), 2.79 (1H, d, *J* = 12.4 Hz), 3.54 (1H, d, *J* = 13.0 Hz), 3.63 (1H, s), 3.71 (1H, d, *J* = 12.9 Hz), 7.25–7.34 (5H, m). ^13^C NMR (125 MHz, CDCl_3_): δ 15.4, 19.0, 20.0, 22.1, 28.7, 31.2, 38.1, 39.2, 40.7, 41.4, 42.1, 42.6, 43.8, 43.9, 51.1, 54.7, 56.8, 57.1, 60.4, 63.0, 74.9, 79.7, 127.4, 128.4, 129.0, 138.1, 177.9. Anal. calcd. for C_29_H_43_NO_4_: C, 74.16; H, 9.23; N, 2.98. Found: C, 74.41; H, 9.35; N, 2.82.

#### 4.6.3. (4*R*,6a*S*,8*S*,9*S*,11a*R*,11b*S*)-Methyl 8,9-dihydroxy-4,11b-dimethyl-8-((((*R*)-1-phenylethyl)amino) methyl) tetradecahydro-6a,9-methanocyclohepta[a]naphthalene-4-carboxylate (**13**)

The reaction was accomplished with (*R*)-α-methylbenzylamine according to the general procedure. Yield: 0.11 g (52%), white crystals, m.p.: 131–133 °C, [α]${}_{D}^{20}$ = –78.0 (c 0.263, CHCl_3_). ^1^H NMR (500 MHz, CDCl_3_): δ 0.69–0.78 (5H, m), 0.92–0.99 (2H, m), 1.14 (3H, s), 1.26–1.41 (8H, m), 1.54–1.83 (10H, m), 2.15 (1H, d, *J* = 13.3 Hz), 2.60 (2H, dd, *J* = 12.0, 37.7 Hz), 3.61 (3H, s), 3.66 (2H, dd, *J* = 6.6, 13.3 Hz), 7.22–7.33 (5H, m). ^13^C NMR (125 MHz, CDCl_3_): δ 15.2, 19.0, 19.7, 22.0, 23.9, 28.6, 33.1, 38.0, 39.2, 40.6, 41.6, 42.0, 43.3, 43.8, 51.0, 51.1, 53.2, 54.7, 56.8, 58.6, 76.5, 80.6, 126.6, 127.2, 128.5, 144.7, 177.9. Anal. calcd. for C_29_H_43_NO_4_: C, 74.16; H, 9.23; N, 2.98. Found: C, 74.45; H, 9.50; N, 2.79.

#### 4.6.4. (4*R*,6a*S*,8*S*,9*S*,11a*R*,11b*S*)-Methyl 8,9-dihydroxy-4,11b-dimethyl-8-((((*S*)-1-phenylethyl)amino) methyl) tetradecahydro-6a,9-methanocyclohepta[a]naphthalene-4-carboxylate (**14**)

The reaction was accomplished with (*S*)-α-methylbenzylamine according to the general procedure. Yield: 0.12 g (59%), white crystals, m.p.: 111–112 °C, [α]${}_{D}^{20}$ = +8 (c 0.263, CHCl_3_). ^1^H NMR (500 MHz, CDCl_3_): δ 0.74–0.84 (5H, m), 0.94–1.01 (2H, m), 1.14 (3H, s), 1.26–1.43 (8H, m), 1.57–1.87 (10H, m), 2.16 (1H, d, *J* = 13.3 Hz), 2.58 (1H, d, *J* = 12.0 Hz), 2.71 (1H, d, *J* = 12.0 Hz), 3.63 (3H, s), 3.77 (1H, dd, *J* = 6.6, 13.2 Hz), 7.23–7.34 (5H, m). ^13^C NMR (125 MHz, CDCl_3_): δ 15.3, 19.0, 19.8, 22.0, 23.8, 28.6, 29.7, 33.3, 38.1, 39.3, 40.7, 41.5, 42.0, 43.5, 43.8, 51.0, 51.1, 53.1, 54.7, 56.8, 58.5, 76.4, 80.6, 126.5, 127.2, 128.6, 177.9. Anal. calcd. for C_29_H_43_NO_4_: C, 74.16; H, 9.23; N, 2.98. Found: 74.47; H, 9.47; N, 2.88. 

#### 4.6.5. (4*R*,6a*S*,8*S*,9*S*,11a*R*,11b*S*)-Methyl 8,9-dihydroxy-8-((isopropylamino)methyl)-4,11b-dimethyltetradecahydro-6a,9-methanocyclohepta[a]naphthalene-4-carboxylate (**15**)

The reaction was accomplished with isopropylamine according to the general procedure. Yield: 0.10 g (57%), white crystals, m.p.: 231–235 °C, [α]${}_{D}^{20}$ = –27 (c 0.254, CDCl_3_). ^1^H NMR (500 MHz, CDCl_3_): δ 0.78–0.81 (4H, m), 0.91–1.02 (3H, m), 1.15 (3H, s), 1.25–1.29 (1H, m), 1.33–1.50 (9H, m), 1.59–1.86 (11H, m), 2.17 (1H, d, *J* = 13.0 Hz), 3.07 (1H, d, *J* = 12.6 Hz), 3.18 (1H, d, *J* = 12.6 Hz), 3.48–3,51 (1H, m), 3.64 (3H, s). ^13^C NMR (125 MHz, CDCl_3_): δ 15.4, 18.8, 19.0, 19.2, 19.8, 22.0, 28.7, 32.7, 38.0, 39.2, 40.6, 41.3, 41.9, 43.0, 43.7, 47.3, 50.8, 51.2, 53.2, 54.4, 56.7, 75.3, 80.5, 177.9. Anal. calcd. for C_24_H_41_NO_4_: C, 70.72; H, 10.14; N, 3.44. Found: C, 70.94; H, 10.01; N, 3.81.

#### 4.6.6. (4*R*,6a*S*,8*S*,9*S*,11a*R*,11b*S*)-Methyl 8-((diethylamino)methyl)-8,9-dihydroxy-4,11b-dimethyltetradecahydro-6a,9-methanocyclohepta[a]naphthalene-4-carboxylate hydrochloride (**16**)

The reaction was accomplished with diethylamine according to the general procedure. Yield: 0.11 g (63%), white crystals, m.p.: 183–185 °C, [α]${}_{D}^{20}$ = –17 (c 0.240, CHCl_3_). ^1^H NMR (500 MHz, DMSO): δ 0.75–0.81 (4H, m), 0.88 (1H, s), 0.98–1.07 (2H, m), 1.11 (3H, s), 1.21–1.23 (6H, m), 1.30–1.23 (2H, m), 1.53–1.77 (13H, m), 2.04 (1H, d, *J* = 12.6 Hz), 3.07–3.21 (6H, m), 3.57 (3H, s), 4.51 (1H, s), 5.09 (1H, s), 9.17 (1H, s). ^13^C NMR (125 MHz, DMSO): δ 8.8, 9.0, 15.4, 19.1, 19.8, 22.2, 28.6, 33.1, 37.9, 39.1, 40.4, 41.7, 41.9, 42.4, 43.6, 46.8, 48.4, 51.5, 53.7, 54.2, 55.5, 56.2, 75.0, 80.3, 177.5. Anal. calcd. for C_25_H_44_ClNO_4_: C, 65.55; H, 9.68; Cl, 7.74; N, 3.06. Found: C, 65.73; H, 9.49; Cl, 7.96; N, 3.11.

#### 4.6.7. (4*R*,6a*S*,8*S*,9*S*,11a*R*,11b*S*)-Methyl 8,9-dihydroxy-4,11b-dimethyl-8-((prop-2-yn-1-ylamino)methyl) tetradecahydro-6a,9-methanocyclohepta[a]naphthalene-4-carboxylate (**17**)

The reaction was accomplished with propargylamine according to the general procedure. Yield: 0.13 g (72%), white crystals, m.p.: 123–124 °C, [α]${}_{D}^{20}$ = –32 (c 0.250, CHCl_3_). ^1^H NMR (500 MHz, CDCl_3_): δ 0.77–0.83 (4H, m), 0.90 (1H, d, *J* = 8.6 Hz), 0.95–1.04 (2H, m), 1.16 (3H, s), 1.35–1.45 (3H, m), 1.61–1.89 (12H, m), 2.17 (1H, d, *J* = 11.0 Hz), 2.25 (1H, m), 2.89 (2H, dd, *J* = 12.0, 23.2 Hz), 3.46 (2H, dd, *J* = 17.1, 35.7 Hz), 3.63 (3H, s). ^13^C NMR (125 MHz, CDCl_3_): δ 15.3, 19.0, 19.8, 22.0, 28.6, 33.1, 38.1, 38.3, 39.3, 40.7, 41.6, 42.0, 43.4, 43.8, 51.1, 52.0, 53.3, 54.7, 56.8, 72.2, 76.4, 80.6, 80.94, 177.9. Anal. calcd. for C_24_H_37_NO_4_: C, 71.43; H, 9.24; N, 3.47. Found: C, 71.69; H, 9.47; N, 3.28.

#### 4.6.8. (4*R*,6a*S*,8*S*,9*S*,11a*R*,11b*S*)-Methyl 8-(((3,5-bis(trifluoromethyl)benzyl)amino)methyl)-8,9-dihydroxy-4,11b-dimethyltetradecahydro-6a,9-methanocyclohepta[a]naphthalene-4-carboxylate (**18**)

The reaction was accomplished with 3,5-*bis*(trifluoromethyl)benzylamine according to the general procedure. Yield: 0.08 g (32%), white crystals, m.p.: 173–176 °C, [α]${}_{D}^{20}$ = –38 (c 0.370, CHCl_3_). ^1^H NMR (500 MHz, CDCl_3_): δ 0.75–0.79 (1H, m), 0.82 (3H, s), 0.86–0.88 (1H, m), 0.95–1.03 (2H, m), 1.15 (3H, s), 1.34–1.57 (5H, m), 1.63–1.89 (10H, m), 2.79 (2H, dd, *J* =11.8, 35.3 Hz), 3.63 (3H, s), 3.90–3.96 (2H, m), 7.78–7.79 (3H, m). ^13^C NMR (125 MHz, CDCl_3_): δ 15.3, 19.0, 19.8, 22.0, 28.6, 33.2, 38.0, 39.2, 40.6, 41.5, 42.0, 43.4, 43.7, 51.1, 52.8, 53.1, 53.2, 54.6, 56.7, 76.6, 80.6, 121.3, 122.2, 124.4, 128.2, 131.8 (2 CF_3_, dd, *J* = 33.2, 66.4 Hz), 142.3, 177.9. Anal. calcd. for C_30_H_39_F_6_NO_4_: C, 60.90; H, 6.64; N, 2.37. Found: C, 61.11; H, 6.89; N, 2.12.

#### 4.6.9. (4*R*,6a*S*,8*R*,9*S*,11a*R*,11b*S*)-Methyl 8-((benzylamino)methyl)-8,9-dihydroxy-4,11b-dimethyltetradecahydro-6a,9-methanocyclohepta[a]naphthalene-4-carboxylate (**22**)

The reaction was accomplished starting from epoxyalcohol **5** (0.12 g, 0.3 mmol), MeCN (10 mL), LiClO_4_ (70.2 mg, 0.7 mmol), and benzylamine (70.7 mg, 0.7 mmol) according to the general procedure. Yield: 0.12 g (77%), white crystals, m.p.: 97–99 °C, [α]${}_{D}^{20}$ = –31 (c 0.164, CHCl_3_). ^1^H NMR (500 MHz, CDCl_3_): δ 0.78–0.85 (4H, m), 0.93–1.03 (3H, m), 1.15 (3H, s), 1.28–1.52 (7H, m), 1.68–1.98 (8H, m), 2.17 (1H, d, *J* = 14.0 Hz), 2.58 (1H, d, *J* = 12.0 Hz), 3.02 (1H, d, *J* = 12.0 Hz), 3.62 (3H, s), 3.76–3.82 (2H, m), 7.24–7.34 (5H, m). ^13^C NMR (125 MHz, CDCl_3_): δ 15.2, 19.1, 20.1, 21.6, 28.7, 35.7, 38.1, 39.3, 40.6, 41.2, 42.1, 43.8, 44.8, 51.1, 53.8, 54.7, 54.8, 56.9, 57.8, 76.3, 82.4, 127.2, 128.2, 128.5, 139.3, 177.9. Anal. calcd. for C_28_H_41_NO_4_: C, 73.81; H, 9.07; N, 3.07. Found: C, 74.03; H, 9.29; N, 2.95.

### 4.7. General Procedure for Ring Closure of **11** and **22** with Formaldehyde

Aqueous formaldehyde (35%; 5 mL) was added to a solution of aminodiol **11** or **22** (0.10 g, 0.2 mmol) in Et_2_O (5 mL) and the mixture was stirred at room temperature. After 1 h of stirring, the mixture was made alkaline with 10% aqueous KOH (10 mL) and extracted with Et_2_O (3×50 mL). After drying (Na_2_SO_4_) and solvent evaporation, crude products **19** and **24** were purified by recrystallization from DCM.

#### 4.7.1. (4*R*,5’*S*,6a*S*,9*S*,11a*R*,11b*S*)-Methyl3’-benzyl-9-hydroxy-4,11b-dimethyldodecahydro-1H-spiro[6a,9-methanocyclohepta[a]naphthalene-8,5’-oxazolidine]-4-carboxylate (**19**)

Yield: 0.09 g (91%), white crystals, m.p.: 106–107 °C, [α]${}_{D}^{20}$ = –44 (c 0.254, CHCl_3_). ^1^H NMR (500 MHz, CDCl_3_): δ 0.75–0.80 (1H, m), 0.82 (3H, s), 0.84–0.86 (1H, m), 0.95–1.03 (2H, m), 1.16 (3H, s), 1.32–1.49 (4H, m), 1.53–1.91 (10H, m), 2.03 (1H, d, *J* = 14.0 Hz), 2.17 (1H, d, *J* = 13.3 Hz), 2.72 (1H, s), 2.93 (2H, dd, *J* = 11.2, 31.0 Hz), 3.63 (3H, s), 3.70–3.75 (2H, s), 4.25 (1H, d, *J* = 4.9 Hz), 4.46 (1H, d, *J* = 4.9 Hz), 7.24–7.36 (5H, m). ^13^C NMR (125 MHz, CDCl_3_): δ 15.4, 19.1, 19.9, 22.0, 28.7, 32.5, 38.1, 39.2, 40.7, 41.4, 41.8, 43.8, 45.4, 51.1, 54.3, 56.9, 57.0, 57.2, 58.2, 79.5, 86.4, 86.5, 127.3, 128.4, 128.7, 138.7, 177.8. Anal. calcd. for C_29_H_41_NO_4_: C, 74.48; H, 8.84; N, 3.00. Found: C, 74.33; H, 9.06; N, 3.18.

#### 4.7.2. (4*R*,5’*R*,6a*S*,9*S*,11a*R*,11b*S*)-Methyl3’-benzyl-9-hydroxy-4,11b-dimethyldodecahydro-1H-spiro[6a,9-methanocyclohepta[a]naphthalene-8,5’-oxazolidine]-4-carboxylate (**24A**) and (4R,6aR,7aR,11aS,13aR,13bS)-Methyl 9-benzyl-7a-hydroxy-4,13b-dimethylhexadecahydro-6a,11a-methanonaphtho[1’,2’:5,6]cyclohepta[1,2-e][1,3]oxazine-4-carboxylate (**24B**)

Yield of a 3:1 mixture: 0.09 g (91%), white crystals, m.p.: 110–114 °C, [α]${}_{D}^{20}$ = –11 (c 0.234, CHCl_3_). ^1^H NMR (500 MHz, CDCl_3_): δ 0.82–0.89 (6H, m, minor overlapped with major), 0.95–1.05 (4H, m, minor overlapped with major), 1.15 (3H, s, minor), 1.16 (3H, s, major), 1.32–1.58 (9H, m, minor overlapped with major), 1.69–1.93 (12H, m, minor overlapped with major), 2.16 (1H, d, major, *J* = 13.7 Hz), 2.21 (1H, d, minor, *J* = 9.0 Hz), 2.39 (1H, d, major, *J* = 12.2 Hz), 2.70 (1H, s, major), 2.81 (1H, d, major, *J* = 12.2 Hz), 3.45 (2H, dd, *J* = 15.2, 13.2 Hz), 3.62 (3H, s, minor), 3.63 (3H, s, major), 3.65–3.66 (1H, m, major), 3.88 (1H, s, minor), 4.01 (1H, d, major, *J* = 8.0 Hz), 4.24 (1H, d, major, *J* = 8.0 Hz), 4.51 (1H, d, minor, *J* = 1.7 Hz), 7.25–7.33 (5H, m, minor overlapped with major). ^13^C NMR (125 MHz, CDCl_3_): δ 15.3, 19.0, 21.6, 28.7, 33.9, 37.5, 38.0, 39.2, 40.6, 41.0, 41.9, 42.5, 43.8, 50.6, 51.1, 51.8, 55.8, 56.0, 56.8 (CH_3_, major), 56.9 (CH_3_, minor), 57.1, 62.9, 71.9, 79.2, 82.8, 85.6, 127.4, 128.5, 128.6, 128.9, 137.1, 177.9. Anal. calcd. for C_29_H_41_NO_4_: C, 74.48; H, 8.84; N, 3.00. Found: C, 74.54; H, 8.85; N, 3.01. 

#### 4.8. (4R,6aS,8S,9S,11aR,11bS)-Methyl 8,9-dihydroxy-4,11b-dimethyl-8-((((1-phenethyl-1H-1,2,3-triazol-4-yl)methyl)amino)methyl)tetradecahydro-6a,9-methanocyclohepta[a]naphthalene-4-carboxylate (**20**)

CuSO_4*_5H_2_O (2 mol%; 1.8 mg) catalyst, 10 mol% sodium ascorbate (7.3 mg), and 2-(azidoethyl)benzene (0.11 g, 0.7 mmol) were added to a solution of **17** aminodiol (0.15 g, 0.4 mmol) in DCM (15 mL). The mixture was stirred for 6 h at room temperature. Upon completion of the reaction (indicated by TLC), the mixture was extracted with CH_2_Cl_2_ and water (3 × 20 mL). The combined organic phase was dried (Na_2_SO_4_), filtered, and concentrated. The crude product was purified by column chromatography on silica gel with CHCl_3_/MeOH 9:1.

Yield: 0.13 g (62%), white crystals, m.p.: 218–220 °C, [α]${}_{D}^{20}$ = –33 (c 0.360, CHCl_3_). ^1^H NMR (500 MHz, CDCl_3_): δ 0.76–0.81 (4H, m), 0.87–0.88 (1H, m), 0.95–1.03 (2H, m), 1.15 (3H, s), 1.33–1.42 (5H, m), 1.50–1.88 (12H, m), 2.16 (1H, d, *J* = 13.2 Hz), 2.80 (2H, dd, *J* = 12.1, 20.3 Hz), 3.20 (2H, t, *J* = 14.6 Hz), 3.63 (3H, s), 3.90 (2H, s), 4.58 (2H, t, *J* = 14.6 Hz), 7.10–7.31 (6H, m). ^13^C NMR (125 MHz, CDCl_3_): δ 15.3, 19.0, 19.8, 22.0, 28.6, 29.7, 33.1, 36.7, 38.0, 39.2, 40.6, 41.5, 42.0, 43.4, 43.8, 44.5, 51.1, 51.7, 52.3, 53.2, 54.6, 56.7, 76.5, 80.5, 122.2, 127.2, 128.7, 128.8, 137.0, 145.3, 177.9. Anal. calcd. for C_33_H_48_N_4_O_4_: C, 70.18; H, 8.57; N, 9.92. Found: C, 70.41; H, 8.89; N, 9.77.

### 4.9. General Procedure for Debenzylation of **11** and **22**

Aminodiol **11** or **22** (0.10 g, 0.2 mmol) in MeOH (50 mL) were added to a suspension of palladium-on-carbon (5% Pd/C, 0.10 g) in MeOH (50 mL), and the mixture was stirred under a H_2_ atmosphere (1 atm) at room temperature. After completion of the reaction (as monitored by TLC, 24 h), the mixture was filtered through a Celite pad and the solution was evaporated to dryness. The crude products were recrystallized in Et_2_O, resulting in primary aminodiols **21** or **23**.

#### 4.9.1. (4*R*,6a*S*,8*S*,9*S*,11a*R*,11b*S*)-Methyl 8-(aminomethyl)-8,9-dihydroxy-4,11b-dimethyltetradecahydro-6a,9-methanocyclohepta[a]naphthalene-4-carboxylate (**21**)

Yield: 0.05 g (57%), white crystals, m.p.: 139–142 °C, [α]${}_{D}^{20}$ = –27 (c 0.225, CHCl_3_). ^1^H NMR (500 MHz, MeOH): δ 0.76–0.80 (4H, m), 0.85–0.86 (1H, m), 0.92–1.01 (2H, m), 1.06 (3H, m), 1.19–1.79 (15H, m), 2.04 (1H, d, *J* = 13.1 Hz), 2.69 (2H, dd, *J* = 13.1, 27.1 Hz), 3.53 (3H, s). ^13^C NMR (125 MHz, MeOH): δ 16.2, 20.3, 20.9, 23.3, 29.2, 34.4, 39.2, 40.6, 41.9, 42.6, 43.3, 44.5, 45.1, 46.0, 51.8, 52.9, 56.1, 58.1, 78.6, 81.1, 179.7. Anal. calcd. for C_21_H_35_NO_4_: C, 69.01; H, 9.65; N, 3.83. Found: C, 69.25; H, 9.37; N, 4.03.

#### 4.9.2. (4*R*,6a*S*,8*R*,9*S*,11a*R*,11b*S*)-Methyl 8-(aminomethyl)-8,9-dihydroxy-4,11b-dimethyltetradecahydro-6a,9-methanocyclohepta[a]naphthalene-4-carboxylate (**23**)

Yield: 0.04 g (58%), white crystals, m.p.: 140–143 °C, [α]${}_{D}^{20}$ = –9 (c 0.365, CHCl_3_). ^1^H NMR (500 MHz, MeOH): δ 0.76–0.81 (4H, m), 0.86–1.03 (3H, m), 1.06 (3H, m), 1.19–1.41 (7H, m), 1.60–2.06 (9H, m), 2.65 (1H, d, *J* = 12.9 Hz), 3.13 (1H, d, *J* = 12.8 Hz), 3.53 (3H, s). ^13^C NMR (125 MHz, MeOH): δ 16.0, 20.3, 21.0, 22.9, 29.2, 35.9, 39.1, 40.6, 41.9, 43.1, 43.1, 45.1, 45.5, 49.1, 51.9, 54.7, 56.2, 58.1, 76.2, 83.3, 178.3. Anal. calcd. for C_21_H_35_NO_4_: C, 69.01; H, 9.65; N, 3.83 Found: C, 69.25; H, 9.44; N, 4.08.

### 4.10. General Procedure for the Reaction of Benzaldehyde with Diethylzinc in the Presence of Chiral Catalysts

To the respective catalyst (0.1 mmol), 1 M Et_2_Zn in *n*-hexane solution (3 mL, 3.0 mmol) was added under an argon atmosphere at room temperature. The solution was stirred for 25 min at room temperature, and then benzaldehyde (1 mmol) was added. After stirring at room temperature for a further 20 h, the reaction was quenched with saturated NH_4_Cl solution (15 mL), and the mixture was extracted with EtOAc (2 × 20 mL). The combined organic phase was washed with H_2_O (10 mL), dried (Na_2_SO_4_), and evaporated under vacuum. The crude secondary alcohols obtained were purified by flash column chromatography (*n*-hexane/EtOAc 4:1). The *ee* and absolute configuration of the resulting material were determined by chiral GC on a Chirasil-DEX CB column after *O*-acetylation in Ac_2_O/DMPA/pyridine [42].

### 4.11. Determination of Antiproliferative Properties

The growth-inhibitory effects of the prepared steviol-based diterpen aminodiols were determined by a standard MTT (3-(4,5-dimethylthiazol-2-yl)-2,5-diphenyltetrazolium bromide) assay on a panel of human gynecological malignant cells, including MDA-MB-231 (breast cancer), Hela and SiHa (cervical cancers), and A2780 (ovarian cancer) [43]. Cell lines were purchased from ECACC (European Collection of Cell Cultures, Salisbury, UK) except the SiHa, which was obtained from ATCC (American Tissue Culture Collection, Manassas, VA, USA). Cells were maintained in minimal essential medium supplemented with 10% fetal bovine serum, 1% non-essential amino acids, and 1% penicillin-streptomycin at 37 °C in a humidified atmosphere containing 5% CO^2^. All media and supplements for these experiments were obtained from Lonza Group Ltd. (Basel, Switzerland).

Cells were seeded into 96-well plates (5000 cells/well) and incubated with the tested compounds at 10 µM and 30 µM under cell-culturing conditions for 72 h. Then, the MTT solution (5 mg/mL) was added to each sample, which were incubated for a further 4 h. The medium was removed and the precipitated formazan crystals were dissolved in DMSO during 60 min of shaking at 37 °C. The absorbance was measured at 545 nm by using a microplate reader, and untreated cells were used as controls. In the case of the most effective compounds, the assays were repeated with a set of dilutions (0.1–30 μM) in order to determine IC^50^ values. Two independent experiments were performed with five wells for each condition. Cisplatin (Ebewe GmbH, Unterach, Austria), a clinically used anticancer agent, was used as a reference agent. Calculations were performed by means of the GraphPad Prism 5.01 software (GraphPad Software Inc., San Diego, CA, USA).

## Figures and Tables

**Table 1:** Ring opening of **4** with primary and secondary amines according to Scheme 4.

Entry	Compound	R^1^	R^2^	Yield (%)
1	**11**	H	Benzyl	67
2	**12**	Me	Benzyl	75
3	**13**	H	(*R*)-α-Methylbenzyl	52
4	**14**	H	(*S*)-α-Methylbenzyl	59
5	**15**	H	*i*-Pr	57
6	**16**	Et	Et	63
7	**17**	H	Propargyl	72
8	**18**	H	3,5-*bis*(trifluoromethyl)benzyl	32

**Table 2:** Addition of diethylzinc to benzaldehyde, catalyzed by aminodiol derivatives.

Entry	Ligand	Yield ^a^ (%)	*ee*^b^ (%)	Major Configuration ^c^
1	**11**	78	23	(*R*)
2	**12**	85	52	(*R*)
3	**17**	77	33	(*R*)
4	**18**	85	31	(*R*)
5	**19**	79	30	(*R*)

^a^ After silica column chromatography. ^b^ Determined using the crude product by GC analysis (Chirasil-DEX CB column). ^c^ Determined by comparing the t_R_ of GC analysis and optical rotations with literature data [10,42].

**Table 3:** Antiproliferative activities of the tested diterpene analogs.

Compound	Conc (μM)	Growth Inhibition (%) ± SEM Calculated IC_50_ (μM)			
		A2780	HeLa	SiHa	MDA-MB-231
**5**	10	<20 *	<20	<20	<20
	30	<20	<20	26.81 ± 2.02	<20
**6**	10	<20	<20	<20	<20
	30	<20	<20	<20	<20
**7**	10	<20	27.07 ± 1.12	<20	<20
	30	38.38 ± 2.53	34.72 ± 0.32	<20	<20
**8**	10	49.78 ± 1.28	43.19 ± 2.20	96.41 ± 0.41	<20
	30	85.21 ± 0.53	51.98 ± 2.65	96.55 ± 0.30	52.04 ± 0.85
		10.18	21.89	4.64	29.90
**9**	10	22.24 ± 1.36	<20	<20	<20
	30	49.35 ± 0.58	26.80 ± 0.62	28.85 ± 0.69	34.62 ± 3.14
**10**	10	<20	25.61 ± 3.14	<20	<20
	30	48.15 ± 0.68	28.46 ± 1.98	<20	36.30 ± 3.34
**11**	10	54.36 ± 3.34	37.41 ± 0.57	<20	<20
	30	99.24 ± 0.16	98.58 ± 0.17	96.42 ± 0.44	98.45 ± 0.06
		6.68	9.37	24.68	26.16
**12**	10	35.57 ± 2.31	39.90 ± 2.76	<20	<20
	30	65.79 ± 3.17	52.72 ± 2.04	<20	23.77 ± 1.59
		17.34	23.49		
**13**	10	84.73 ± 0.84	60.46 ± 1.65	92.54 ± 0.77	94.09 ± 0.59
	30	98.75 ± 0.17	98.81 ± 0.10	96.89 ± 0.93	98.30 ± 0.24
		4.19	4.79	6.07	4.32
**14**	10	89.99 ± 1.16	92.25 ± 0.99	91.23 ± 0.89	97.00 ± 0.16
	30	98.87 ± 0.19	98.59 ± 0.07	94.34 ± 0.62	98.27 ± 0.20
		4.91	3.96	6.54	4.39
**15**	10	<20	<20	<20	<20
	30	<20	39.18 ± 1.84	20.41 ± 2.30	<20
**16**	10	<20	<20	<20	<20
	30	29.07 ± 1.42	27.44 ± 1.06	20.83 ± 2.29	<20
**17**	10	20.75 ± 0.77	<20	<20	<20
	30	43.94 ± 2.99	30.21 ± 0.96	<20	26.32 ± 1.04
**18**	10	72.12 ± 1.13	69.15 ± 2.86	59.69 ± 1.52	97.68 ± 0.13
	30	99.14 ± 0.12	98.25 ± 0.22	91.88 ± 1.34	98.26 ± 0.23
		6.25	5.73	7.84	4.76
**19**	10	98.53 ± 0.16	98.72 ± 0.11	96.50 ± 0.32	97.56 ± 0.42
	30	98.97 ± 0.09	98.87 ± 0.03	97.06 ± 0.31	98.48 ± 0.40
		1.07	1.05	1.62	1.25
**20**	10	47.33 ± 0.91	42.91 ± 1.19	<20	<20
	30	98.83 ± 0.23	98.95 ± 0.22	94.82 ± 0.05	95.81 ± 0.24
		9.78	10.39	14.95	15.09
**21**	10	<20	<20	<20	<20
	30	<20	<20	<20	<20
**22**	10	52.78 ± 2.29	56.41 ± 0.96	86.27 ± 1.83	83.98 ± 0.41
	30	99.08 ± 0.06	99.01 ± 0.72	90.88 ± 1.03	98.09 ± 0.13
		8.60	4.13	8.58	6.58
**23**	10	<20	23.98 ± 2.06	20.95 ± 1.64	<20
	30	<20	44.56 ± 1.21	28.13 ± 0.75	89.42 ± 1.00
**24**	10	<20	20.53 ± 0.36	<20	<20
	30	22.70 ± 0.56	28.92 ± 0.53	<20	<20
Cisplatin	10	83.57 ± 1.21	42.61 ± 2.33	88.64 ± 0.50	67.51 ± 1.01
	30	95.02 ± 0.28	99.93 ± 0.26	90.18 ± 1.78	87.75 ± 1.10
		1.30	12.43	7.84	3.74

* Cell growth inhibition values less than 20% were considered negligible and are not given numerically.

## Supplementary Materials

Supplementary materials can be found at https://www.mdpi.com/1422-0067/21/1/184/s1.

## Abbreviations

Et_2_O	Diethyl ether
EtOH	Ethanol
HCHO	Formaldehyde
EtOAc	Ethyl acetate
*t*-BuOOH	*tert*-Butyl hydroperoxide
VO(acac)_2_	Vanadyl acetylacetonate
DCM	Dichloromethane
MeCN	Acetonitrile
THF	Tetrahydrofuran
DMDO	Dimethyldioxirane
Et_2_Zn	Diethylzinc
