# Supplementary material for: Stereoselective Synthesis and Antiproliferative Activity of Steviol-Based Diterpen Aminodiols
Source: Int J Mol Sci. 2019 Dec 26;21(1):184. doi: 10.3390/ijms21010184 (PMC6981646; doi:10.3390/ijms21010184)

Supporting informations
for
Stereoselective synthesis and cytoselective toxicity of steviol-based diterpen aminodiols

Dániel Ozsvár [1], Viktória Nagy [2], István Zupkó [2,3] and Zsolt Szakonyi [1,3*]

[1] Institute of Pharmaceutical Chemistry, University of Szeged, Interdisciplinary Excellent Center, H-6720 Szeged, Eötvös utca 6, Hungary; daniel.ozsvar@pharm.u-szeged.hu
[2] Department of Pharmacodynamics and Biopharmacy, University of Szeged, H-6720 Szeged, Eötvös utca 6, Hungary; zupko@pharm.u-szeged.hu, nagy.viktoria@pharm.u-szeged.hu
[3] Interdisciplinary Centre of Natural Products, University of Szeged, H-6720 Szeged, Eötvös utca 6, Hungary
* Correspondence: szakonyi@pharm.u-szeged.hu; Tel.: +36-62-546809; Fax: +36-62-545705

Contents

^{1}H, ^{13}C NMR, COSY, NOESY, HSQC, HMBC spectra of new compounds 3 - 111

^{1}H-NMR of compound **5**

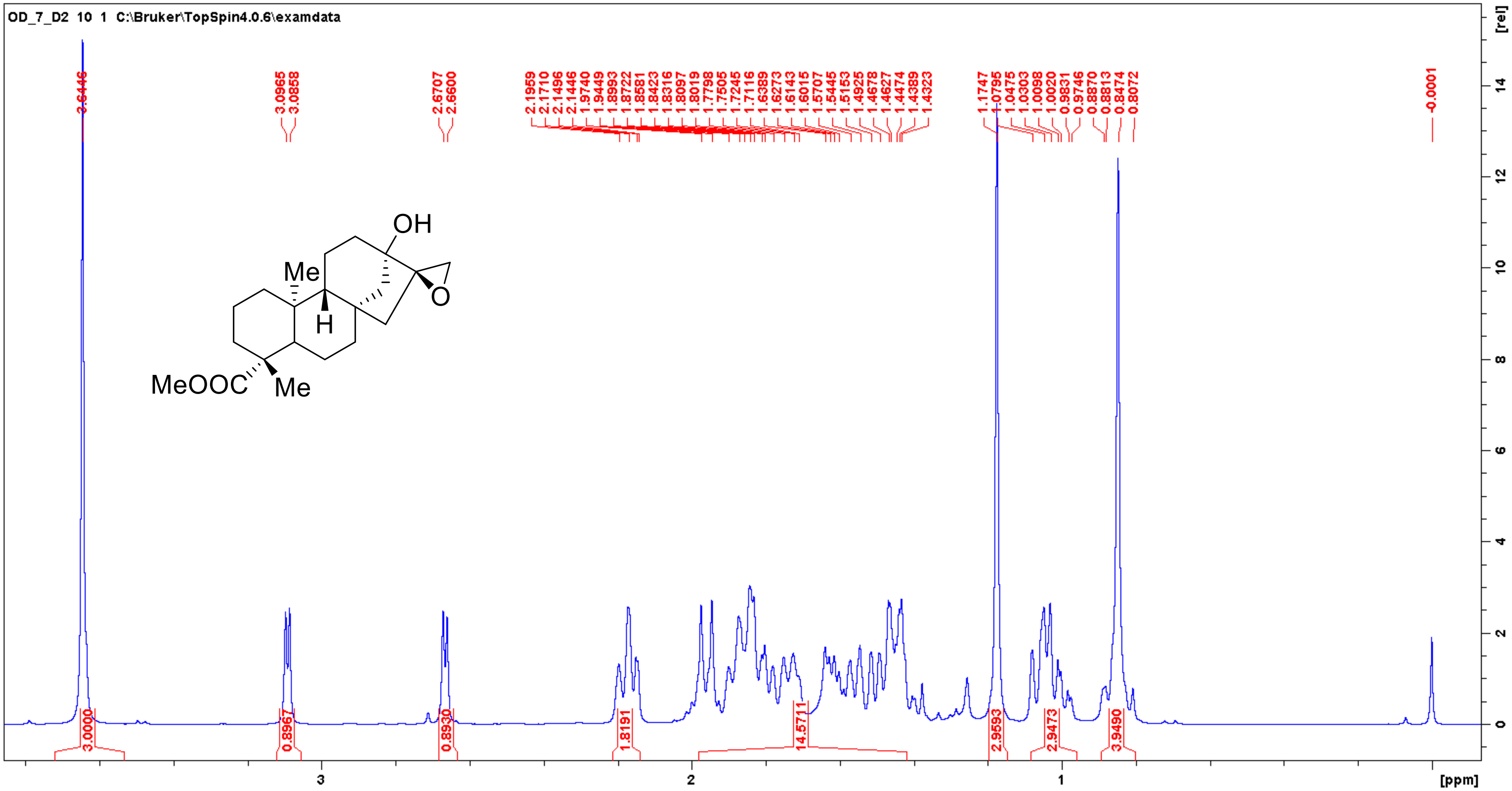

^{13}C-NMR of compound **5**

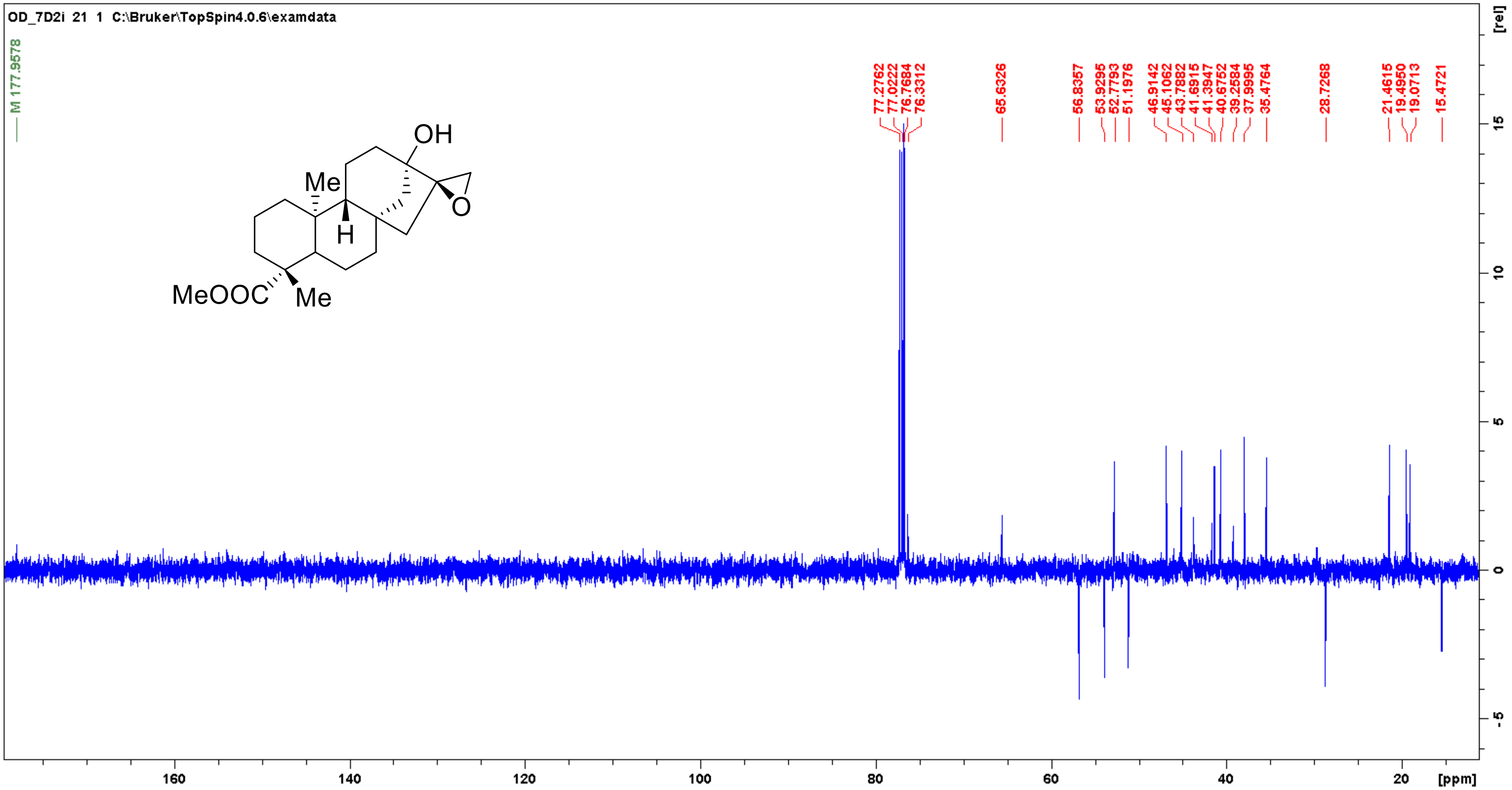

COSY of compound **5**

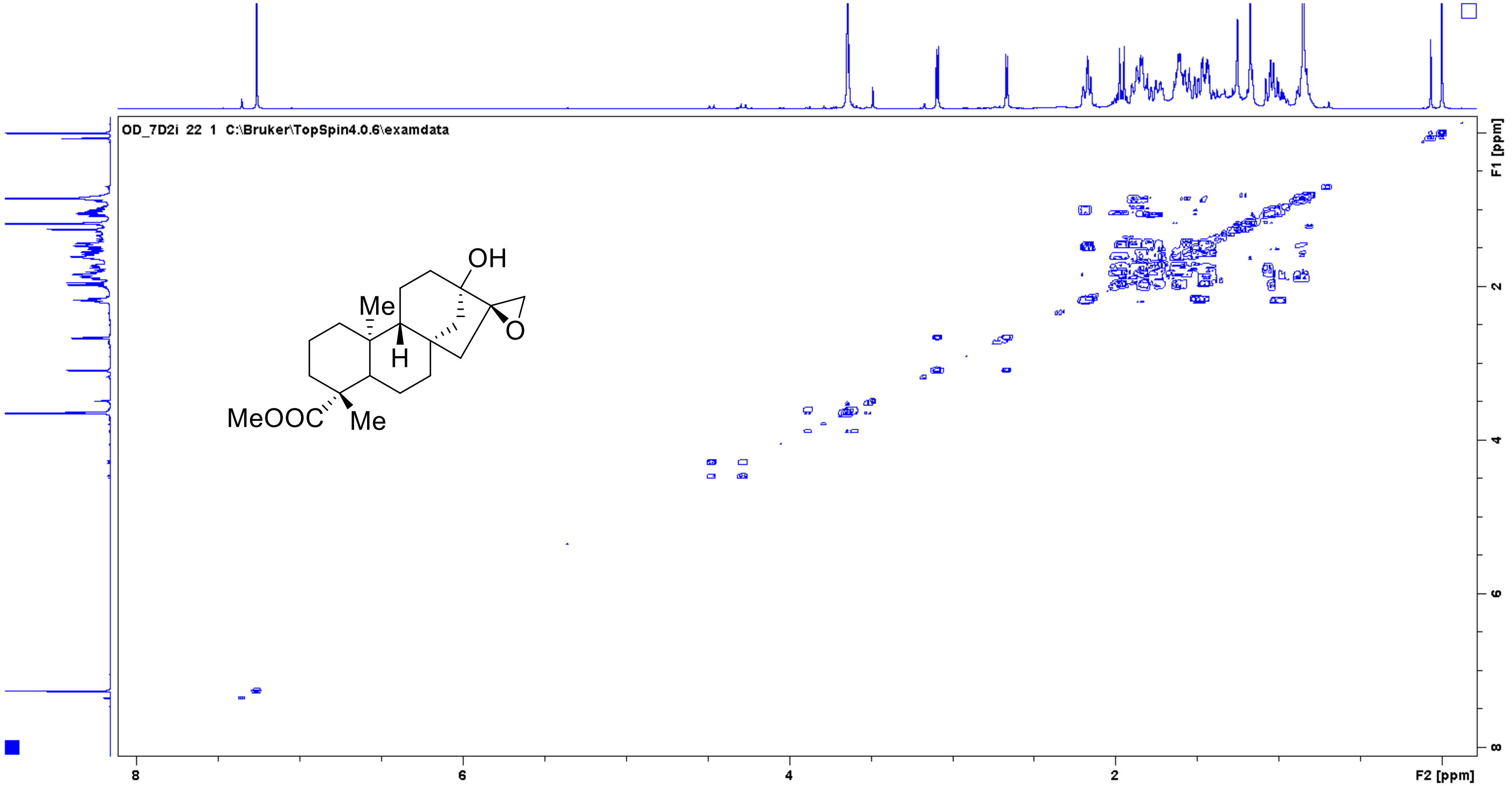

NOESY of compound **5**

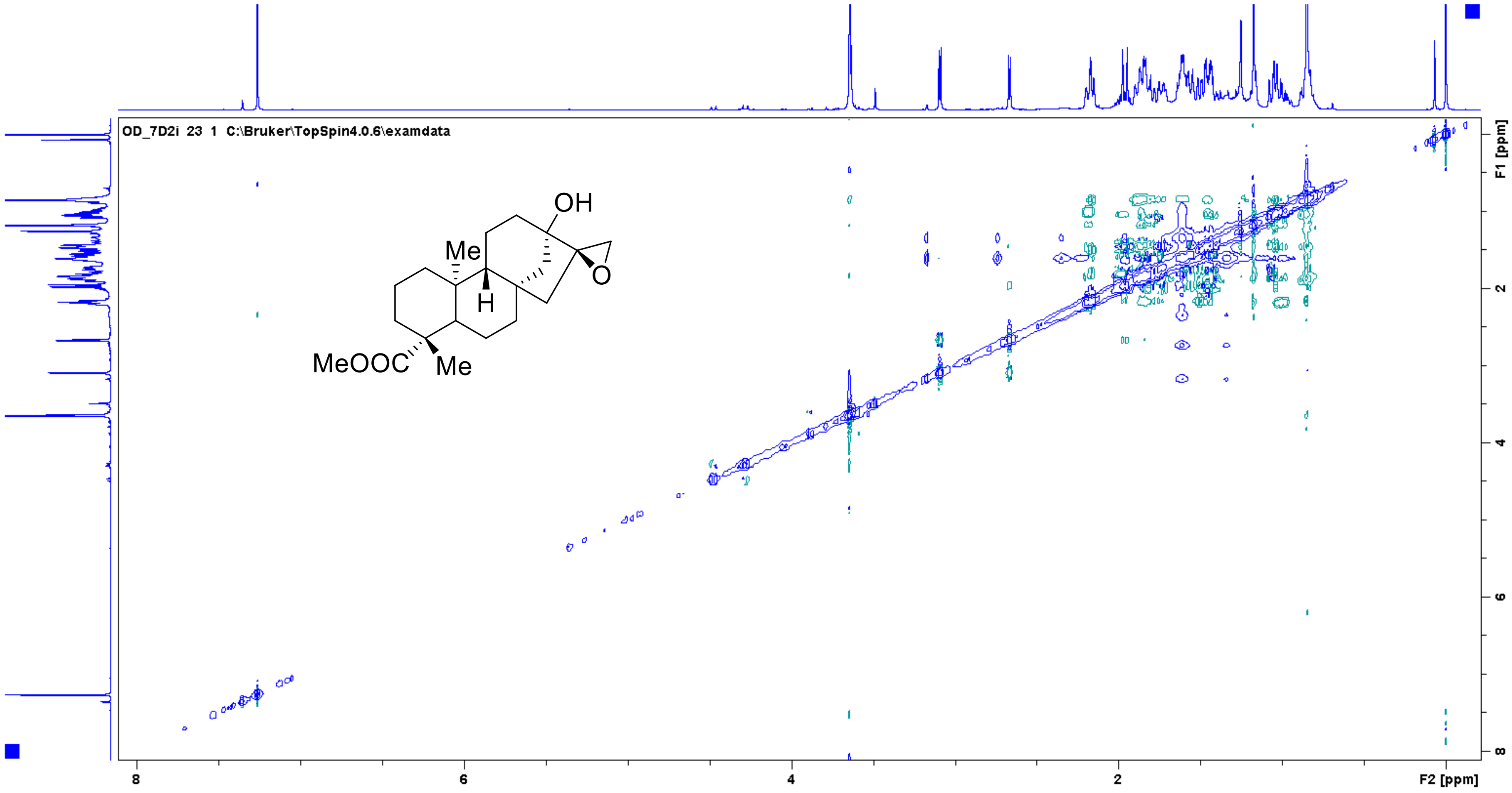

HSQC of compound **5**

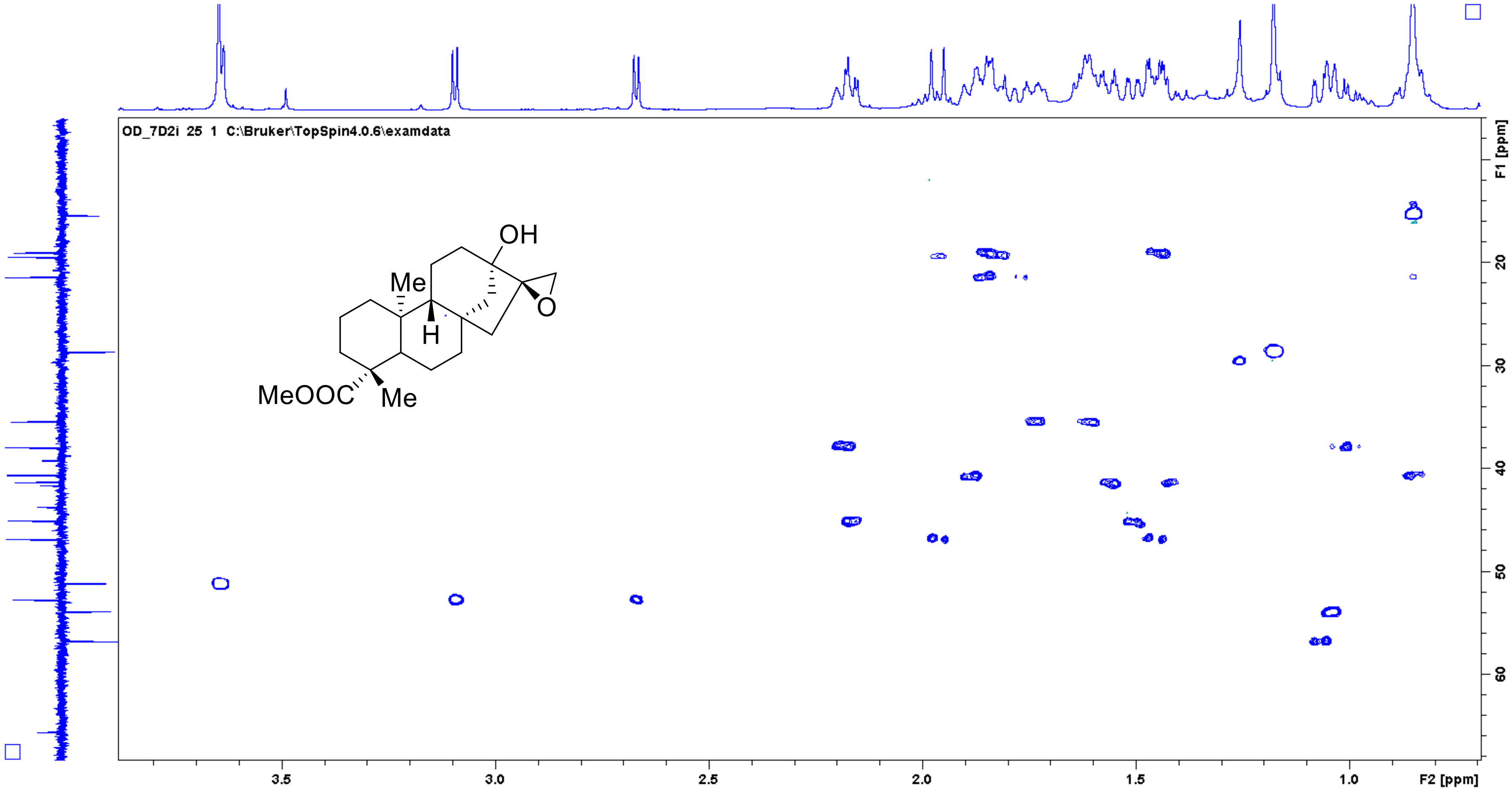

HMBC of compound **5**

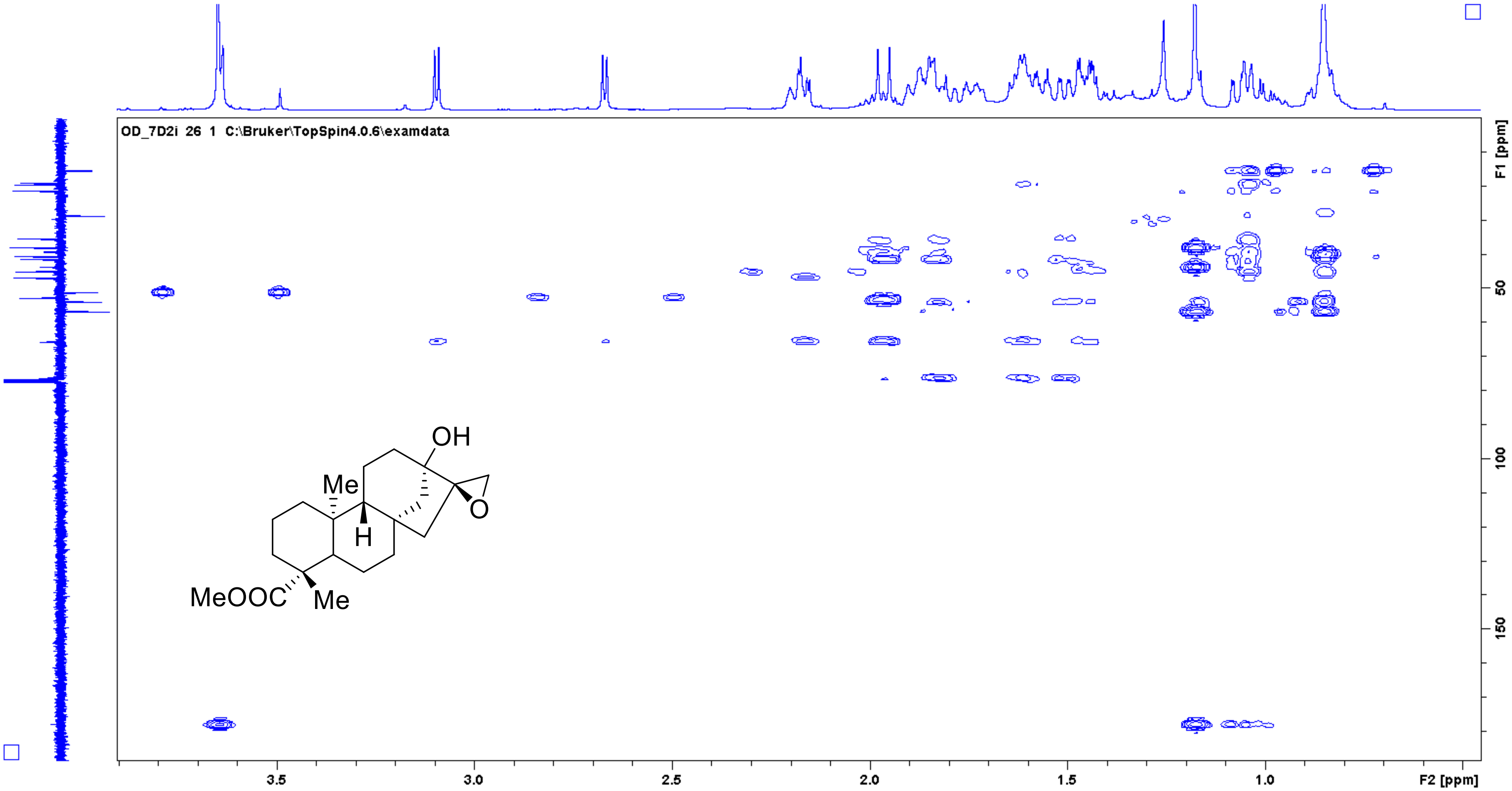

^{1}H-NMR of compound **6**

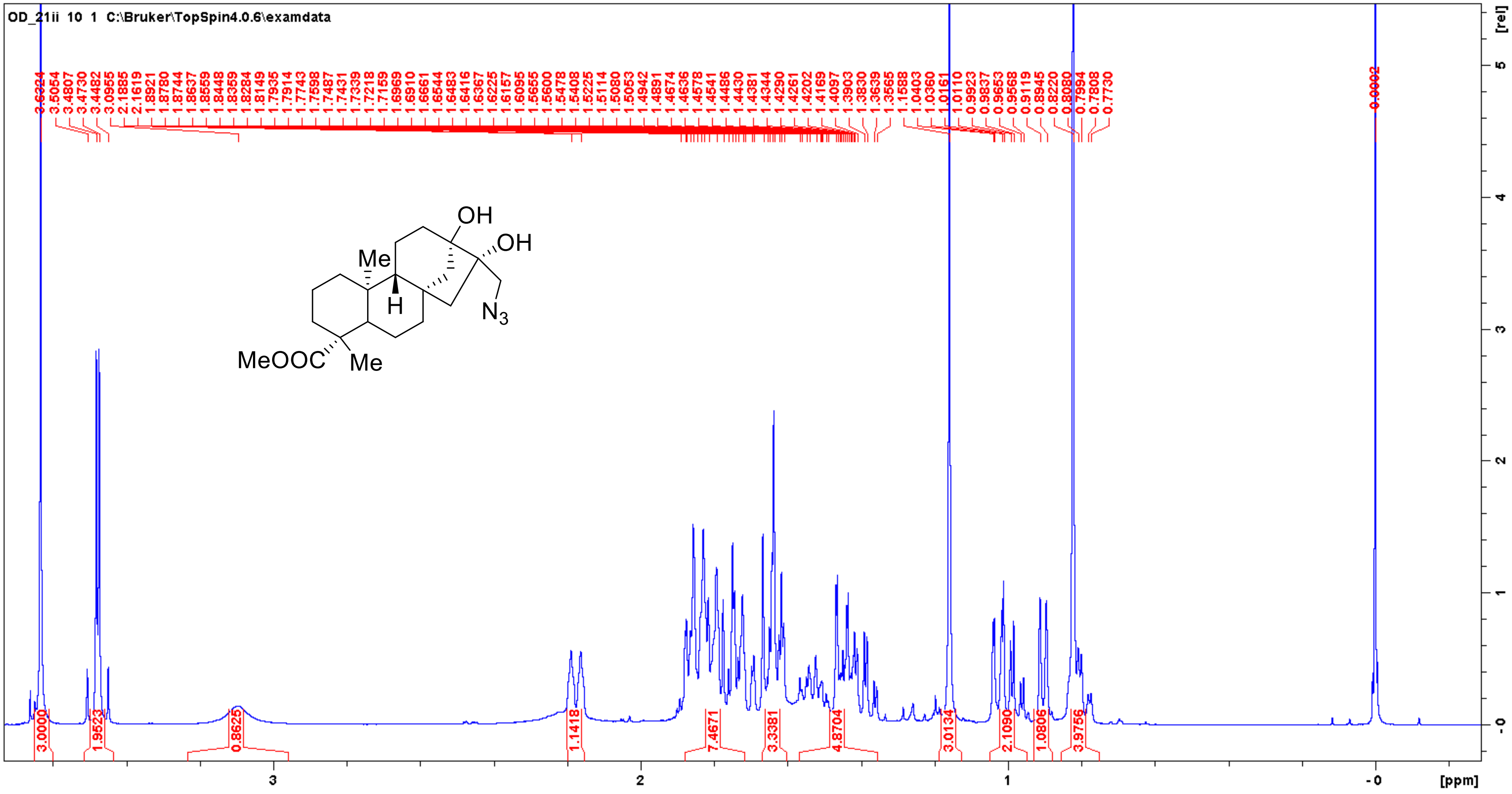

^{13}C-NMR of compound **6**

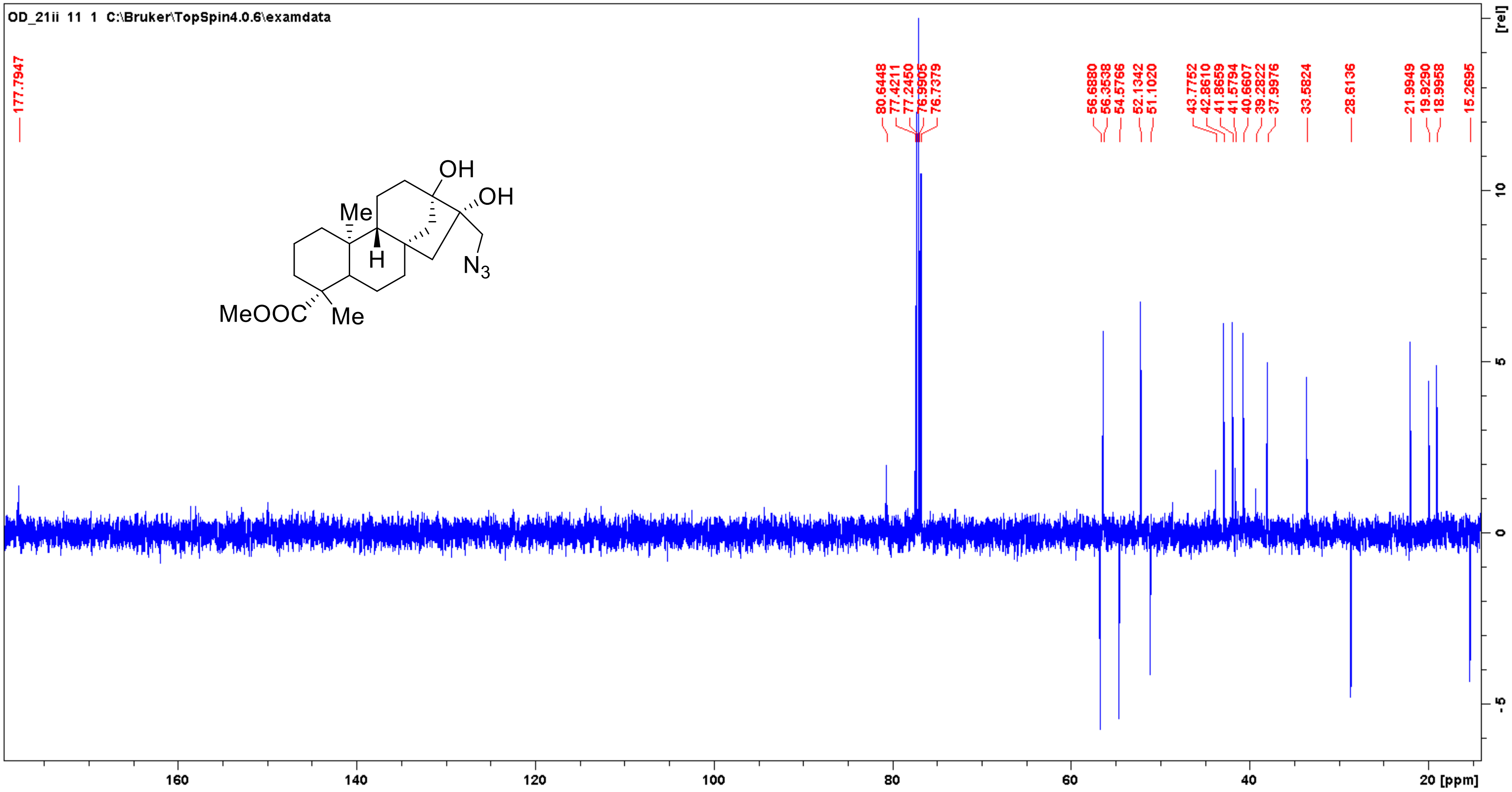

COSY of compound **6**

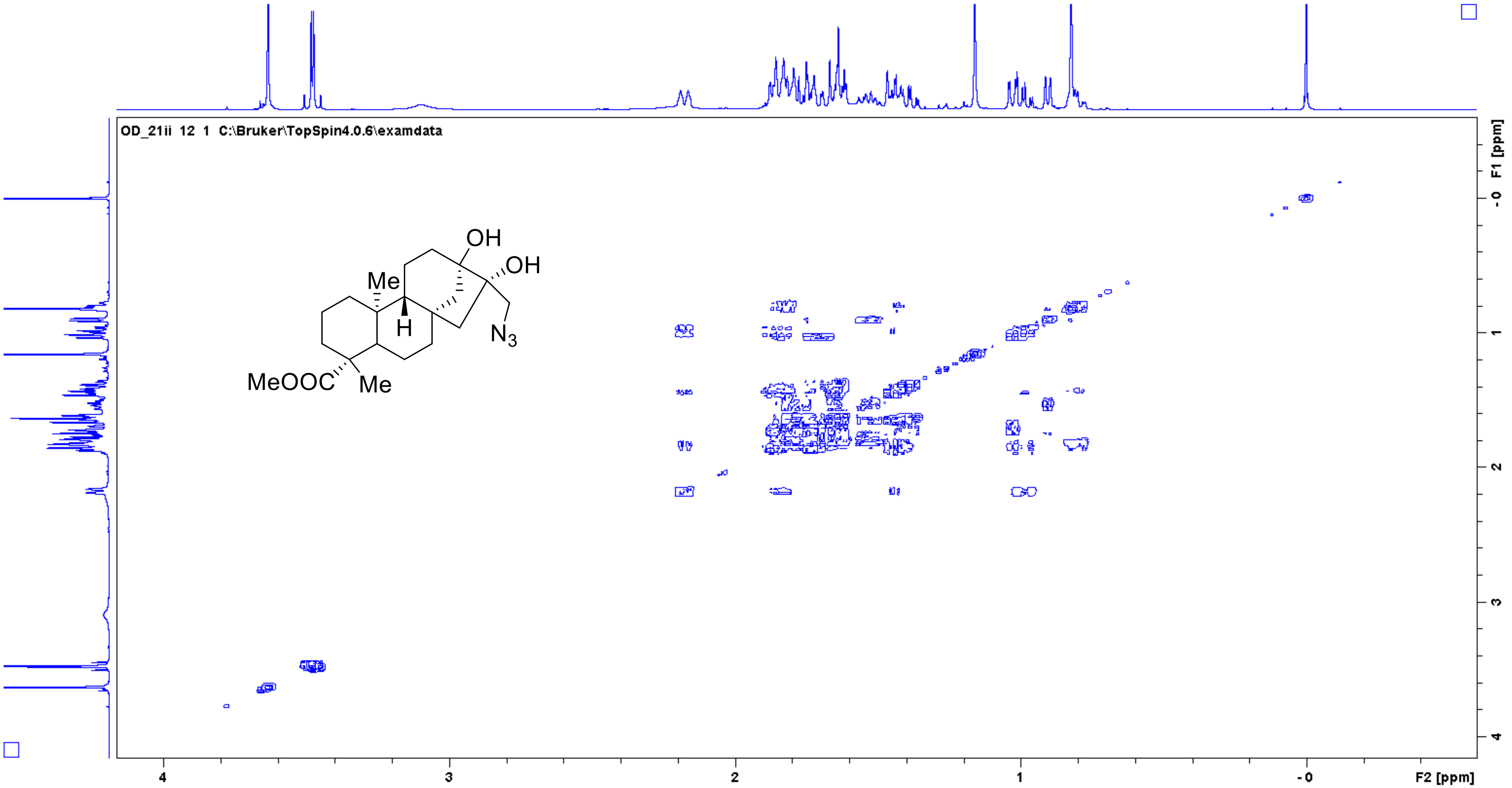

NOESY of compound **6**

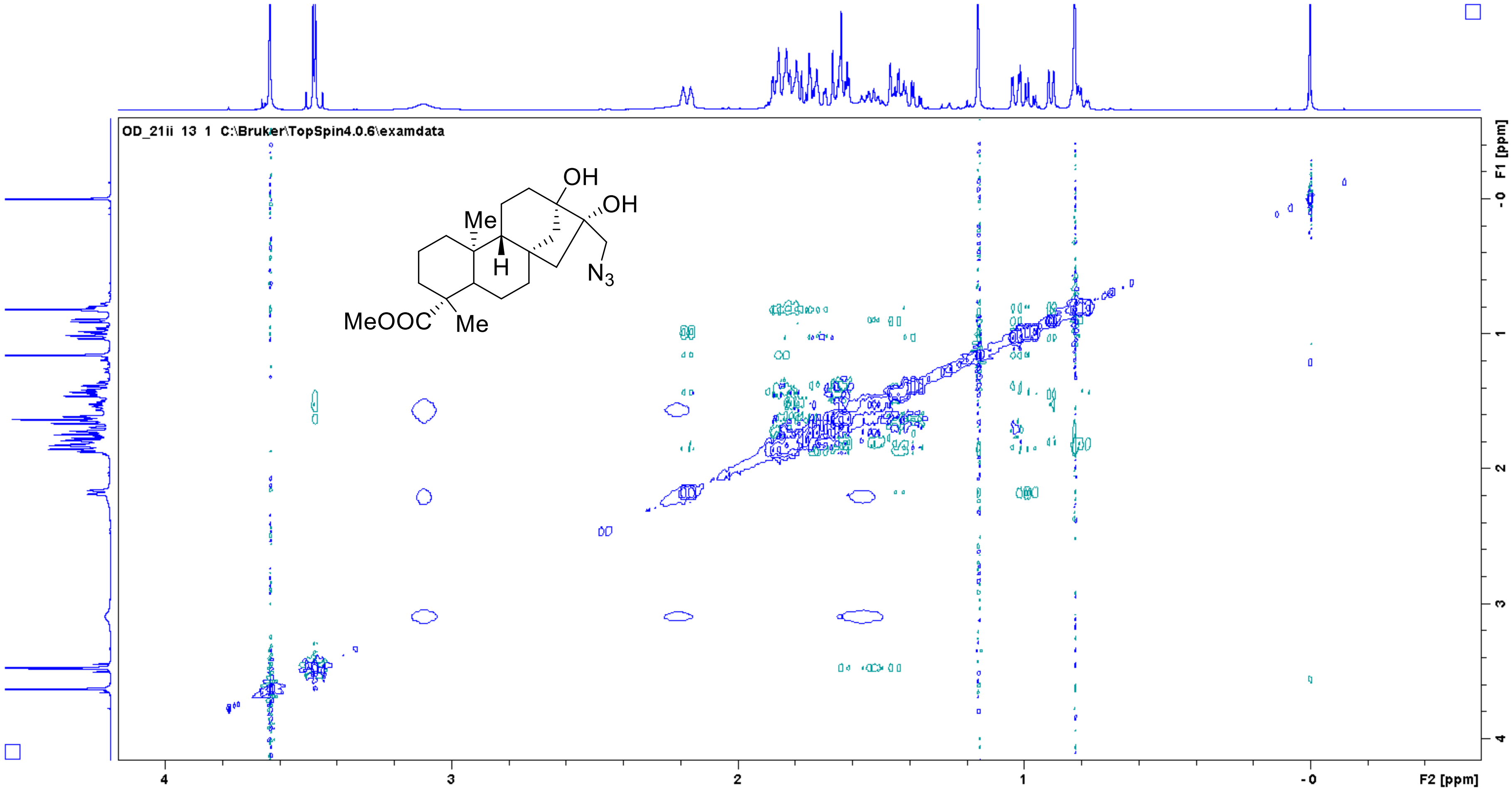

HSQC of compound **6**

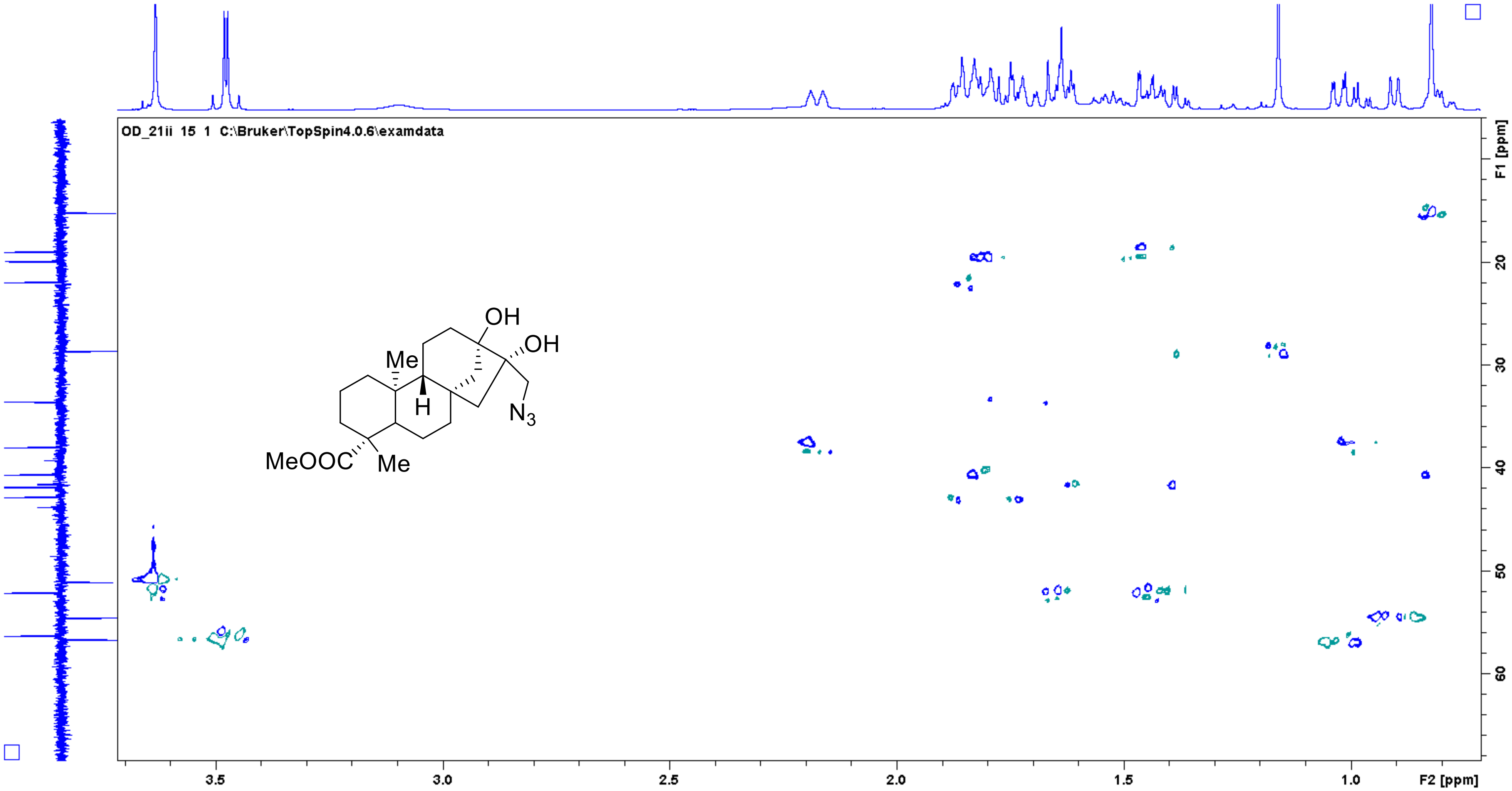

HMBC of compound **6**

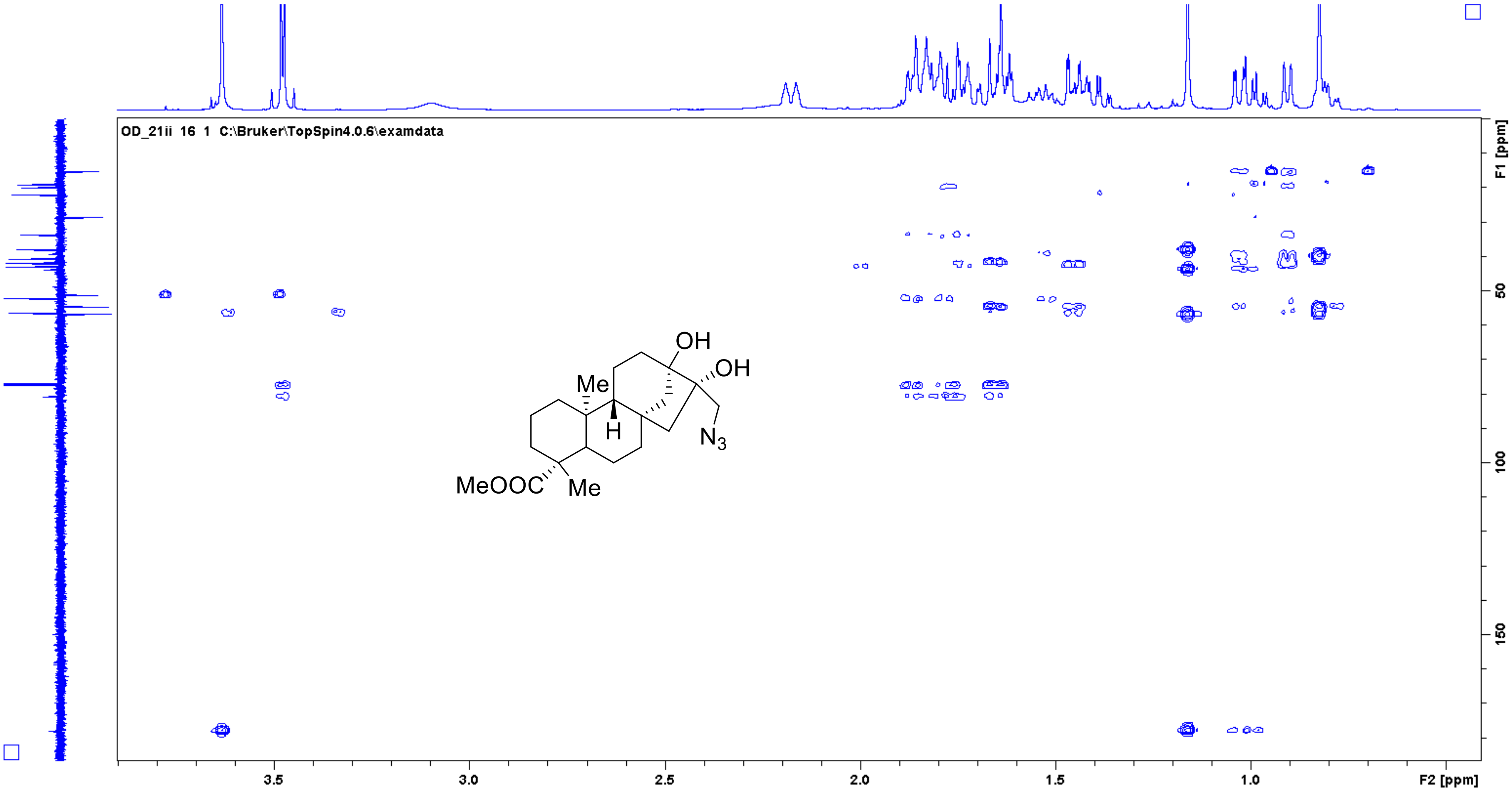

^{1}H-NMR of compound **7**

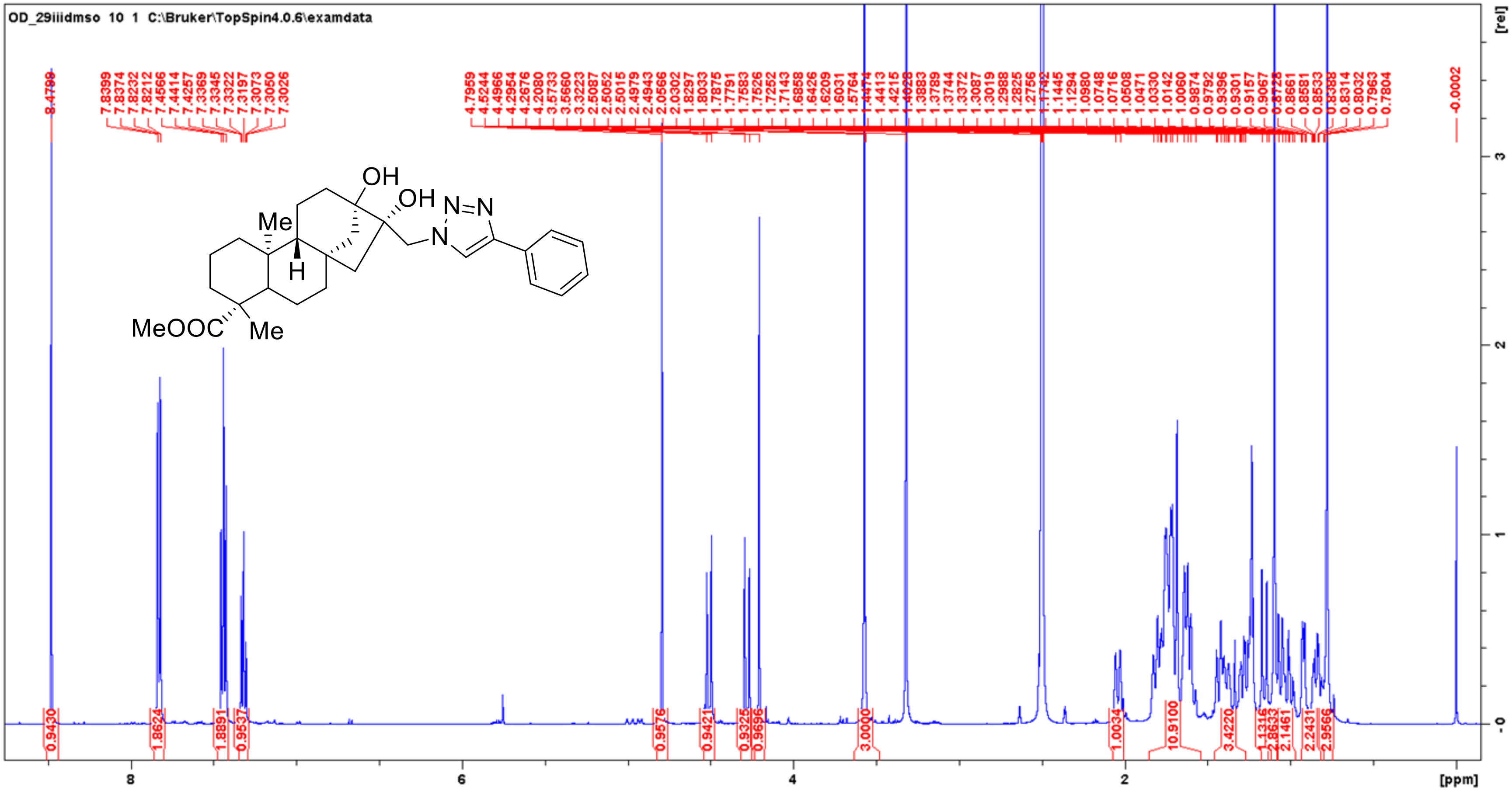

^{13}C-NMR of compound **7**

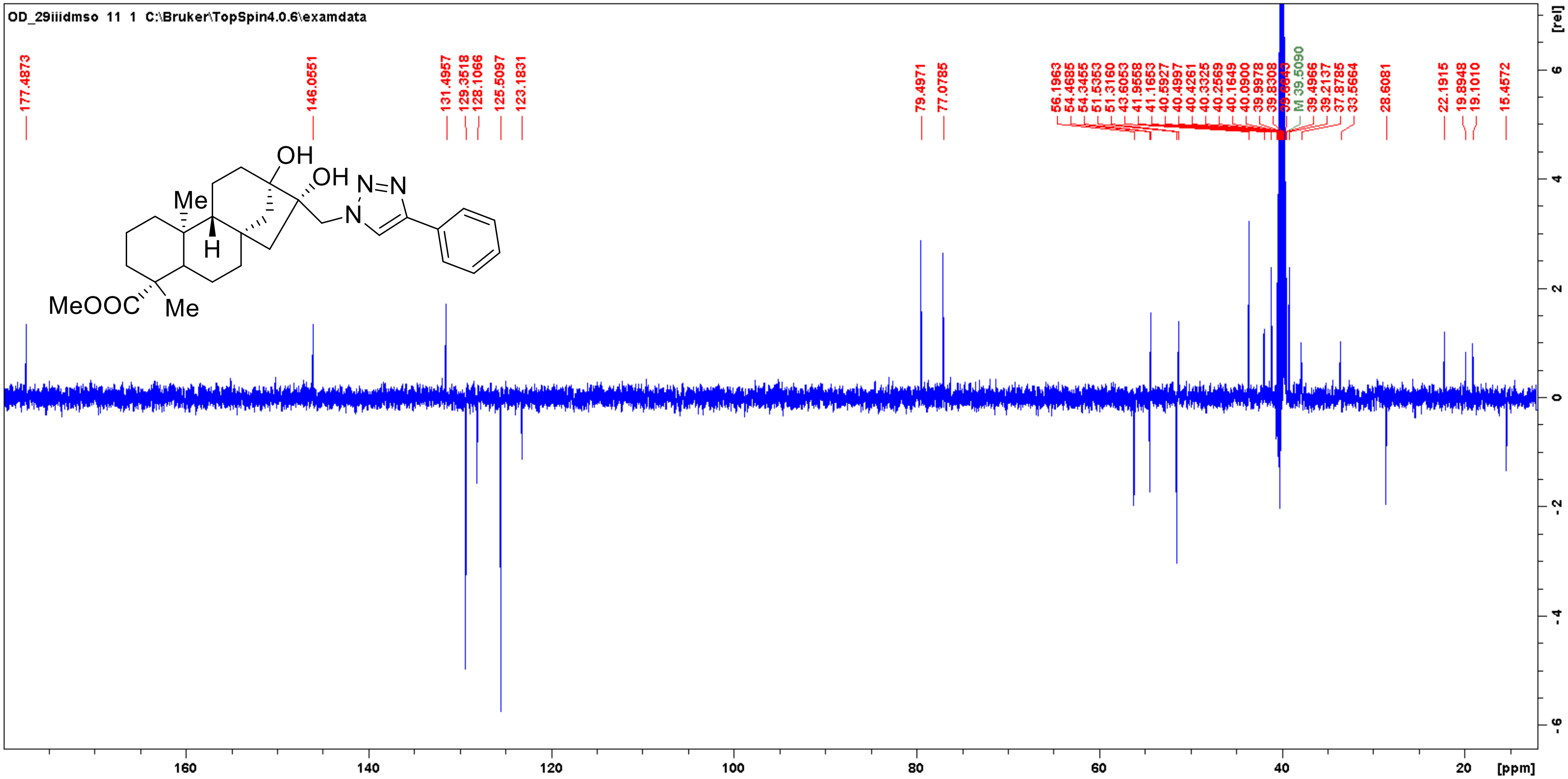

COSY of compound **7**

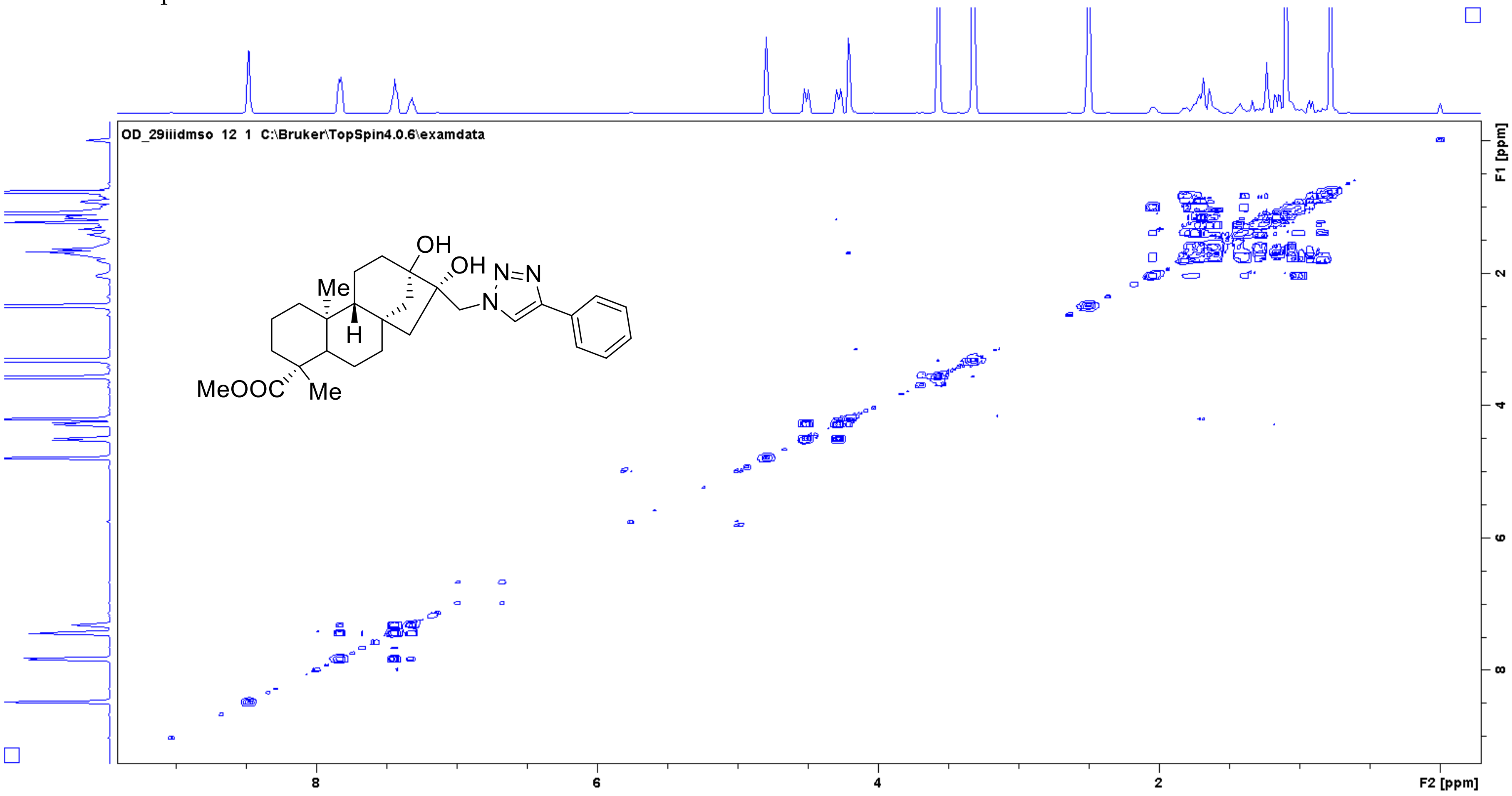

HSQC of compound **7**

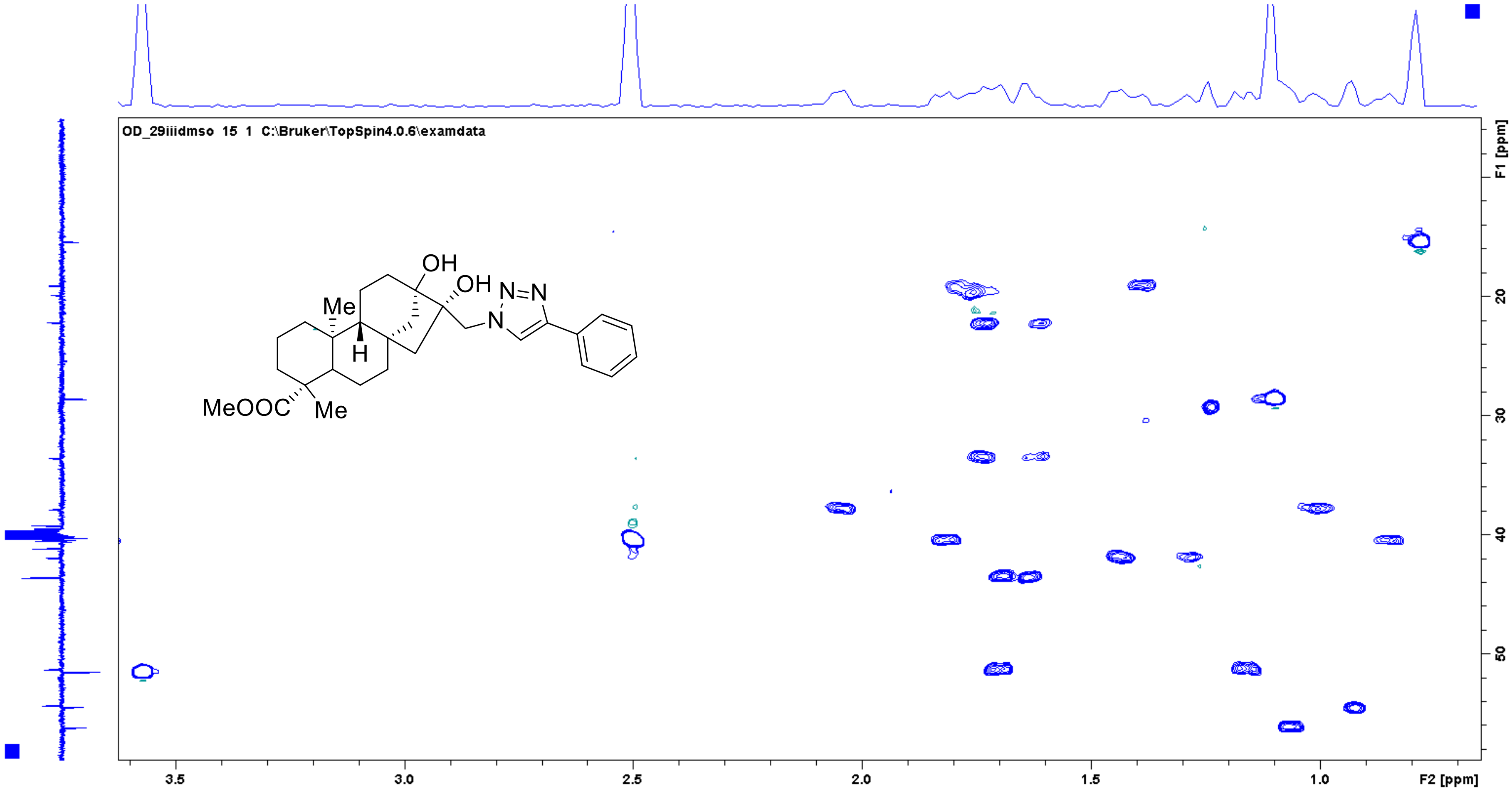

HMBC of compound **7**

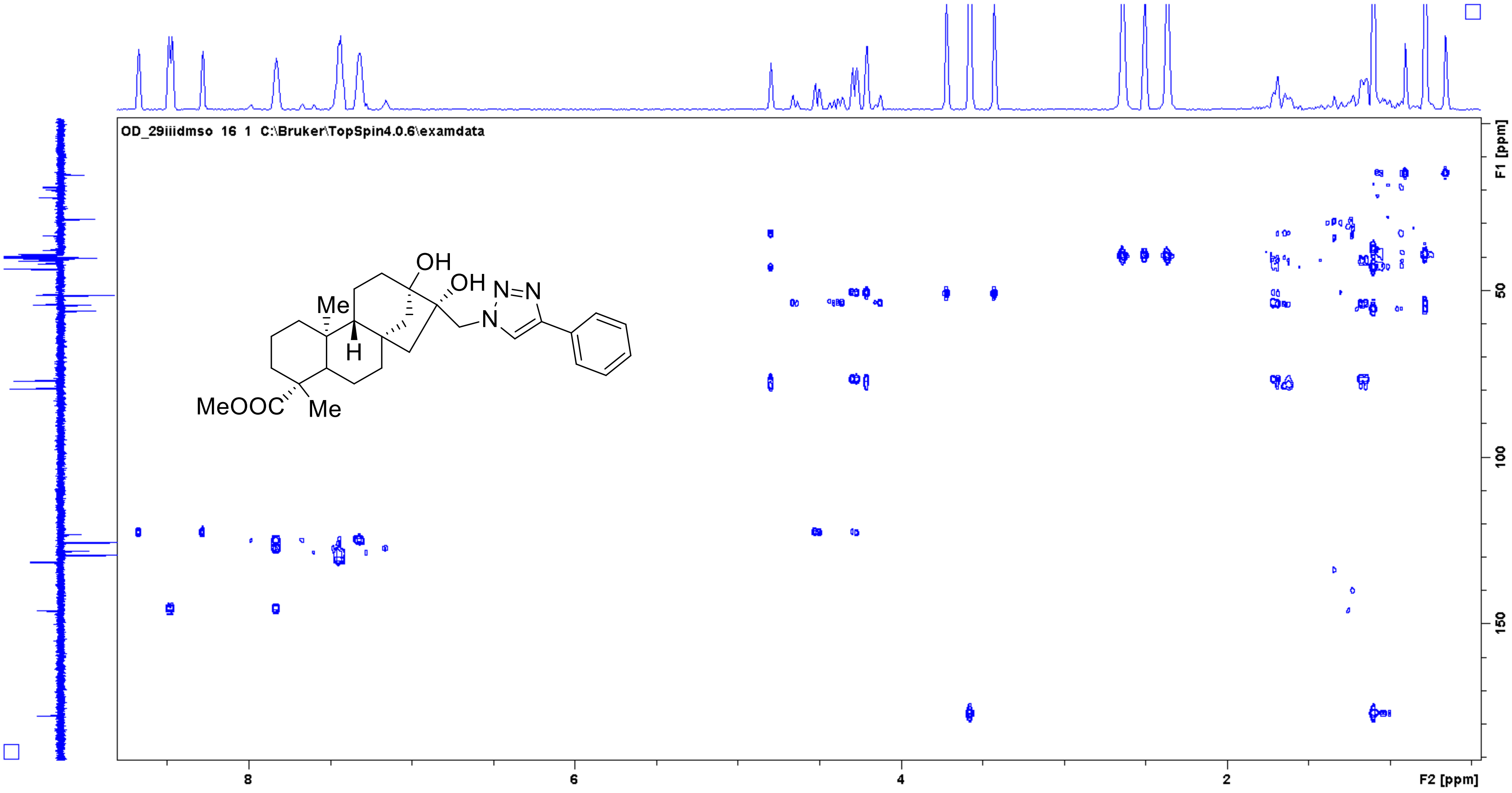

^{1}H-NMR of compound **8**

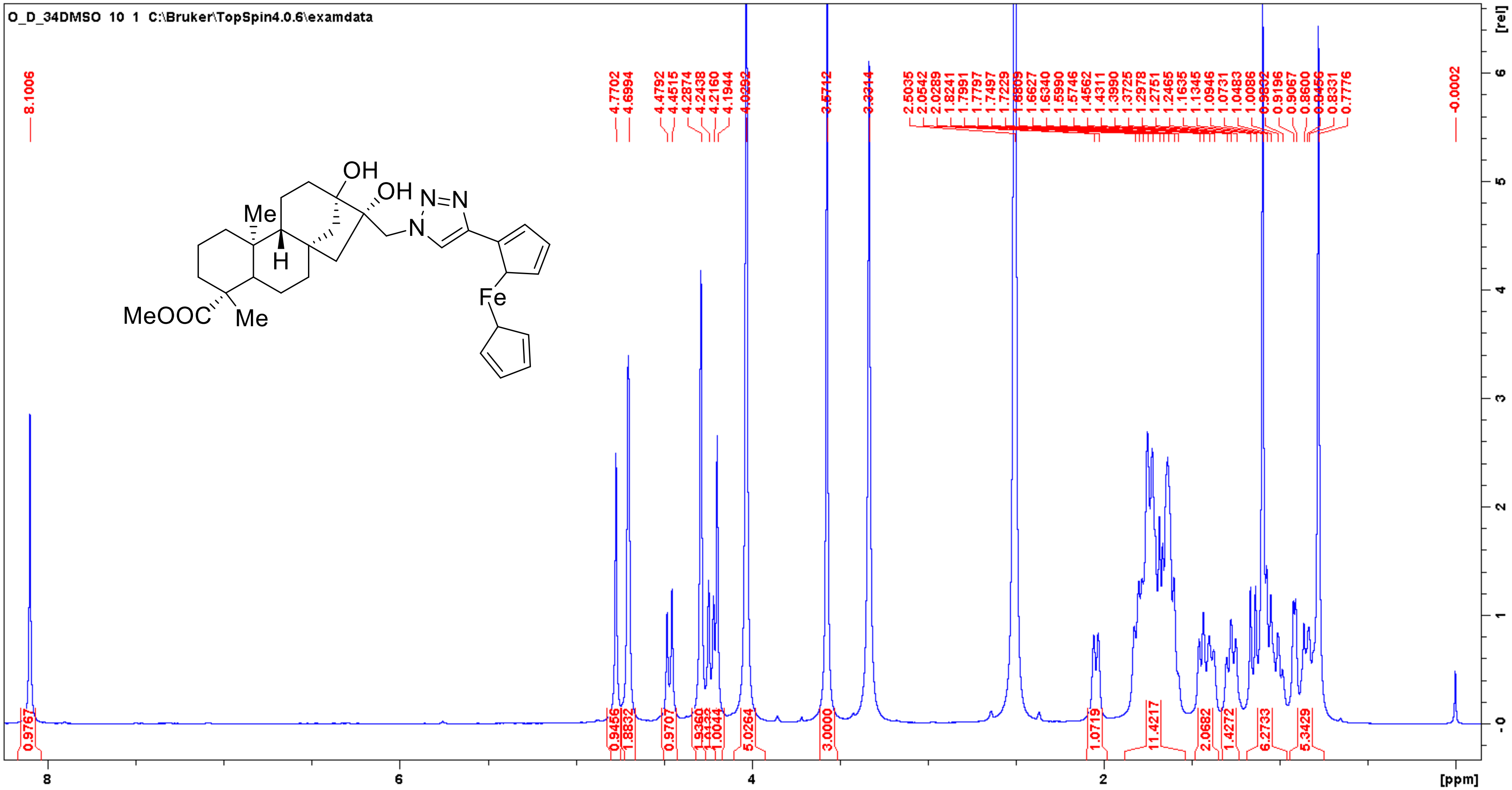

^{13}C-NMR of compound **8**

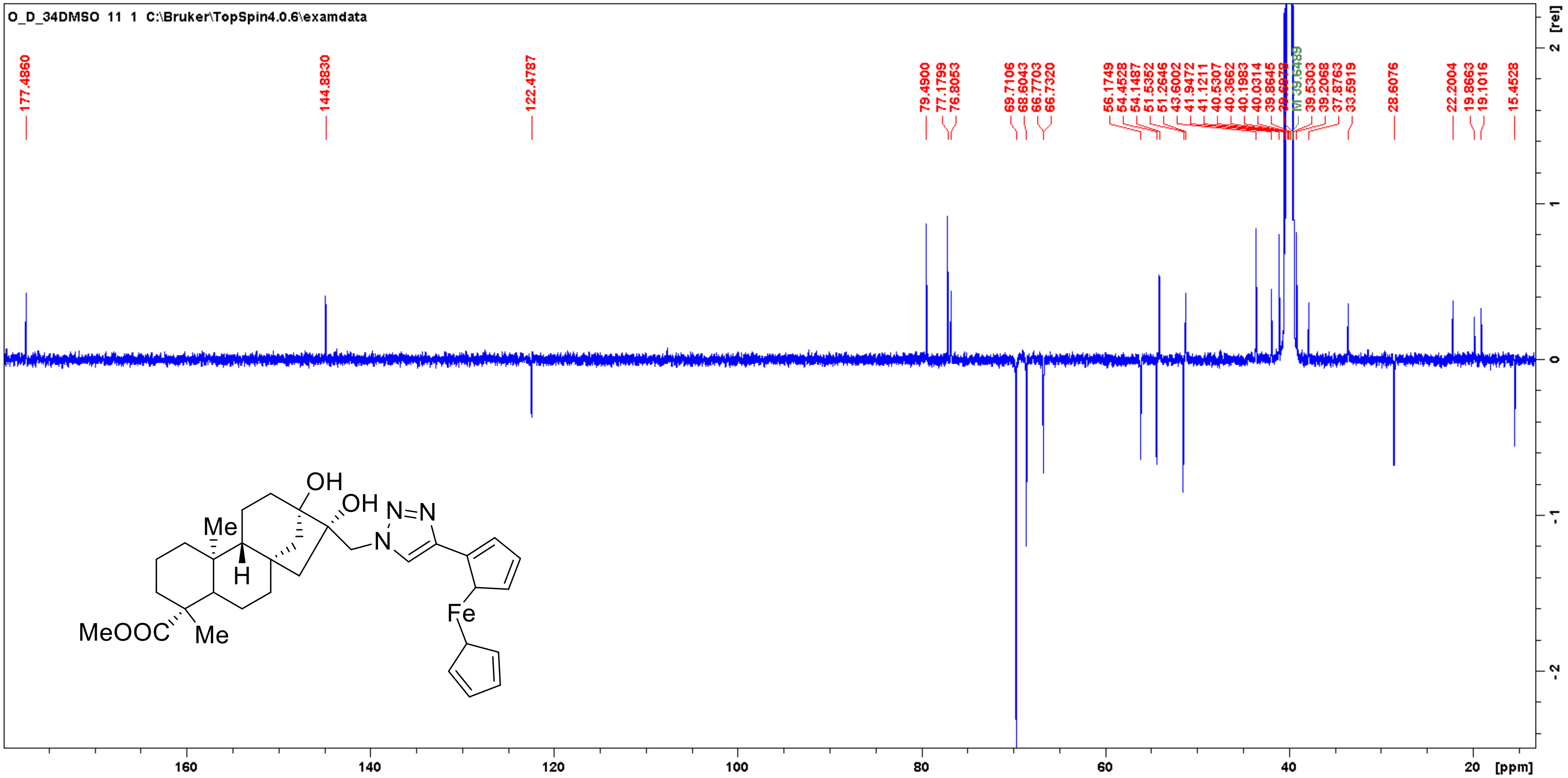

COSY of compound **8**

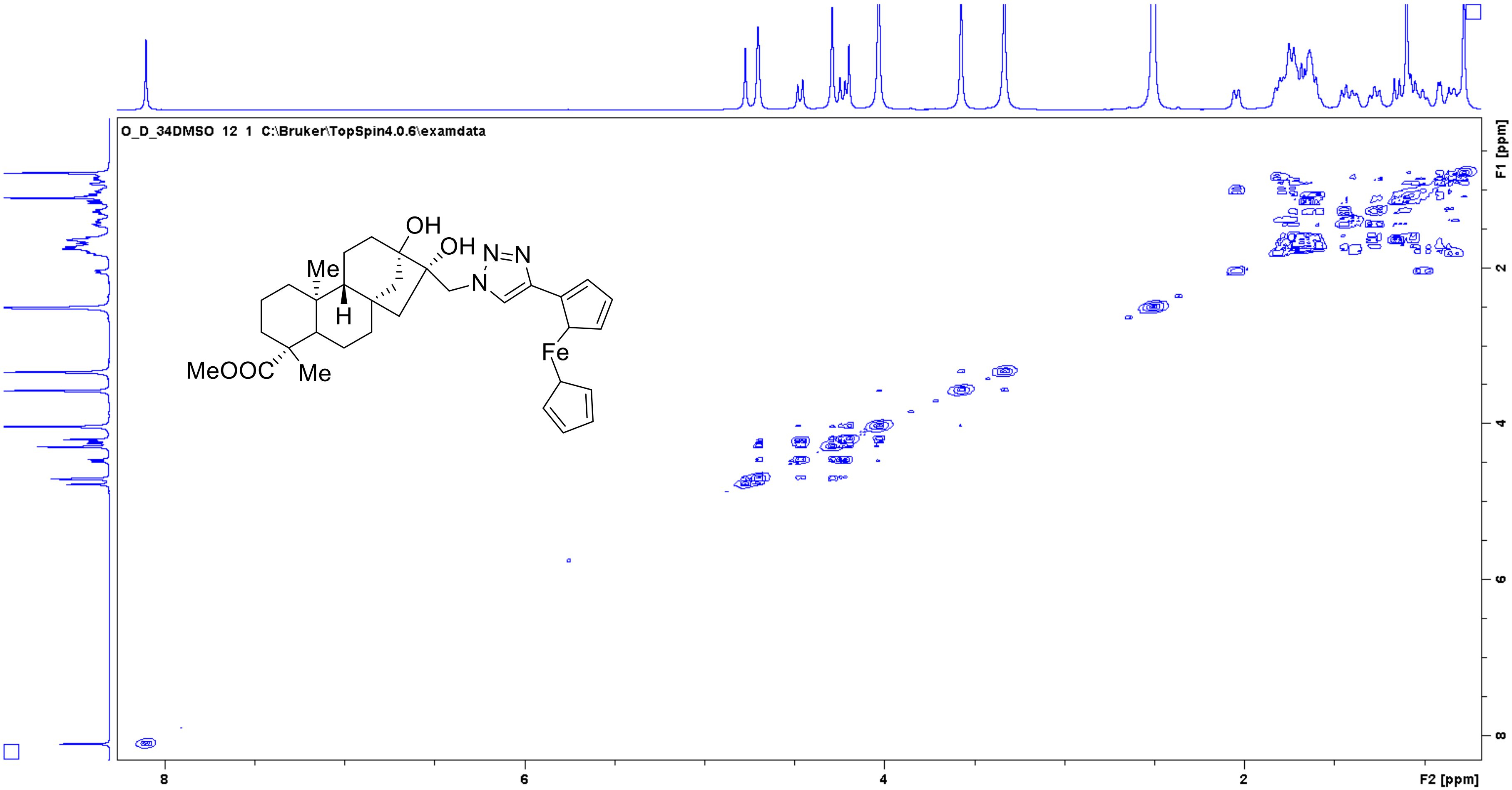

HSQC of compound **8**

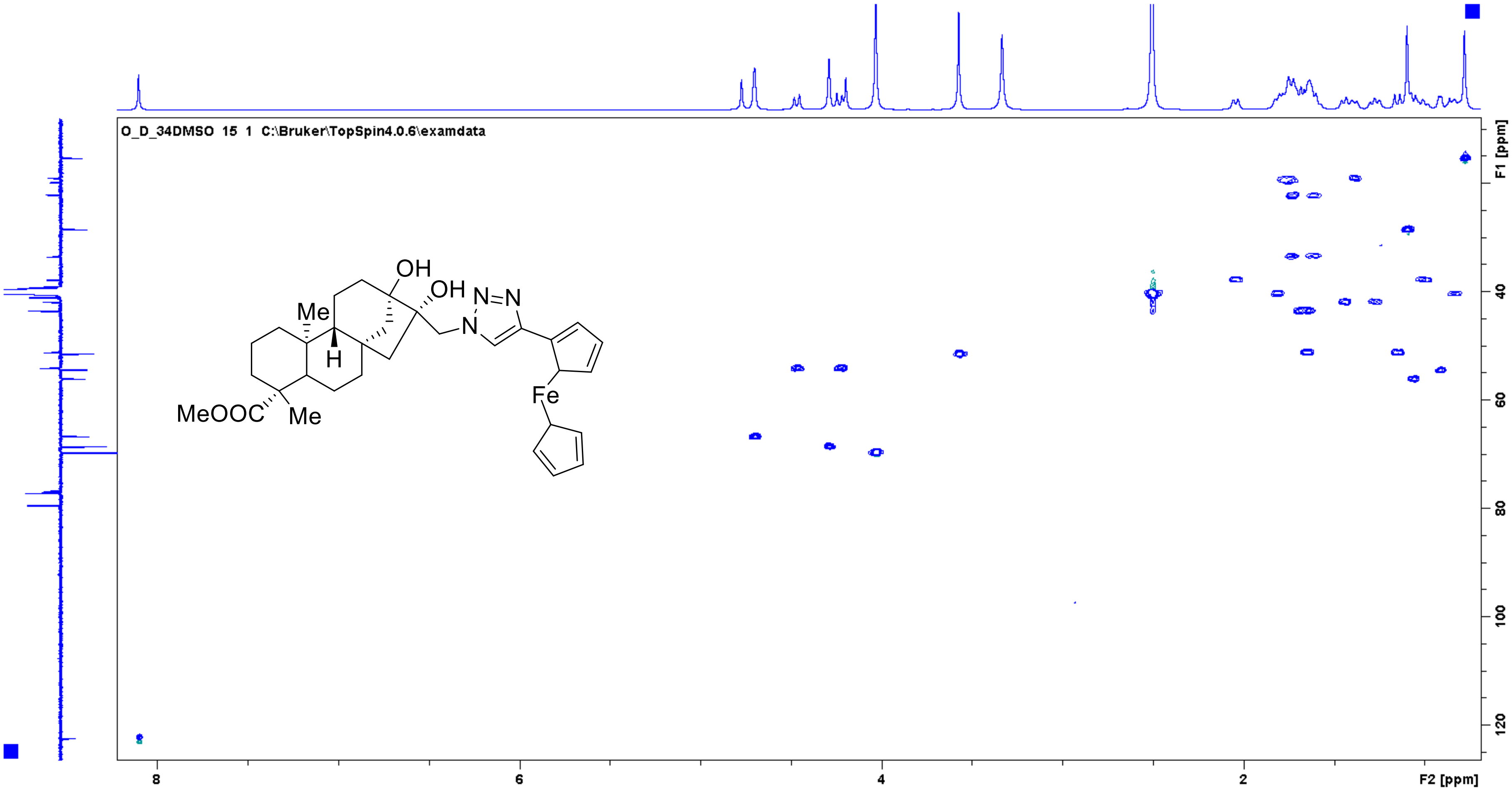

HMBC of compound **8**

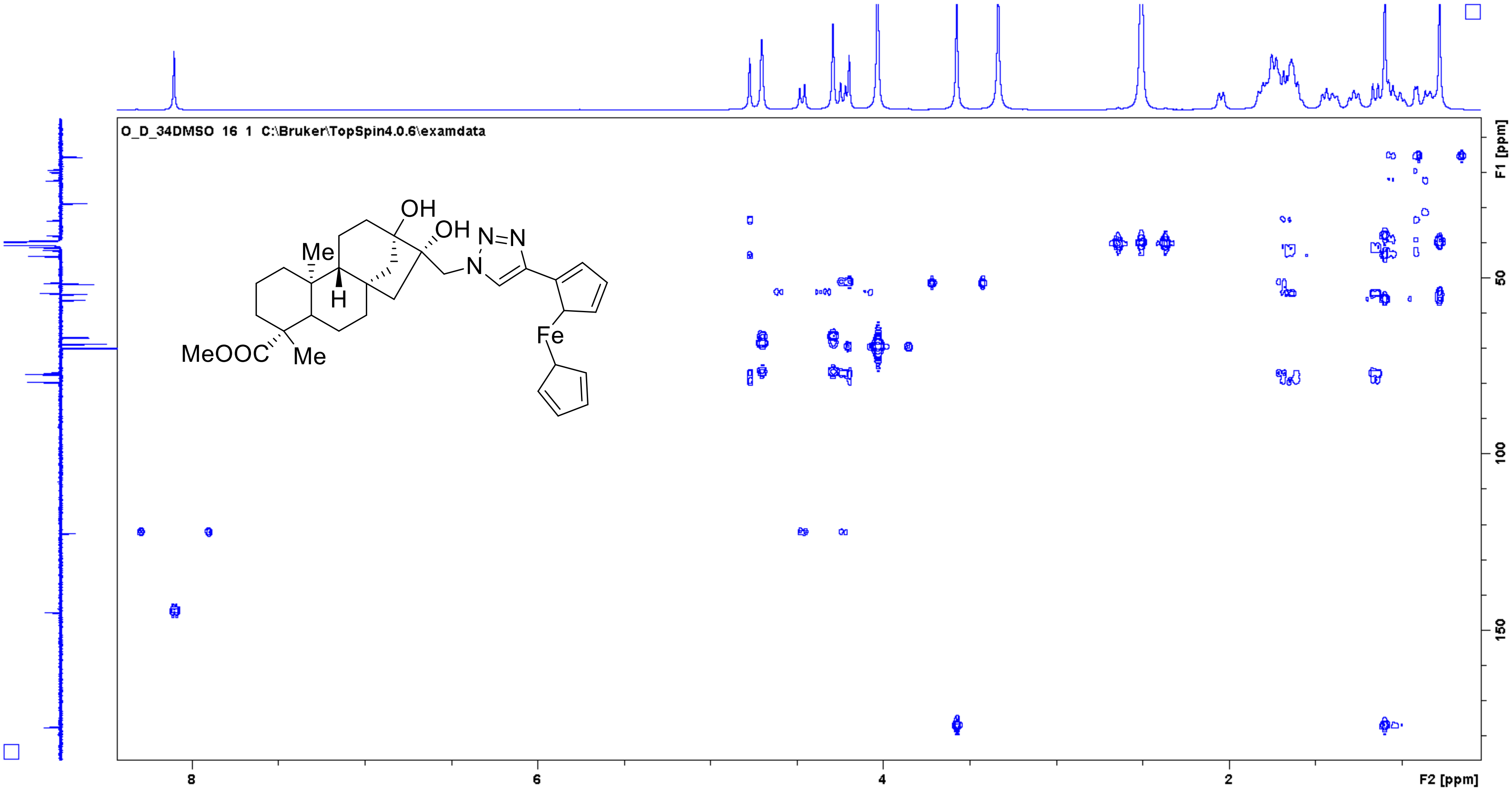

^{1}H-NMR of compound **9**

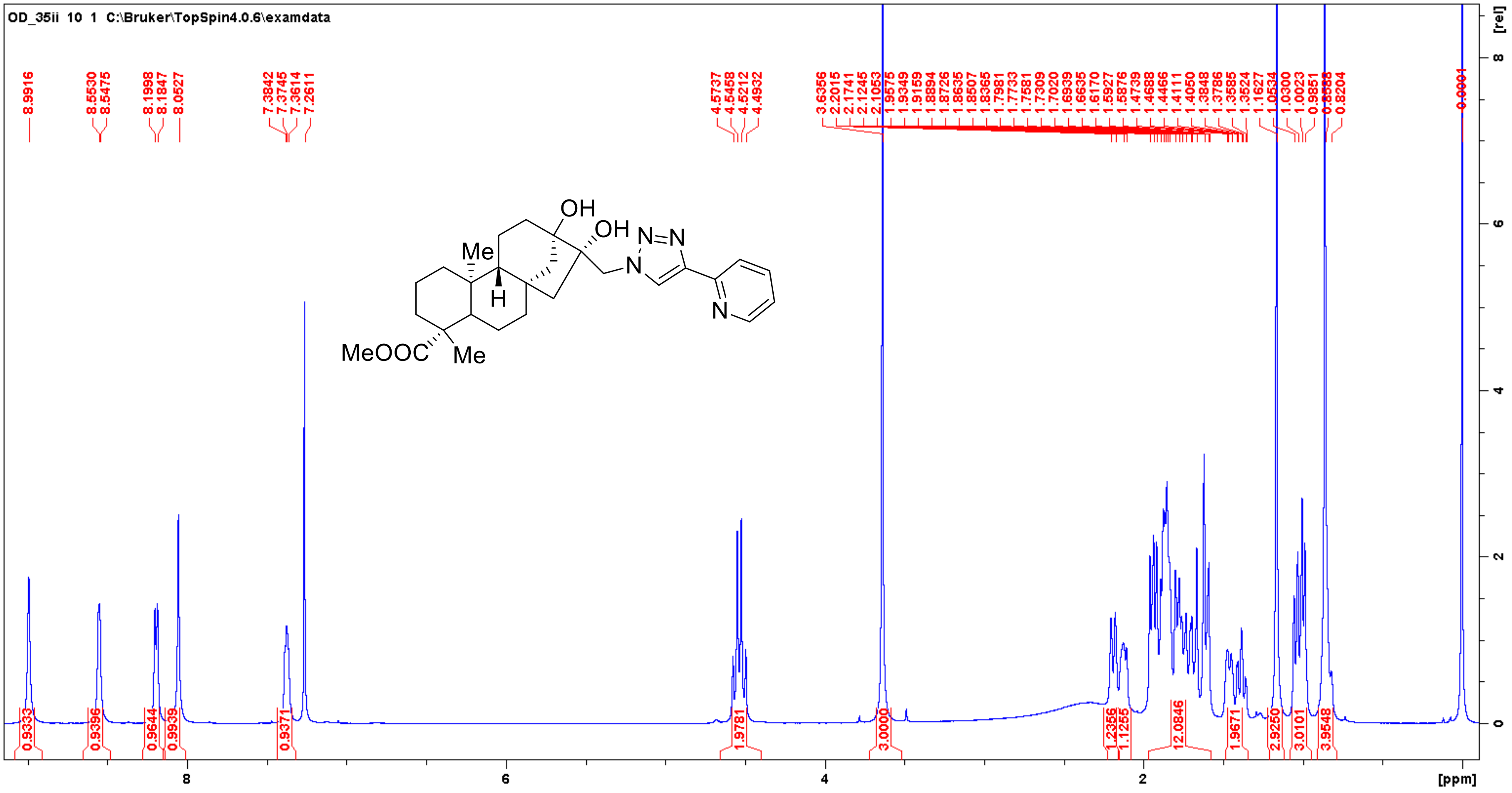

^{13}C-NMR of compound **9**

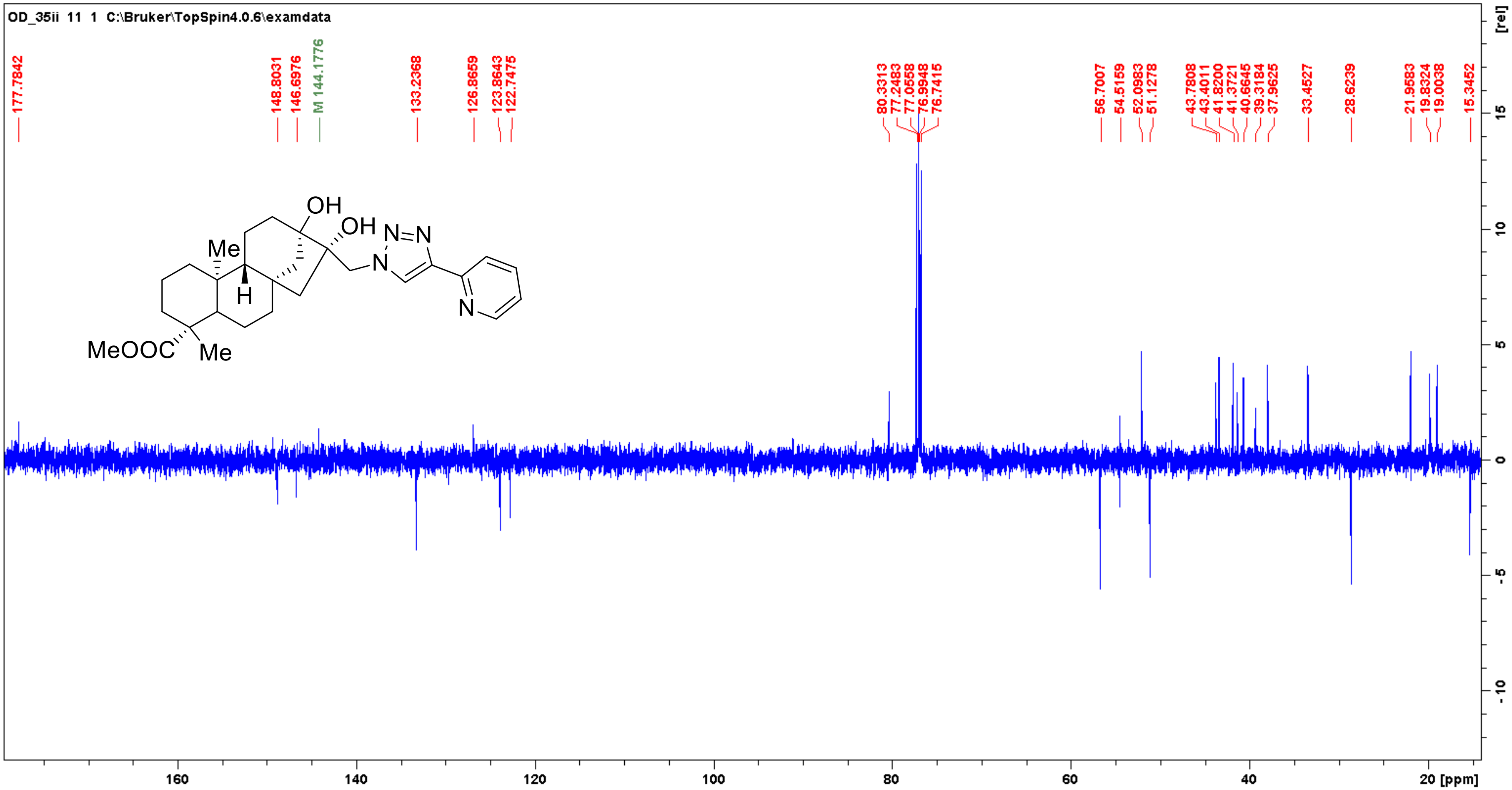

COSY of compound **9**

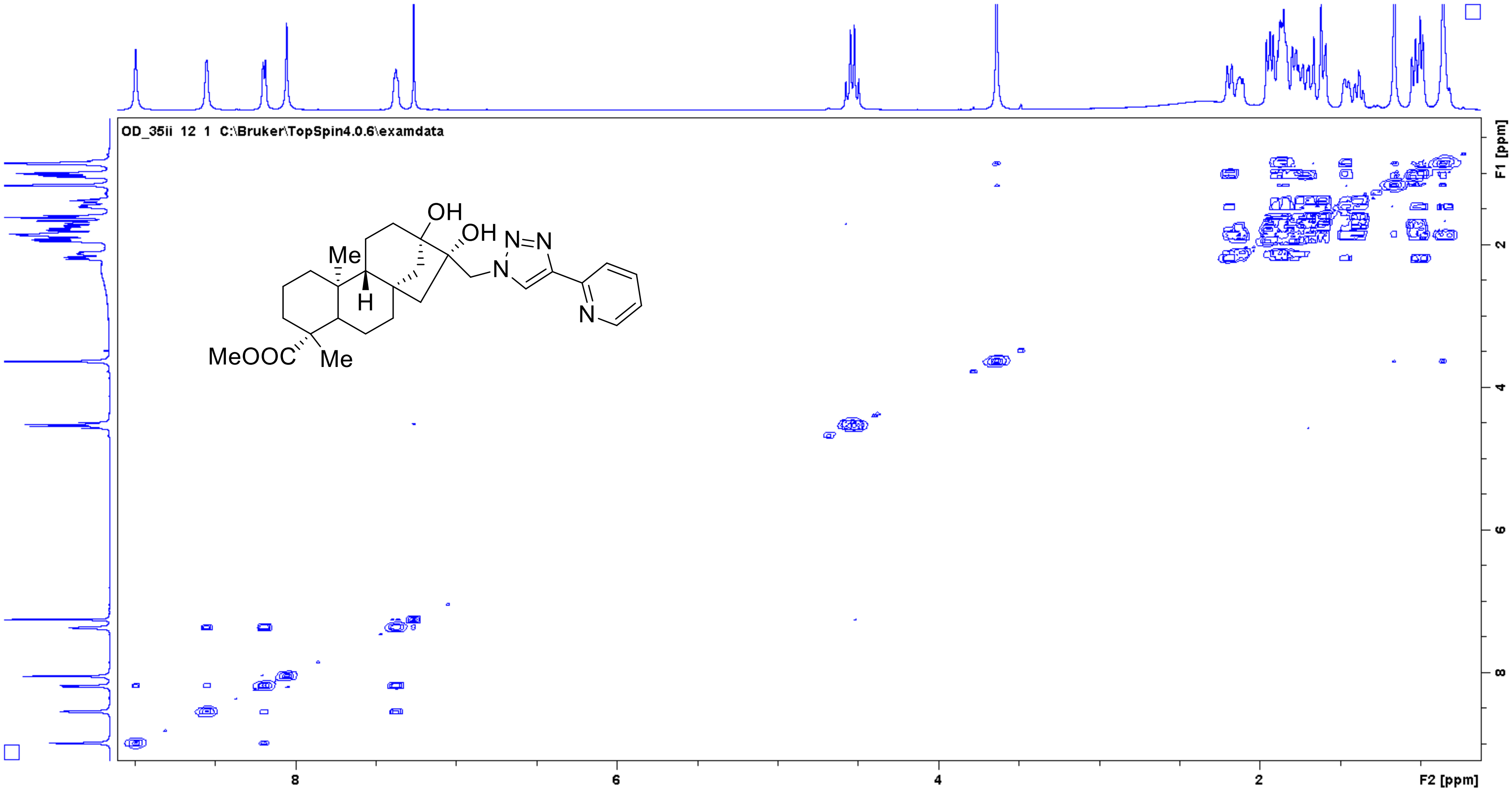

HSQC of compound **9**

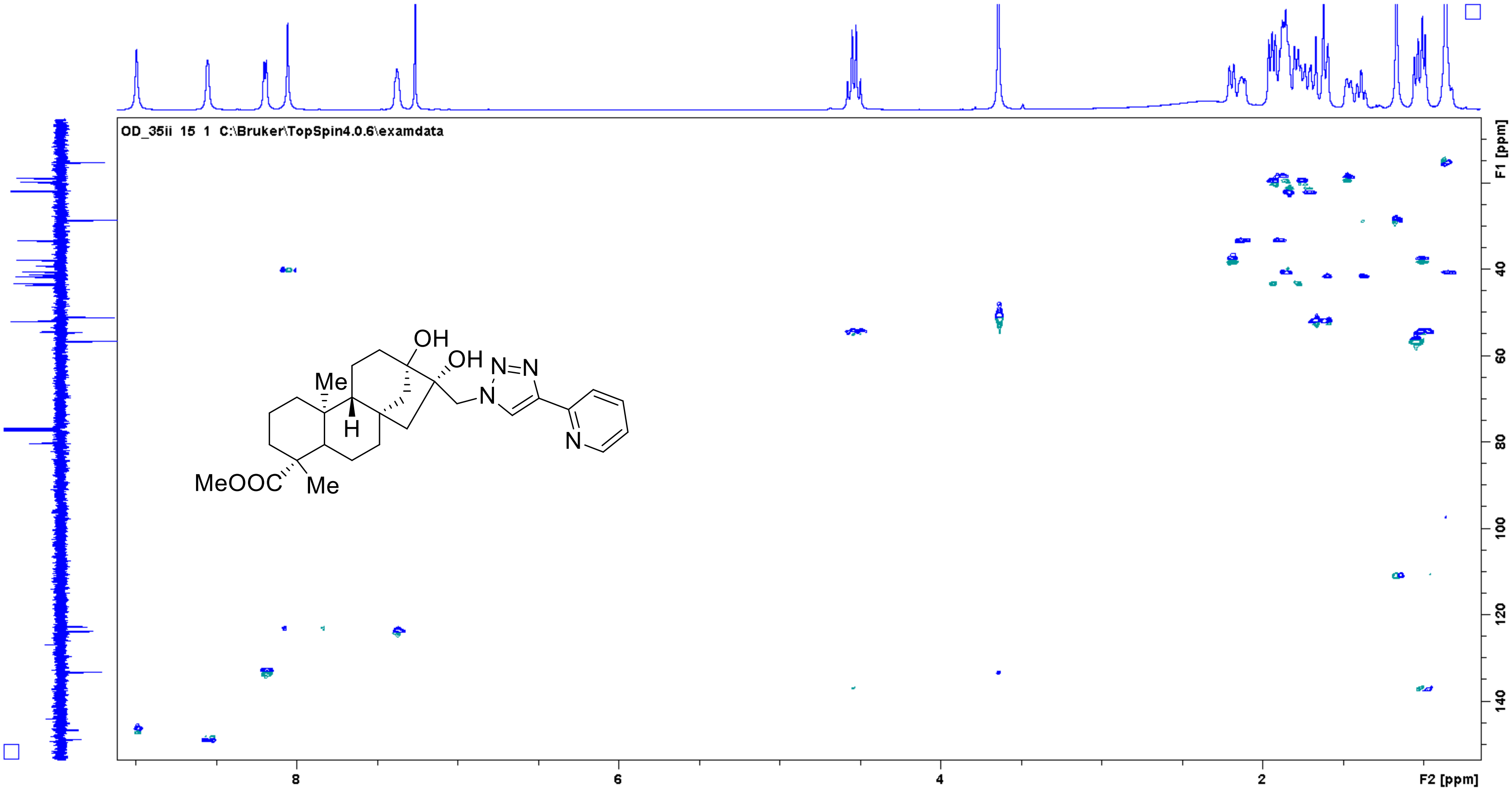

HMBC of compound **9**

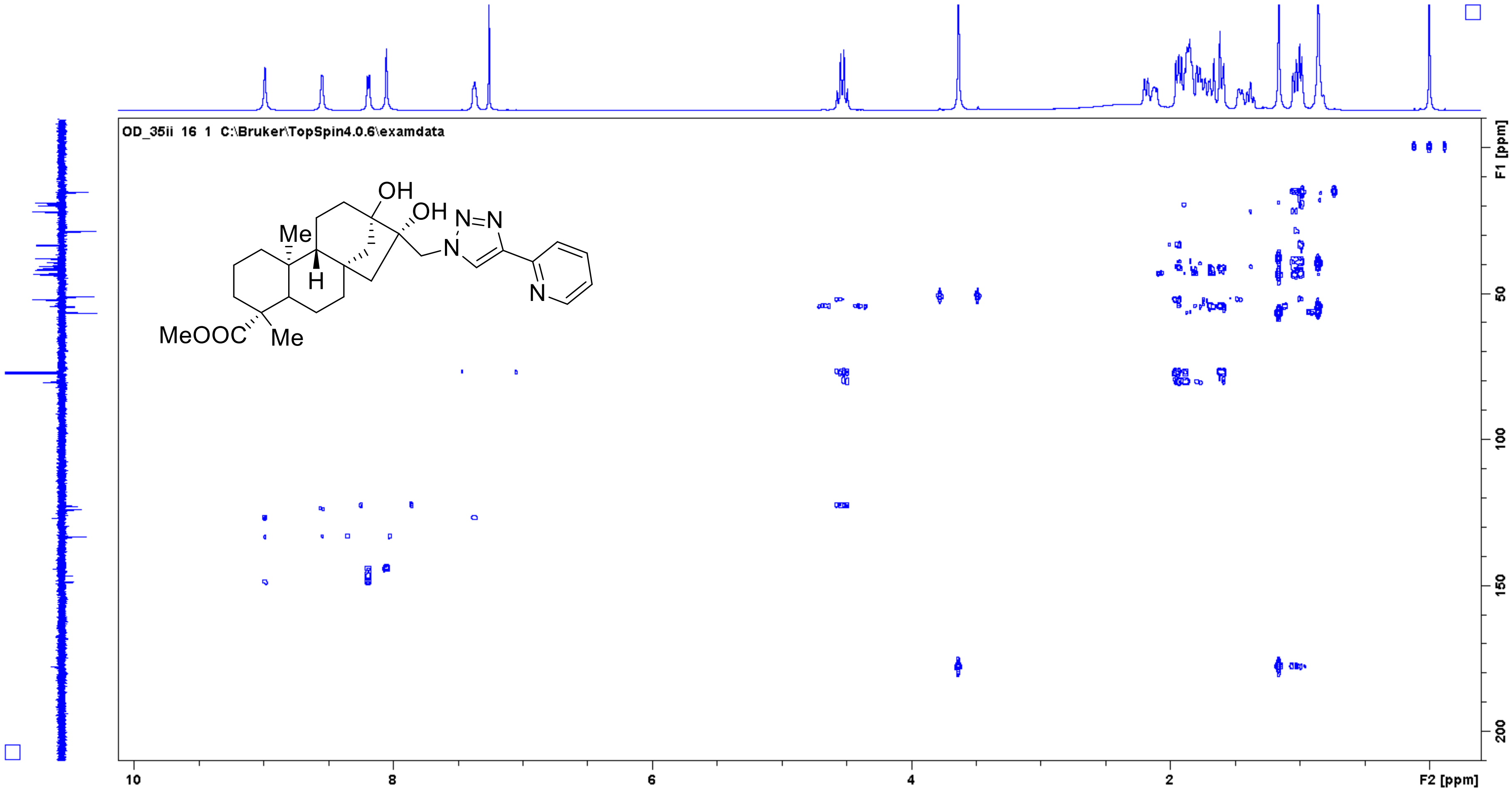
OD_35ii 16 1 C:\Bruker\TopSpin4.0.6\examdata
OH
OH
N=N
N
Me
H
N
MeOOC
Me
F1 [ppm]
50
100
150
200
10
8
6
4
2
F2 [ppm]

^{1}H-NMR of compound **10**

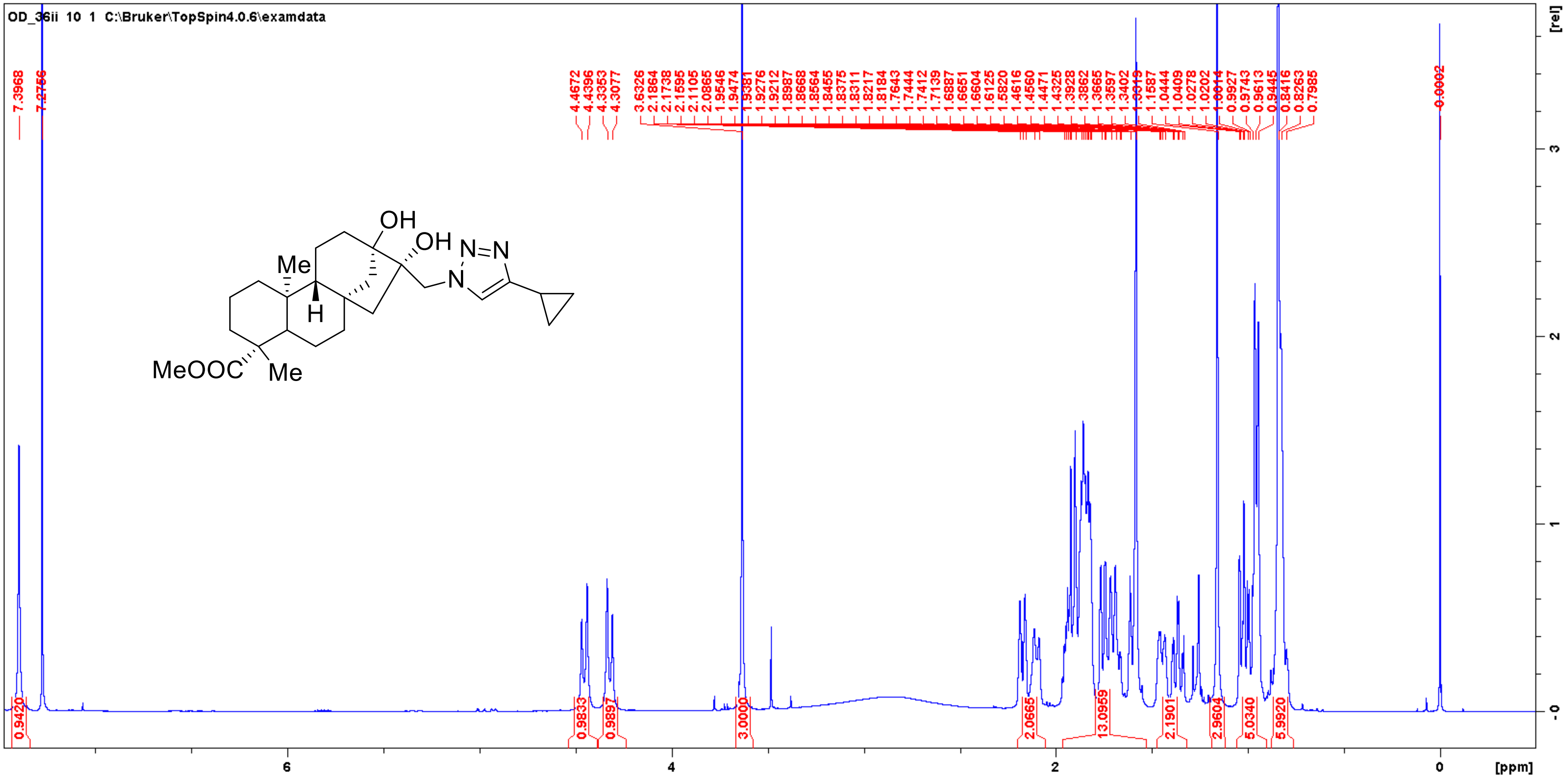

^{13}C-NMR of compound **10**

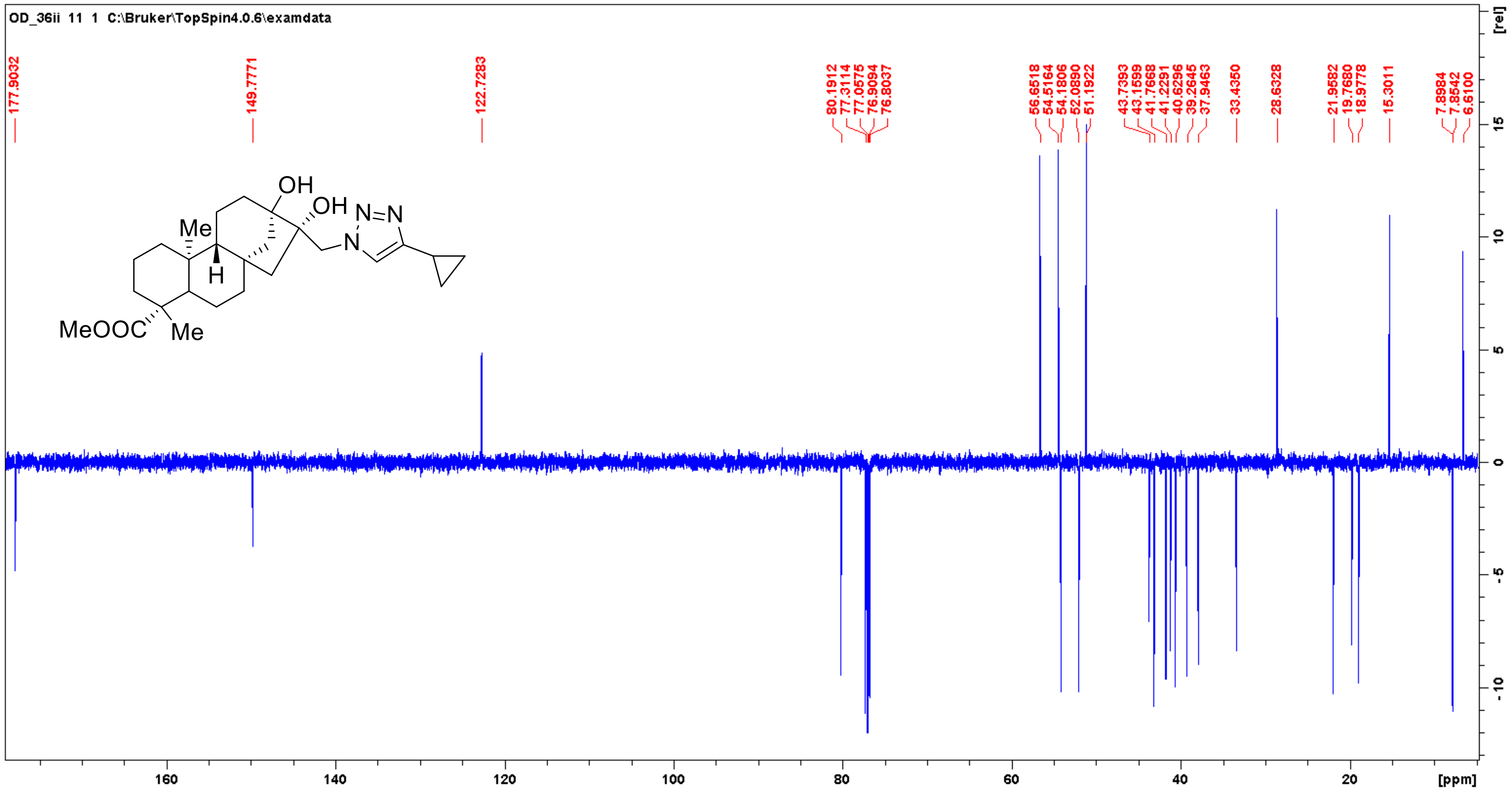

COSY of compound **10**

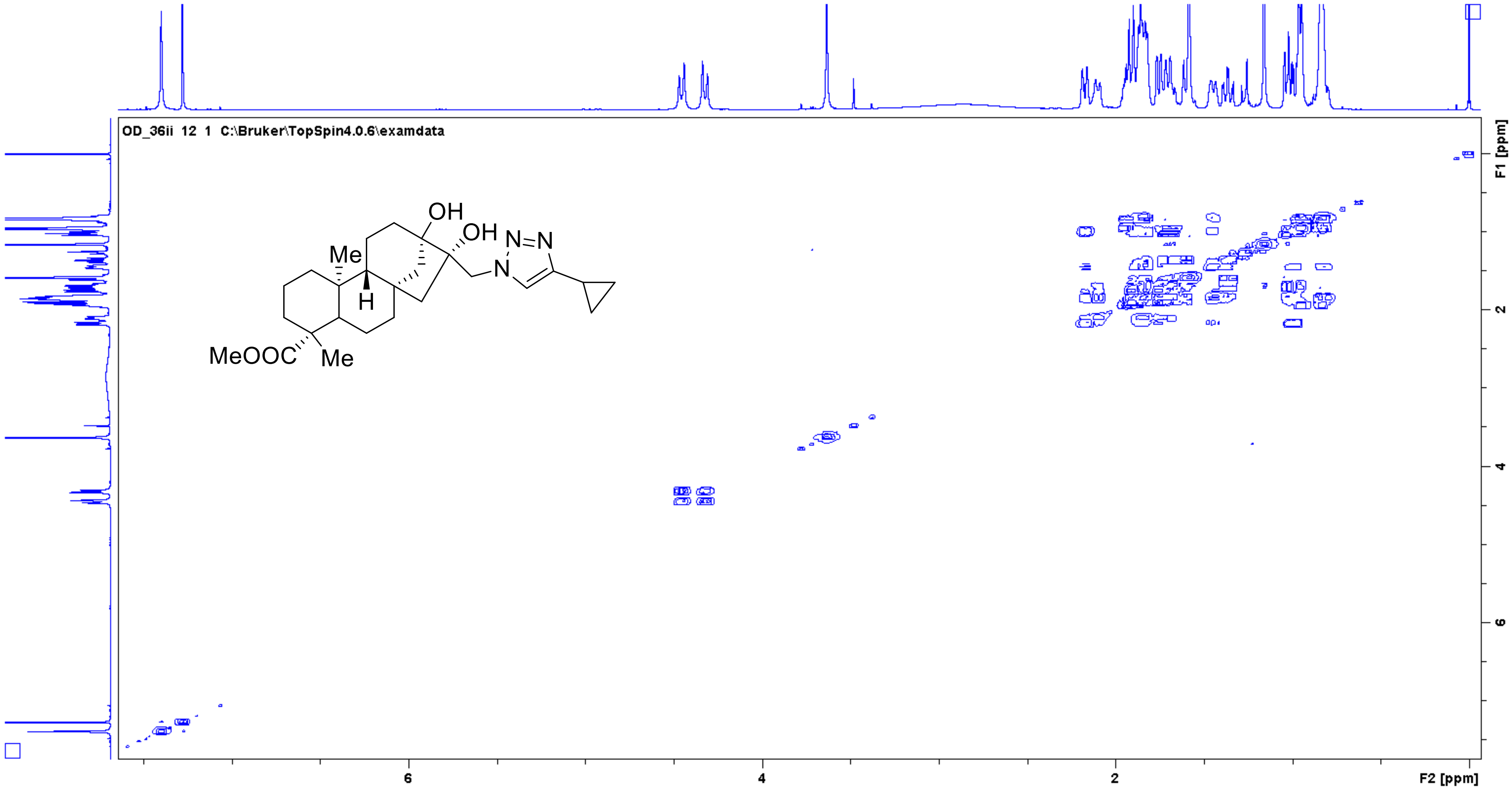

HSQC of compound **10**

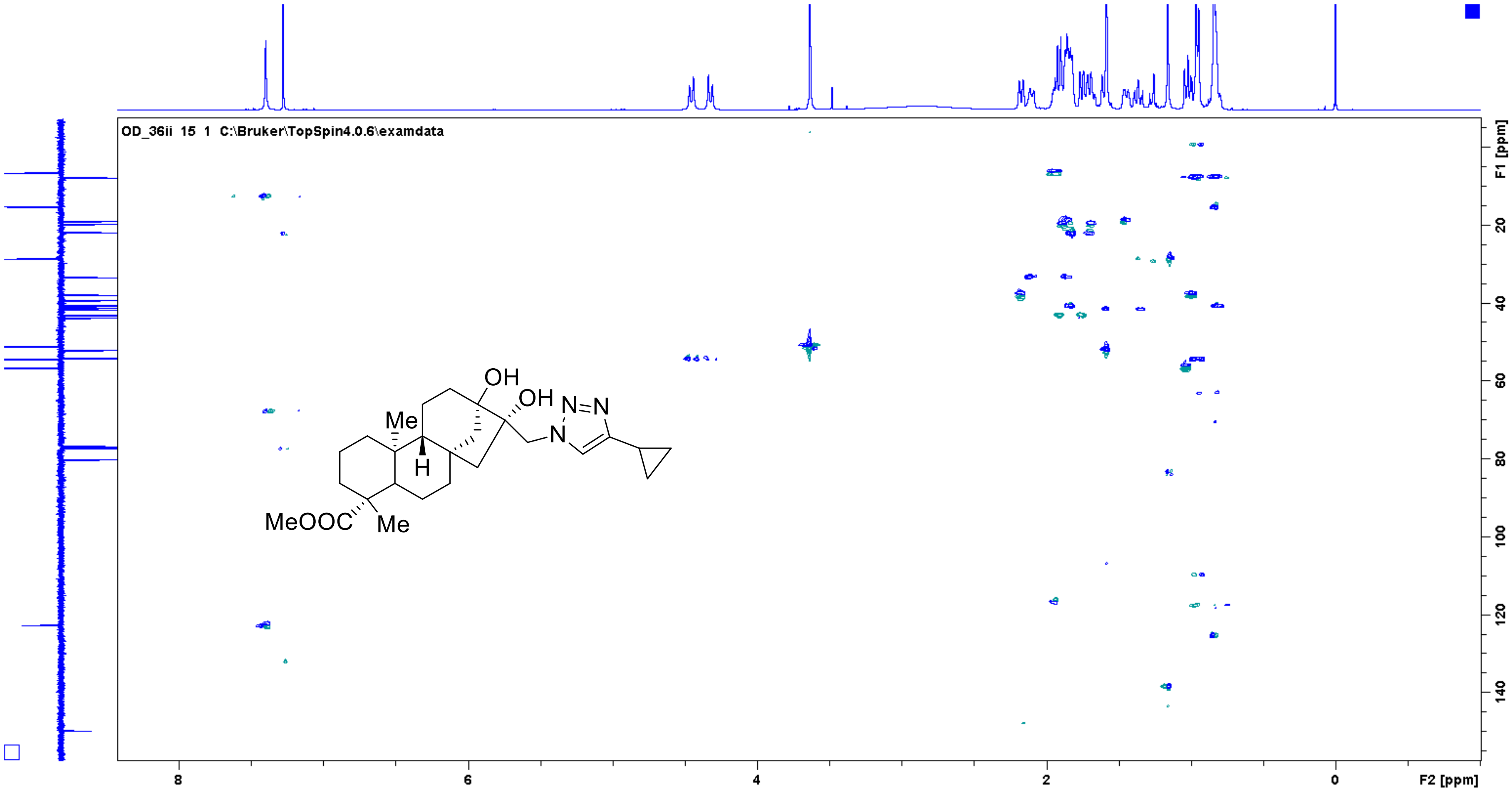

HMBC of compound **10**

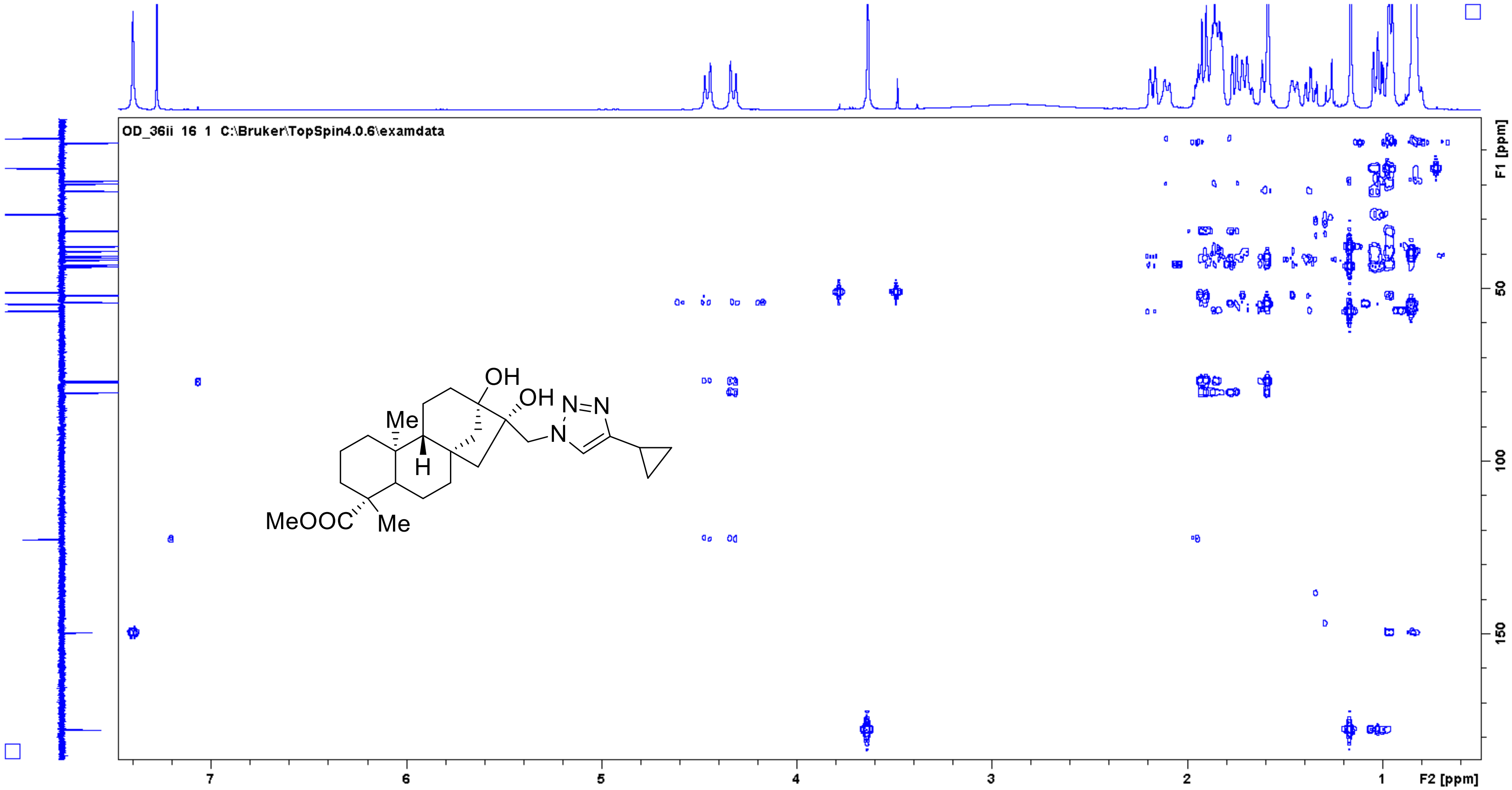

^{1}H-NMR of compound **11**

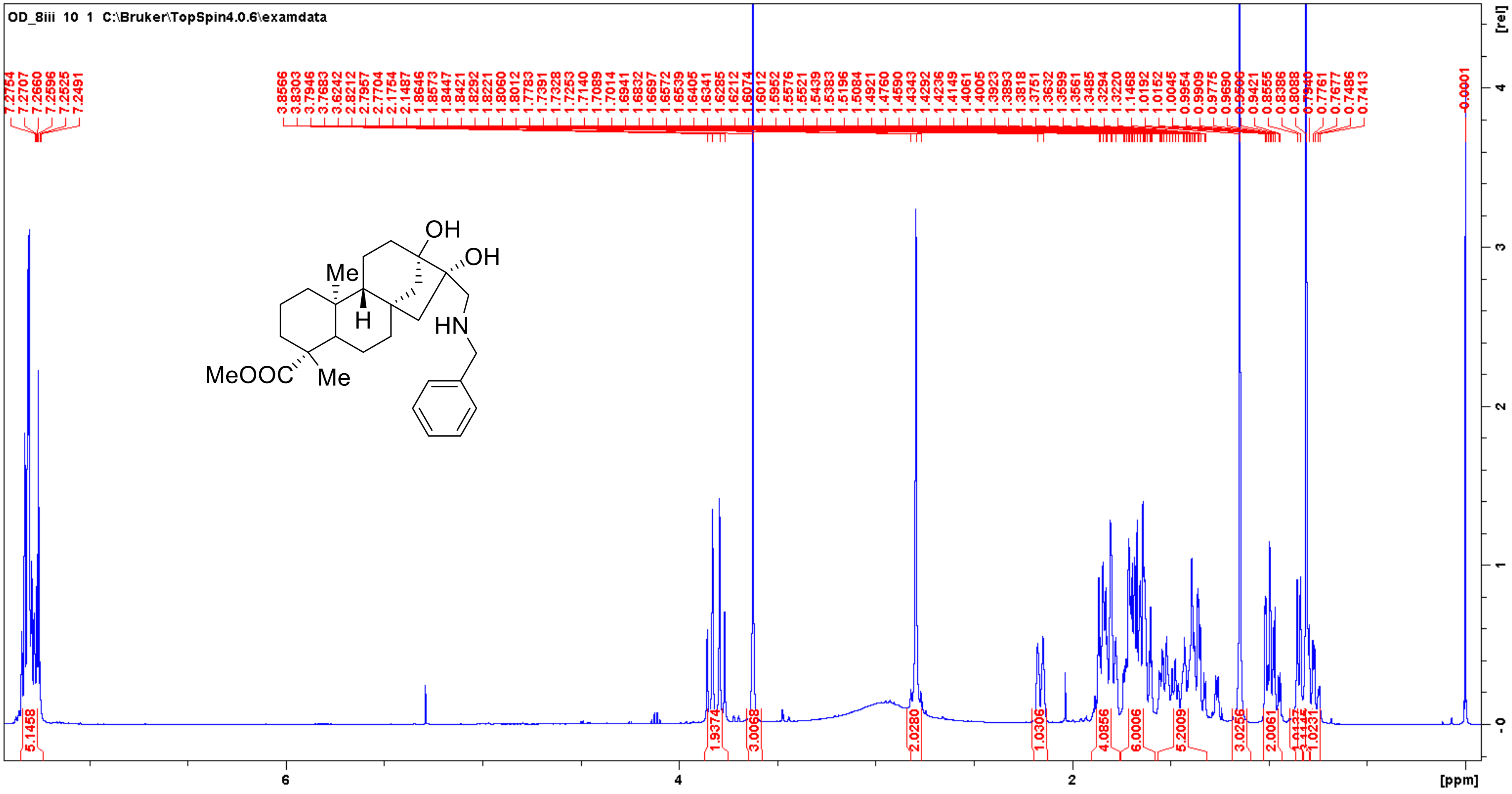

^{13}C-NMR of compound **11**

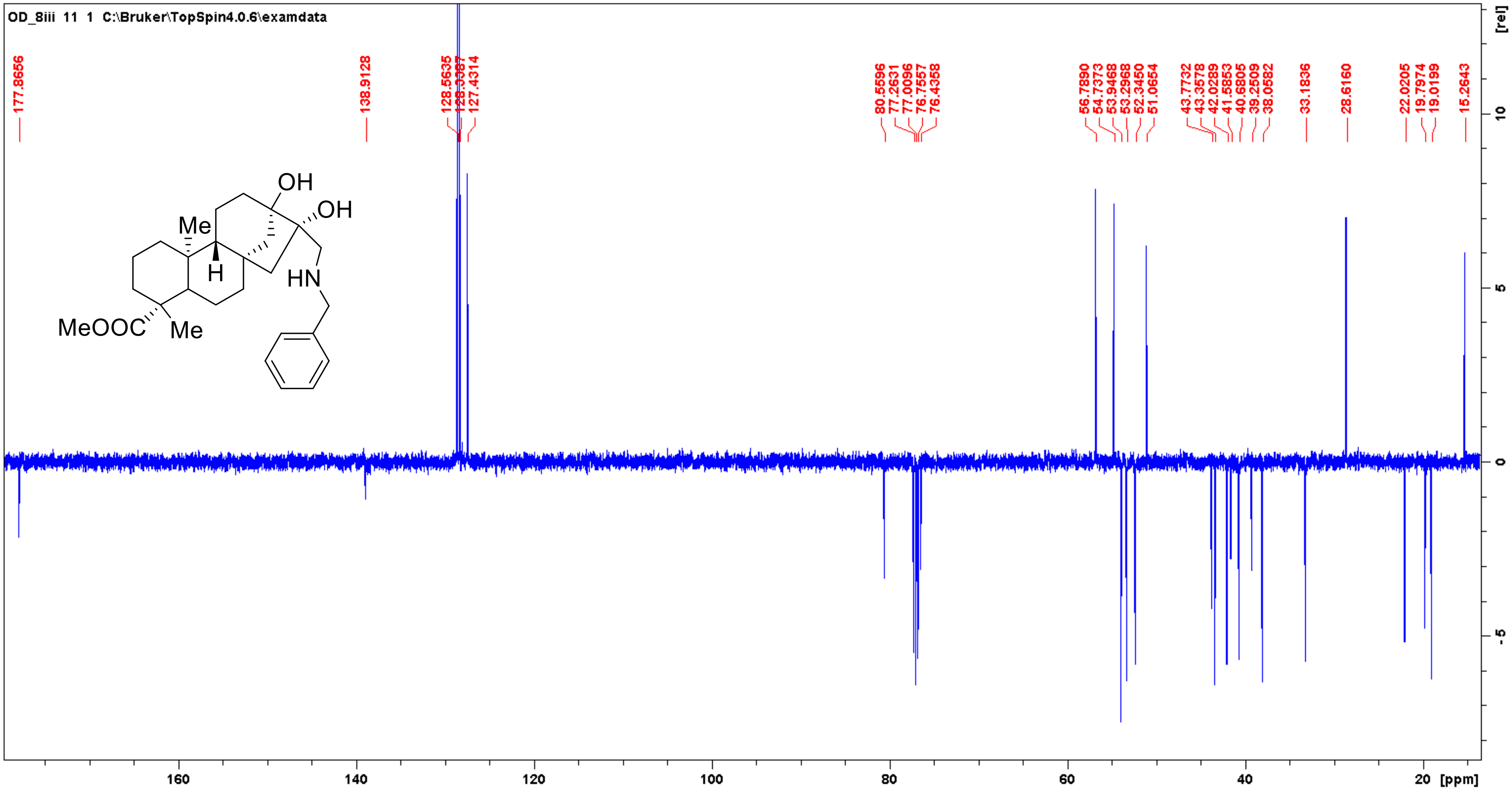

COSY of compound **11**

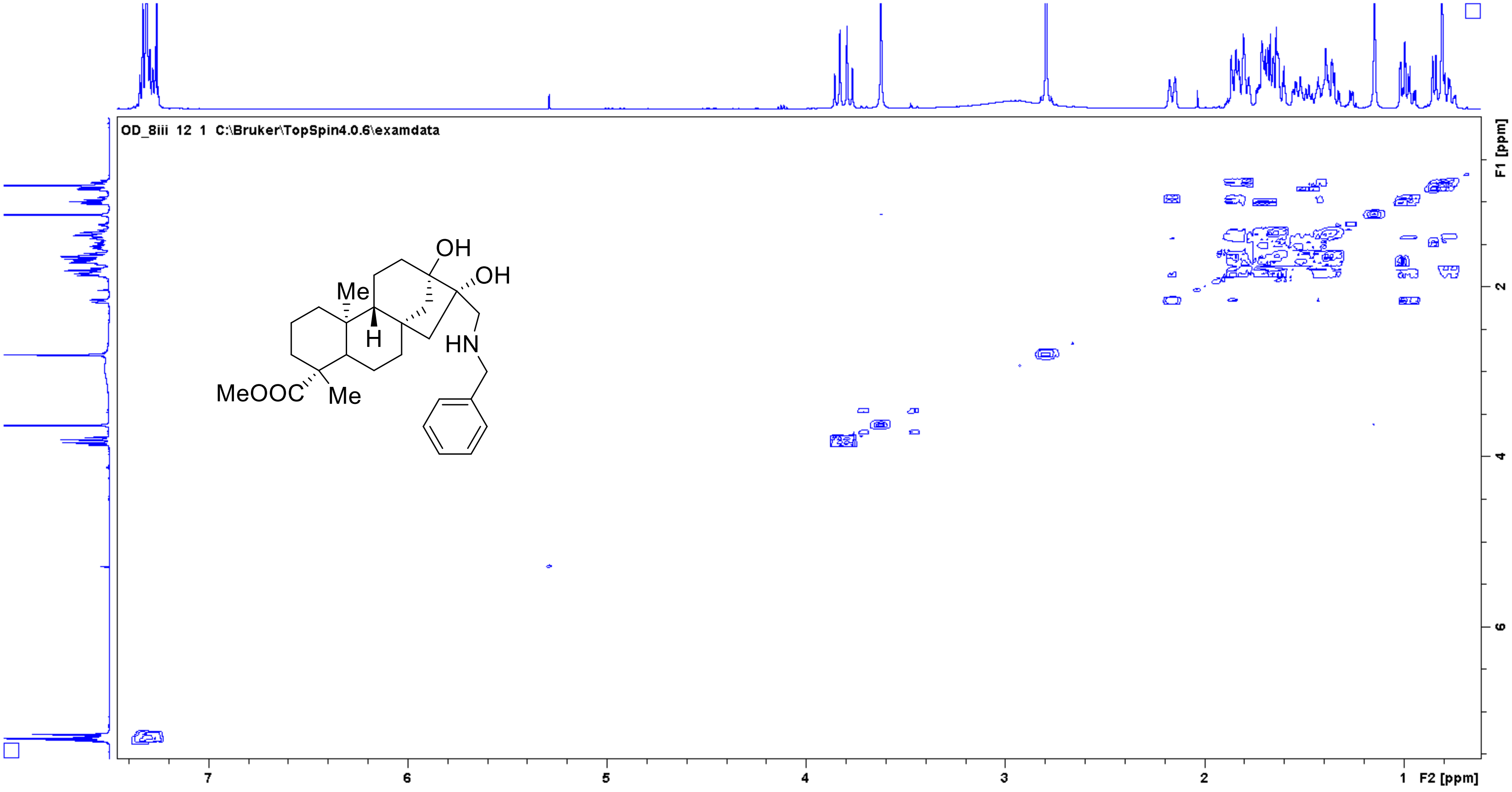

NOESY of compound **11**

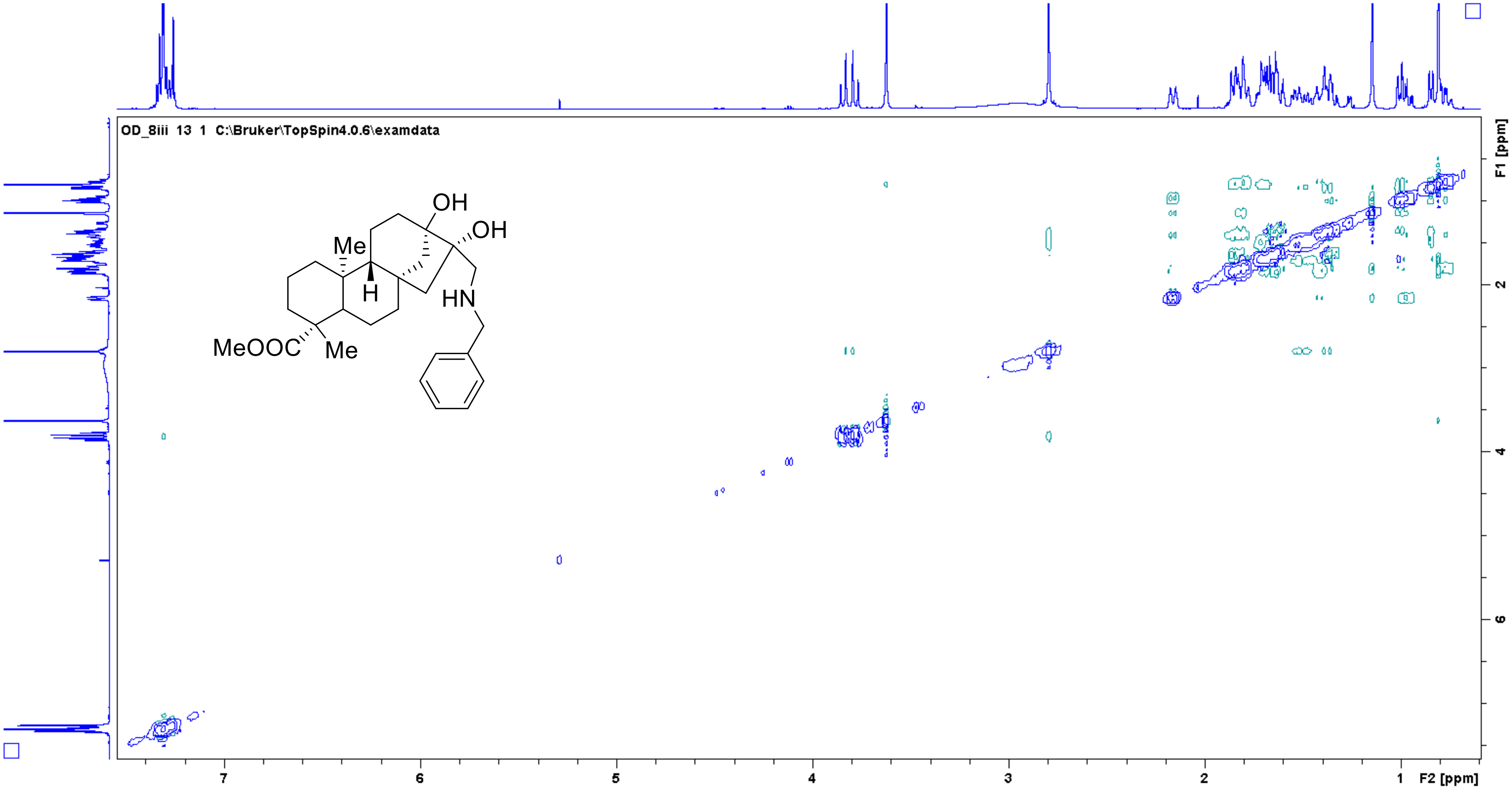

HSQC of compound **11**

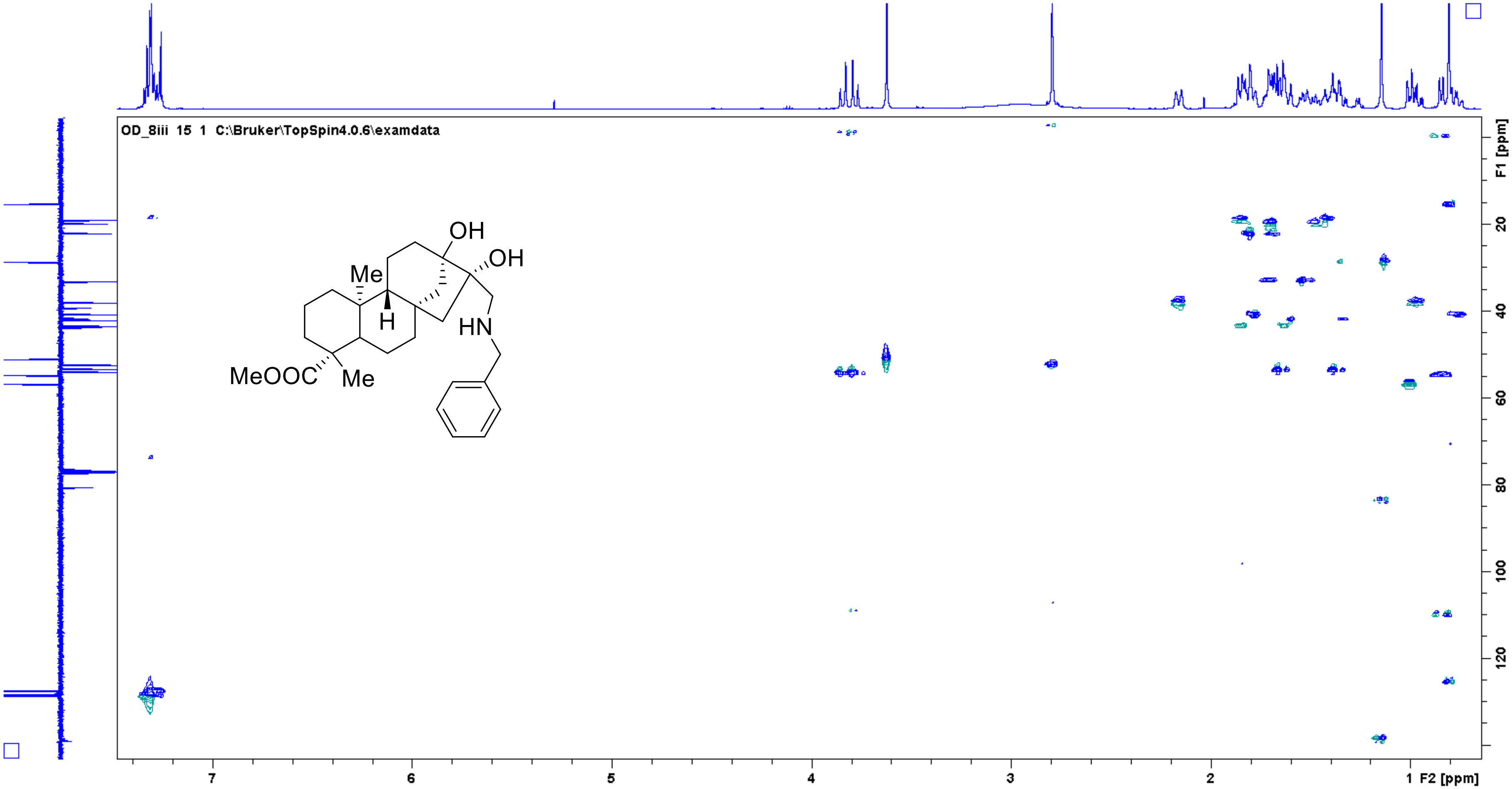

HMBC of compound **11**

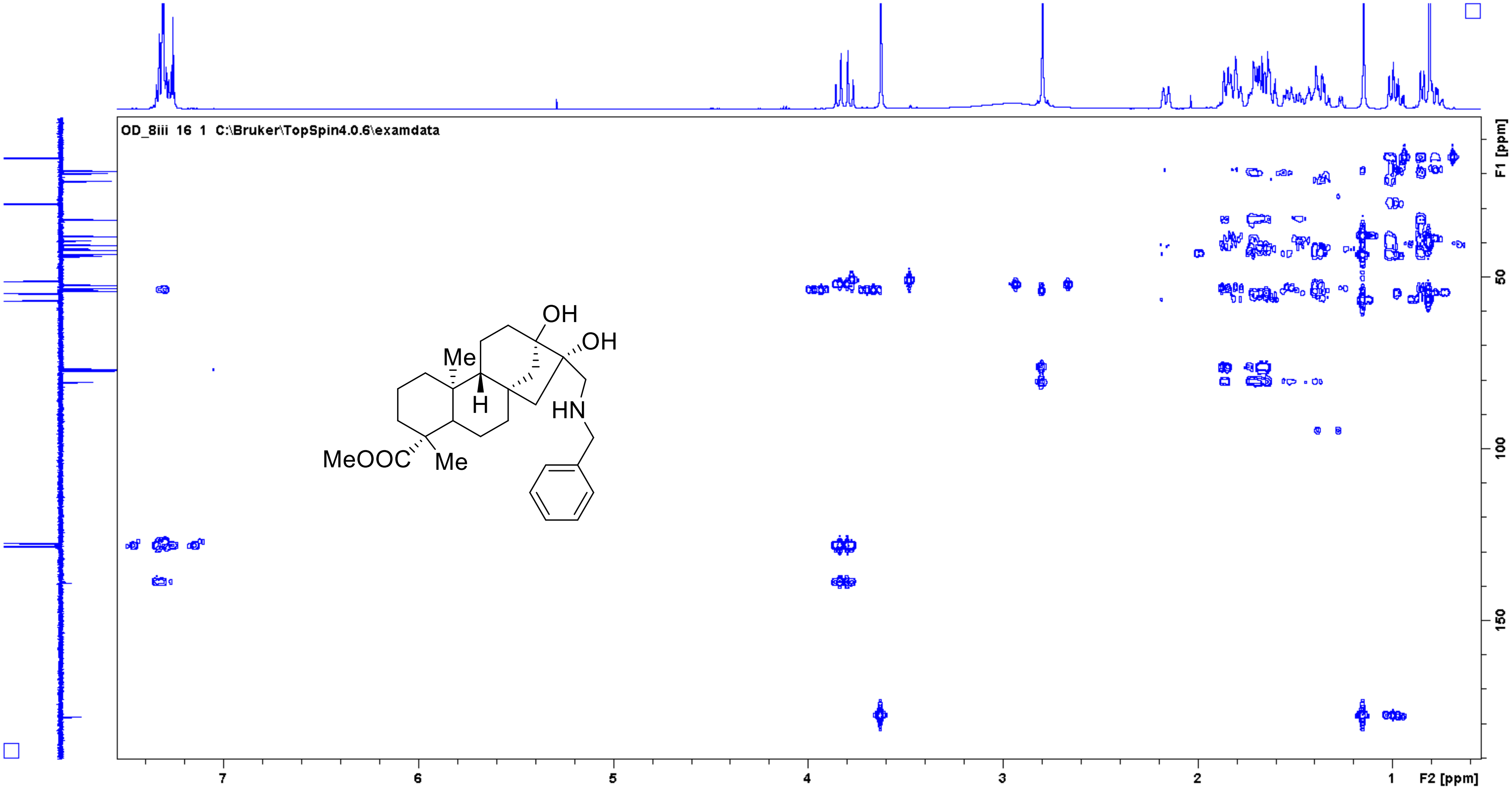

^{1}H-NMR of compound **12**

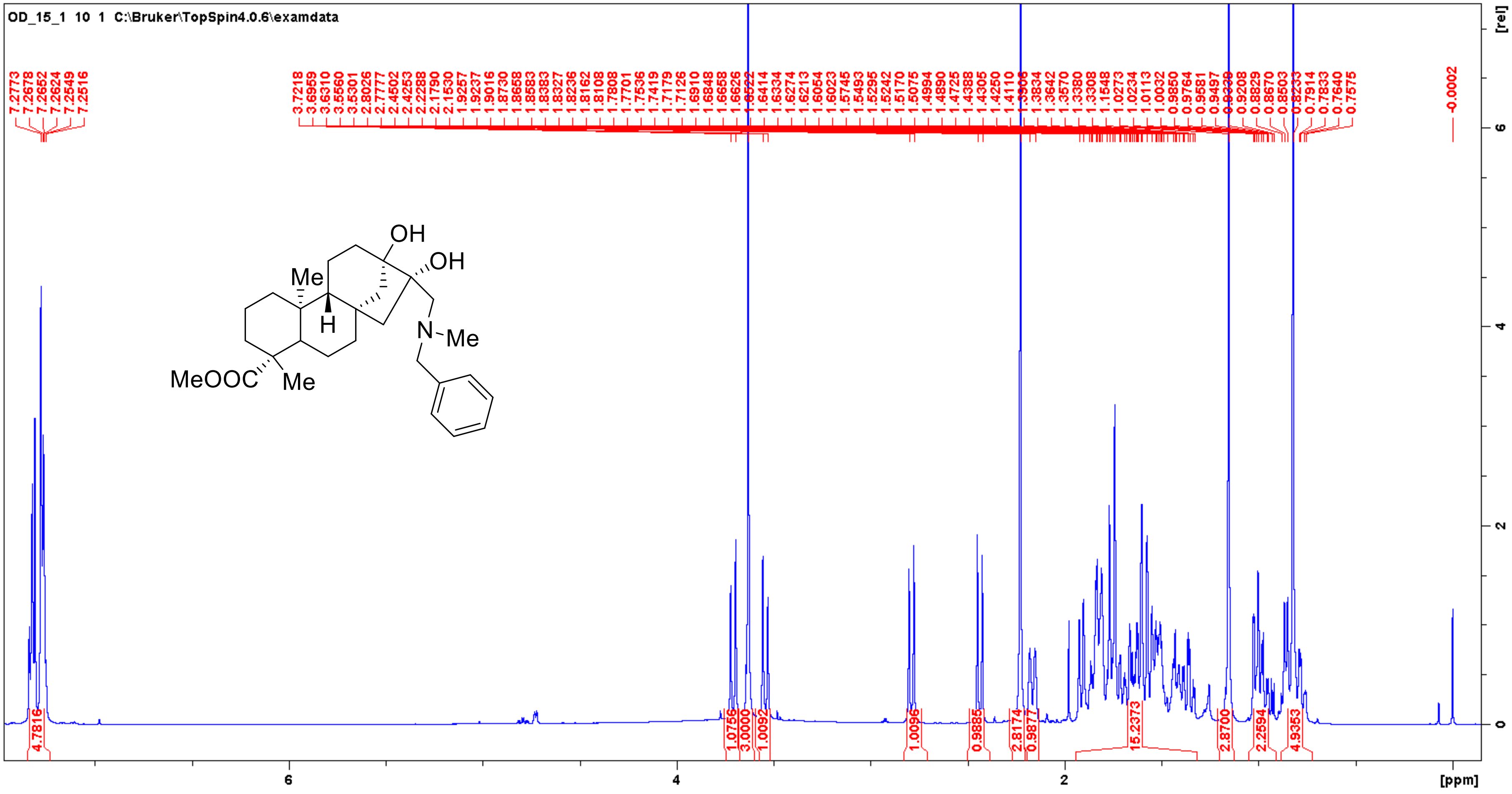

^{13}C-NMR of compound **12**

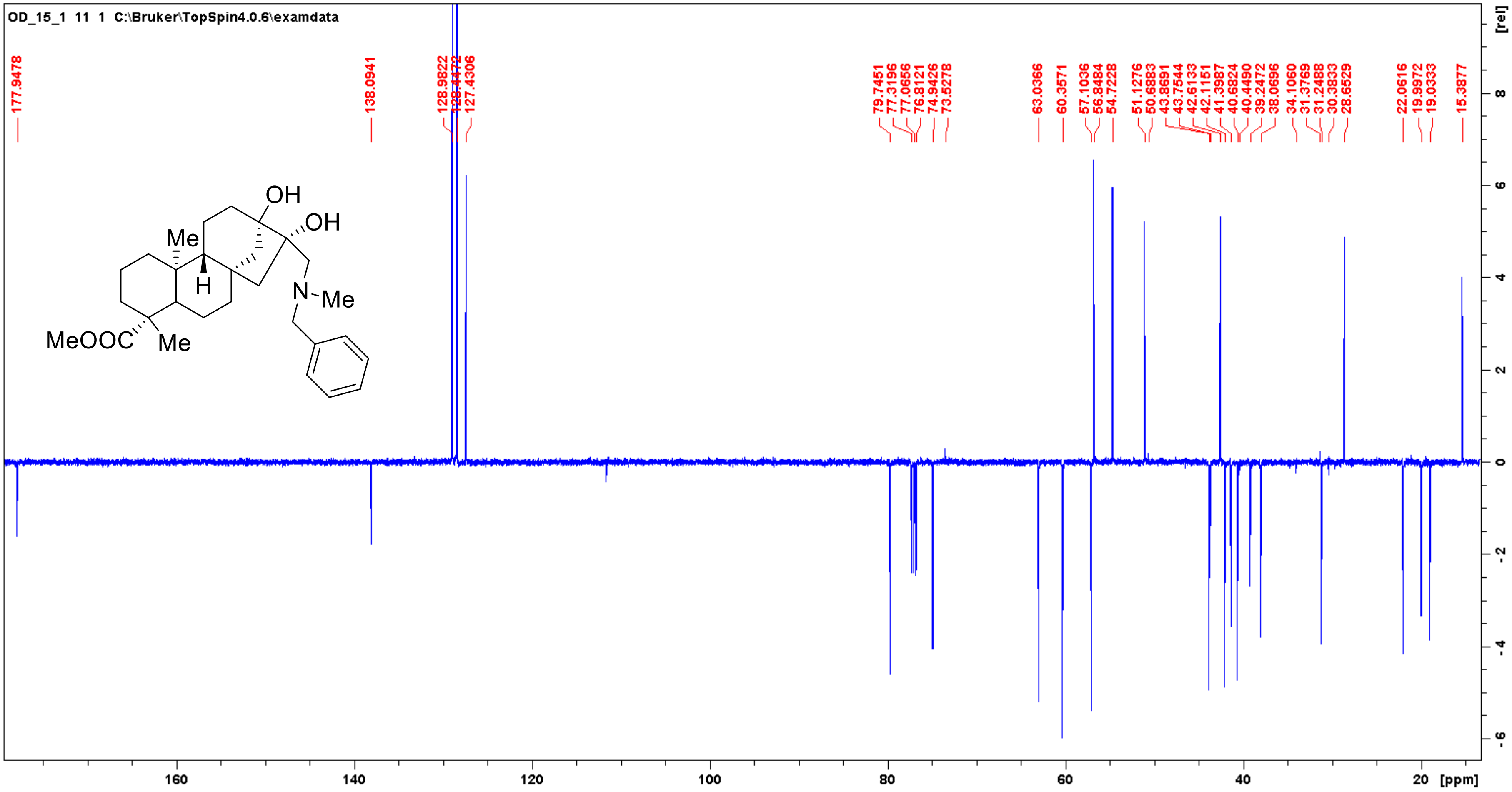

COSY of compound **12**

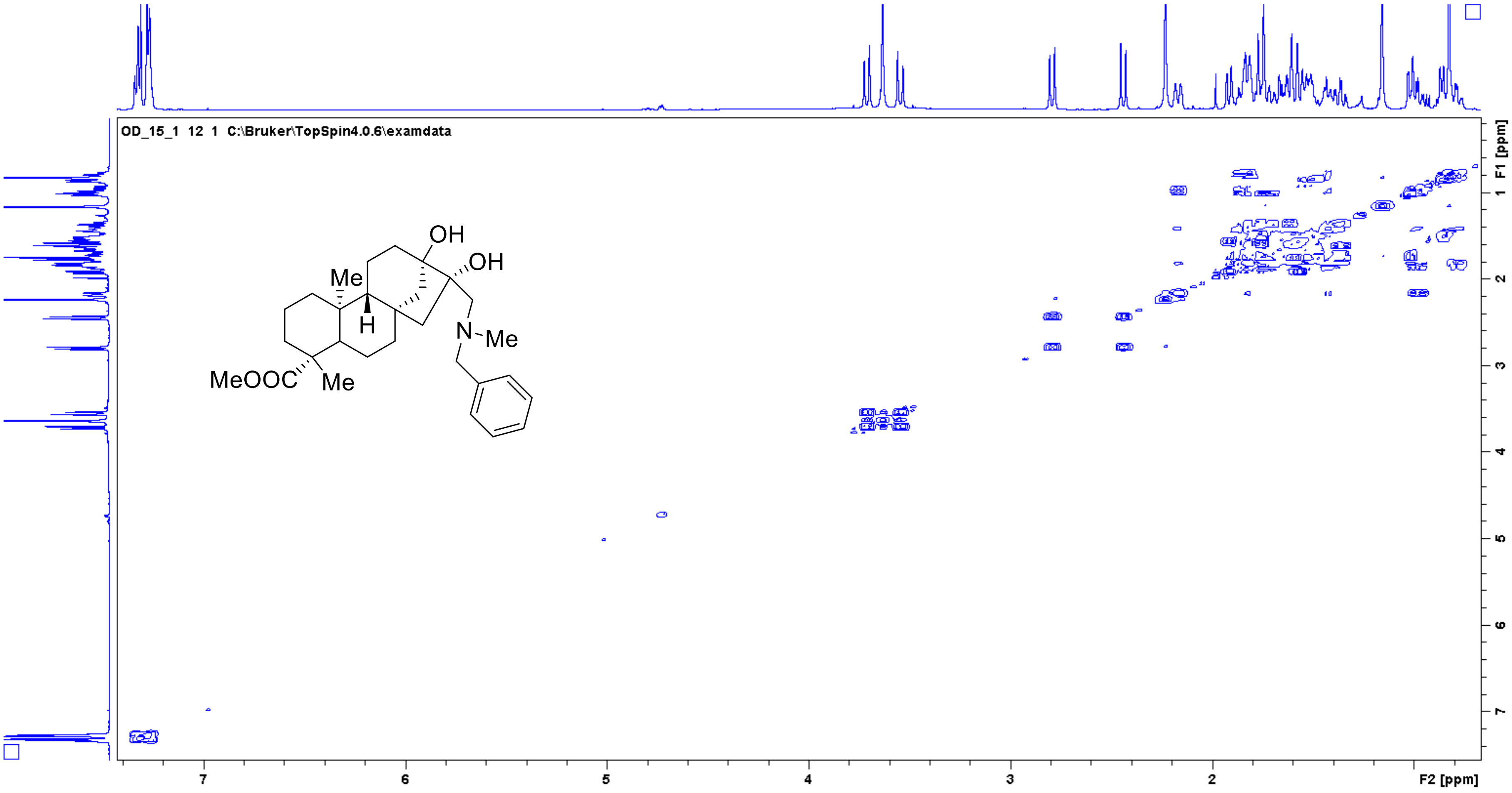

NOESY of compound **12**

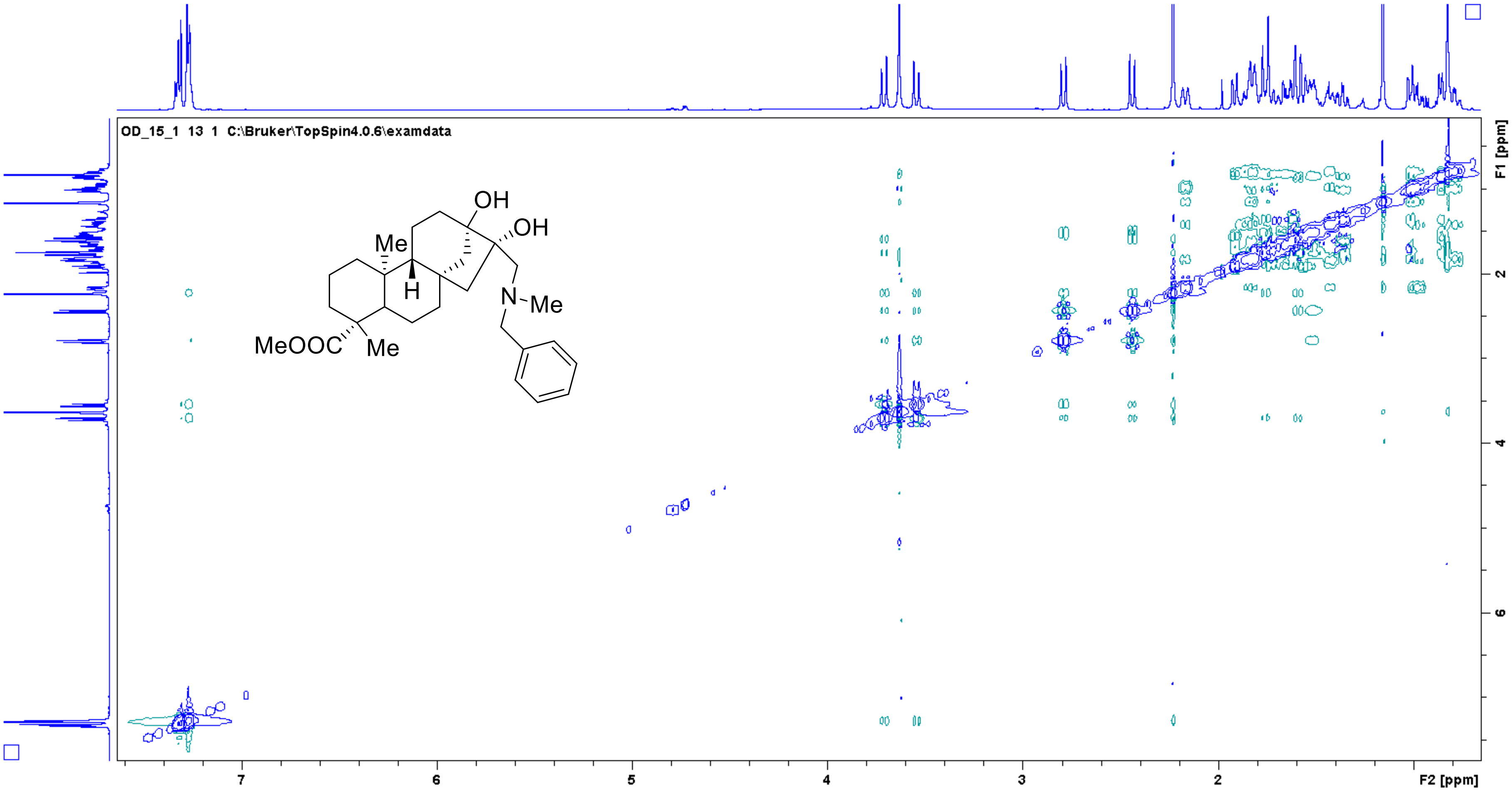

HSQC of compound **12**

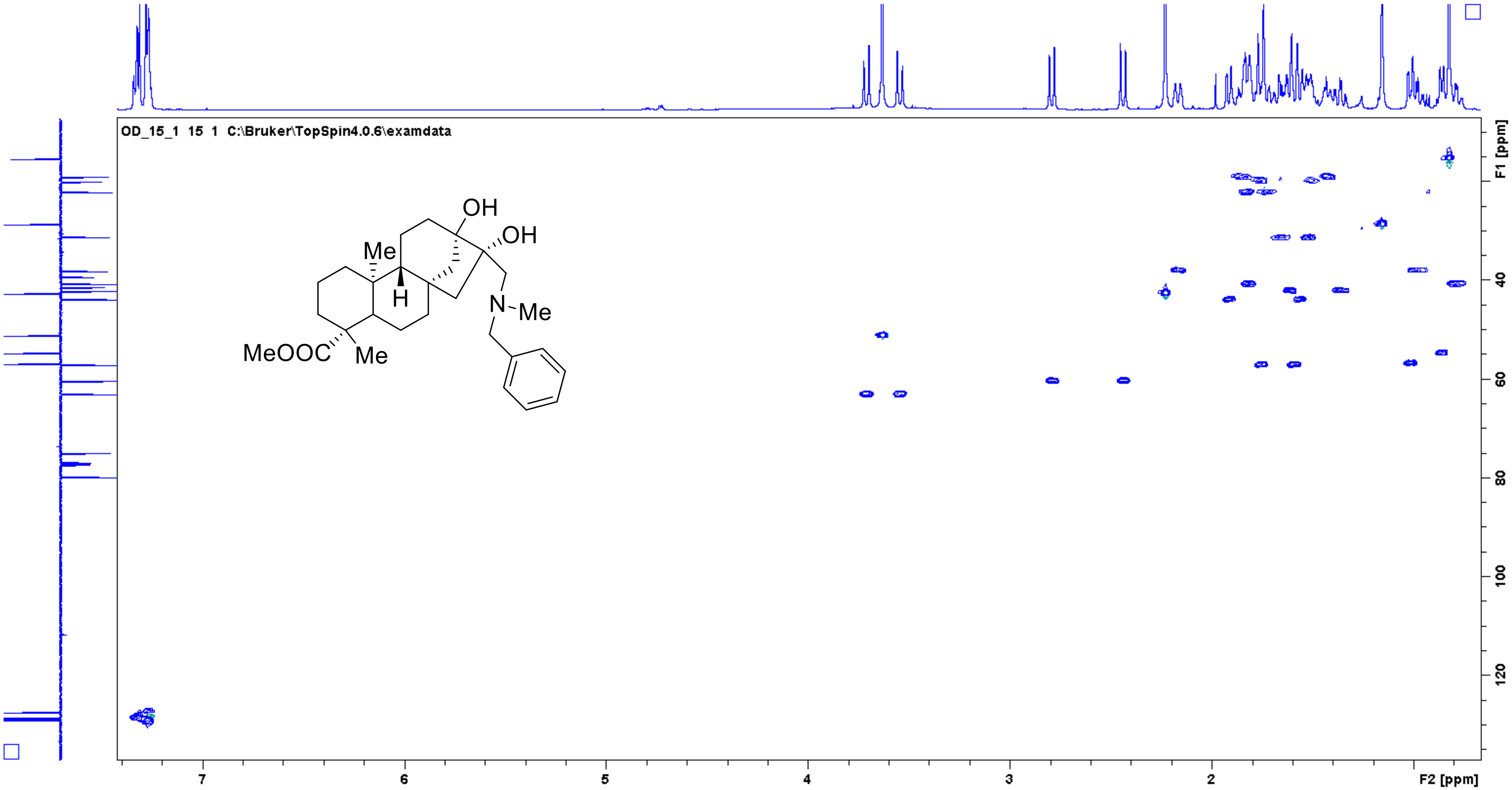

HMBC of compound **12**

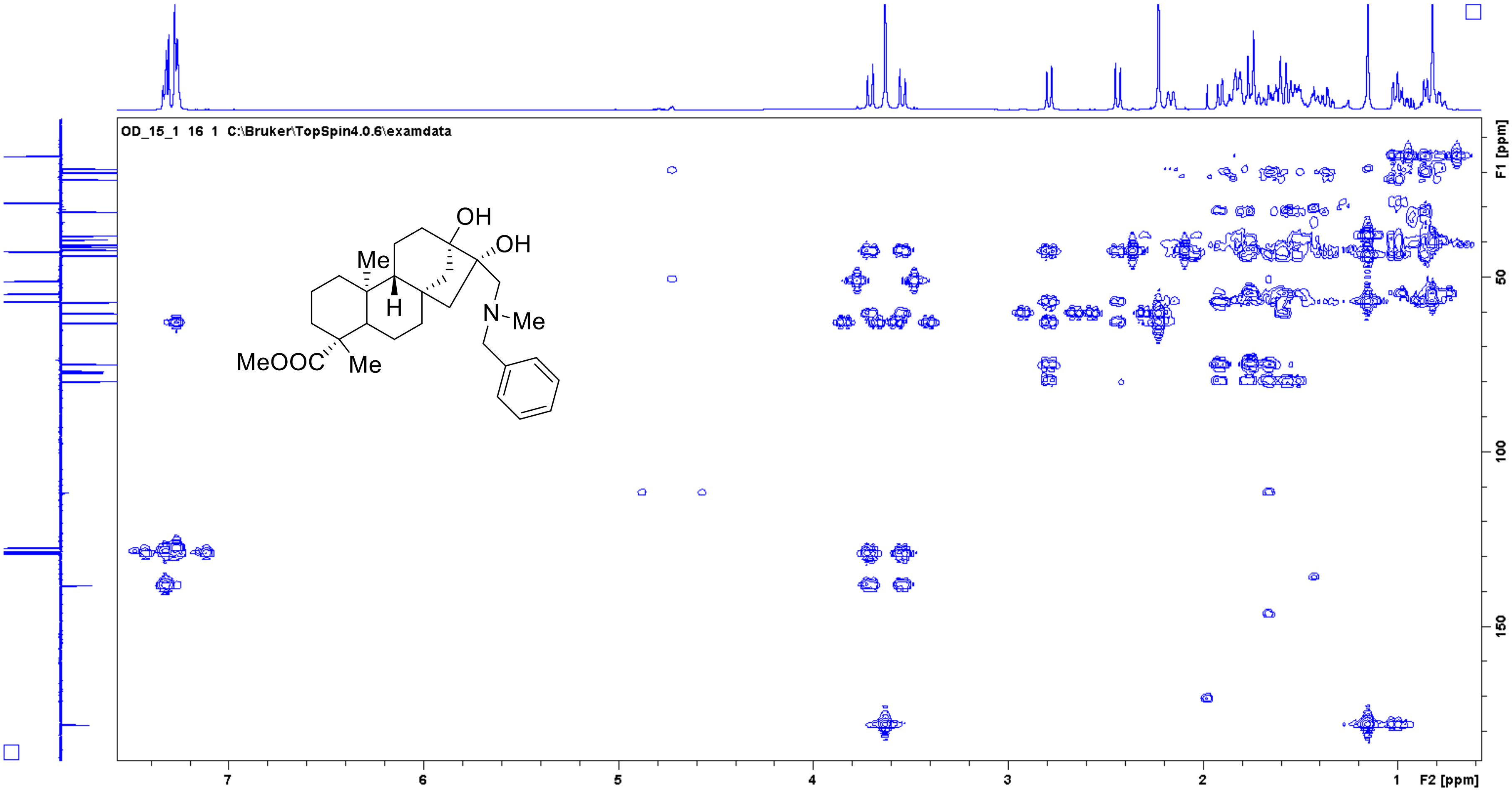

^{1}H-NMR of compound **13**

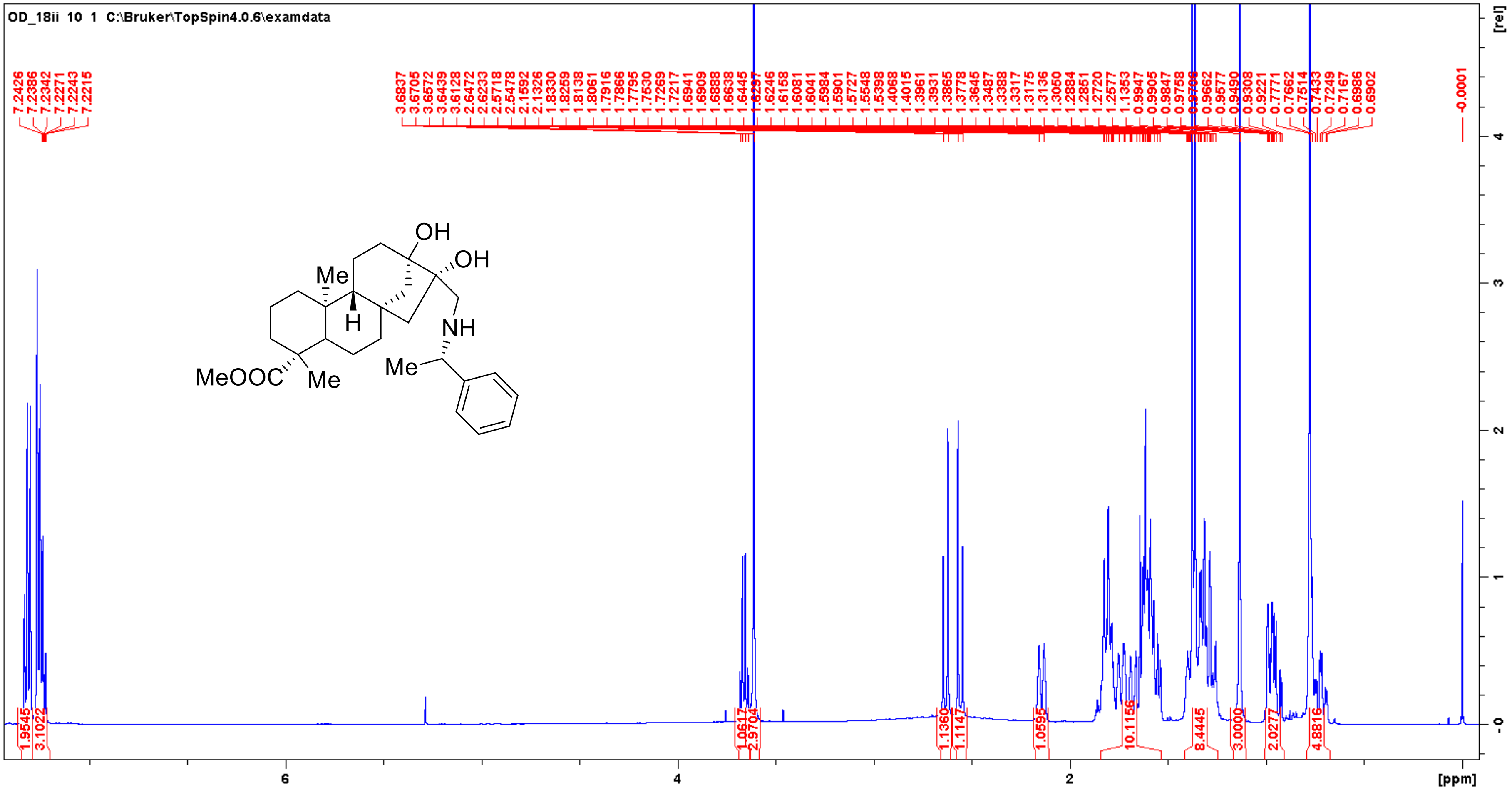

^{13}C-NMR of compound **13**

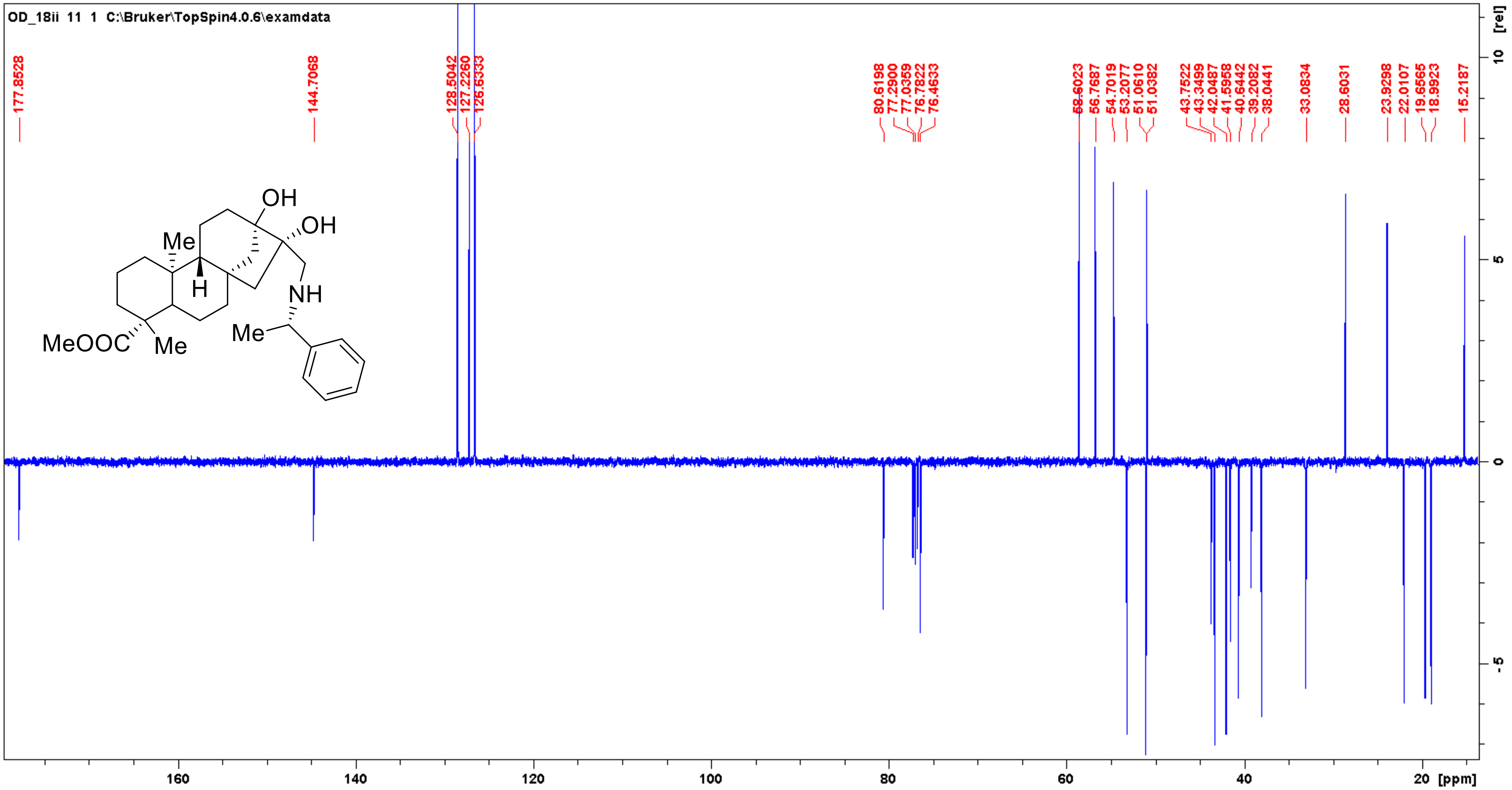

COSY of compound **13**

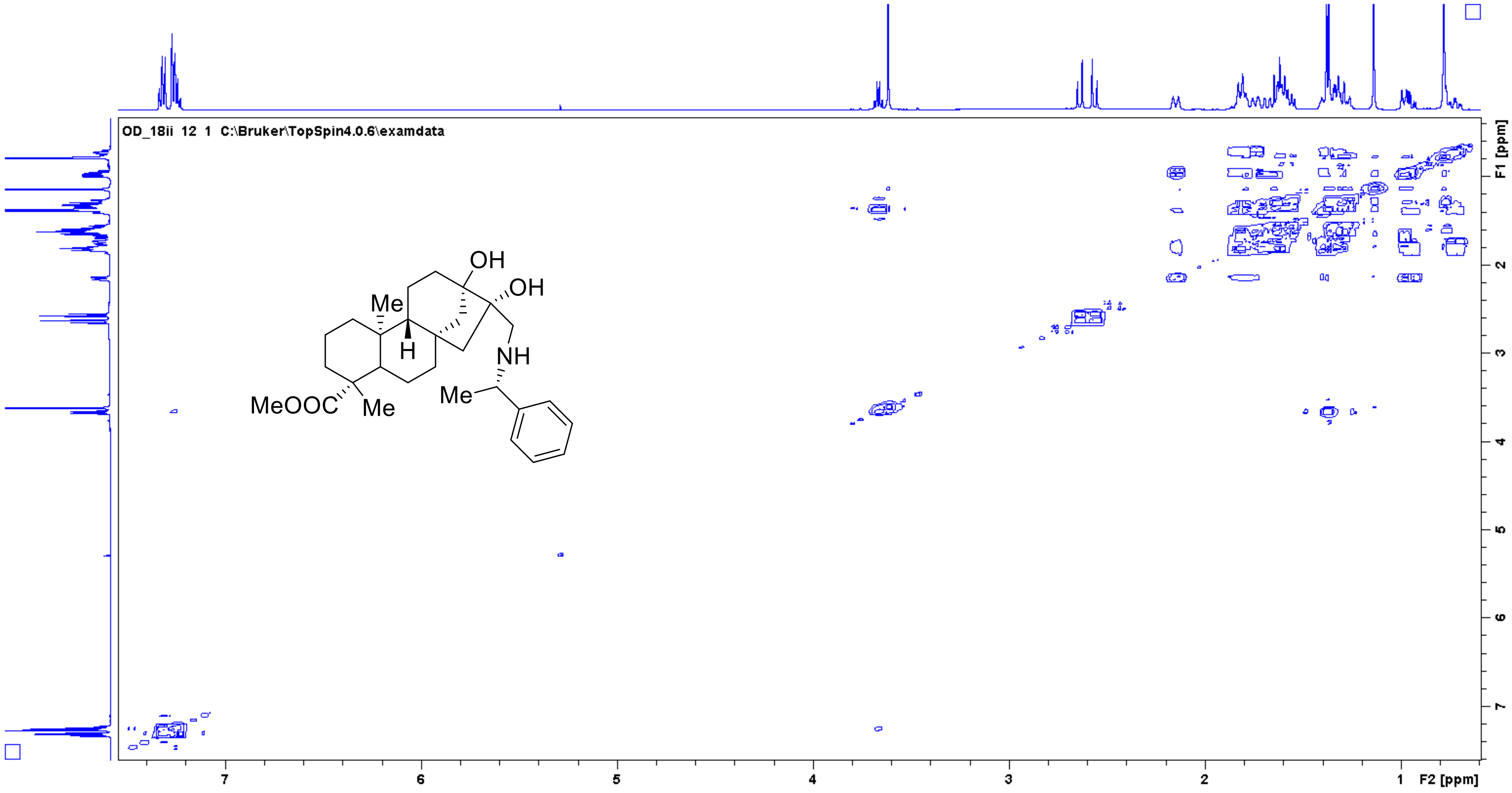

NOESY of compound **13**

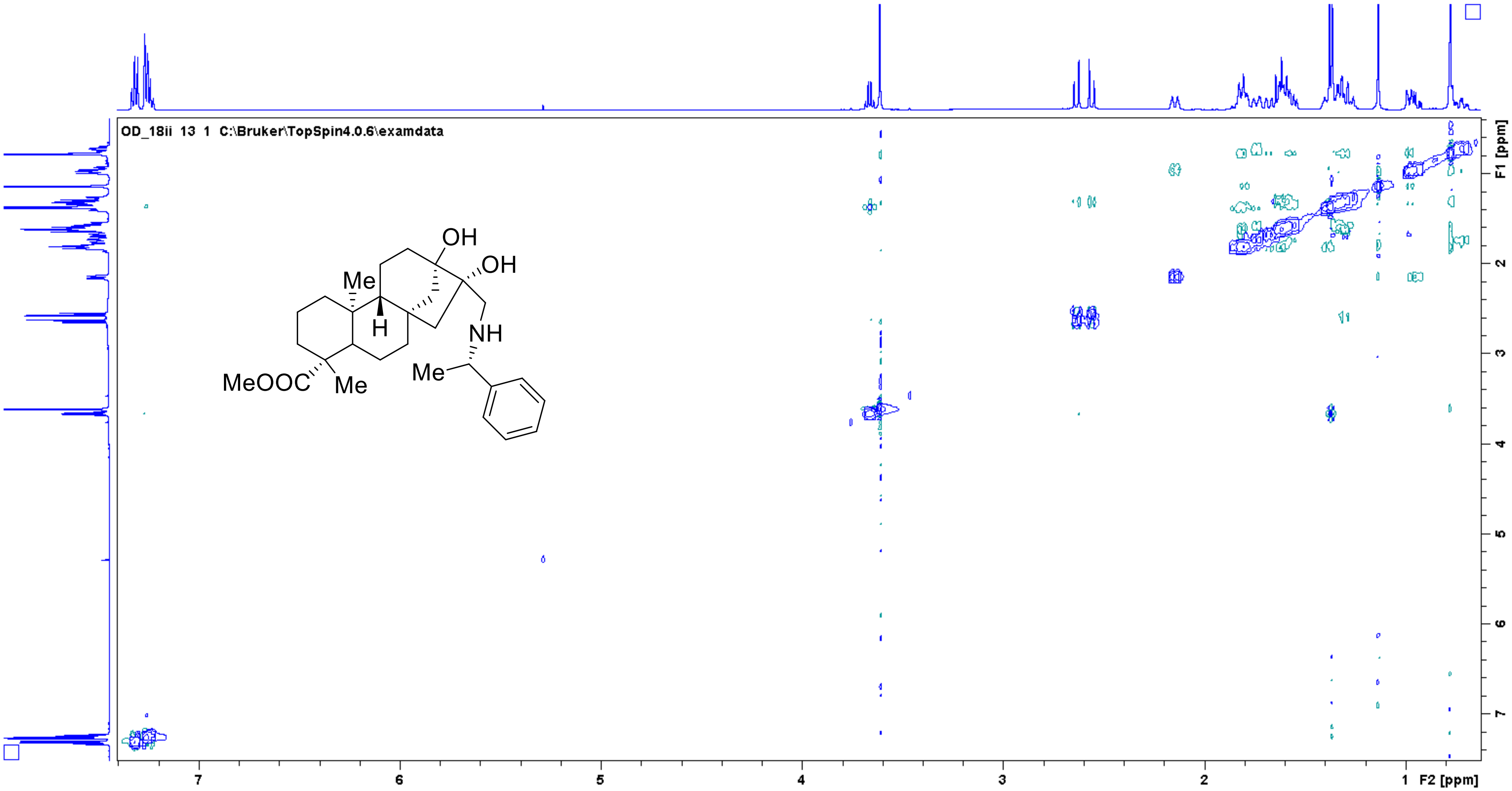

HSQC of compound **13**

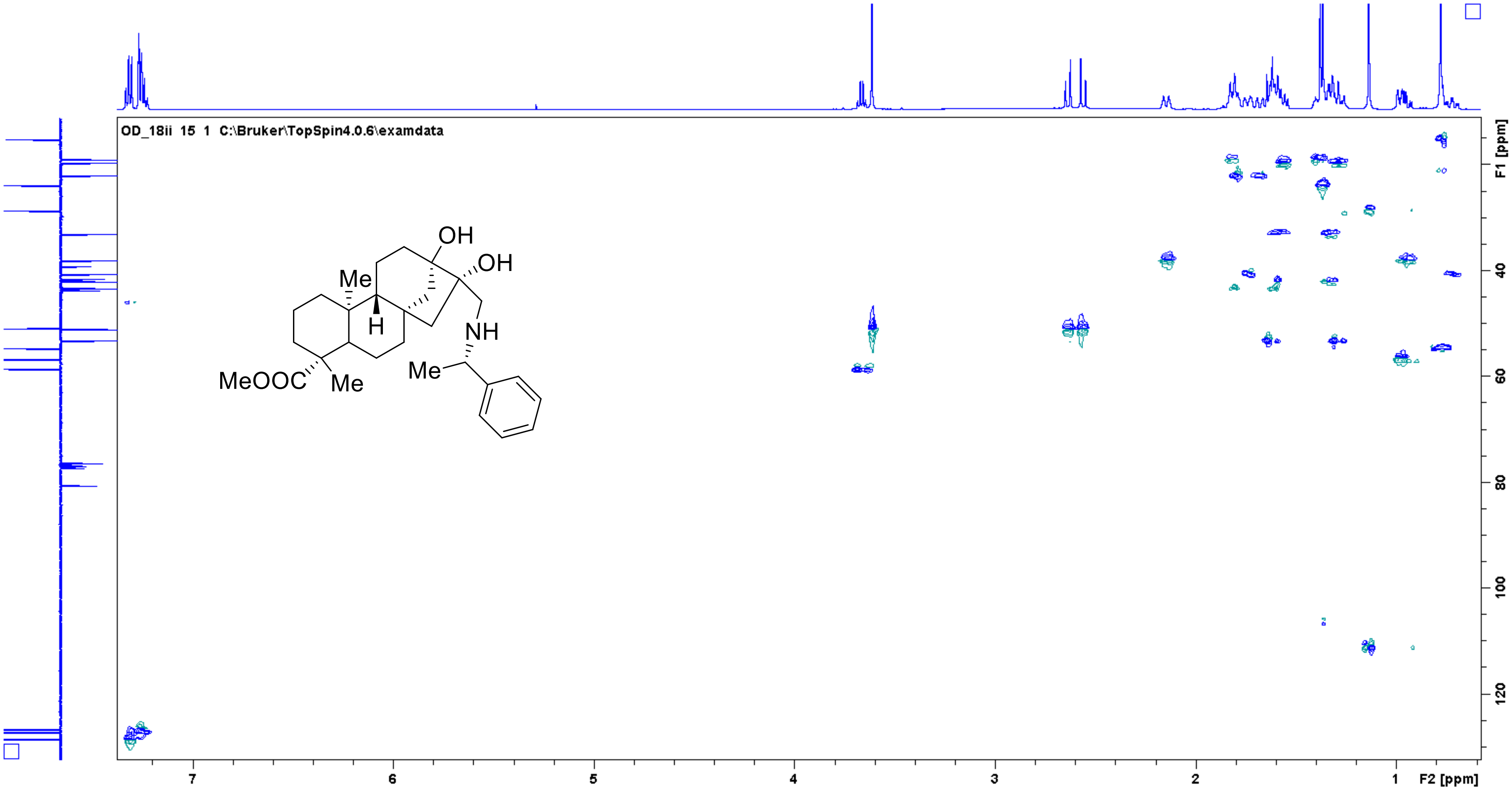

HMBC of compound **13**

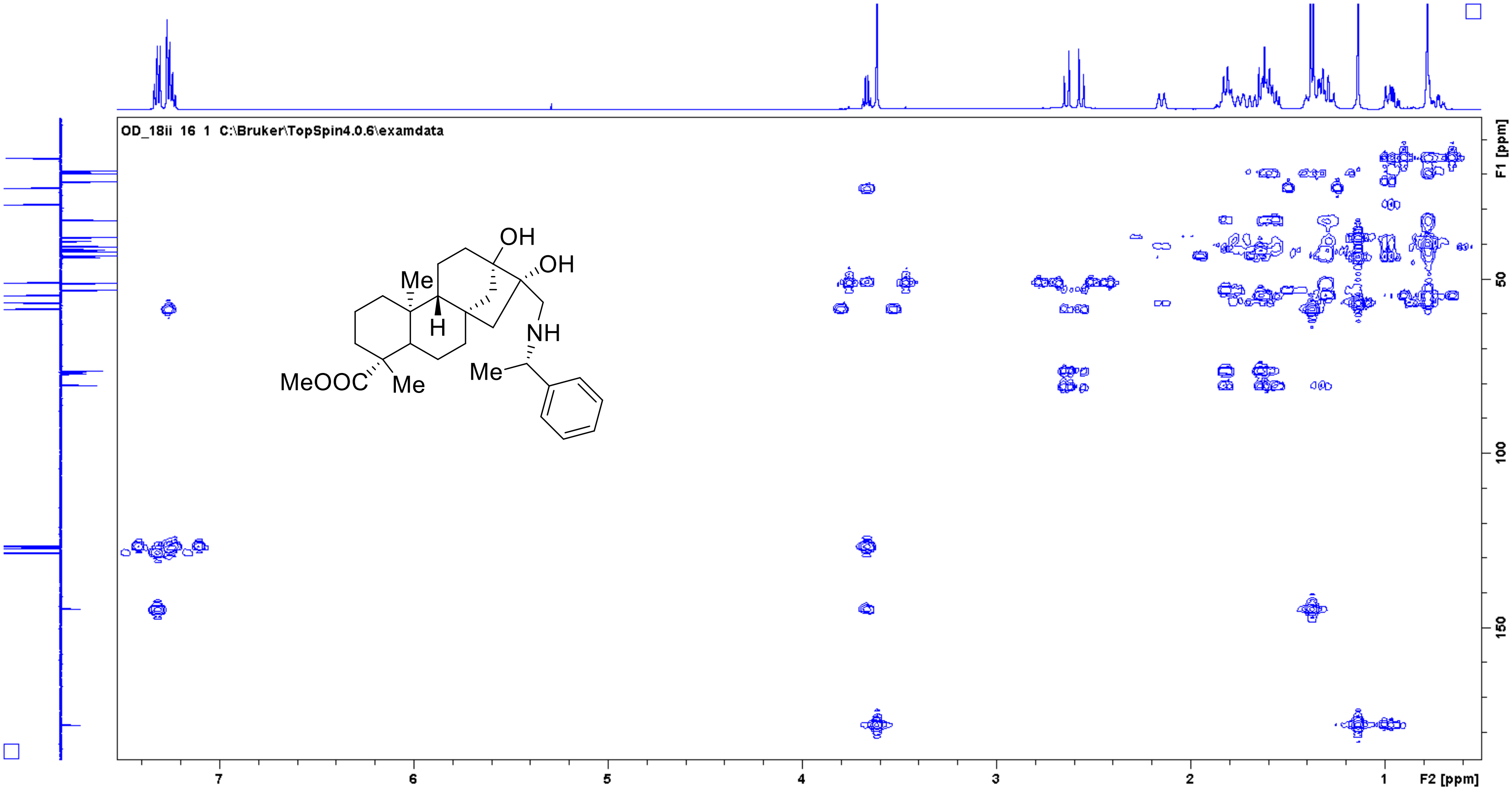

^{1}H-NMR of compound **14**

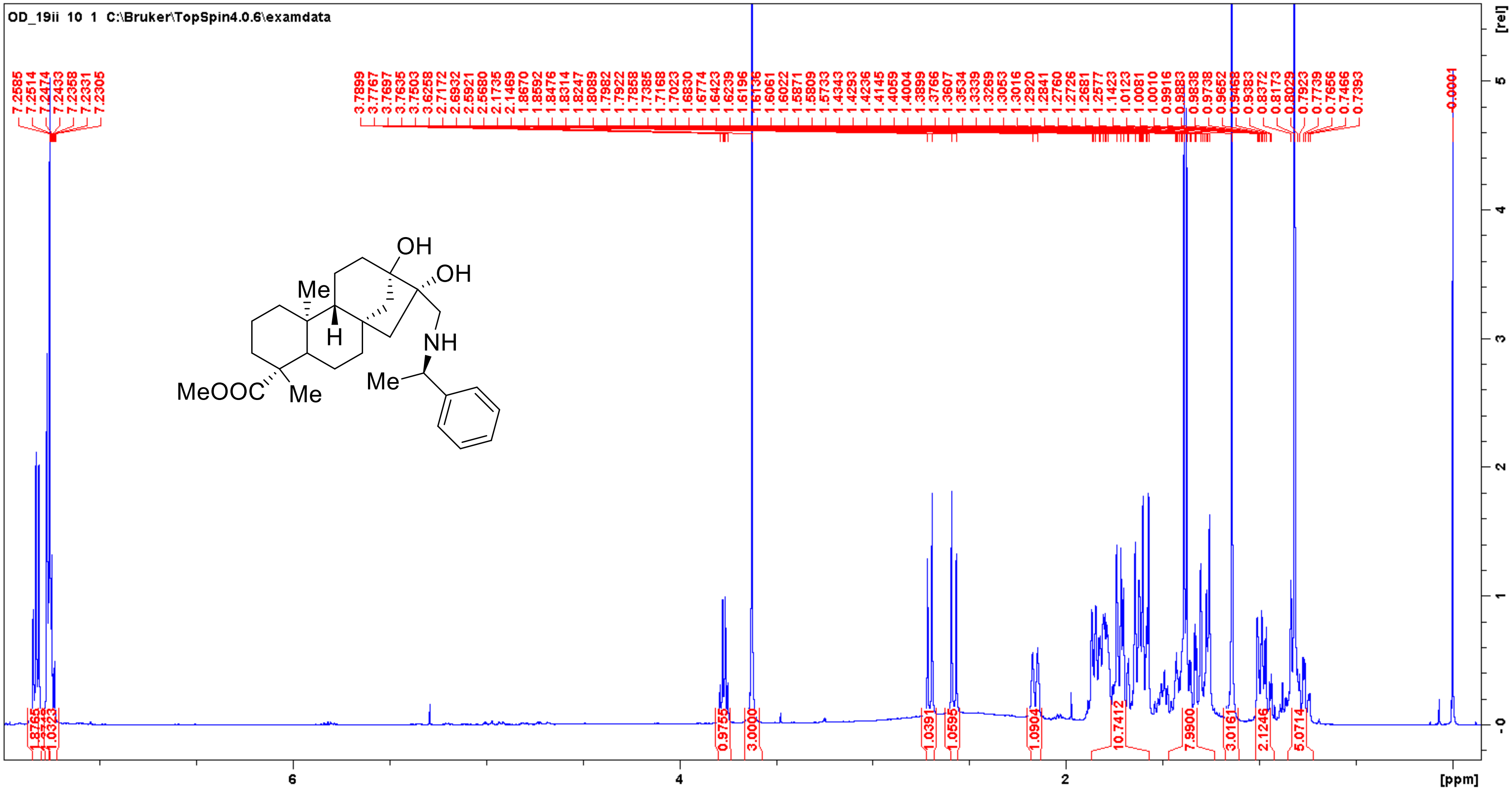

^{13}C-NMR of compound **14**

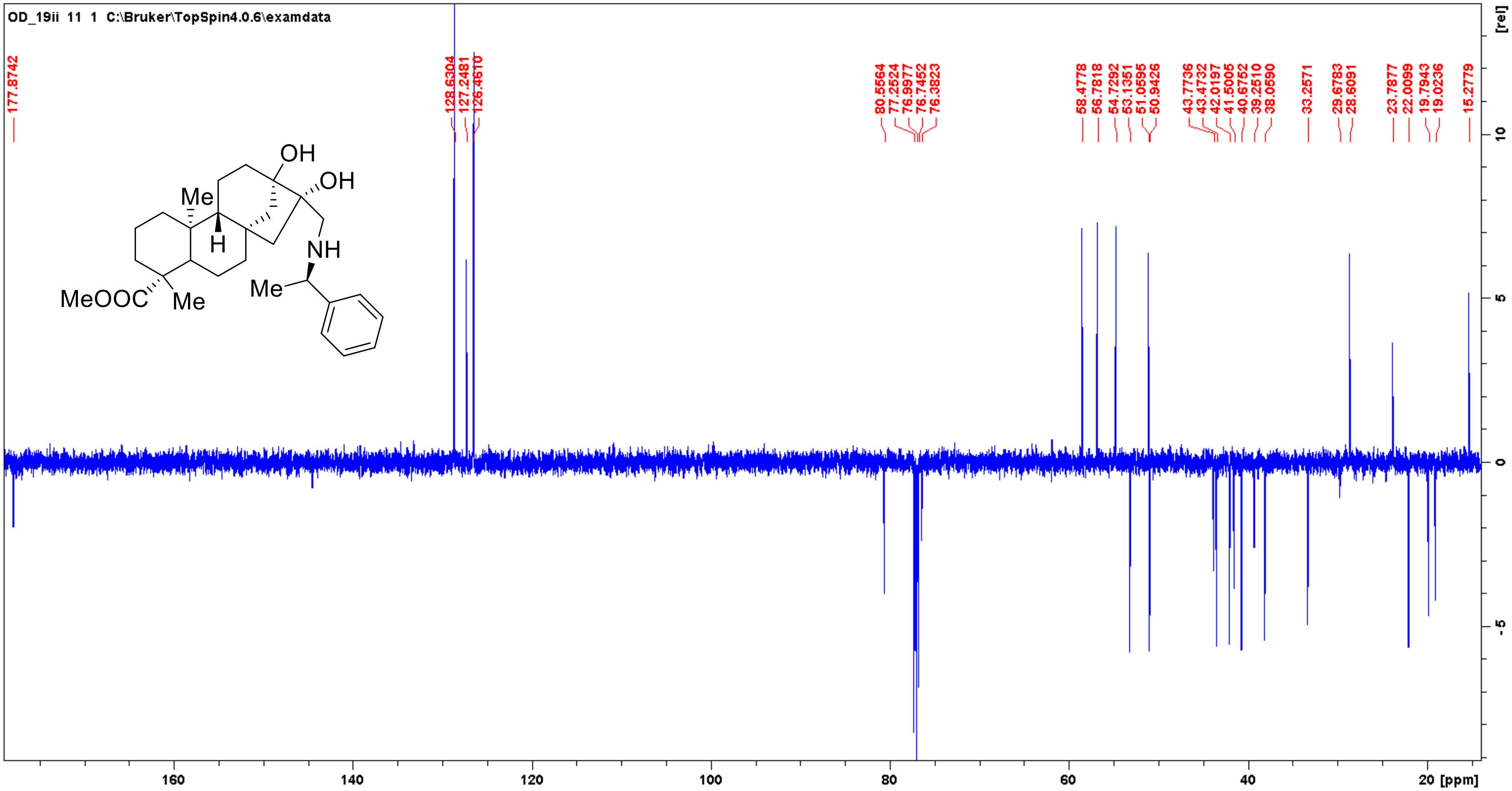

COSY of compound **14**

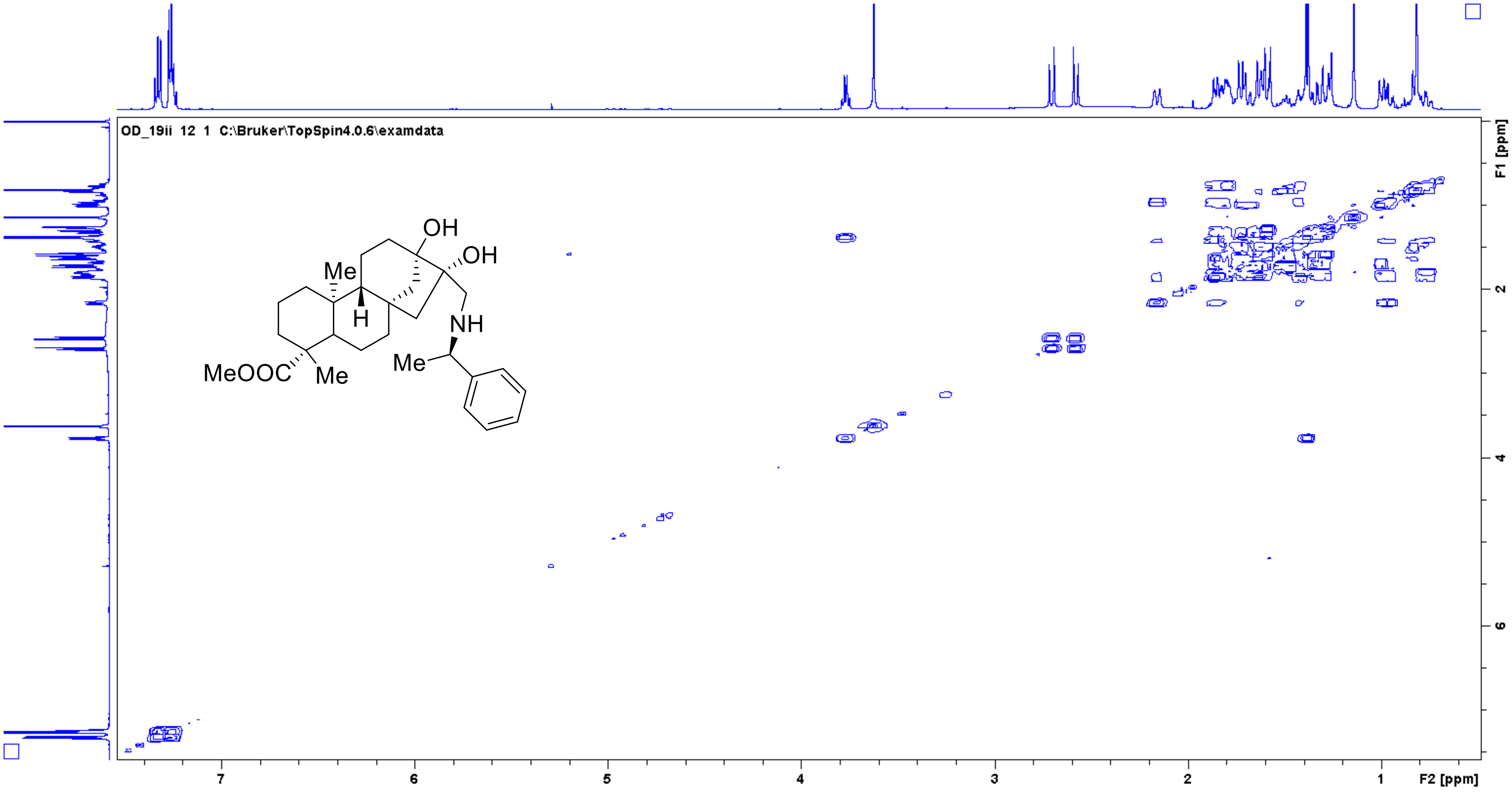

NOESY of compound **14**

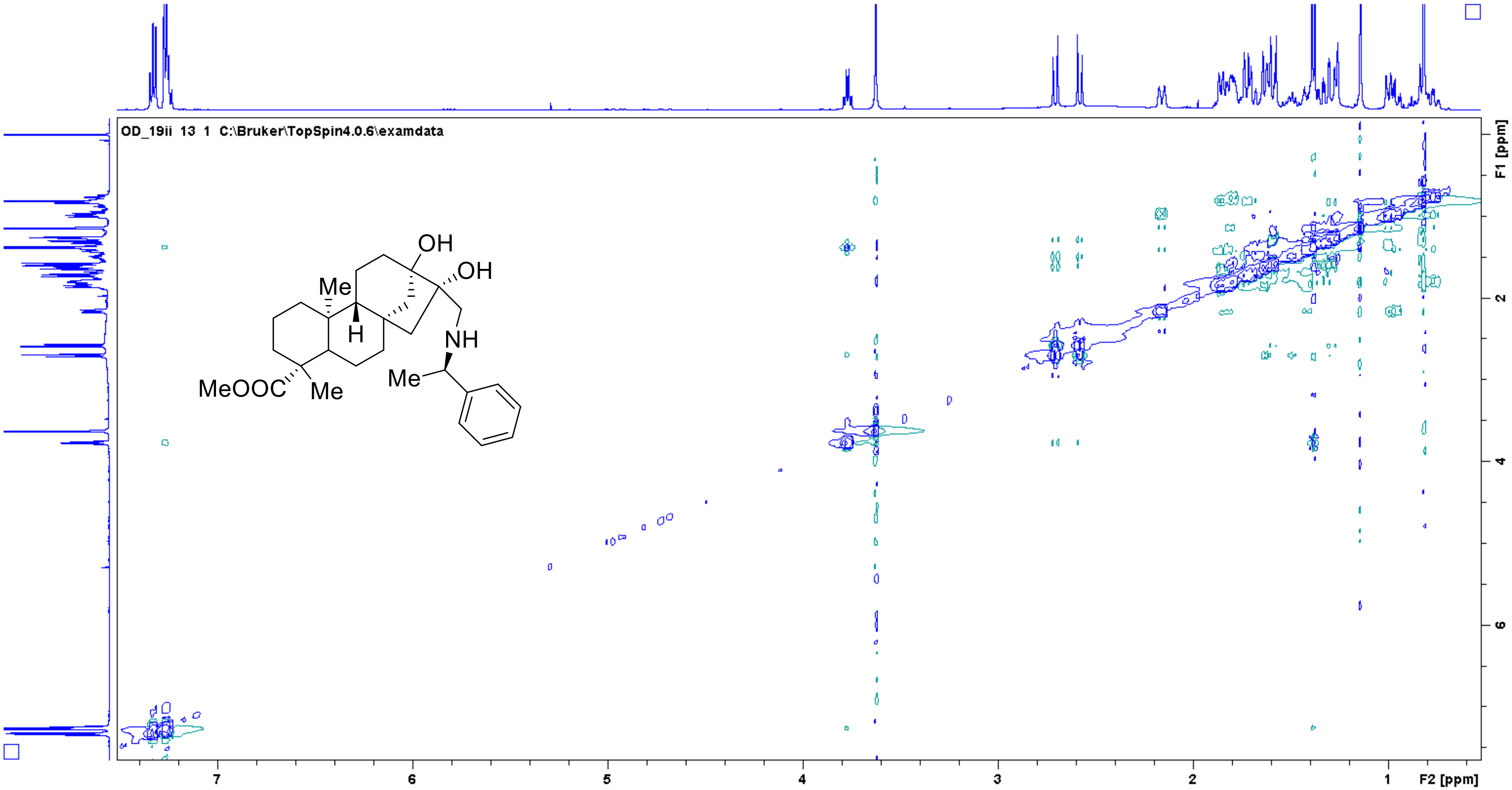

HSQC of compound **14**

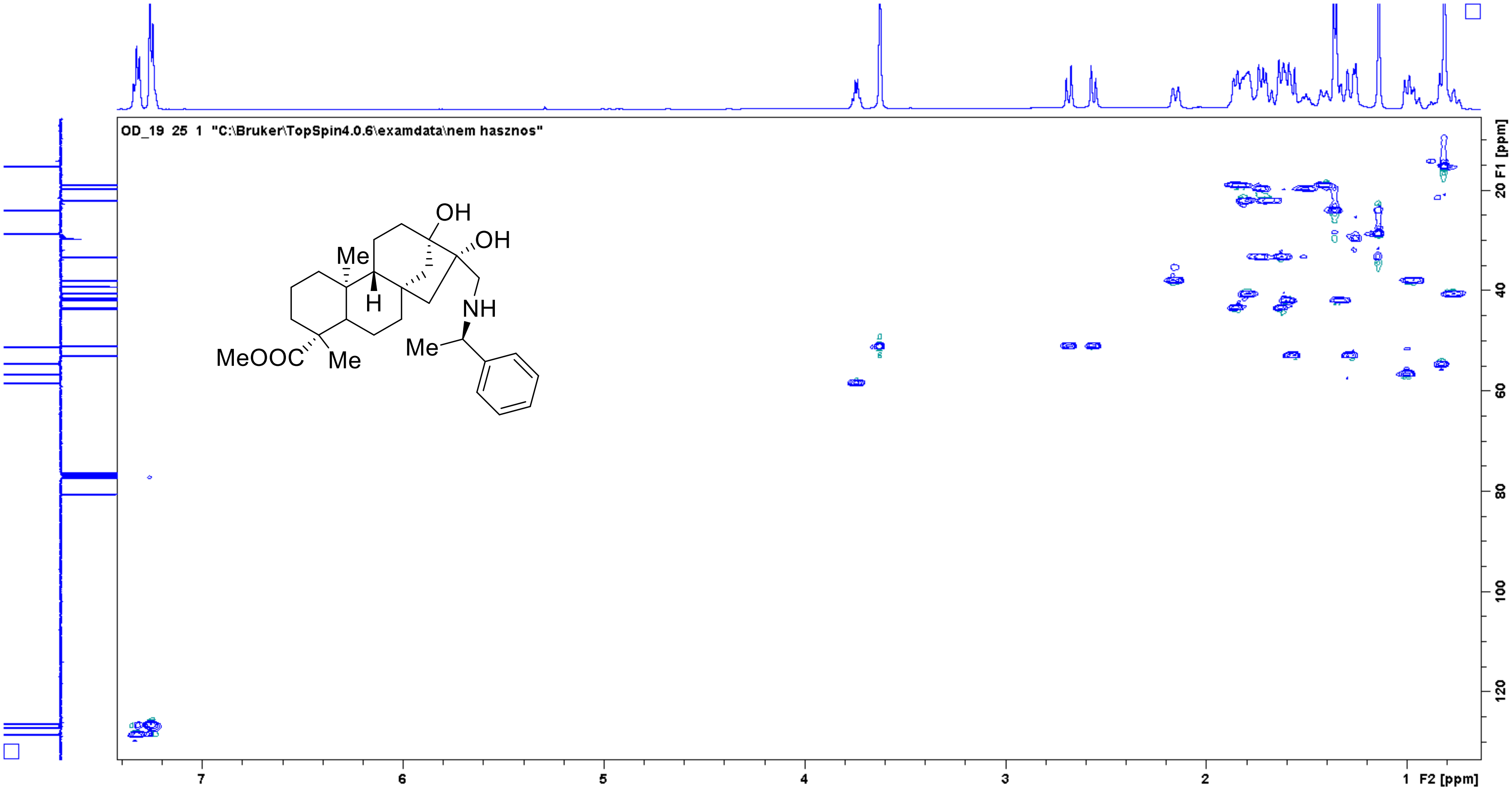

HMBC of compound **14**

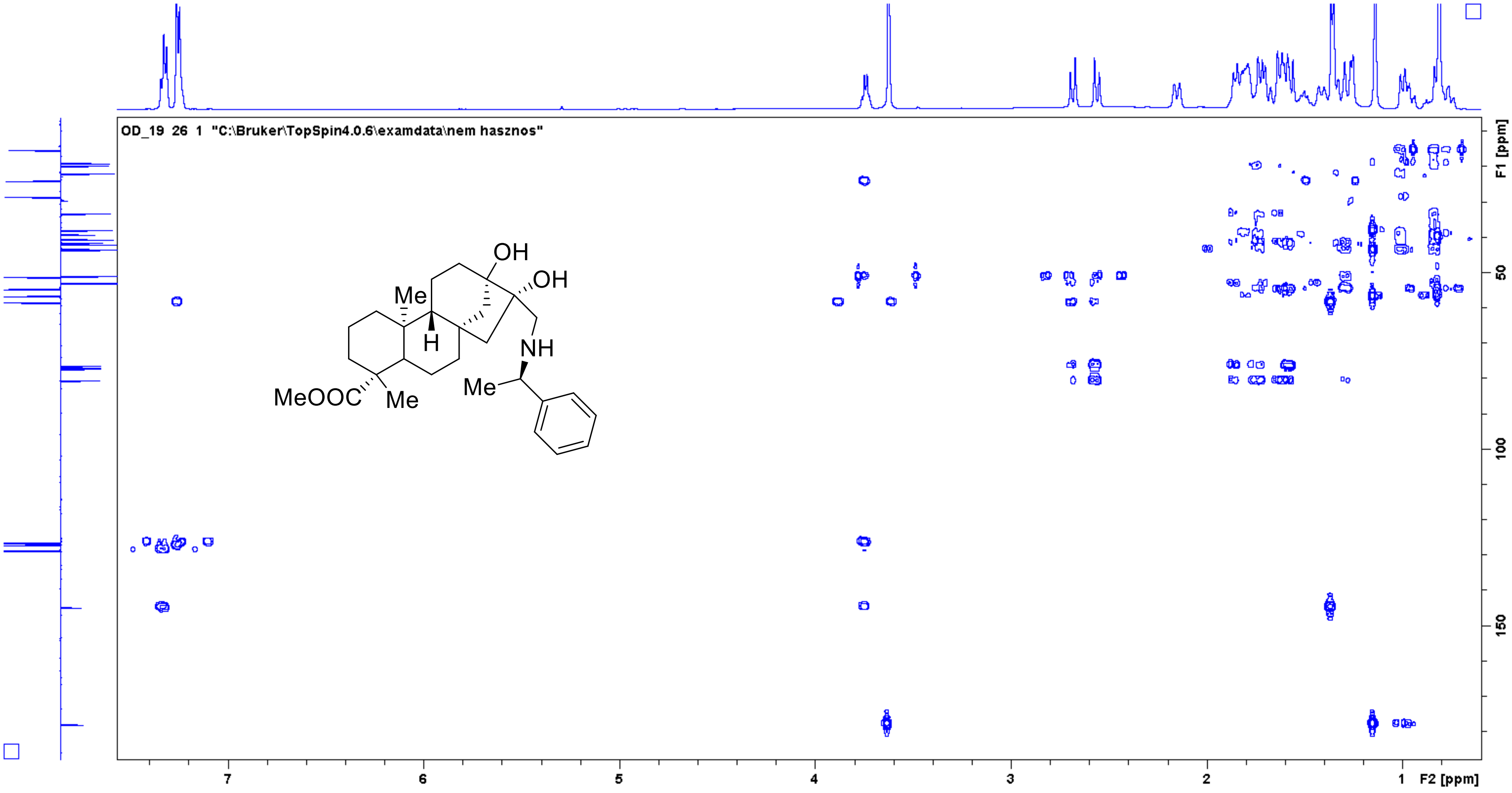

^{1}H-NMR of compound **15**

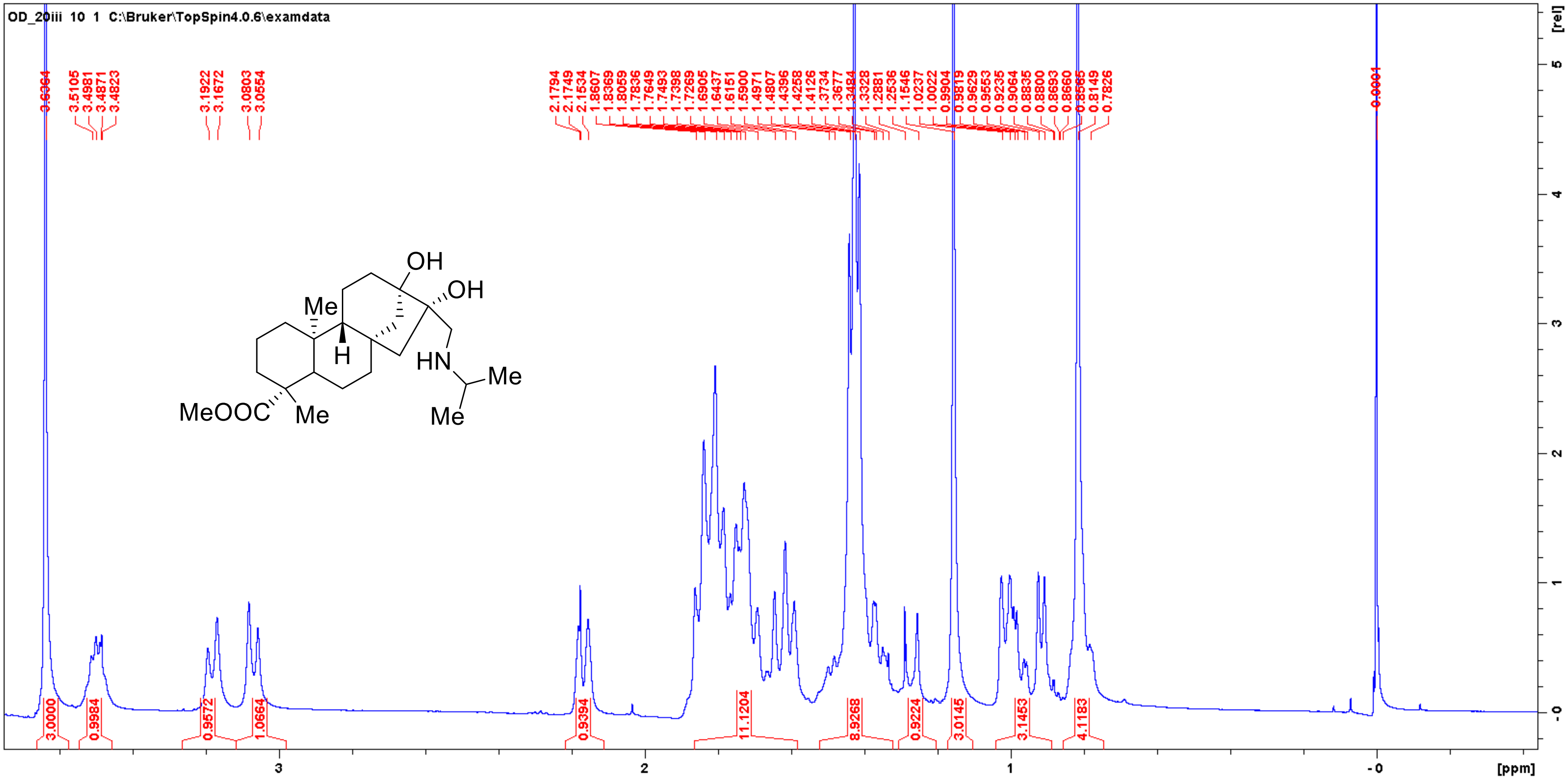

^{13}C-NMR of compound **15**

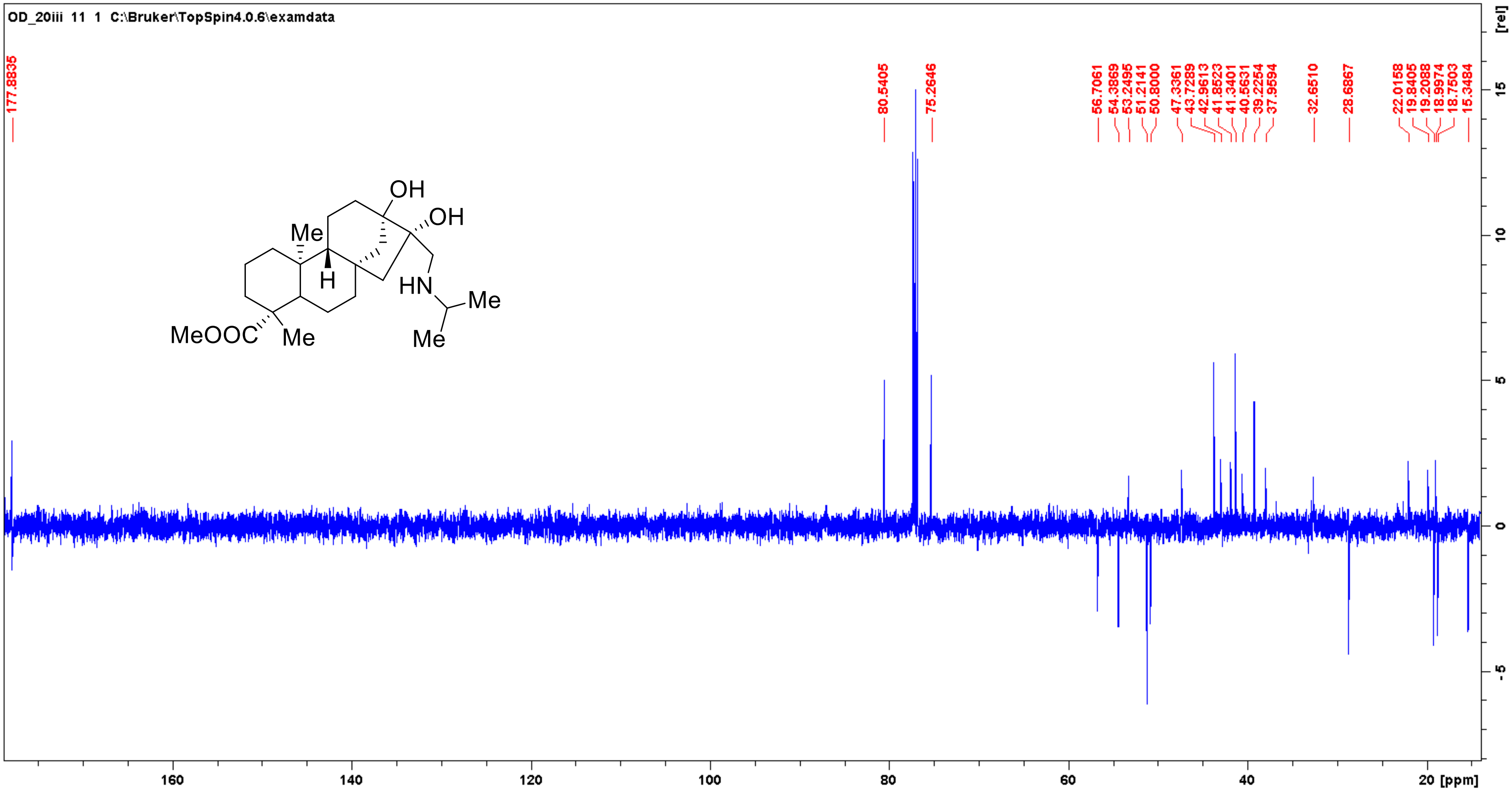

COSY of compound **15**

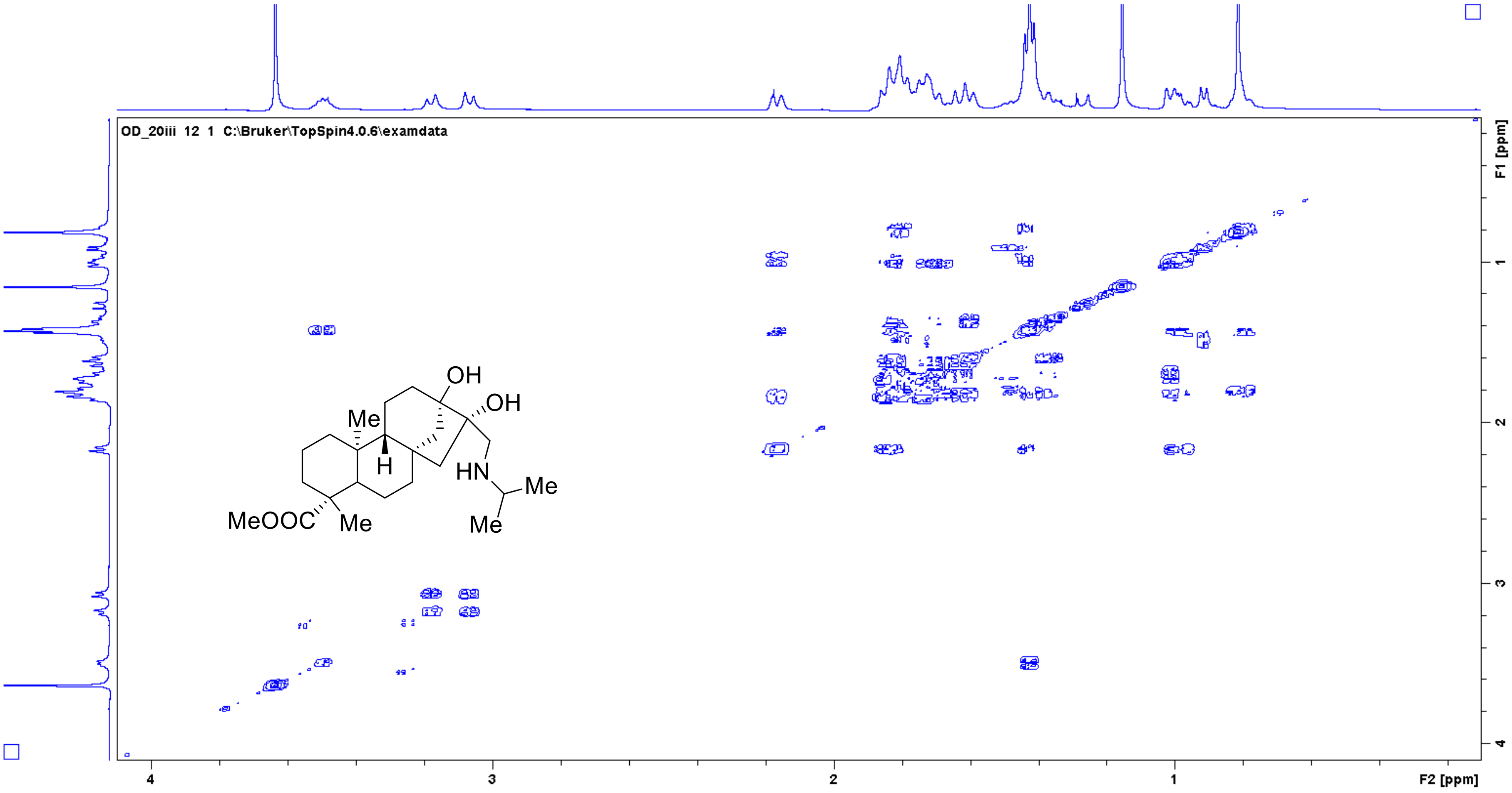

NOESY of compound **15**

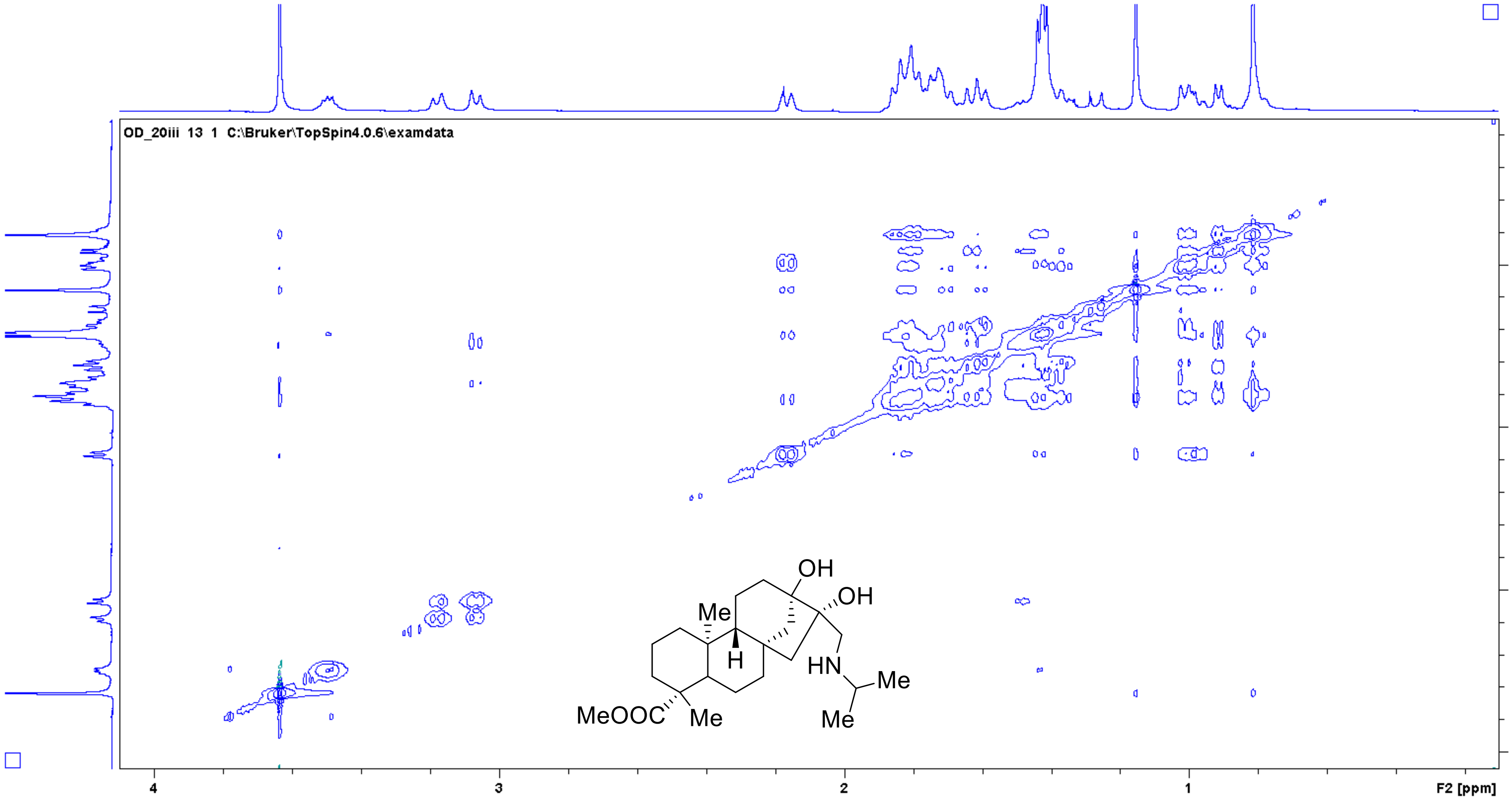

HSQC of compound **15**

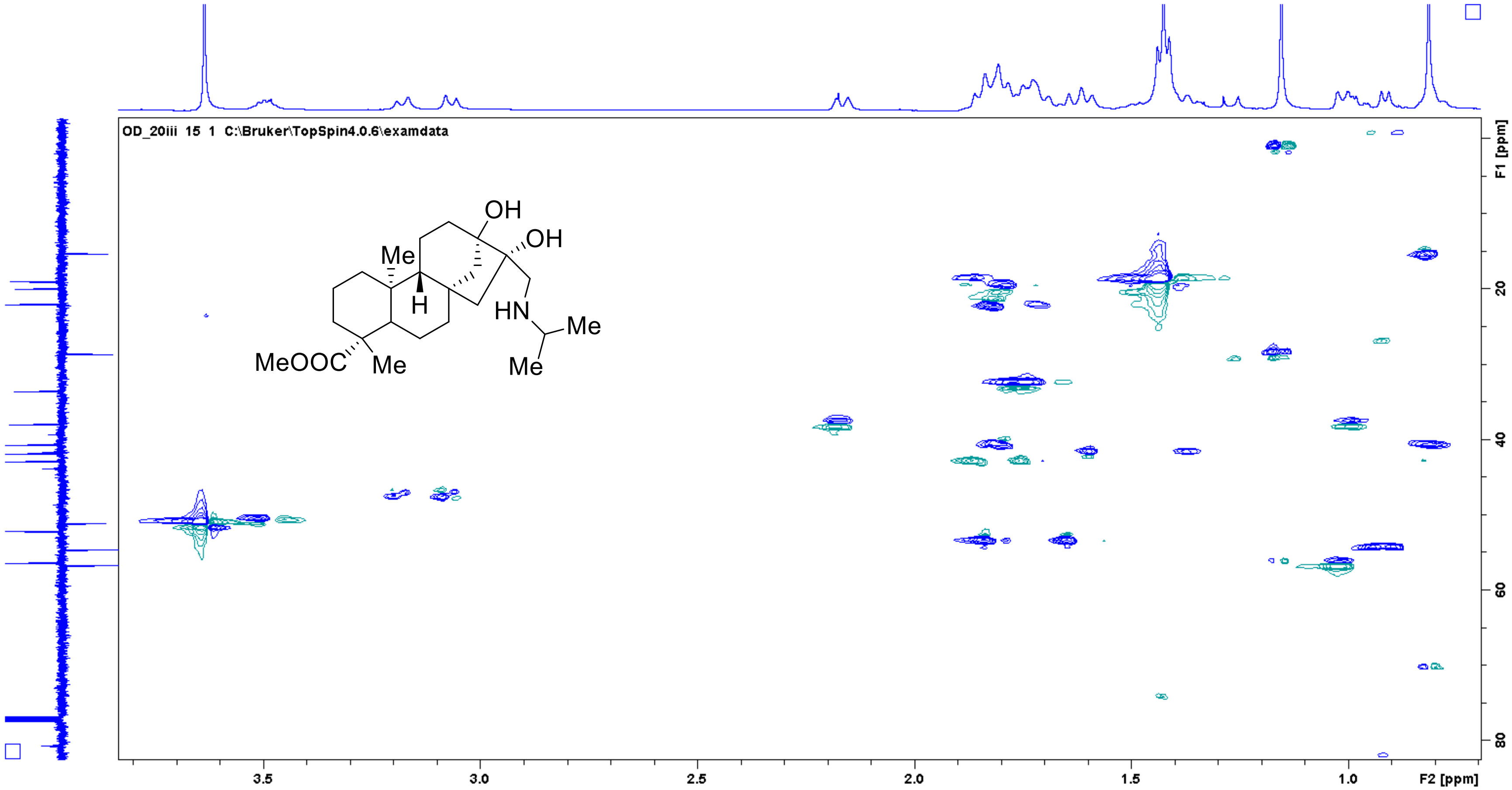

HMBC of compound **15**

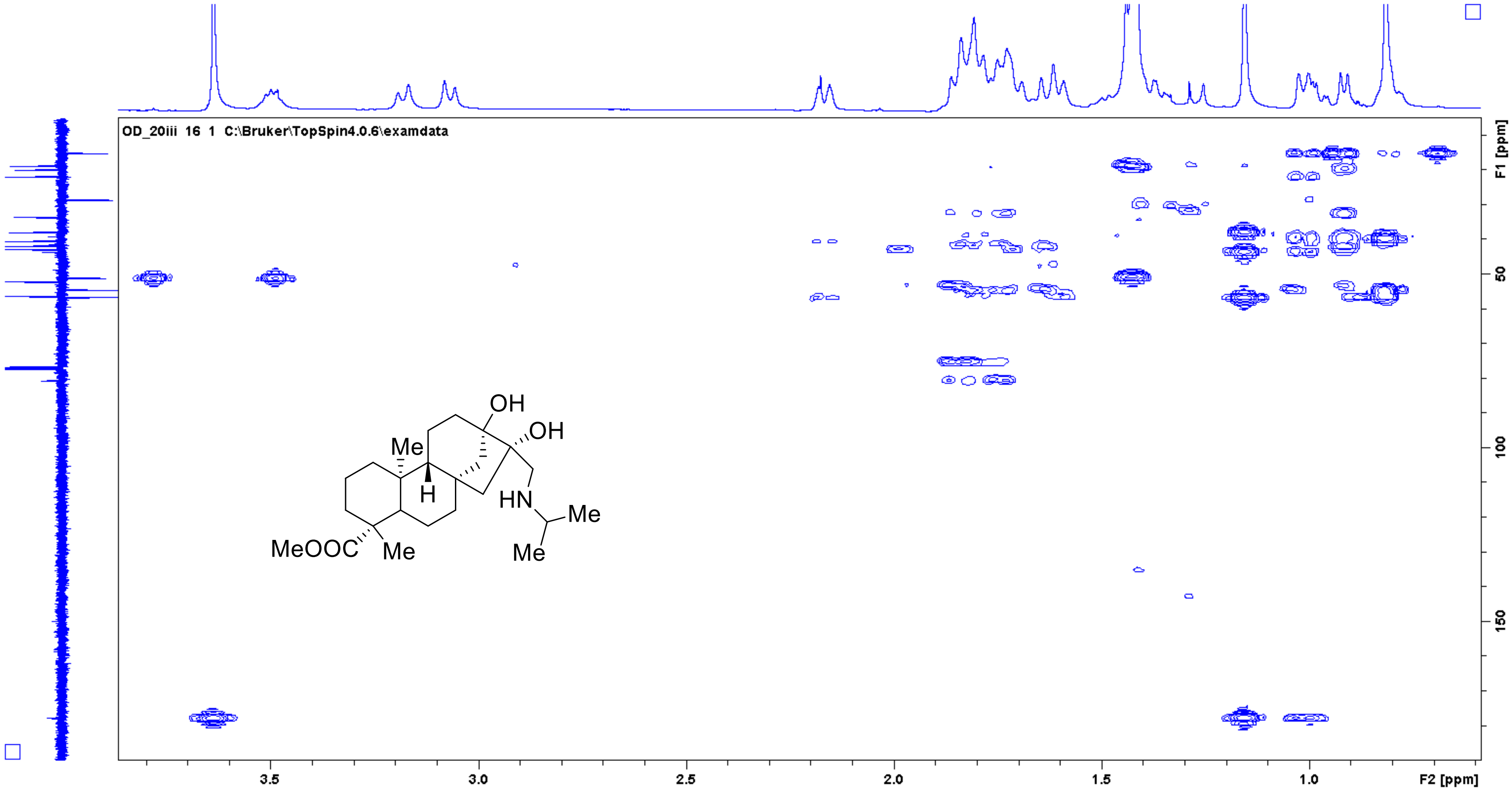

^{1}H-NMR of compound **16**

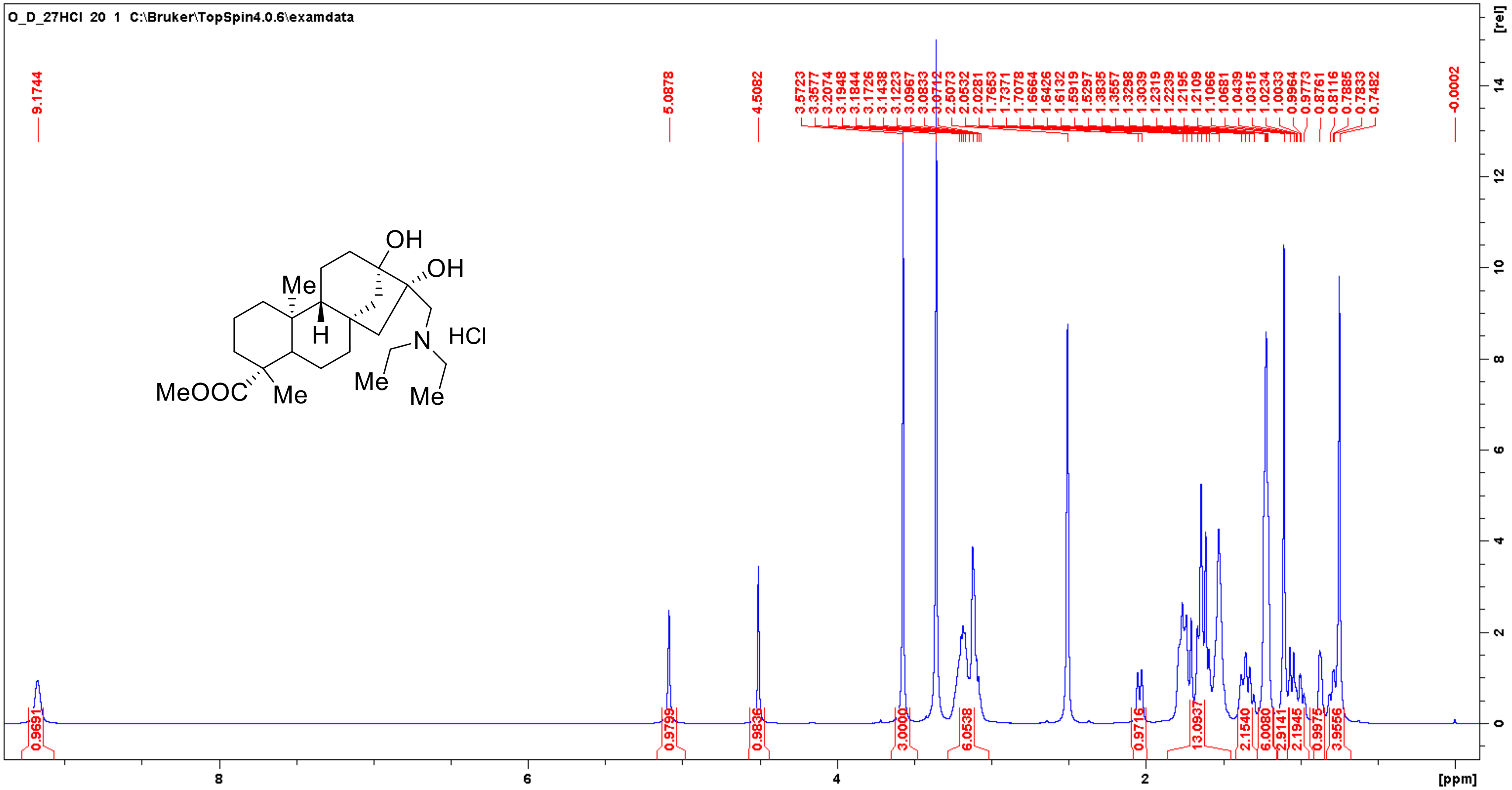

^{13}C-NMR of compound **16**

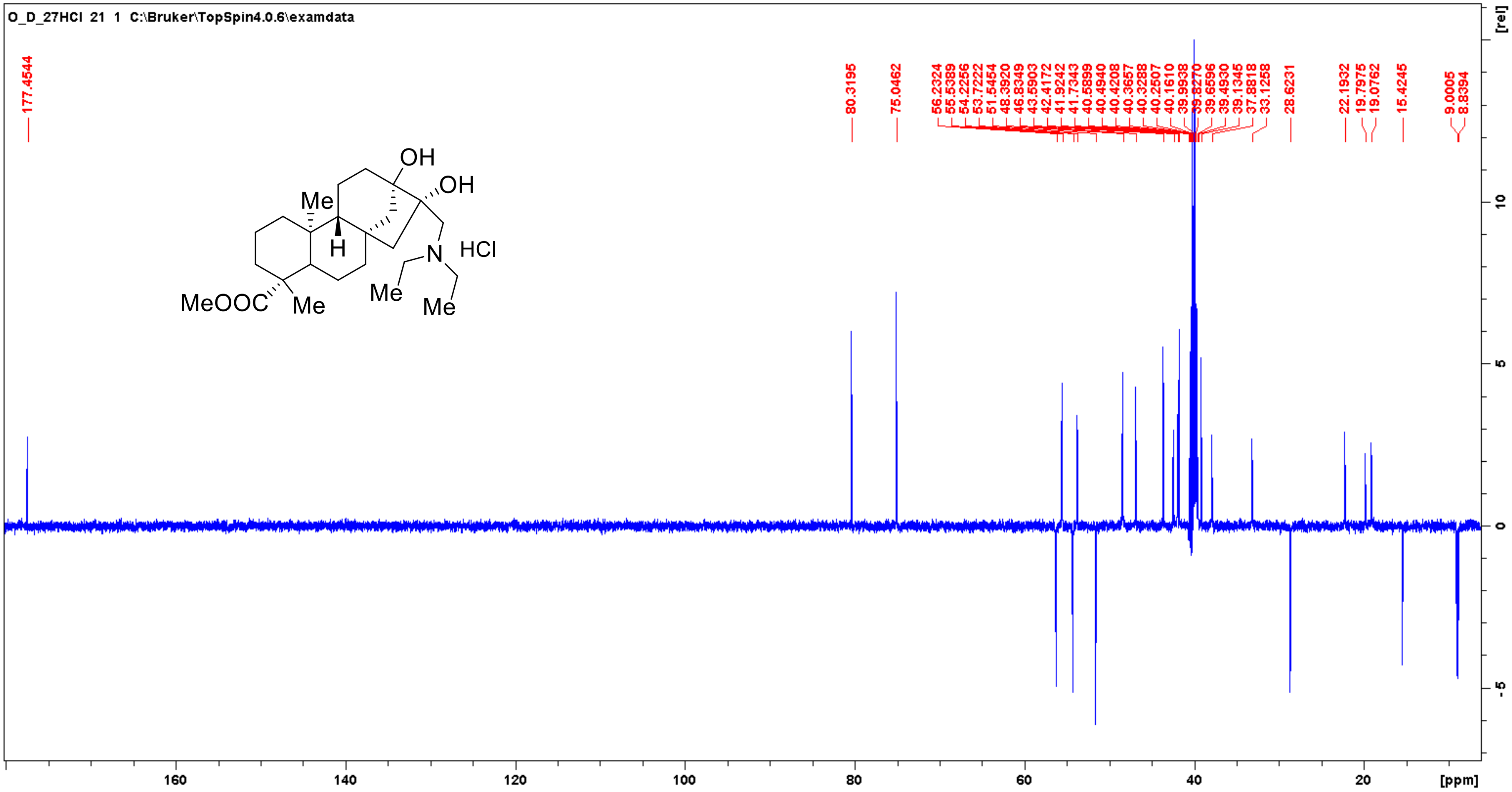

COSY of compound **16**

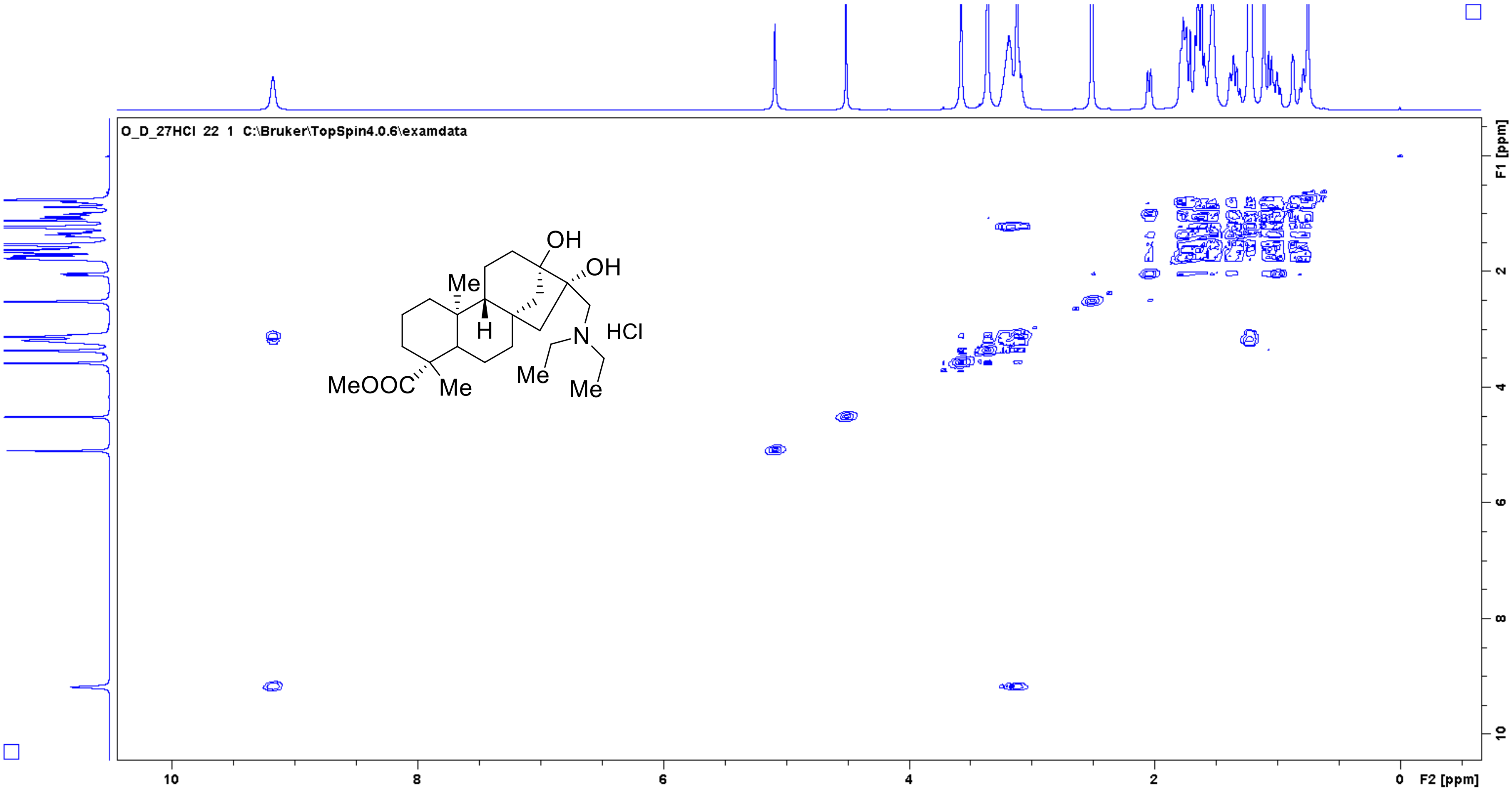

NOESY of compound **16**

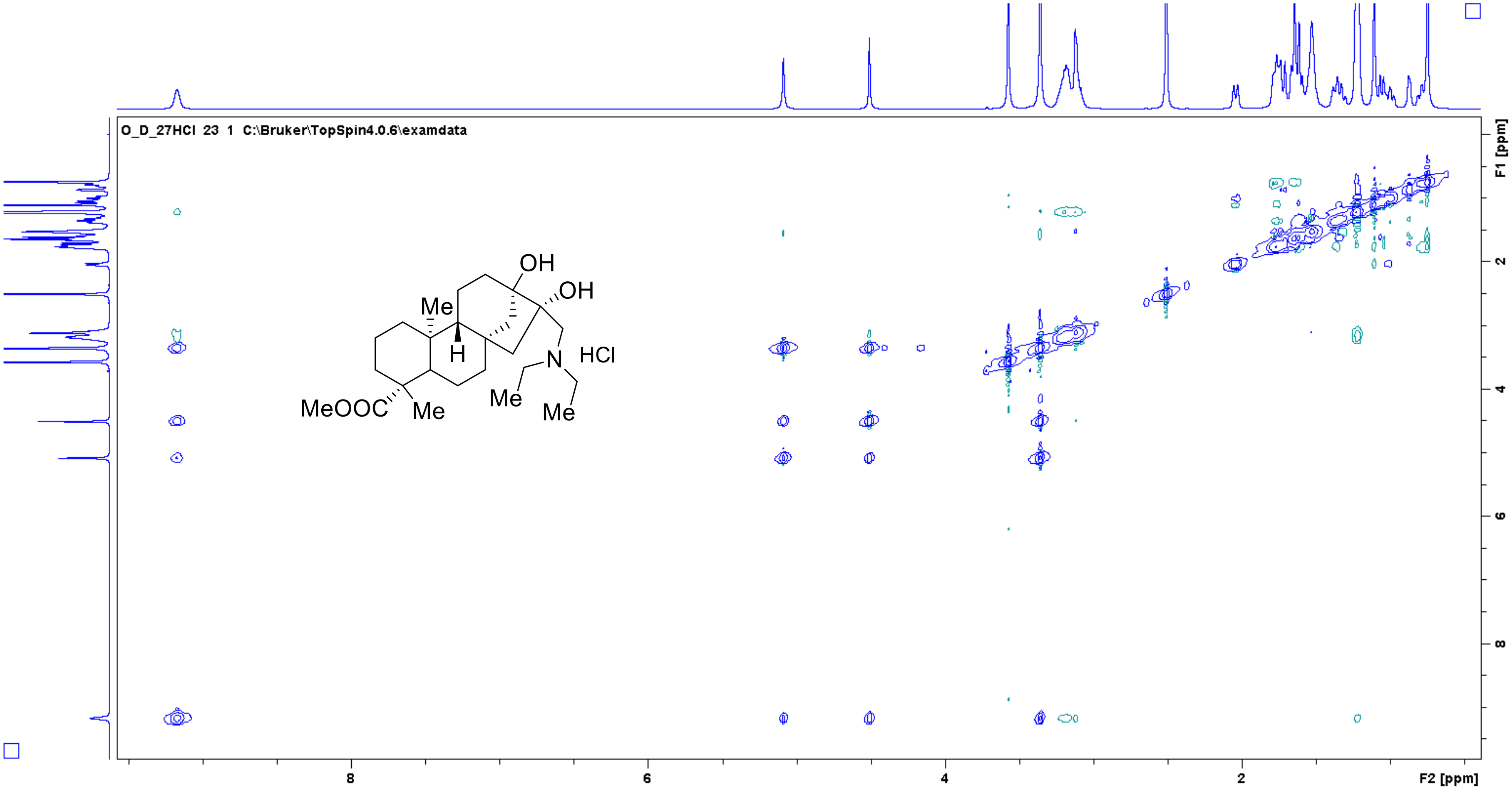

HSQC of compound **16**

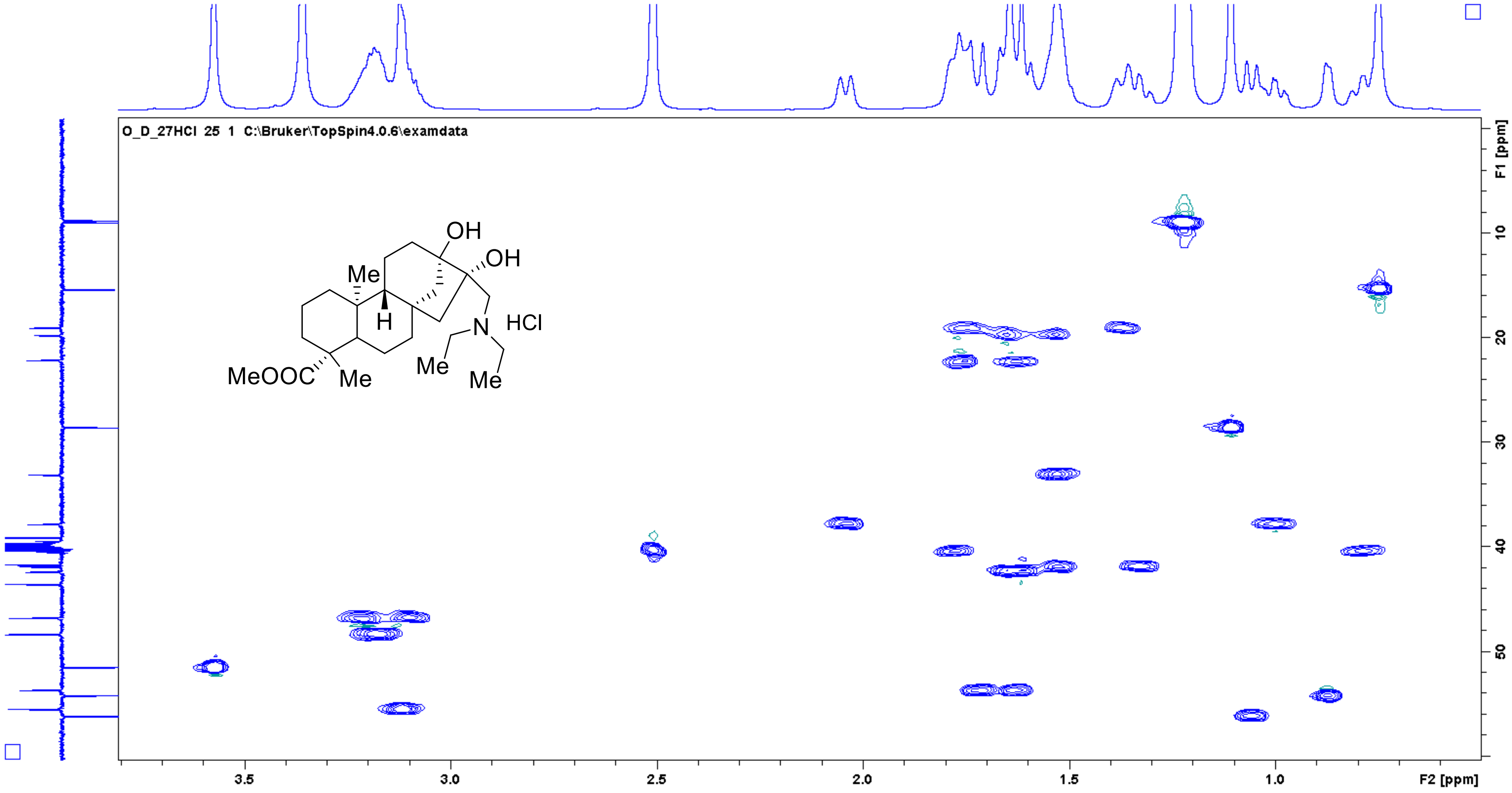

HMBC of compound **16**

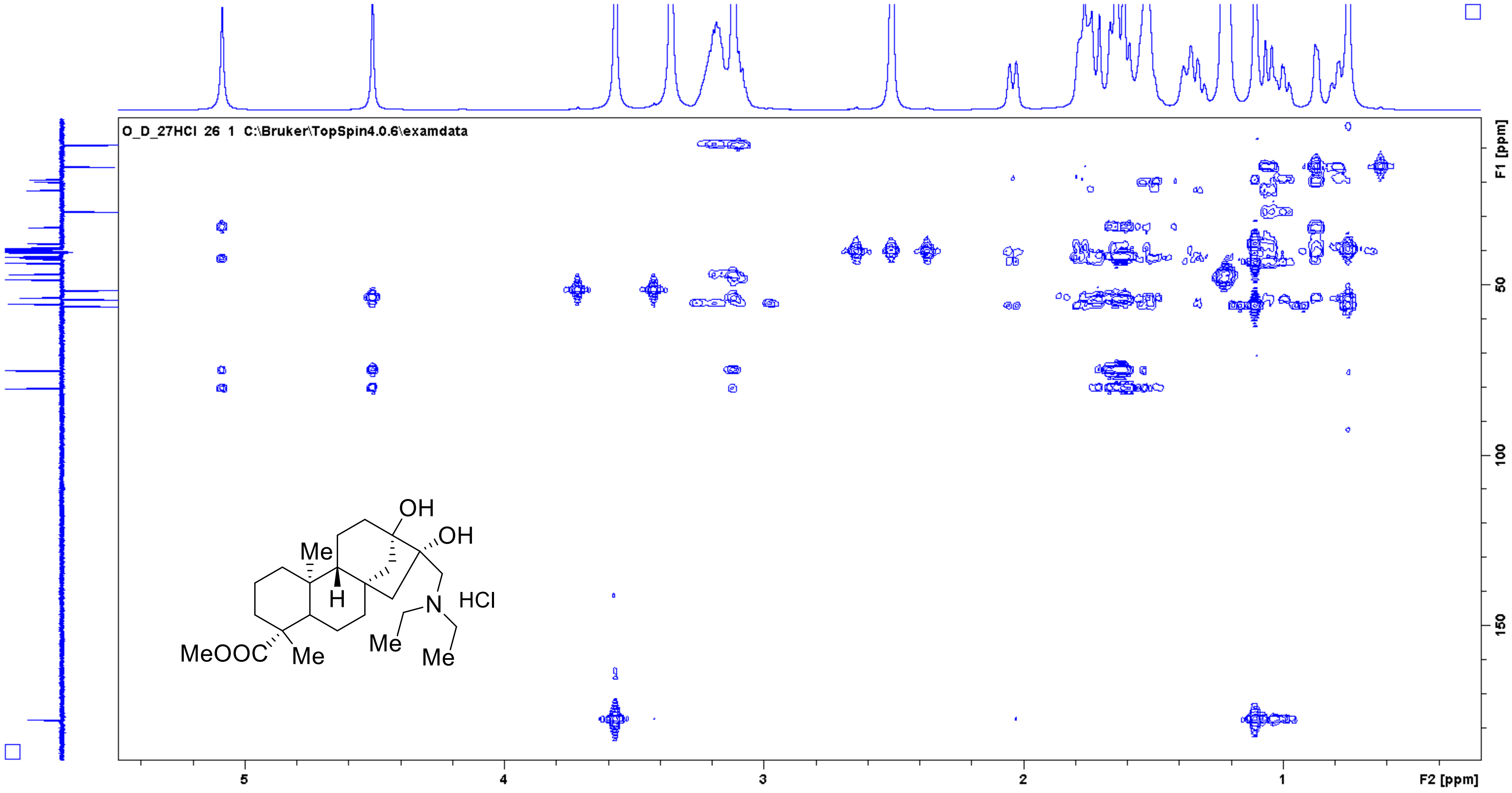

^{1}H-NMR of compound **17**

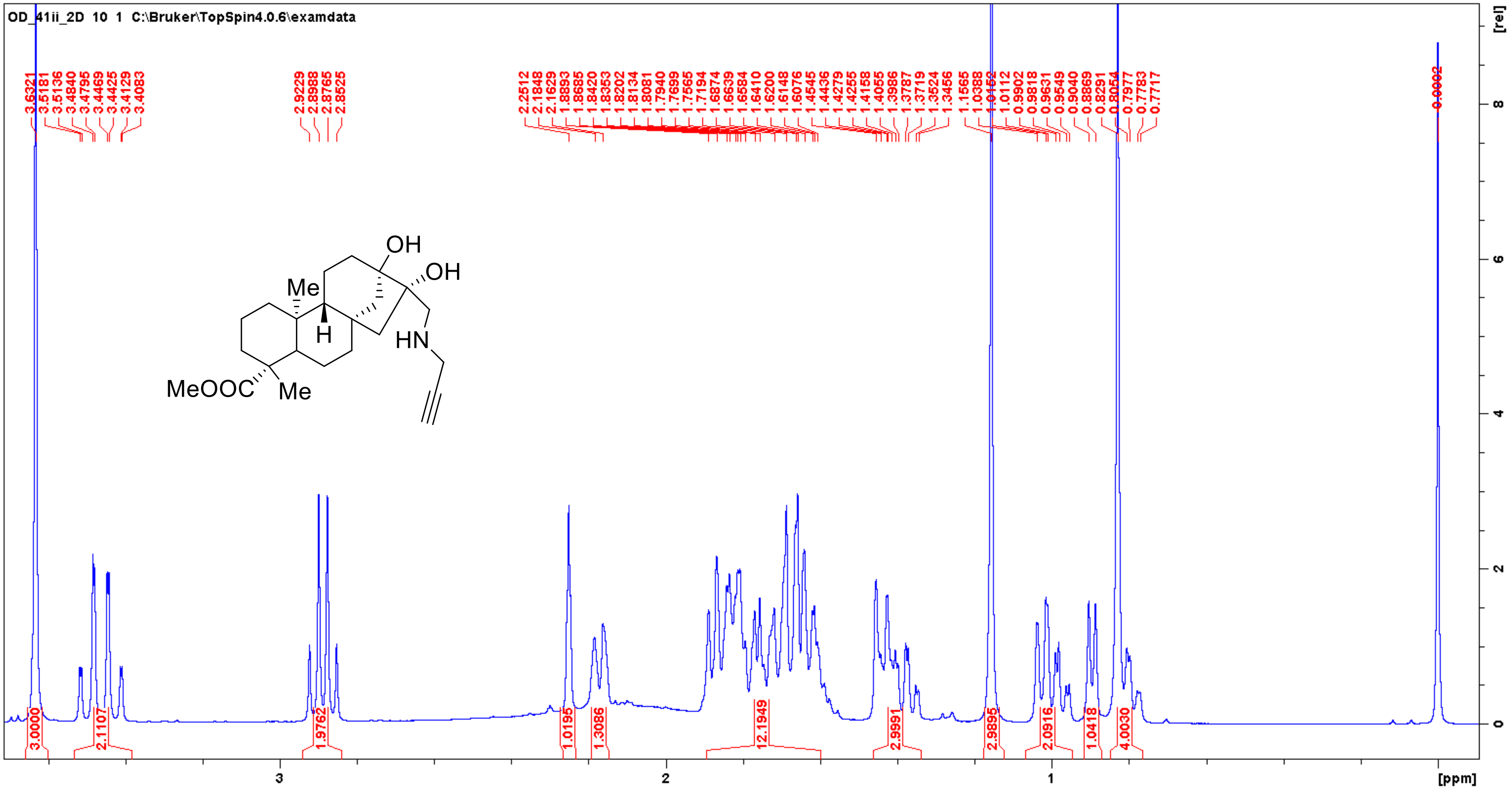

^{13}C-NMR of compound **17**

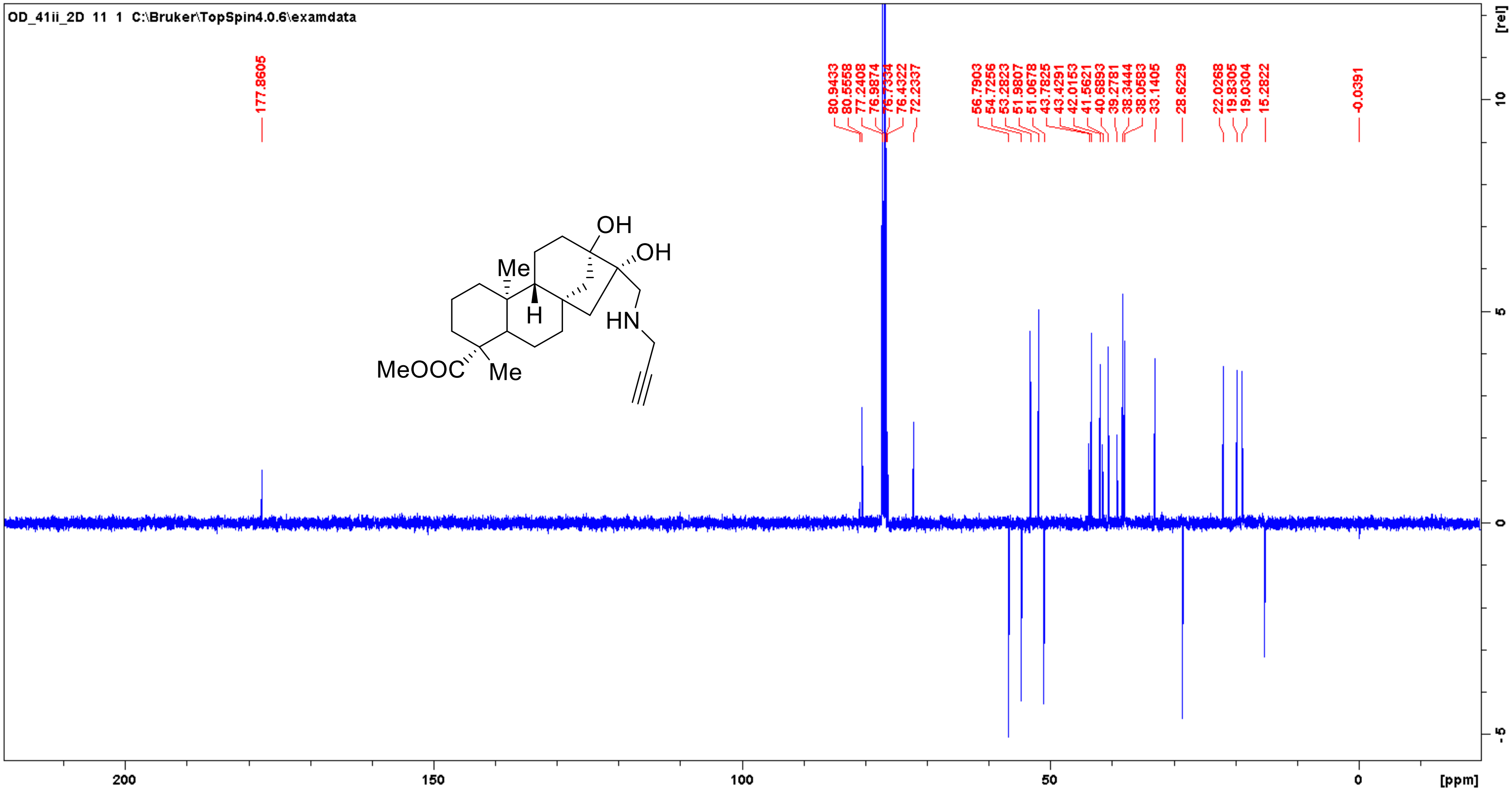

COSY of compound **17**

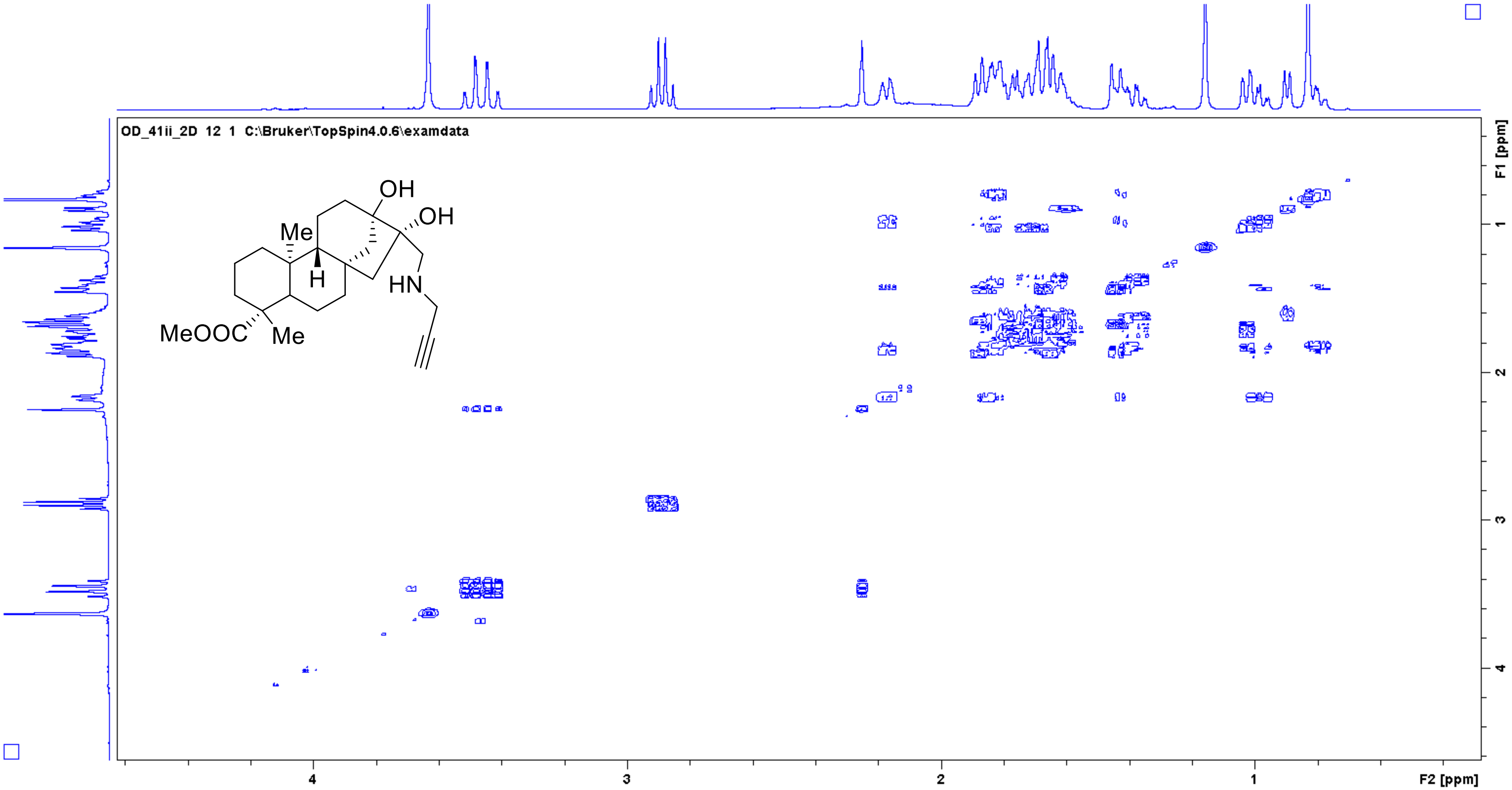

NOESY of compound **17**

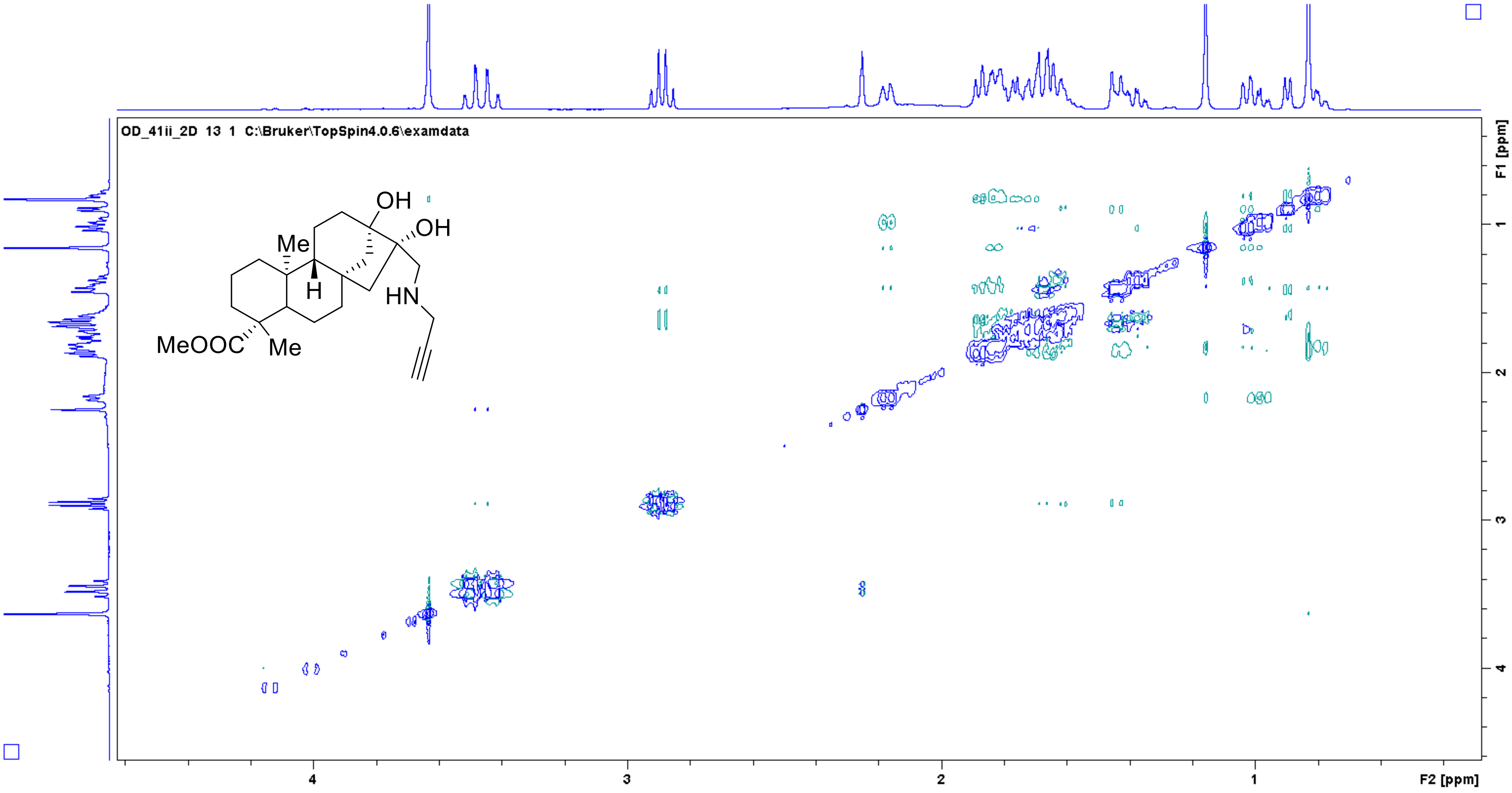

HSQC of compound **17**

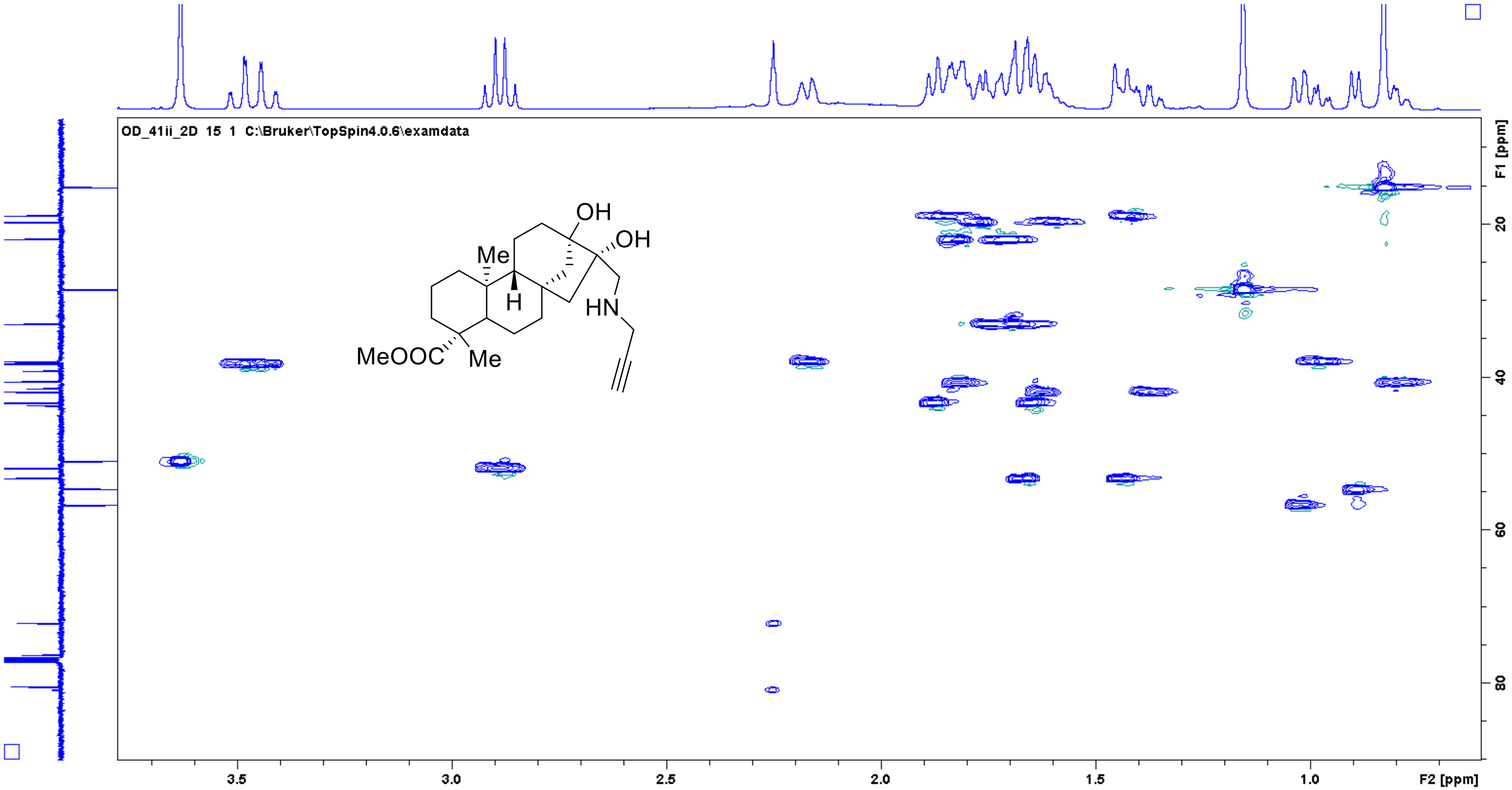

HMBC of compound **17**

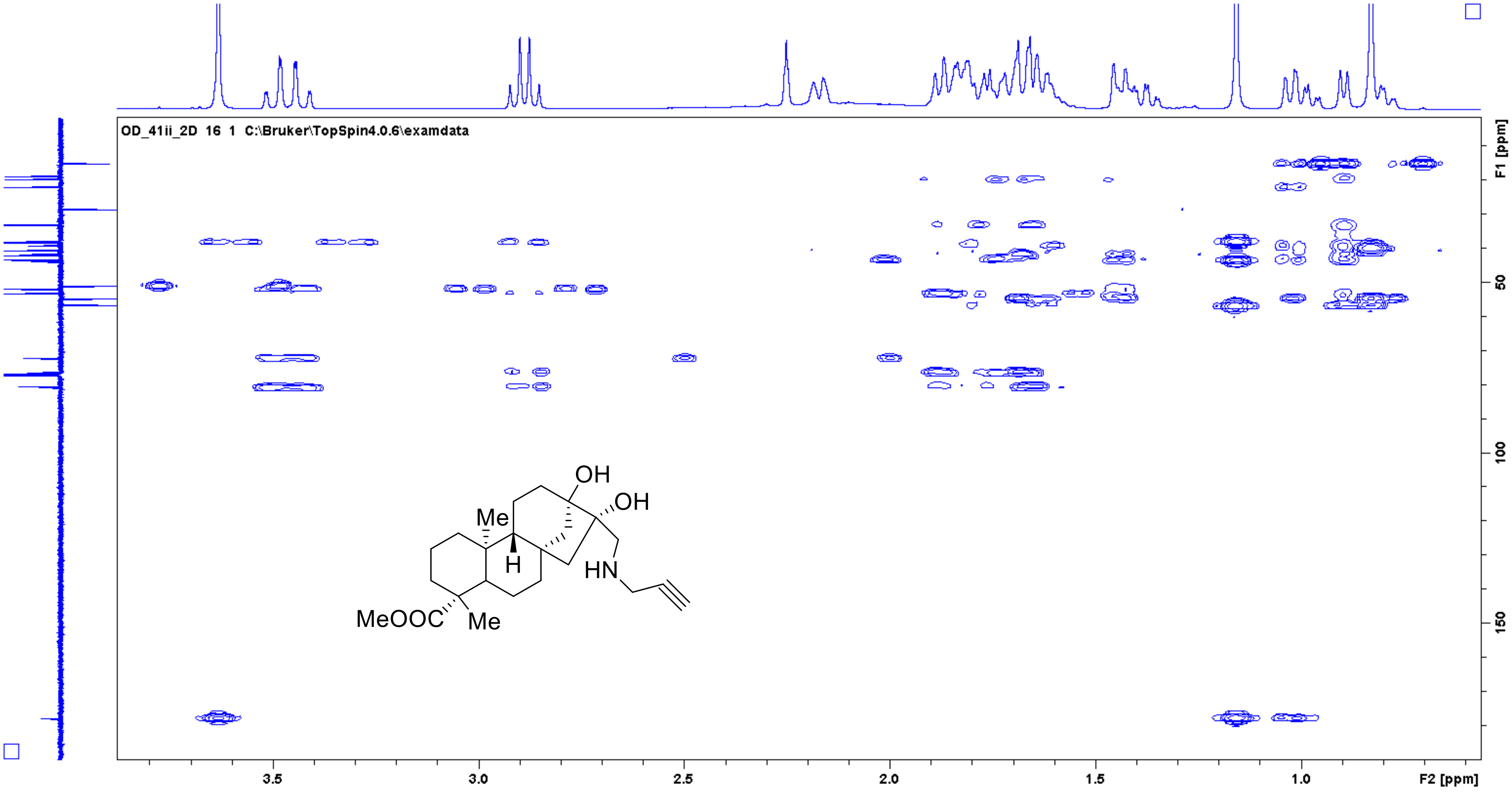

^{1}H-NMR of compound **18**

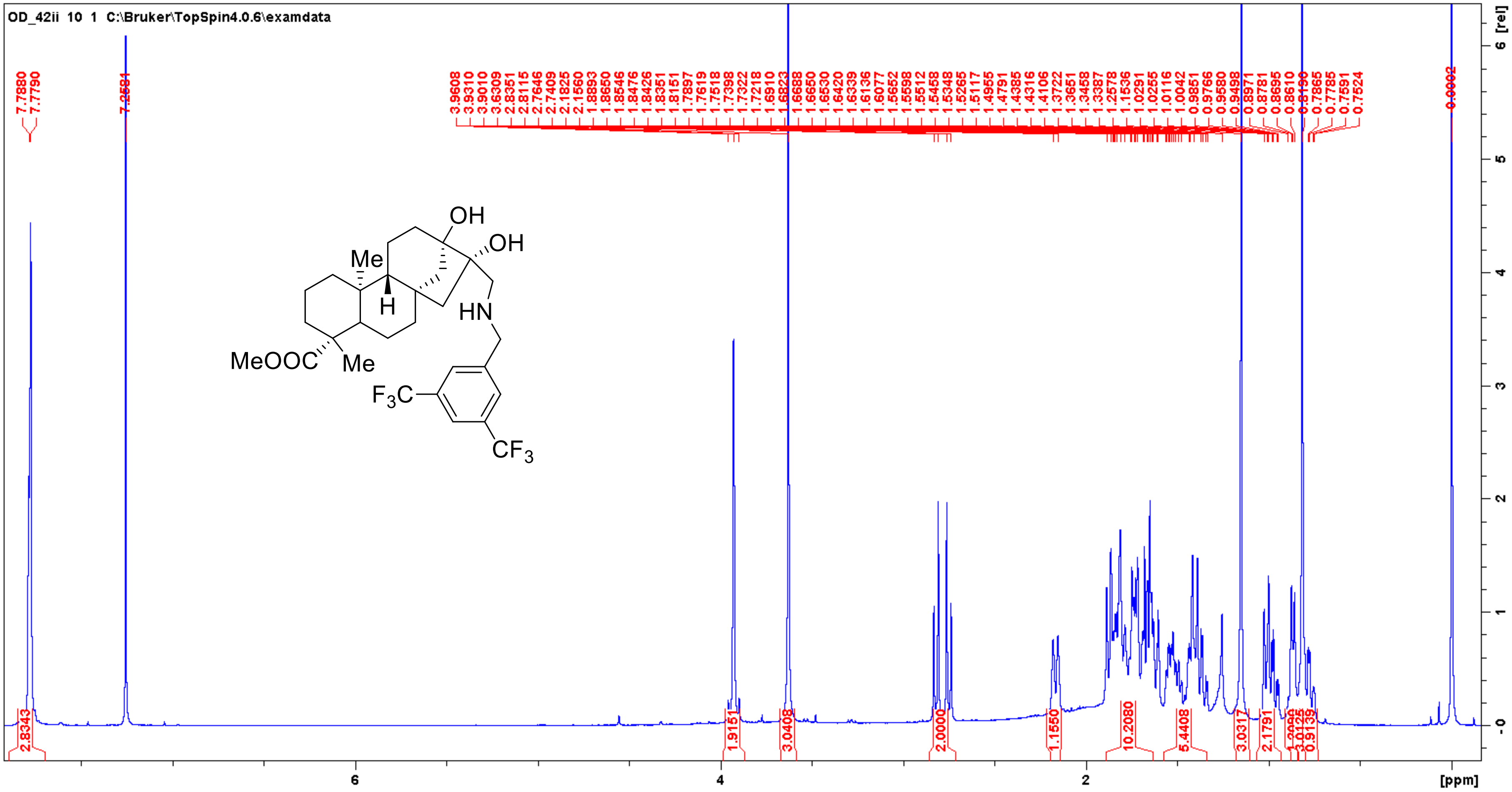

^{13}C-NMR of compound **18**

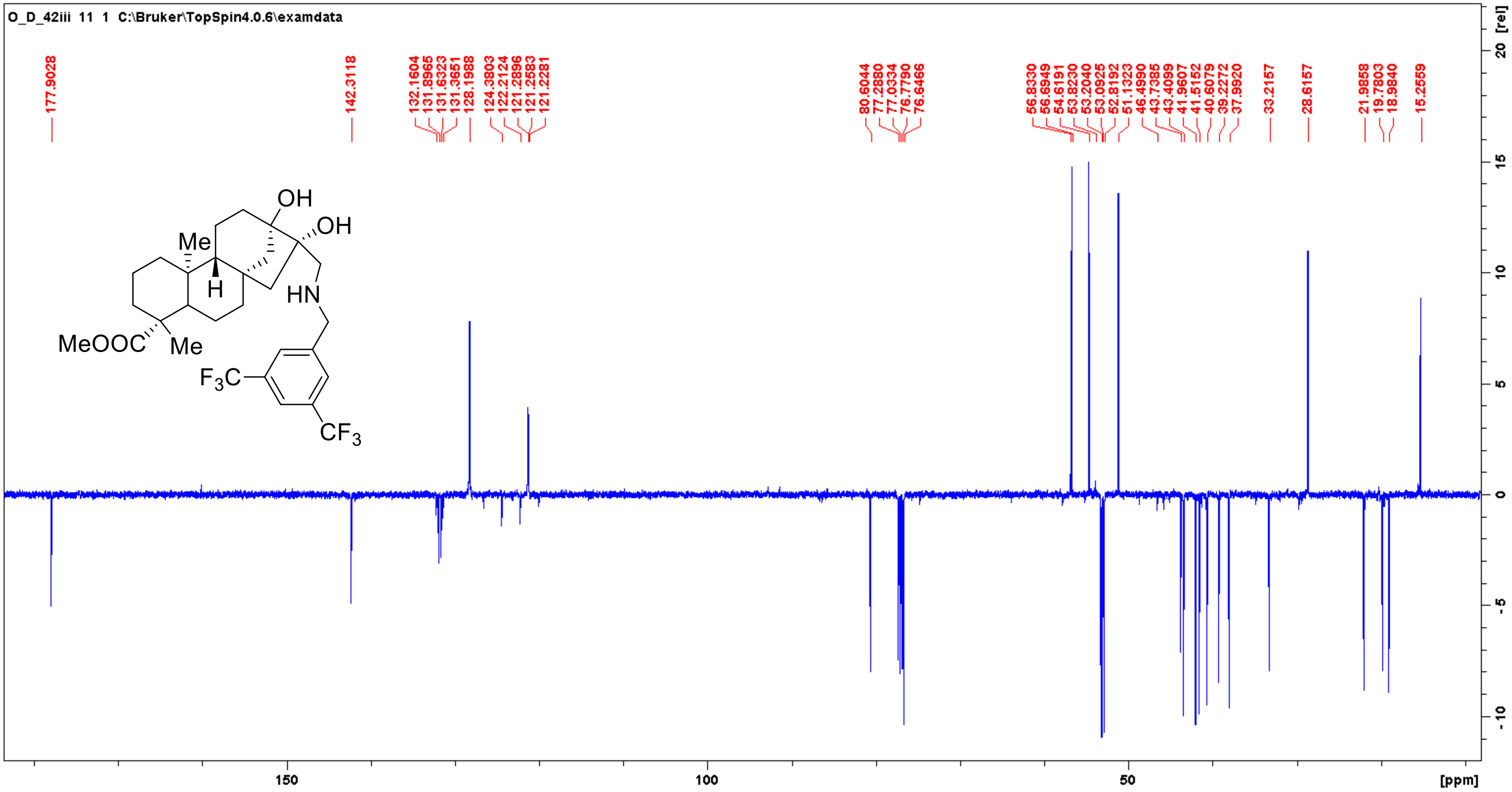

HSQC of compound of **18**

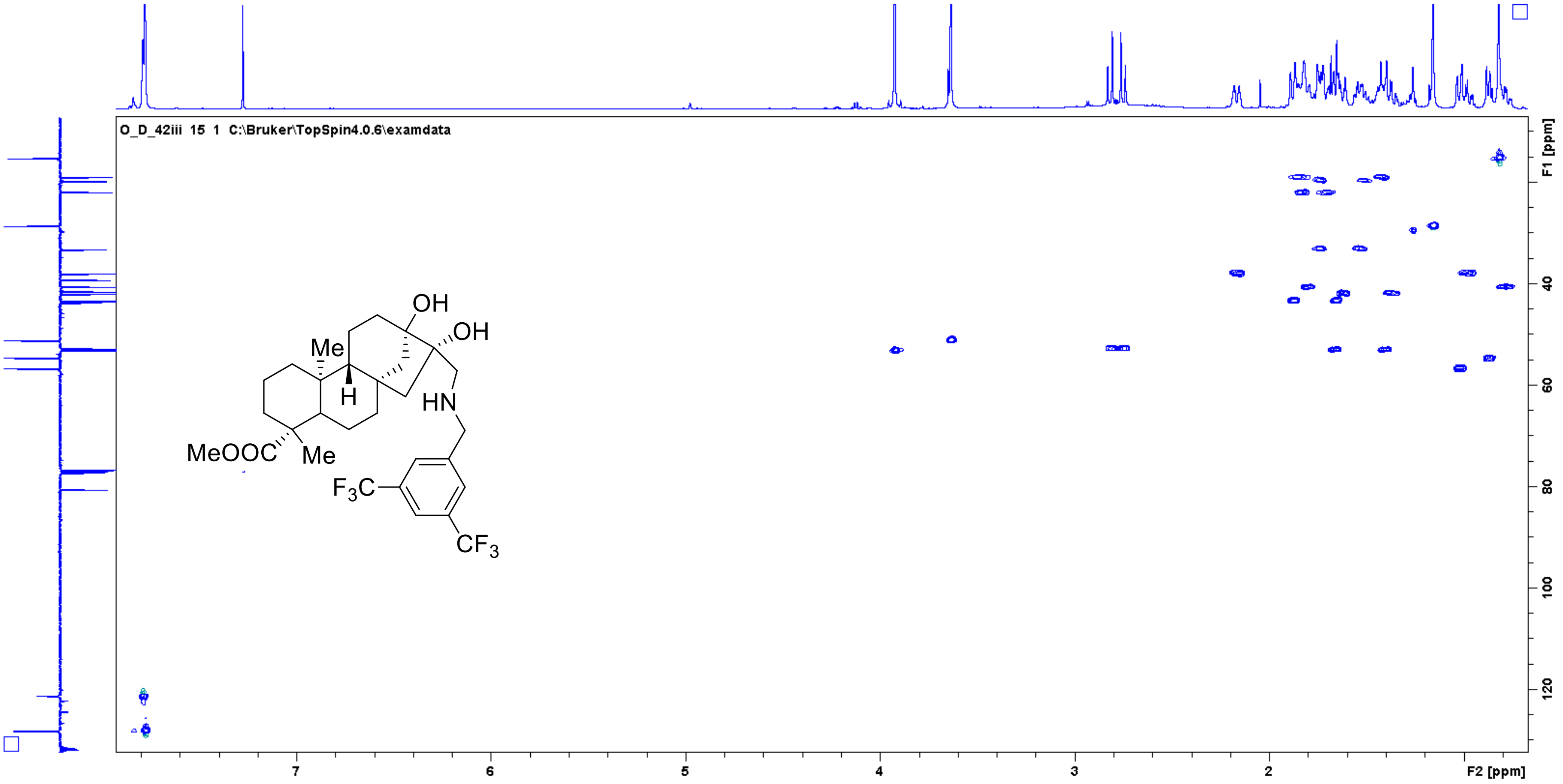

HMBC of compound of **18**

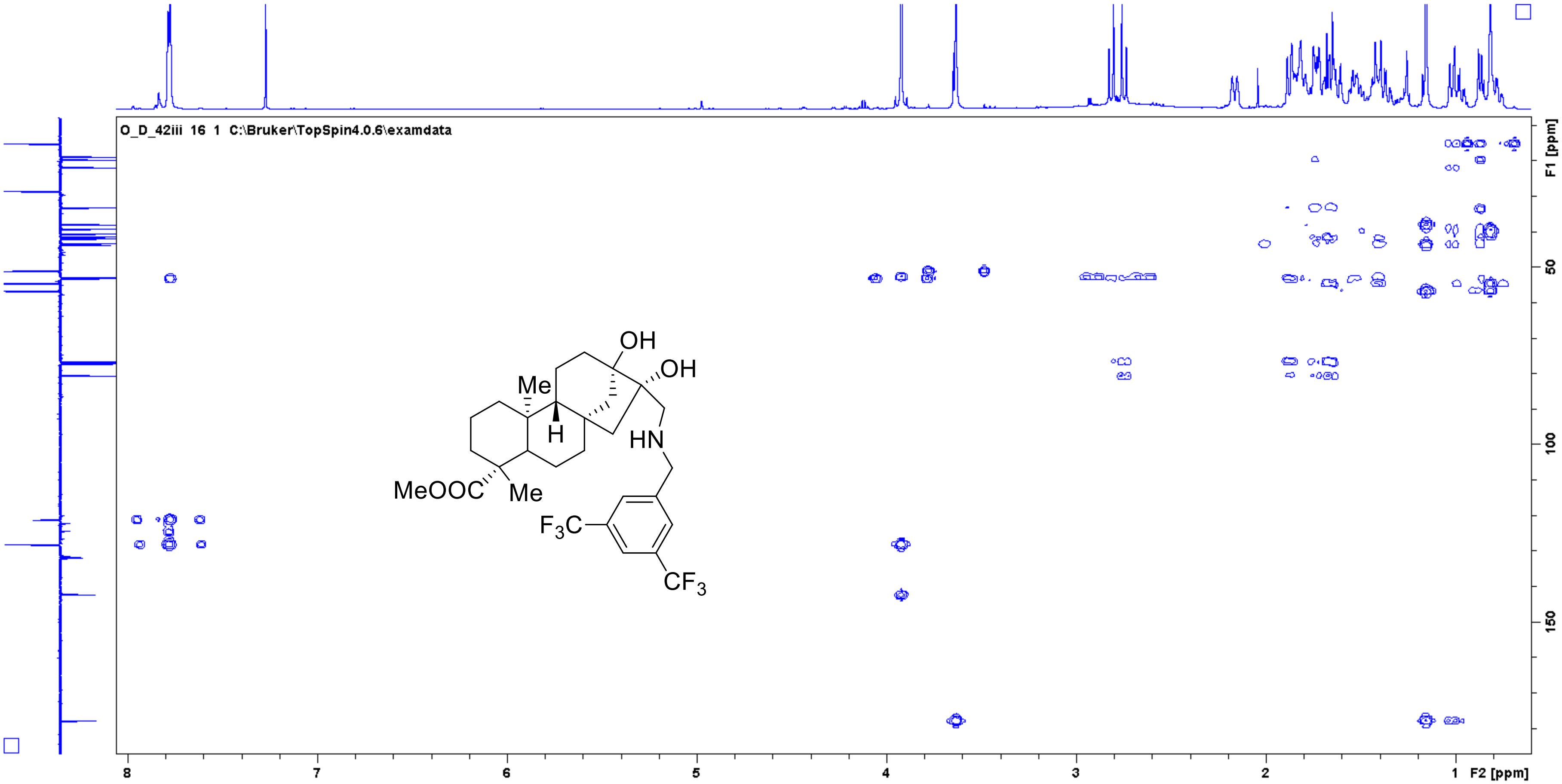

^{1}H-NMR of compound of **19**

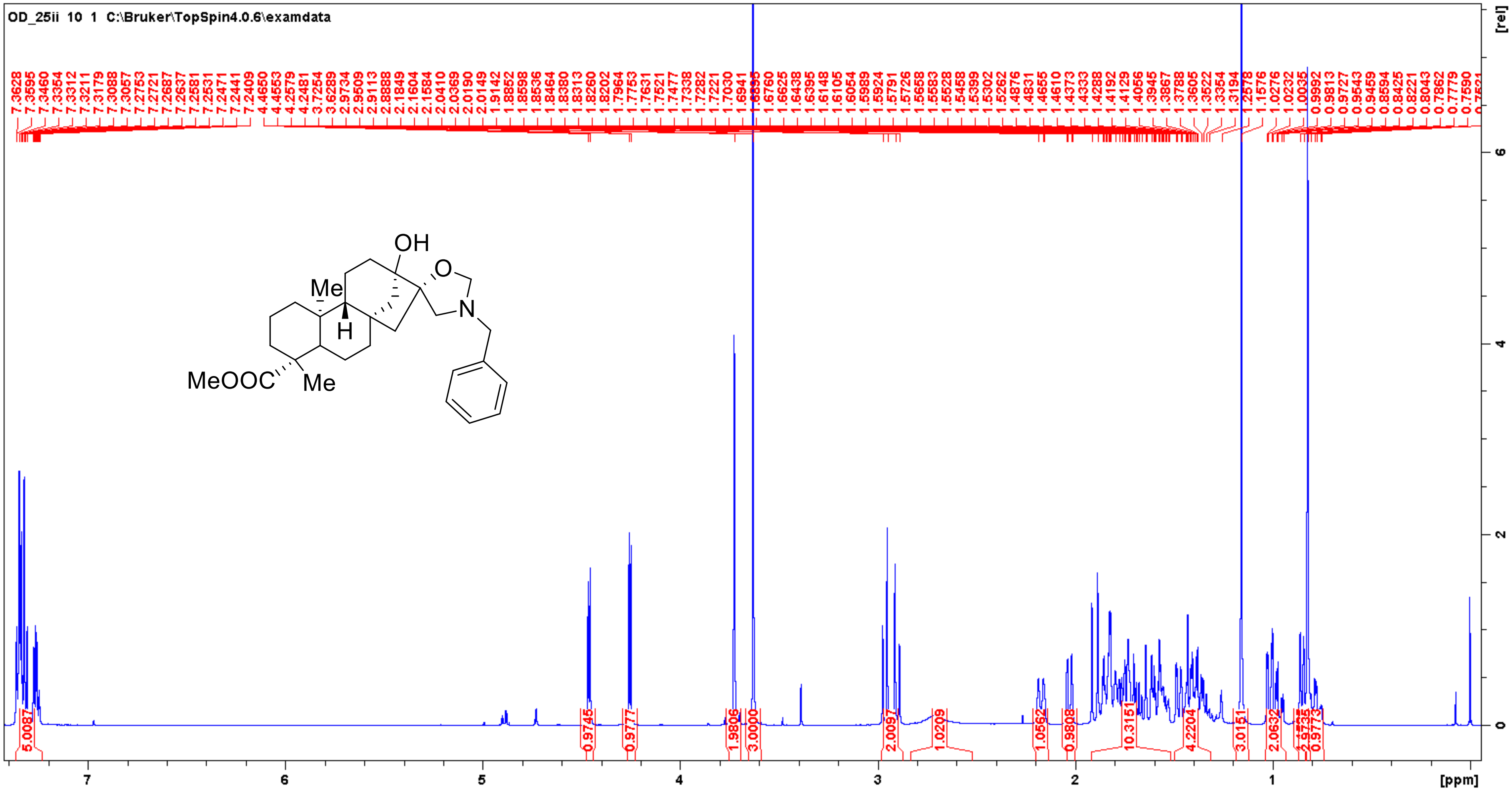

^{13}C-NMR of compound **19**

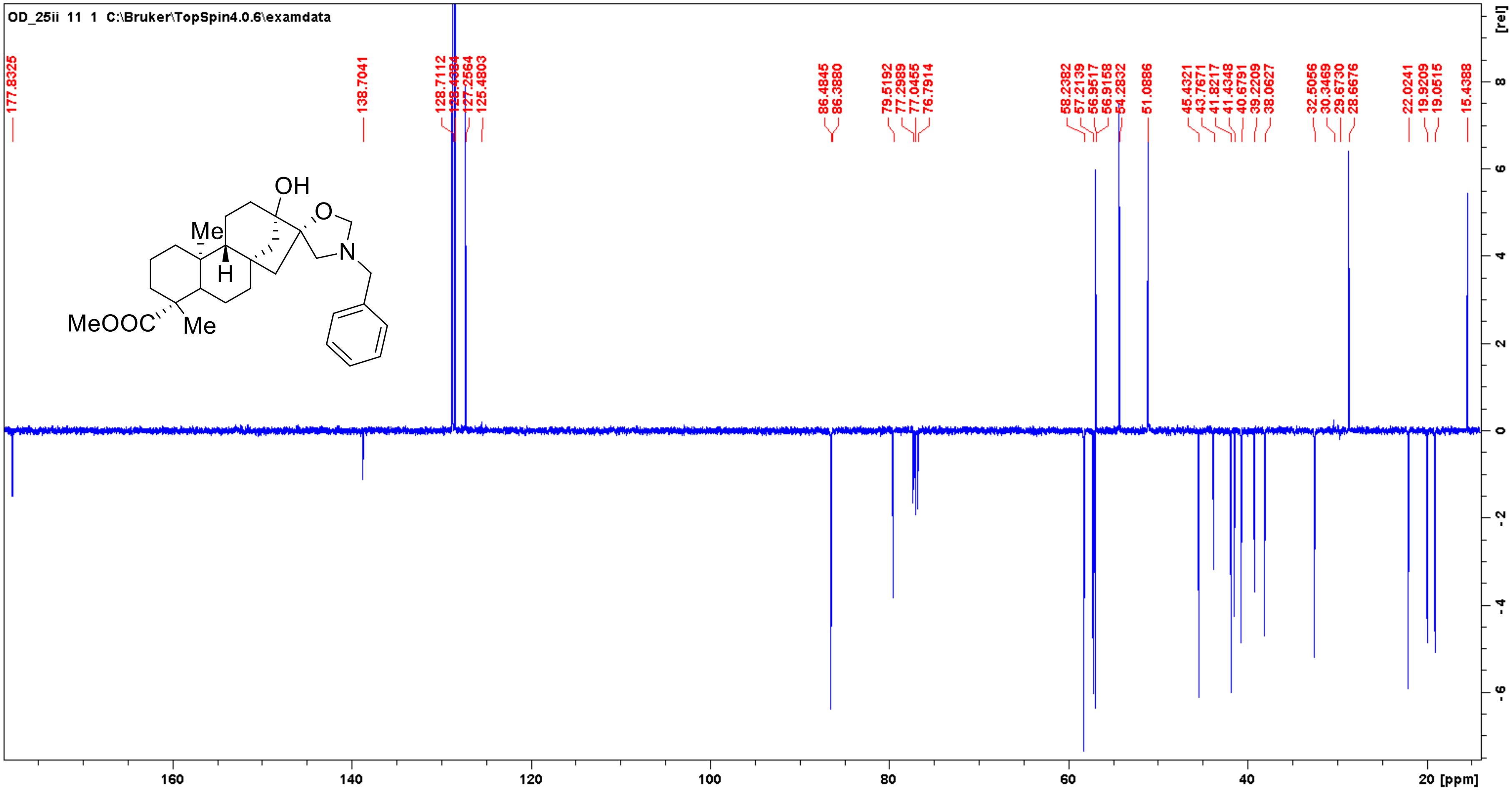

COSY of compound **19**

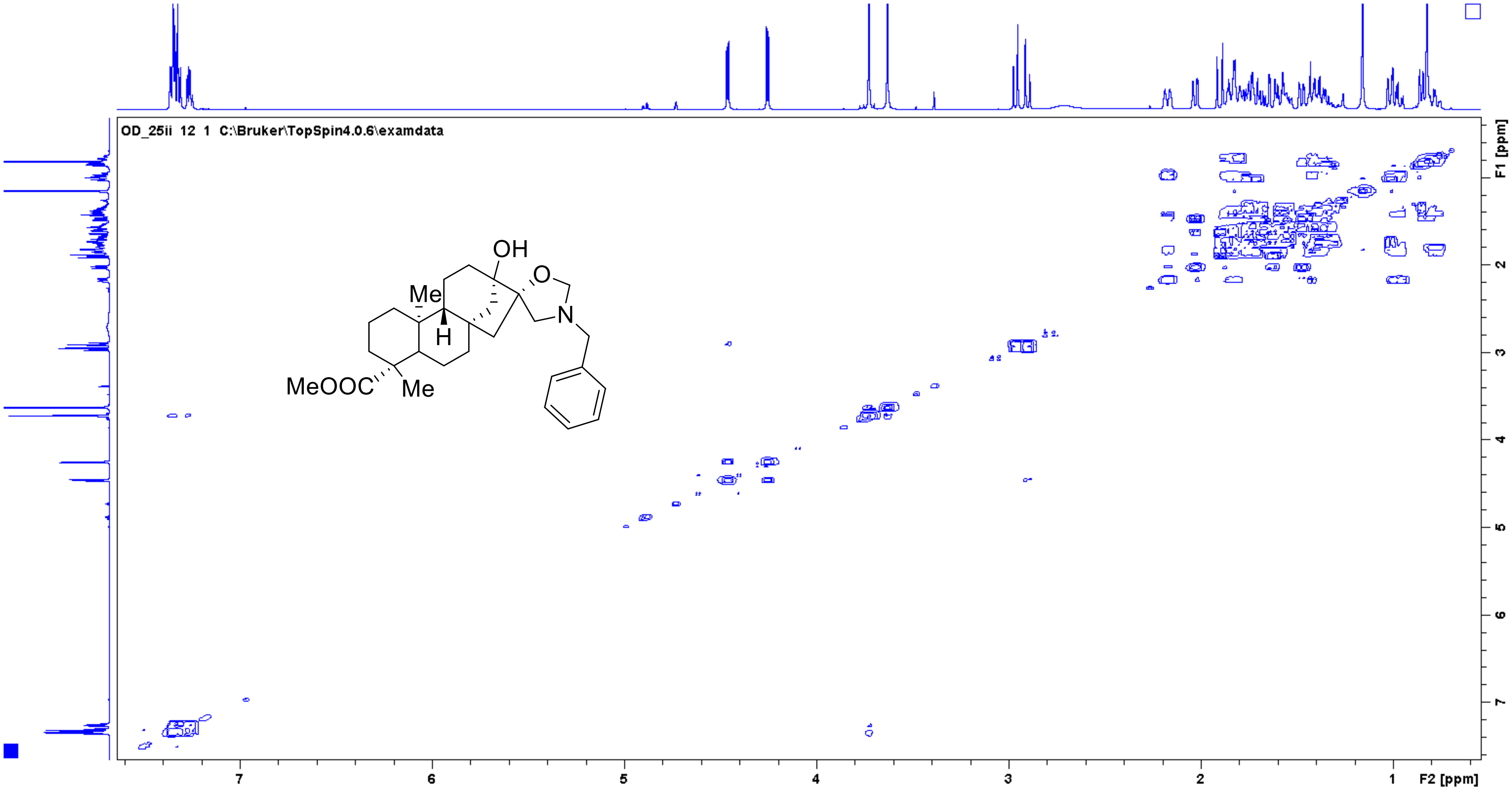

NOESY of compound **19**

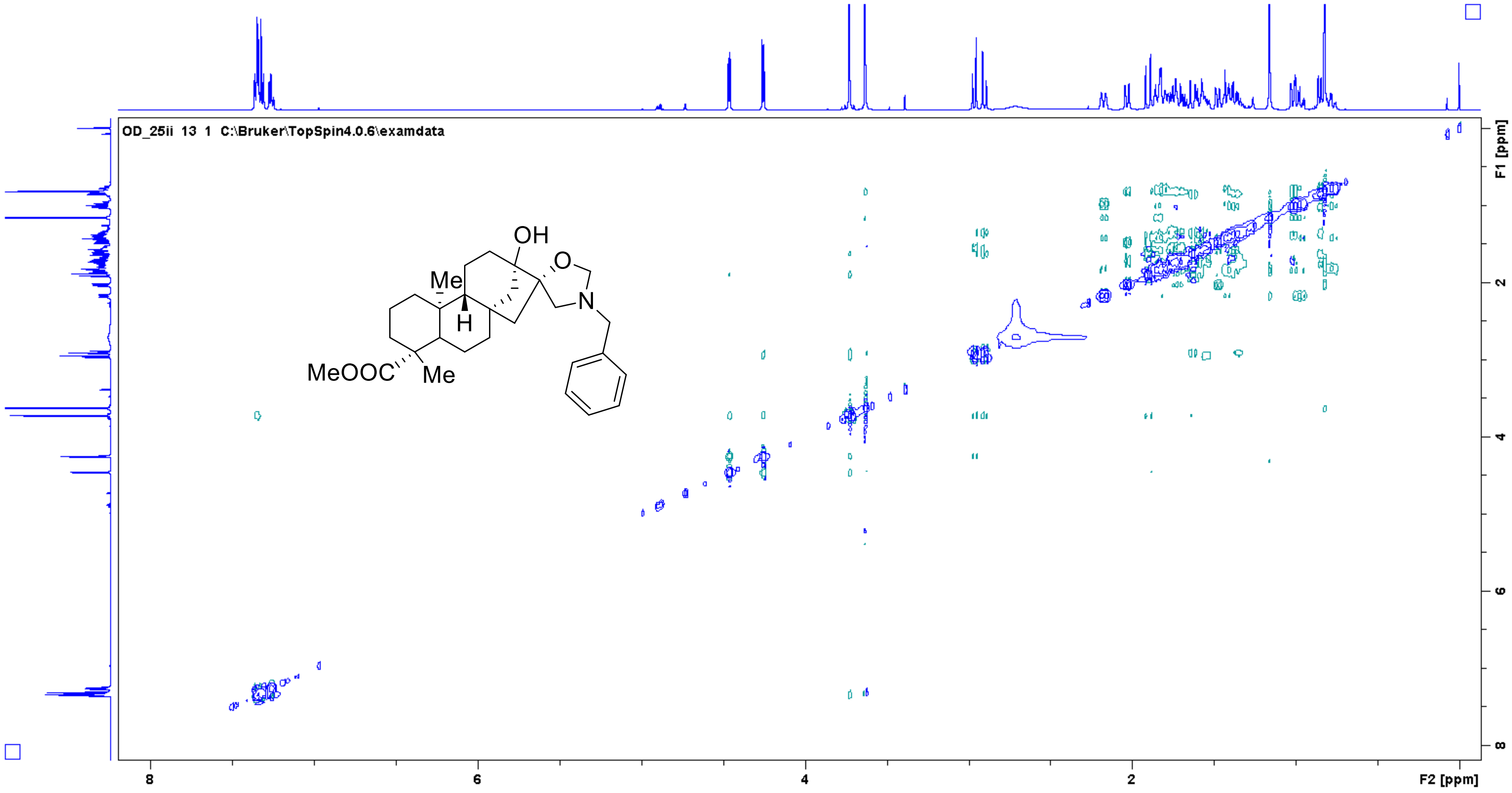

HSQC of compound **19**

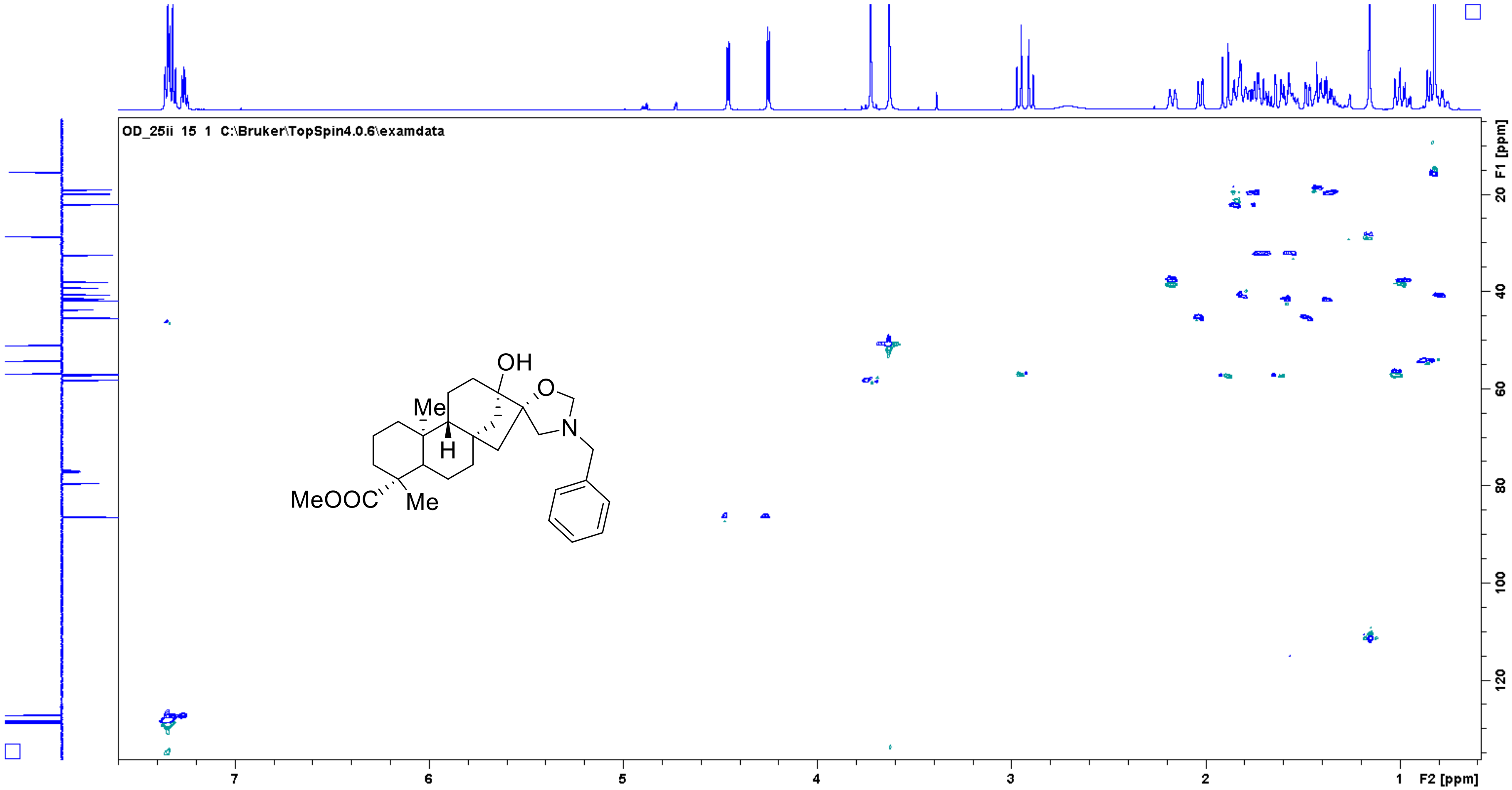

HMBC of compound **19**

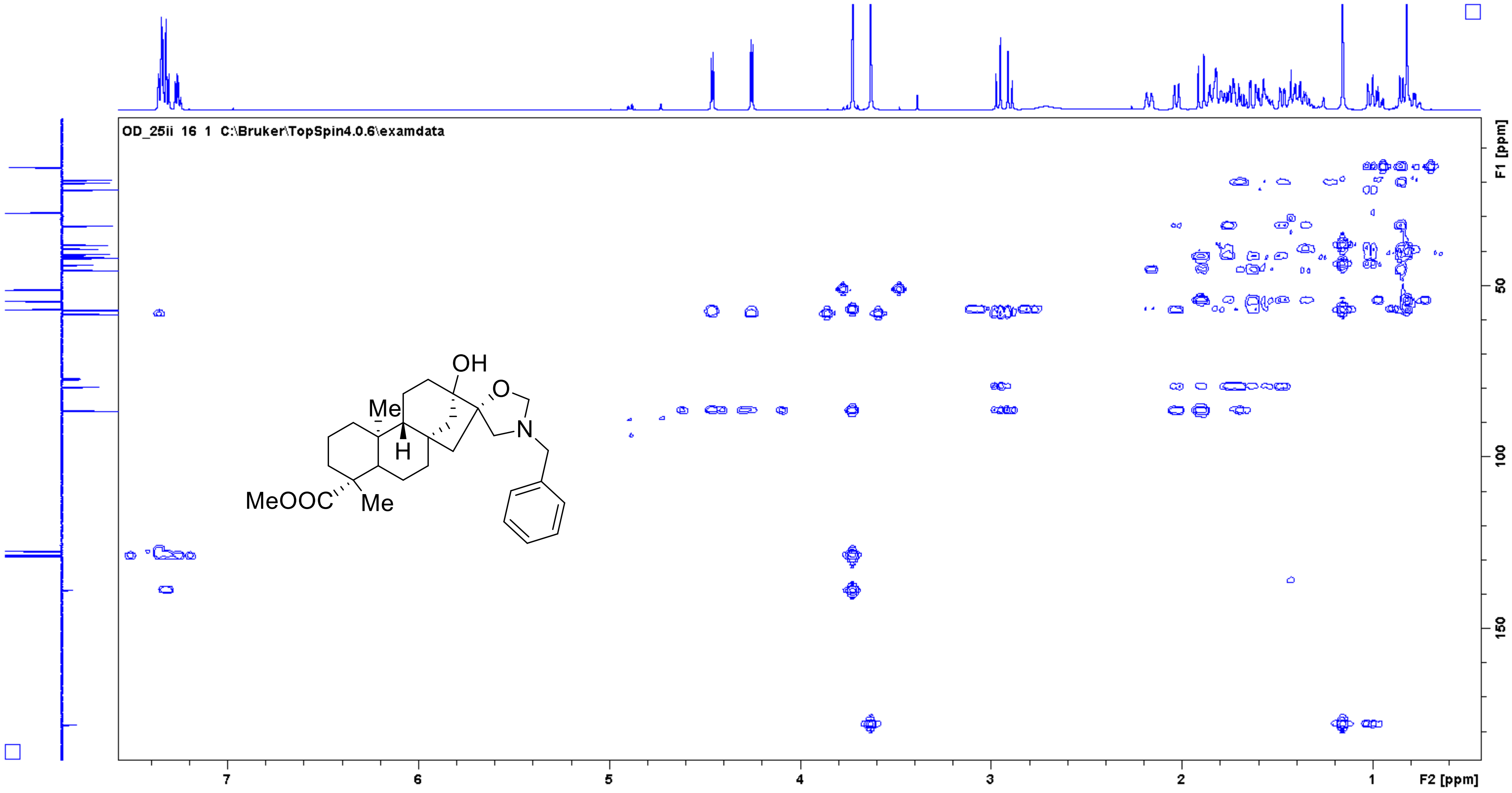

^{1}H-NMR of compound **20**

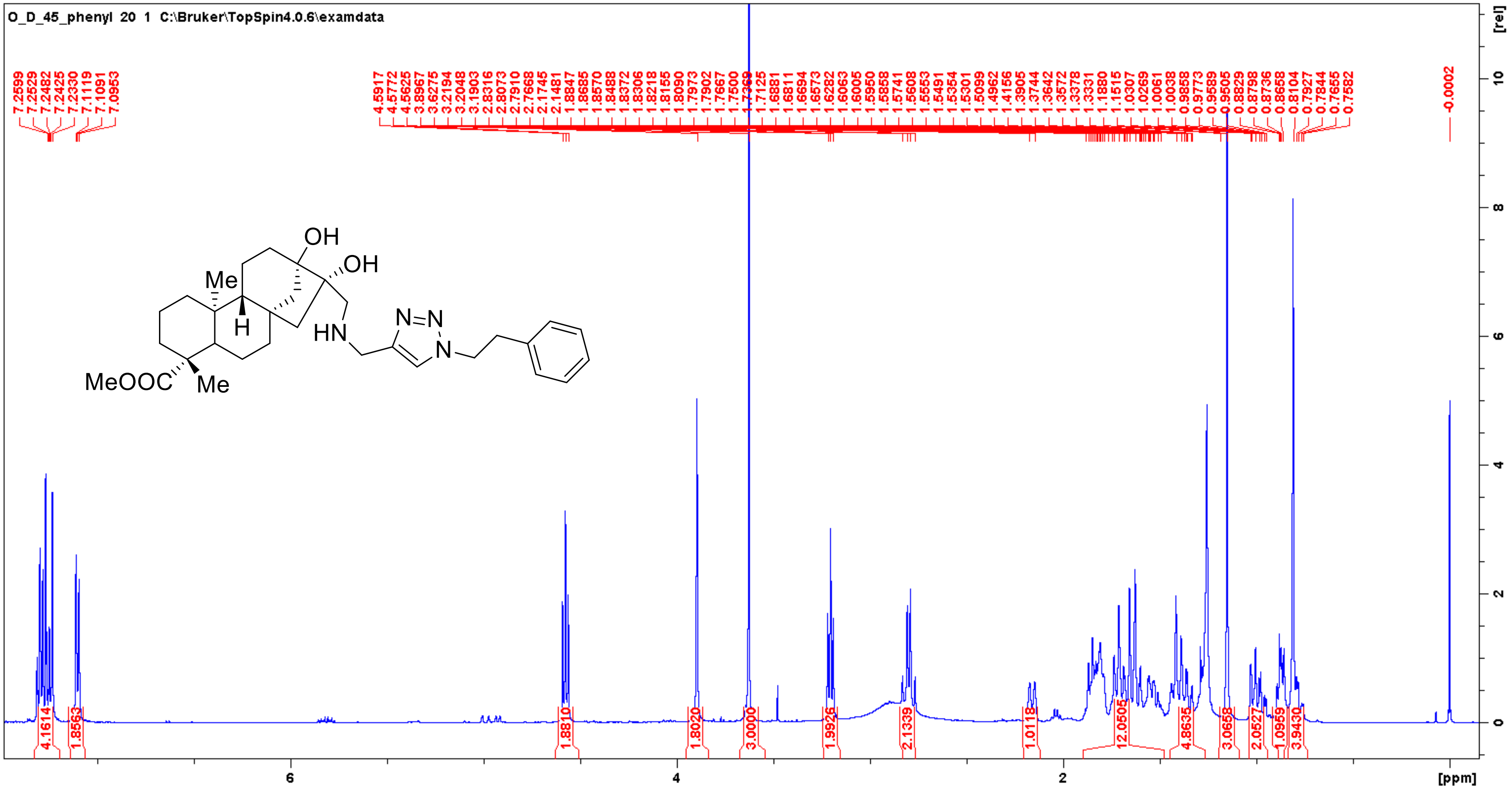

^{13}C-NMR of compound **20**

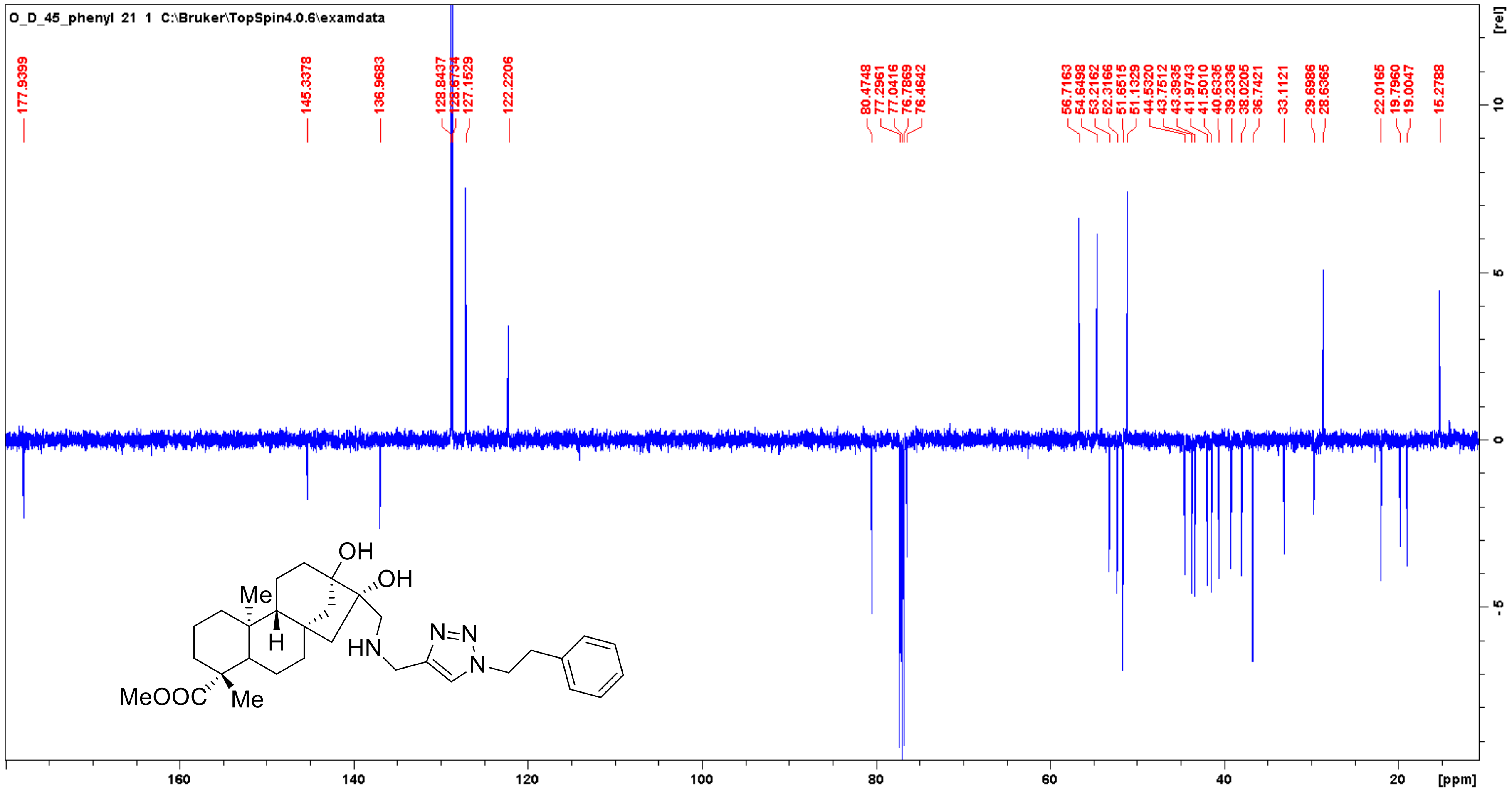

COSY of compound **20**

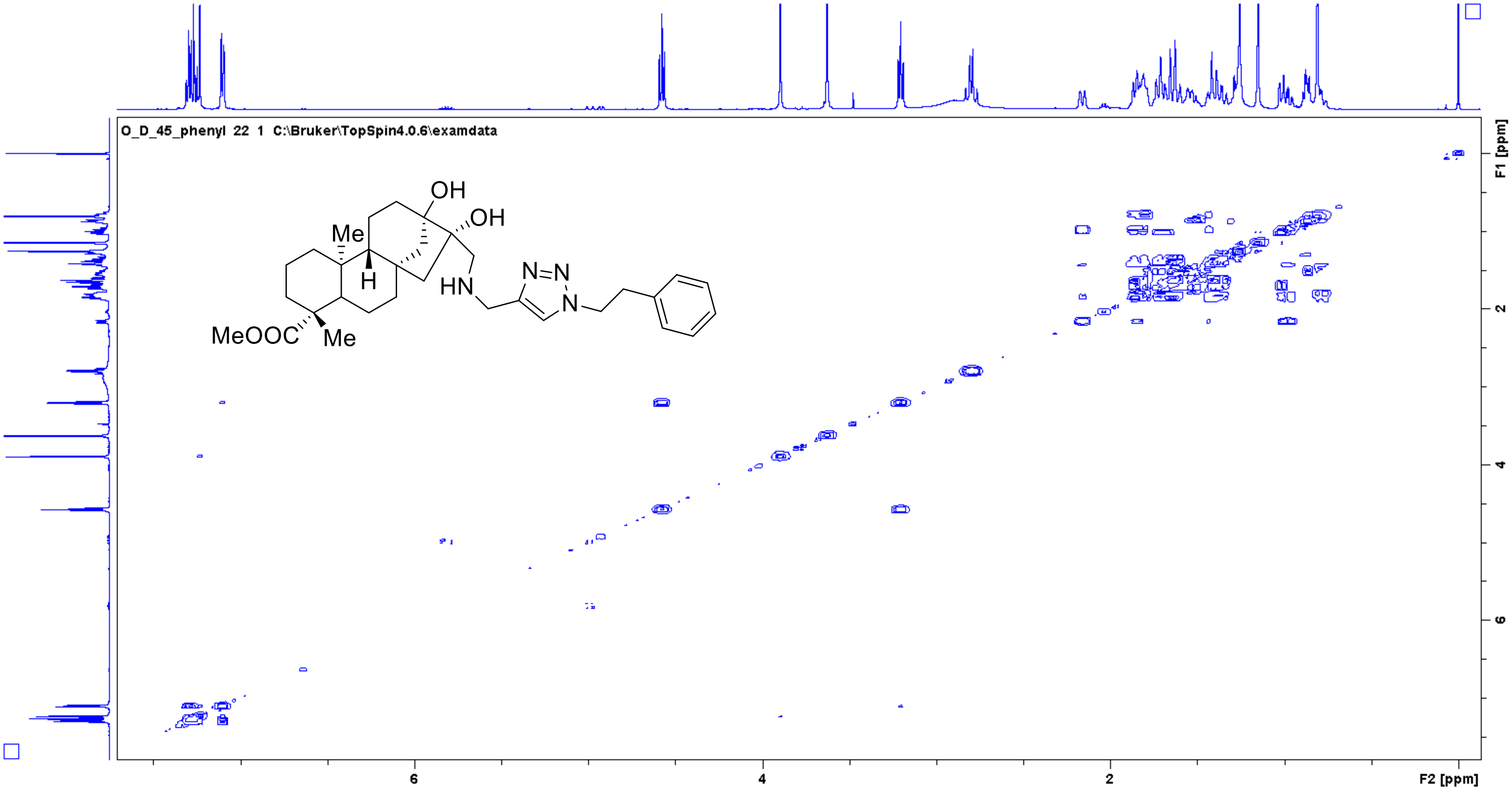

HSQC of compound **20**

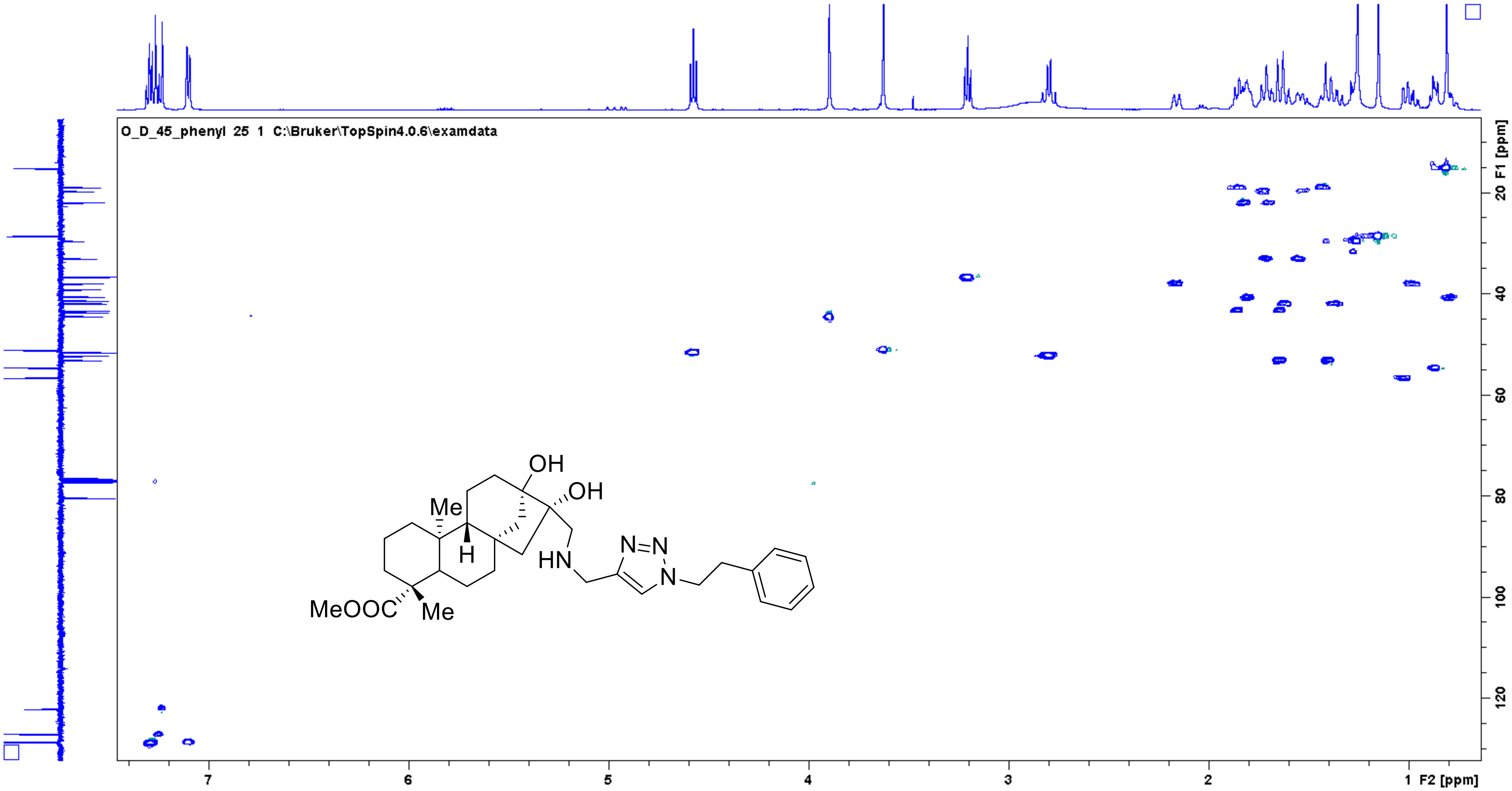

HMBC of compound **20**

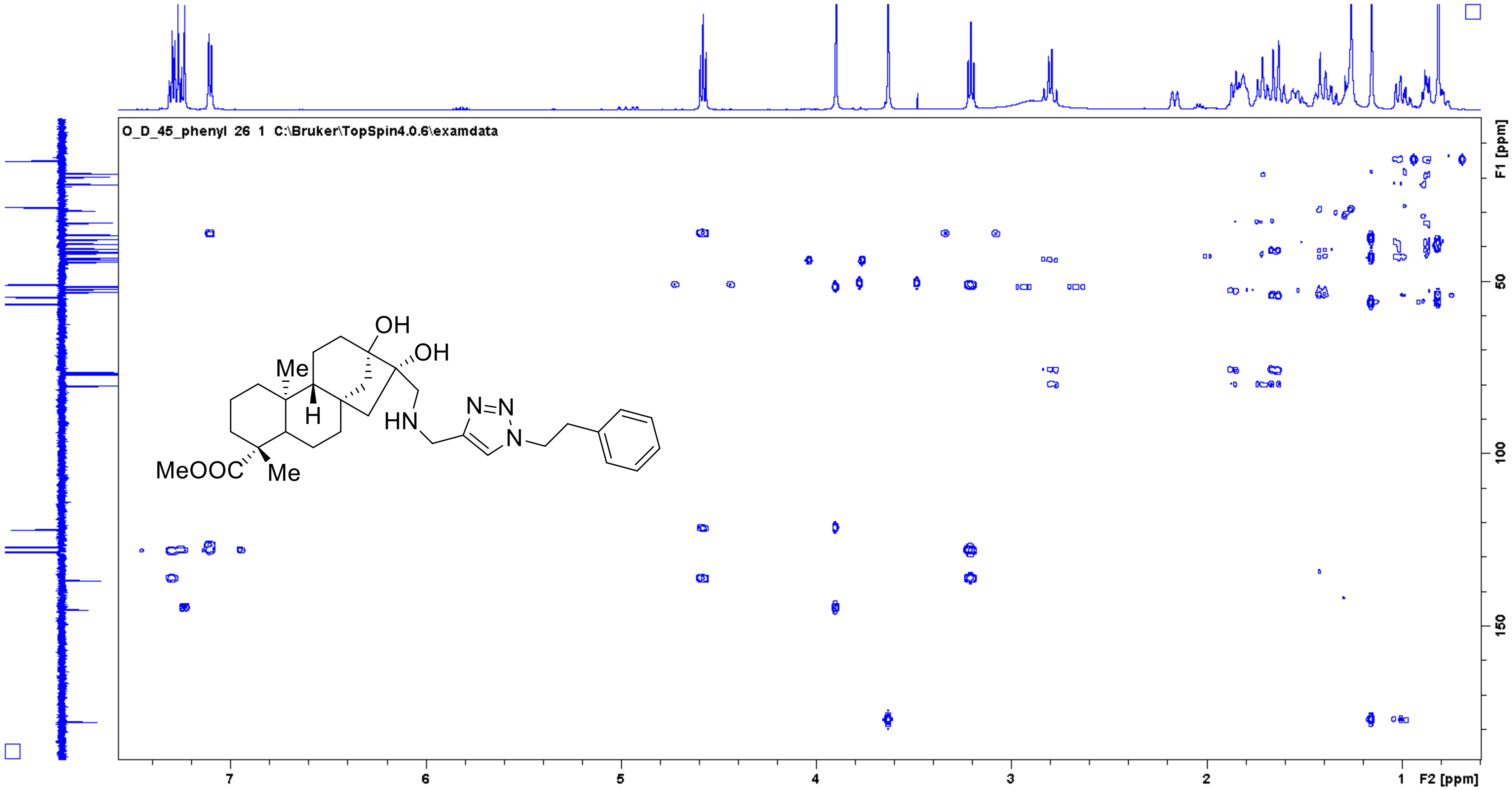

^{1}H-NMR of compound **21**

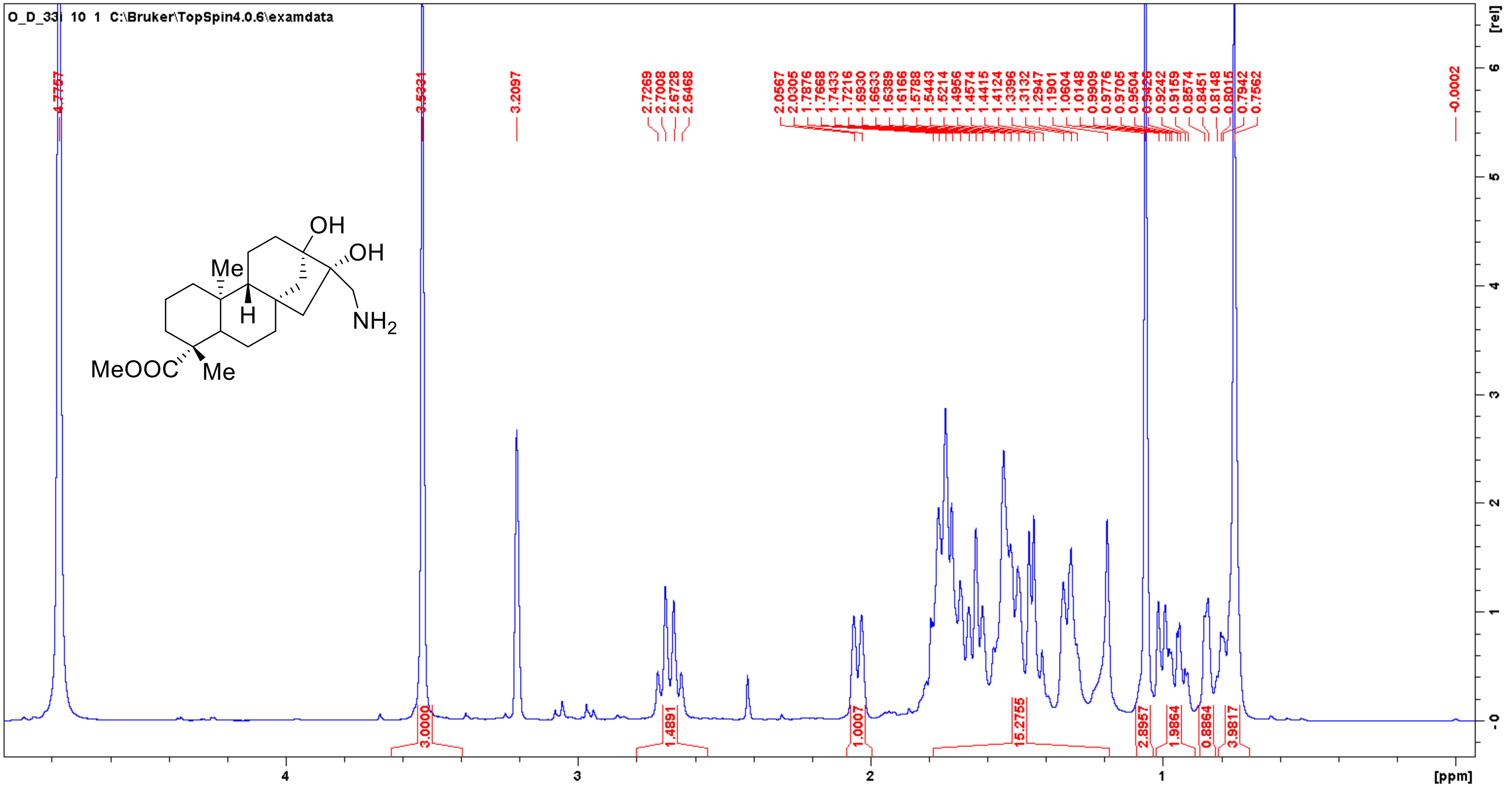

^{13}C-NMR of compound **21**

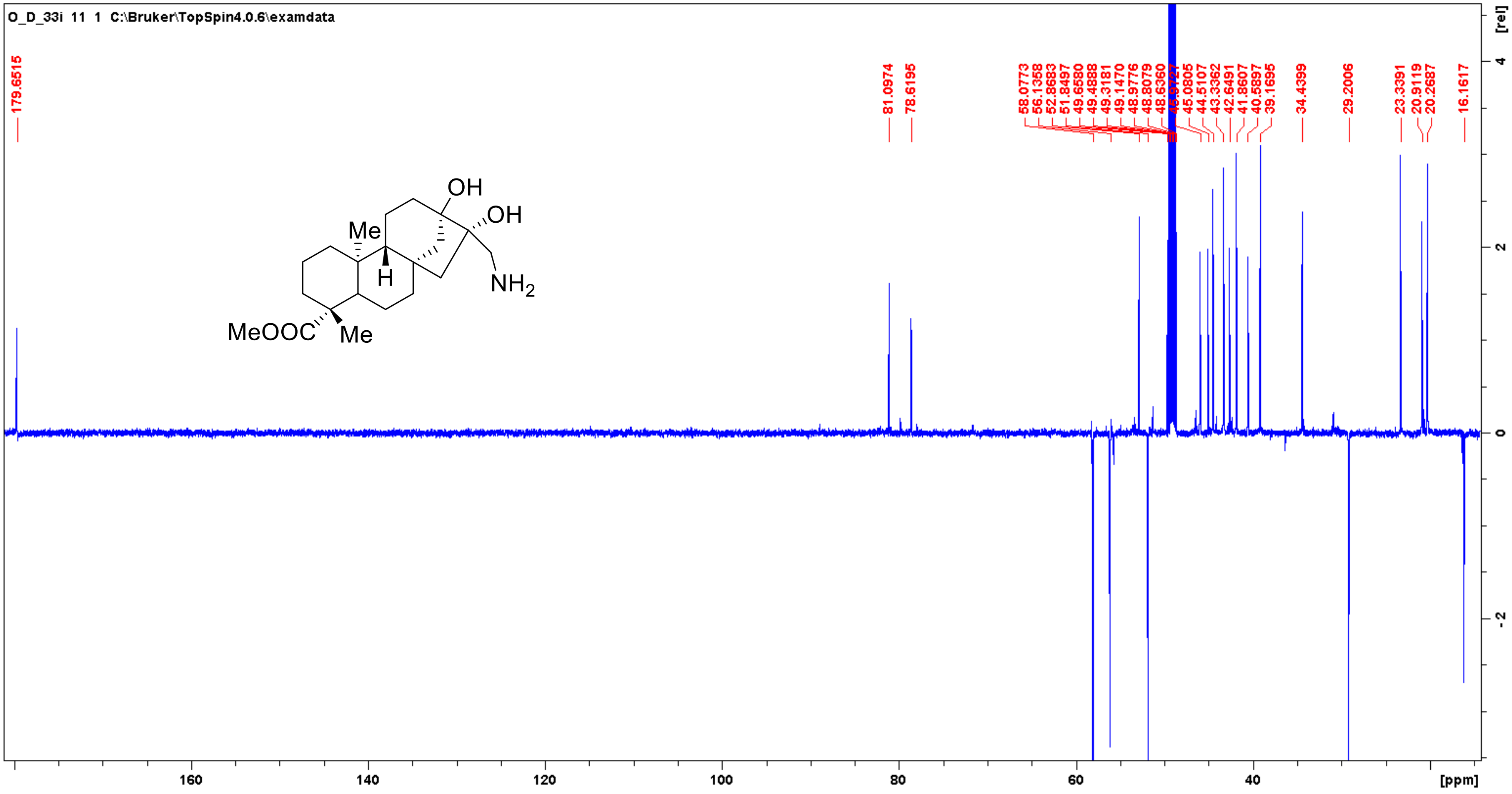

COSY of compound **21**

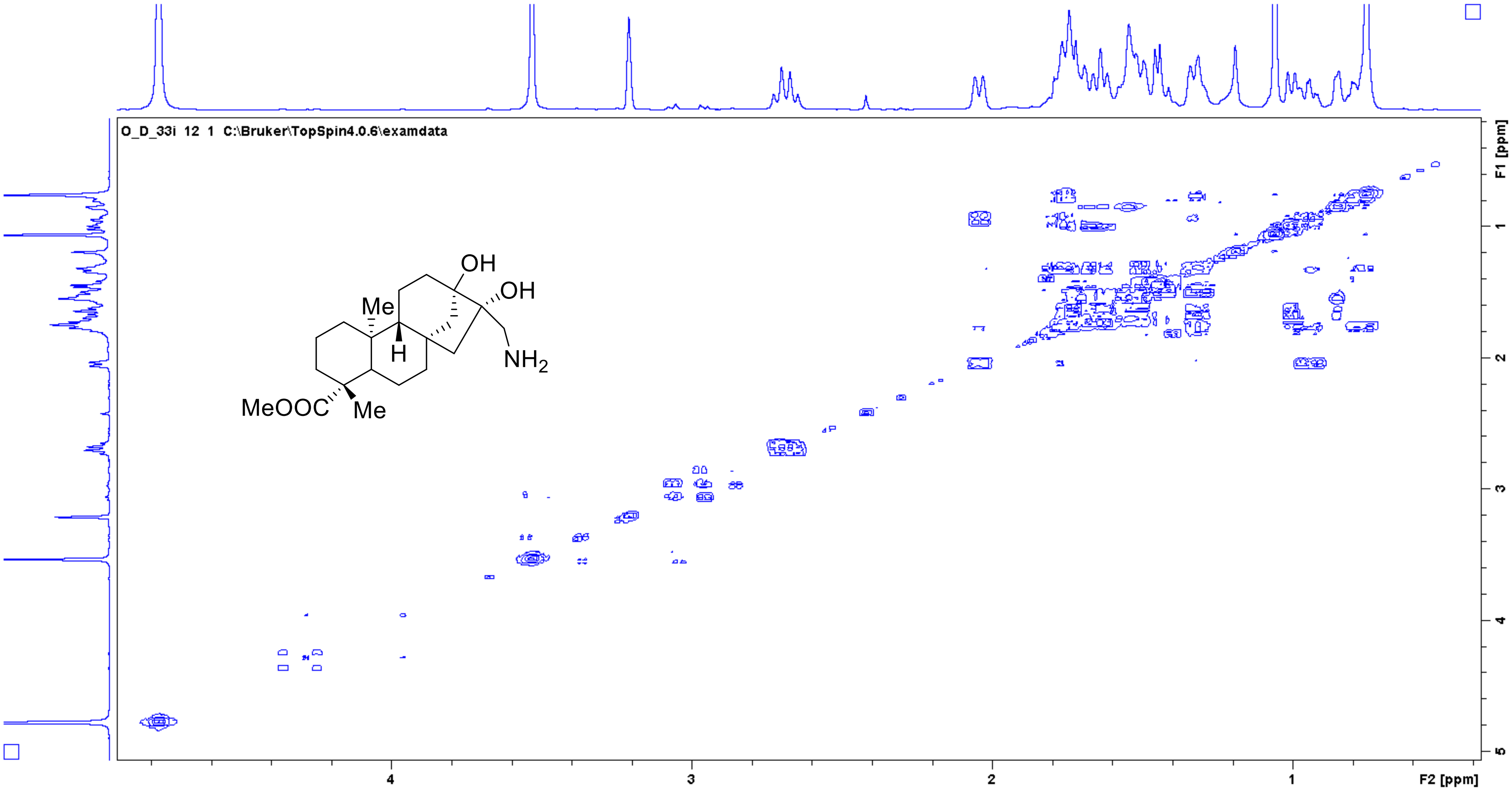

NOESY of compound **21**

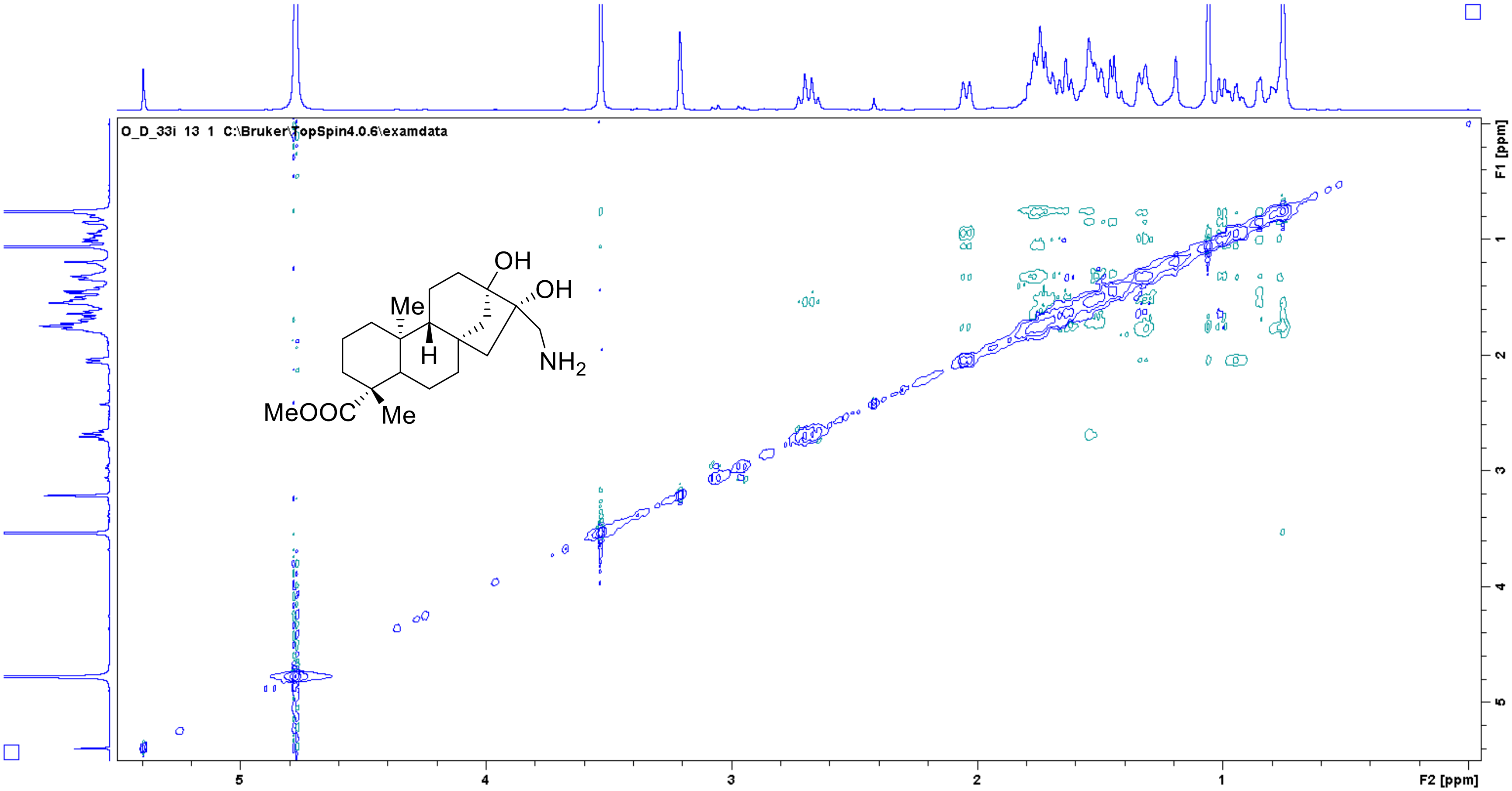

HSQC of compound **21**

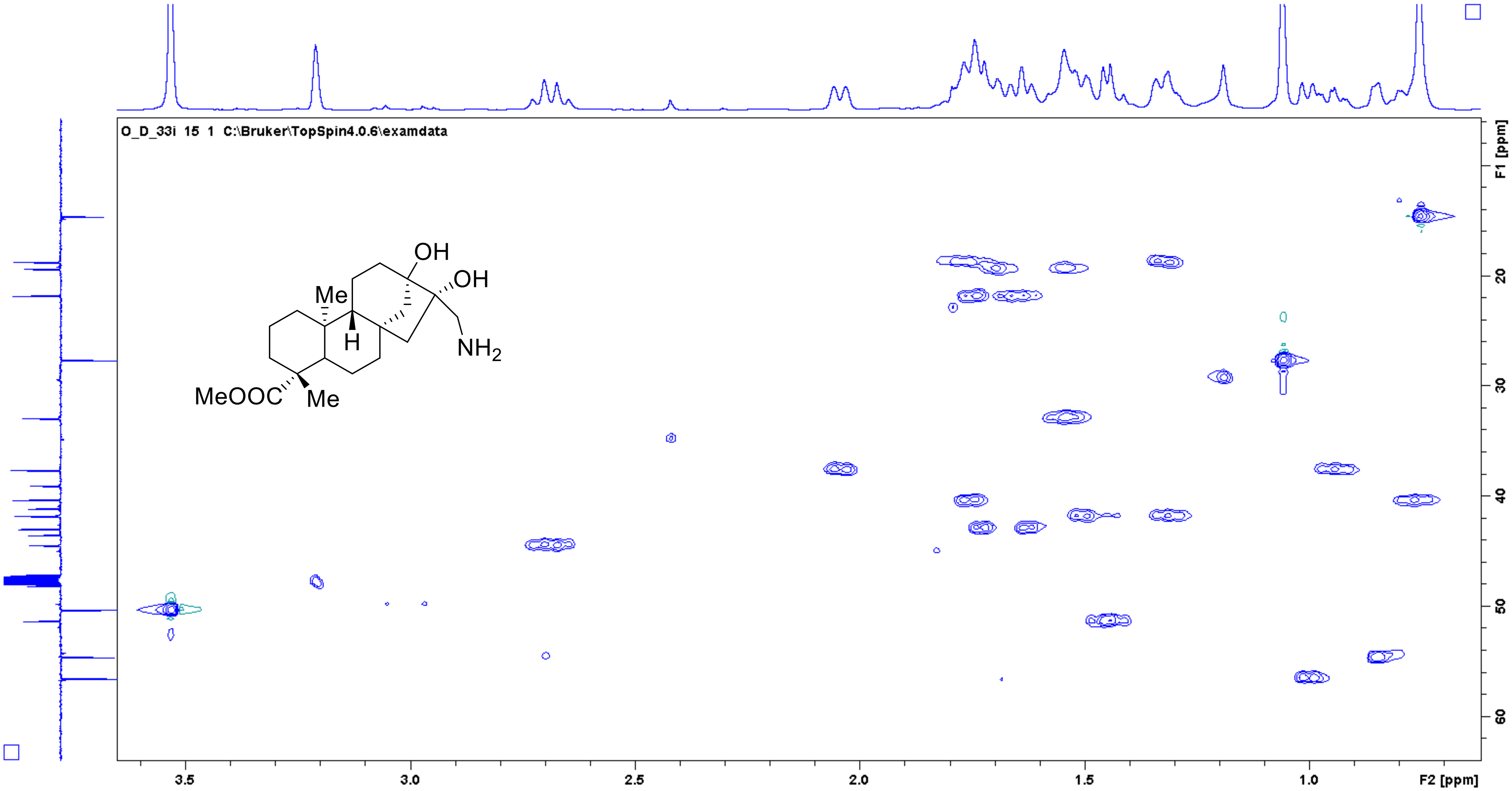

HMBC of compound **21**

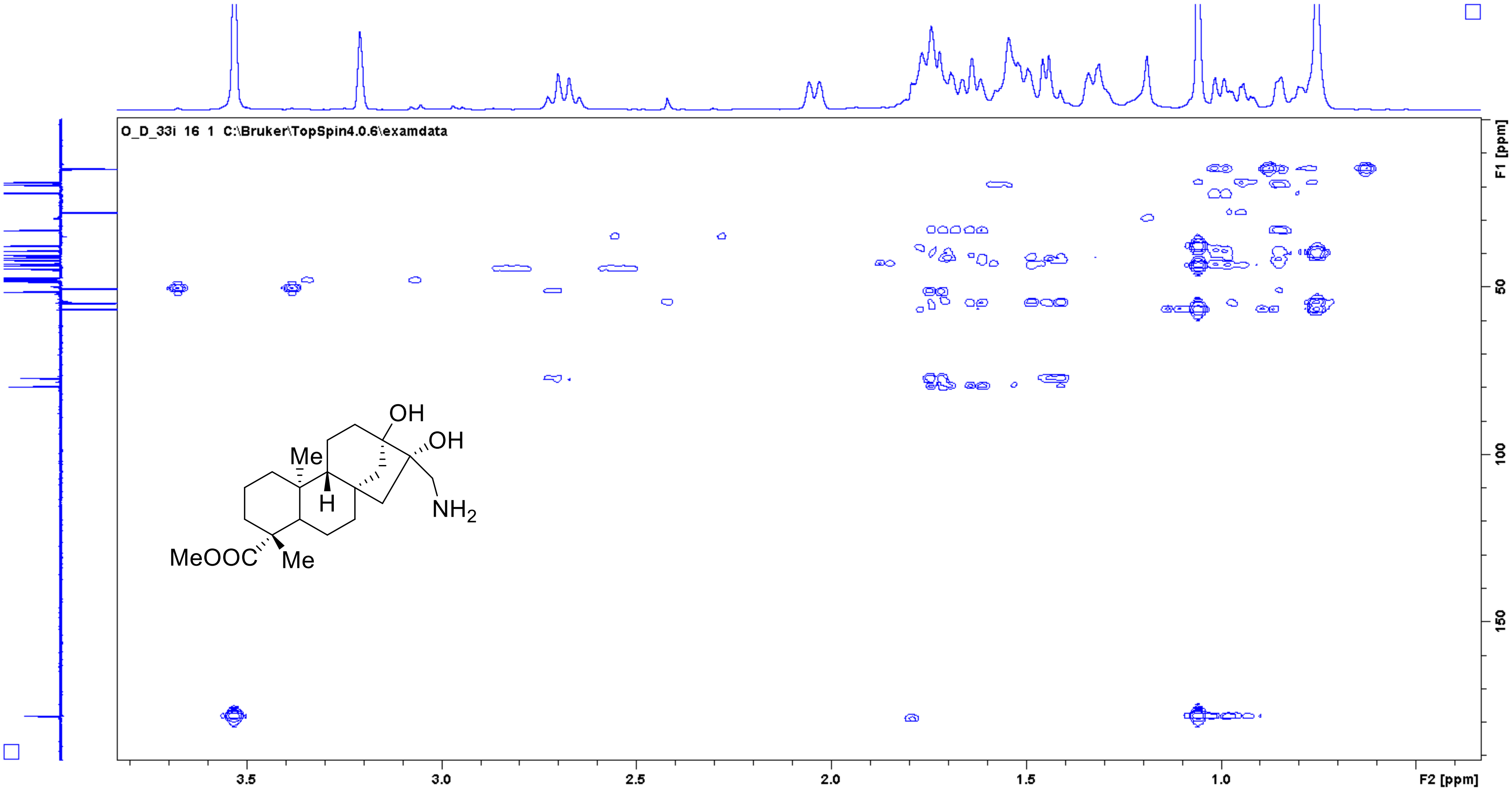

^{1}H-NMR of compound **22**

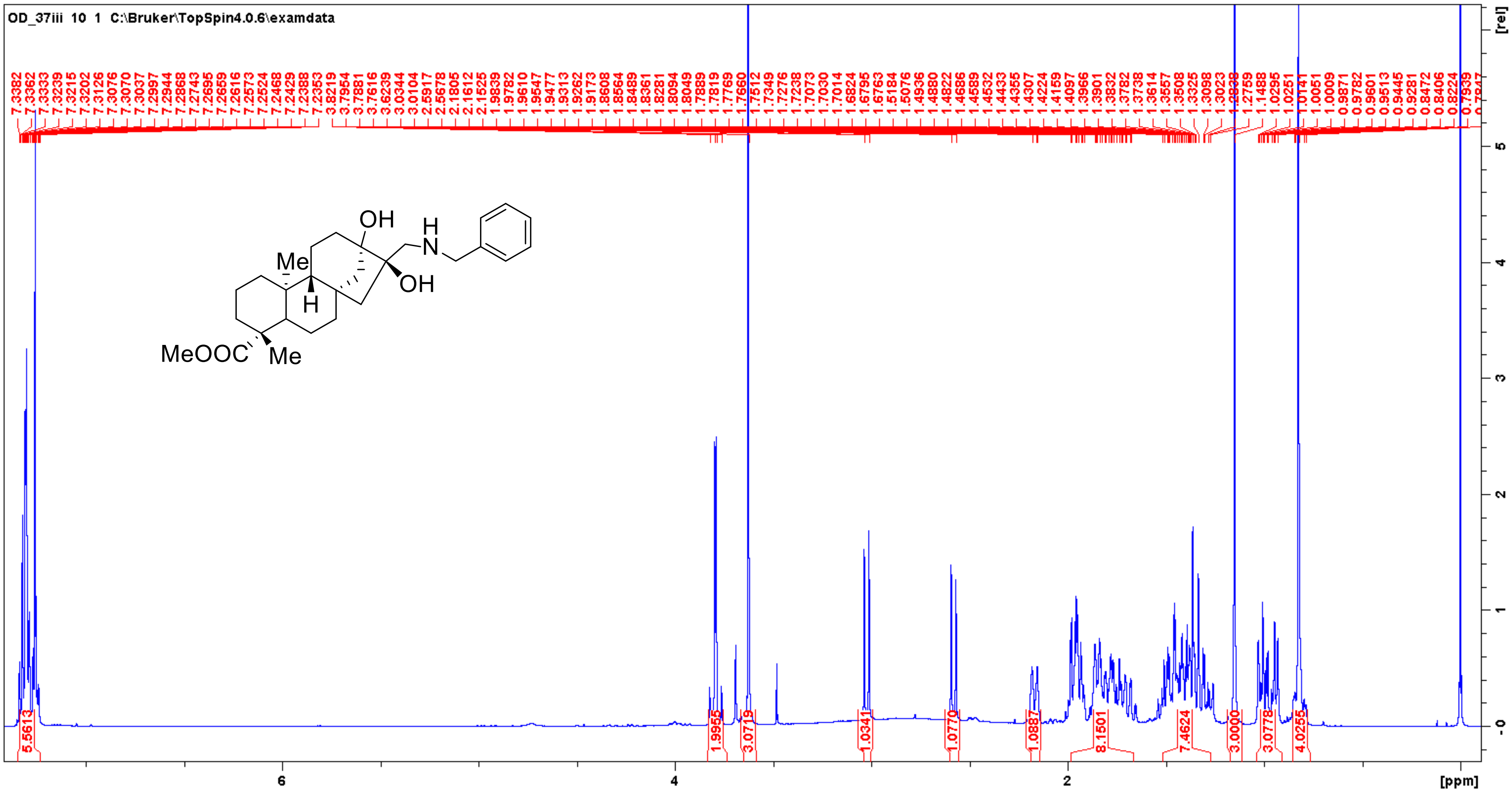

^{13}C-NMR of compound **22**

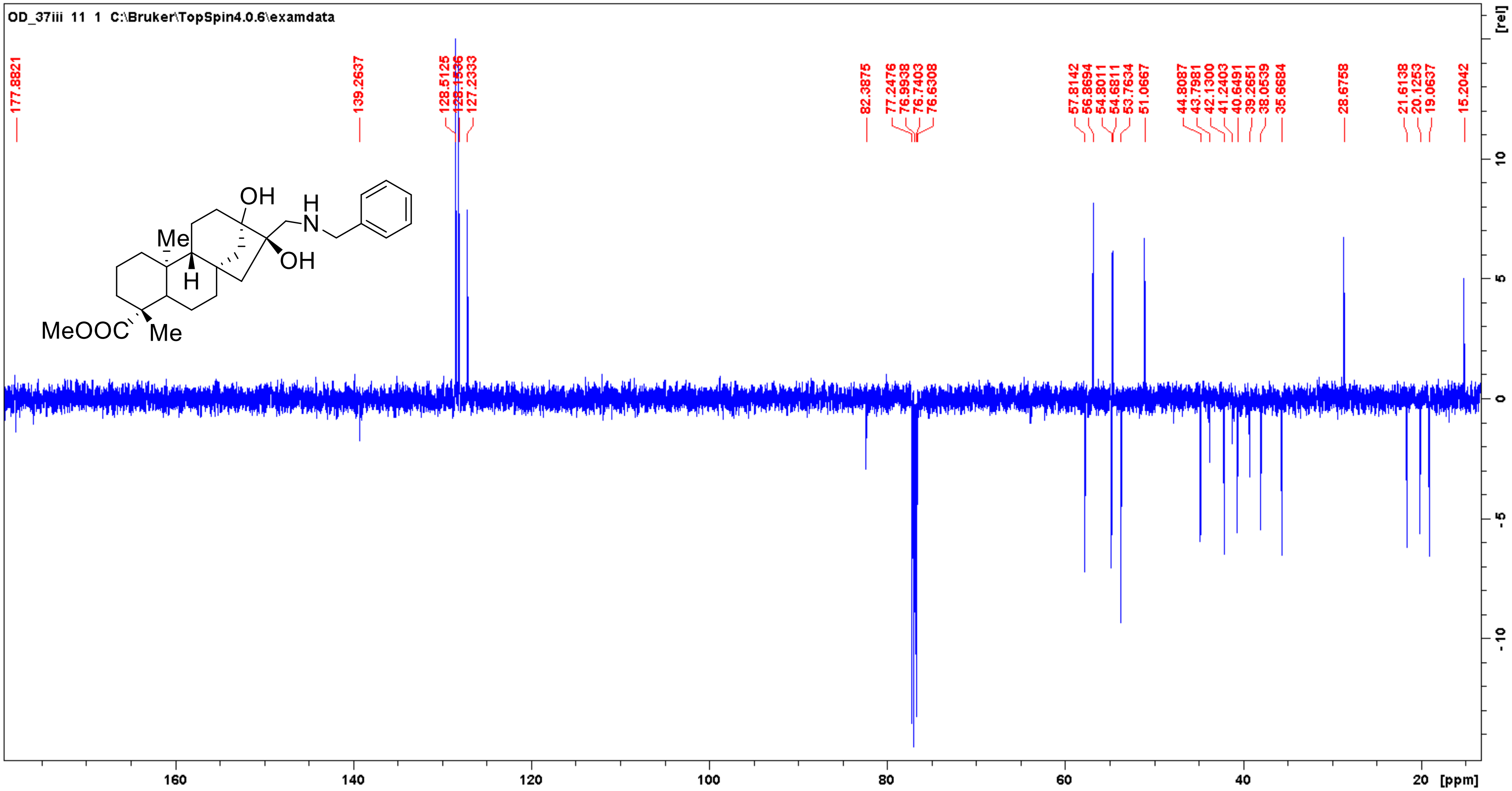

COSY of compound **22**

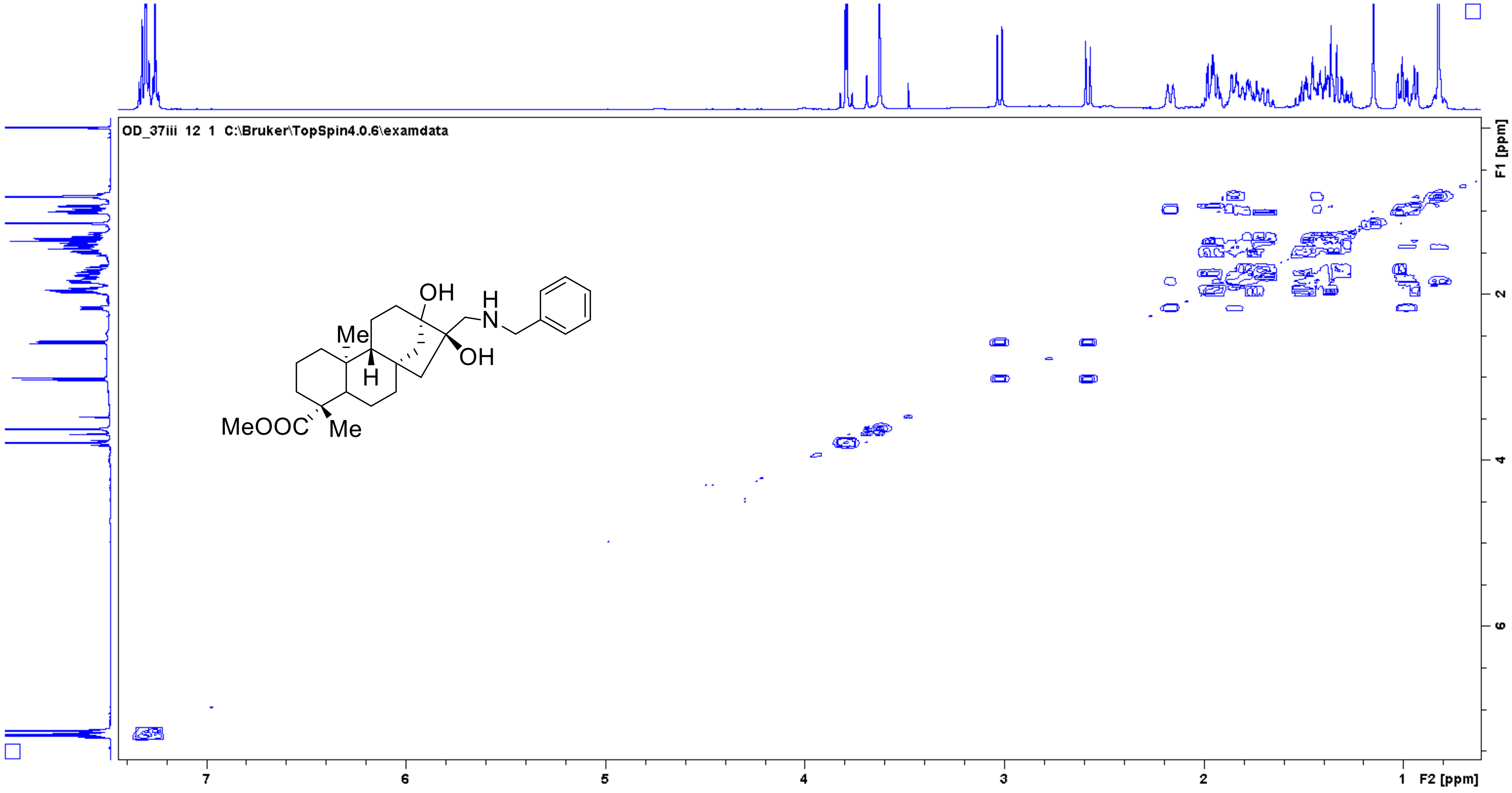

NOESY of compound **22**

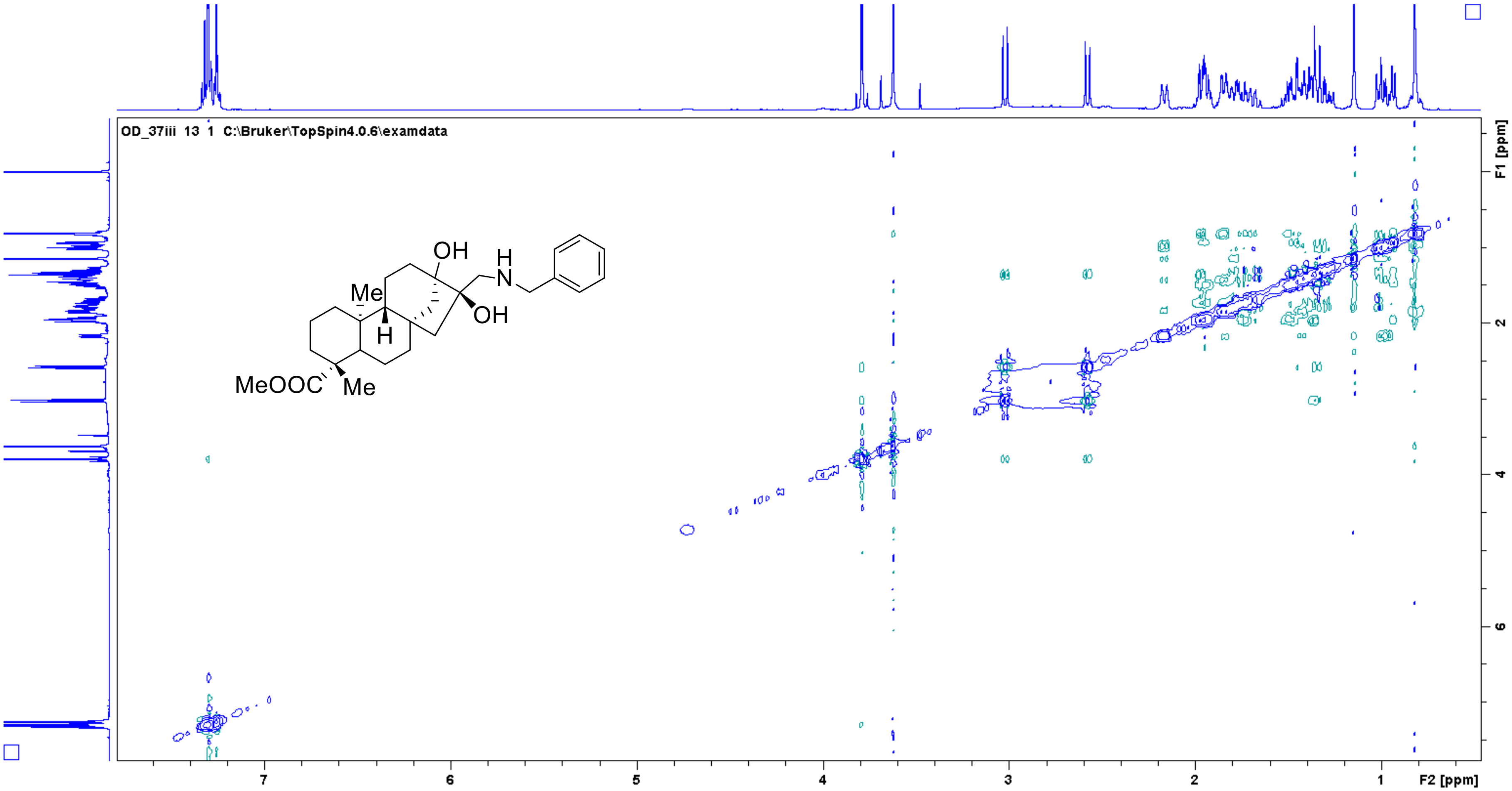

HSQC of compound **22**

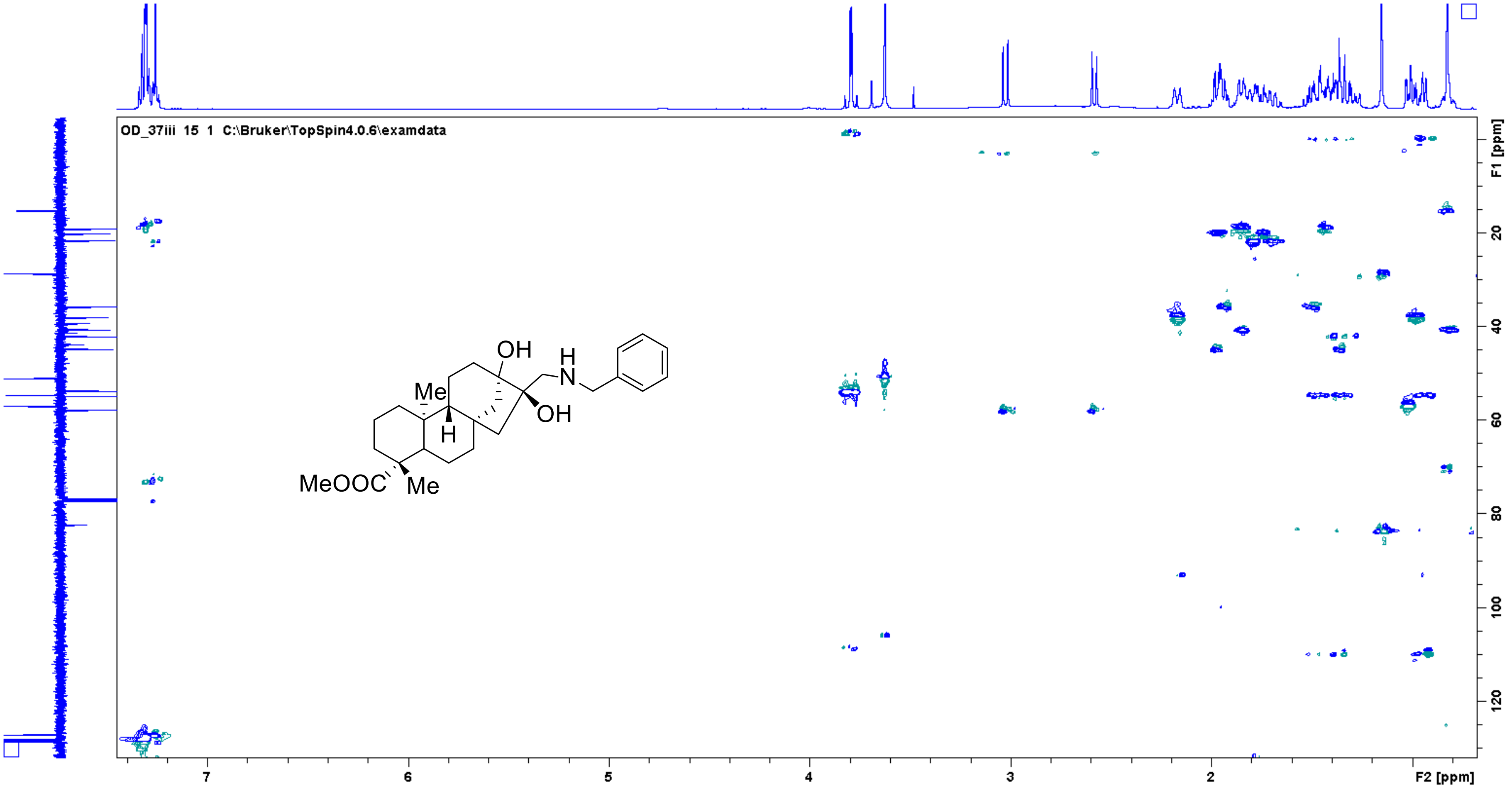

HMBC of compound **22**

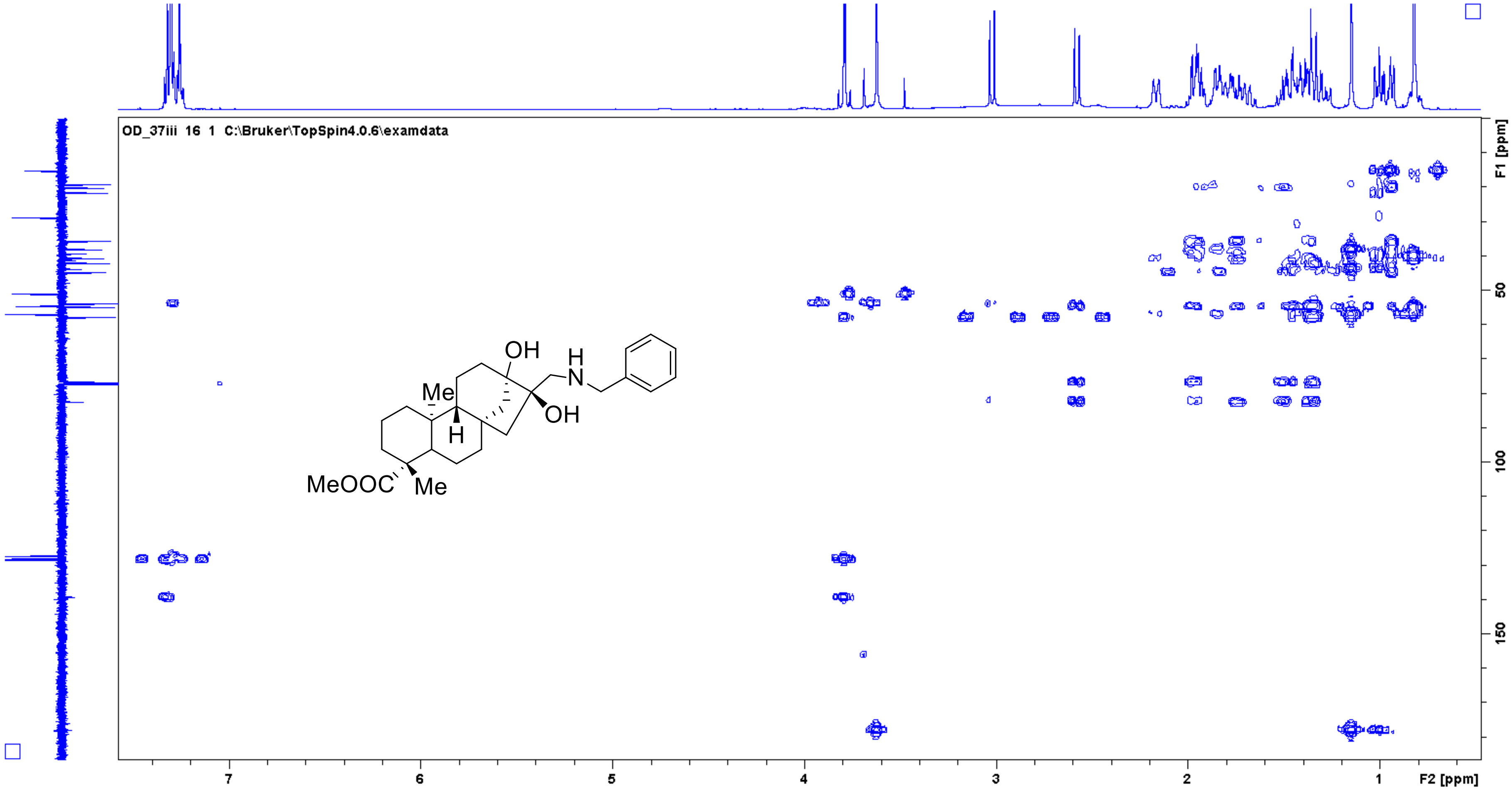

^{1}H-NMR of compound **23**

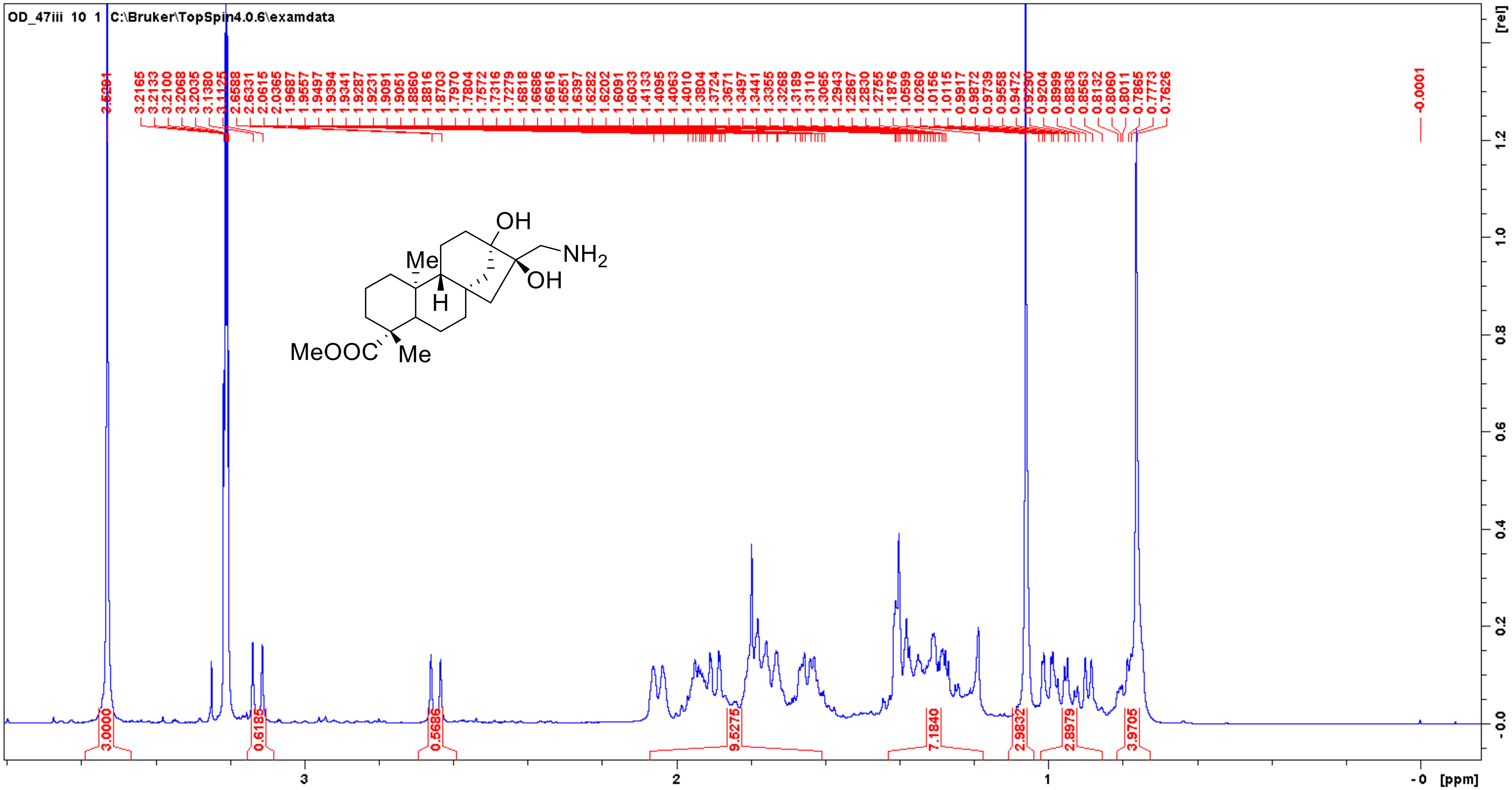

^{13}C-NMR of compound **23**

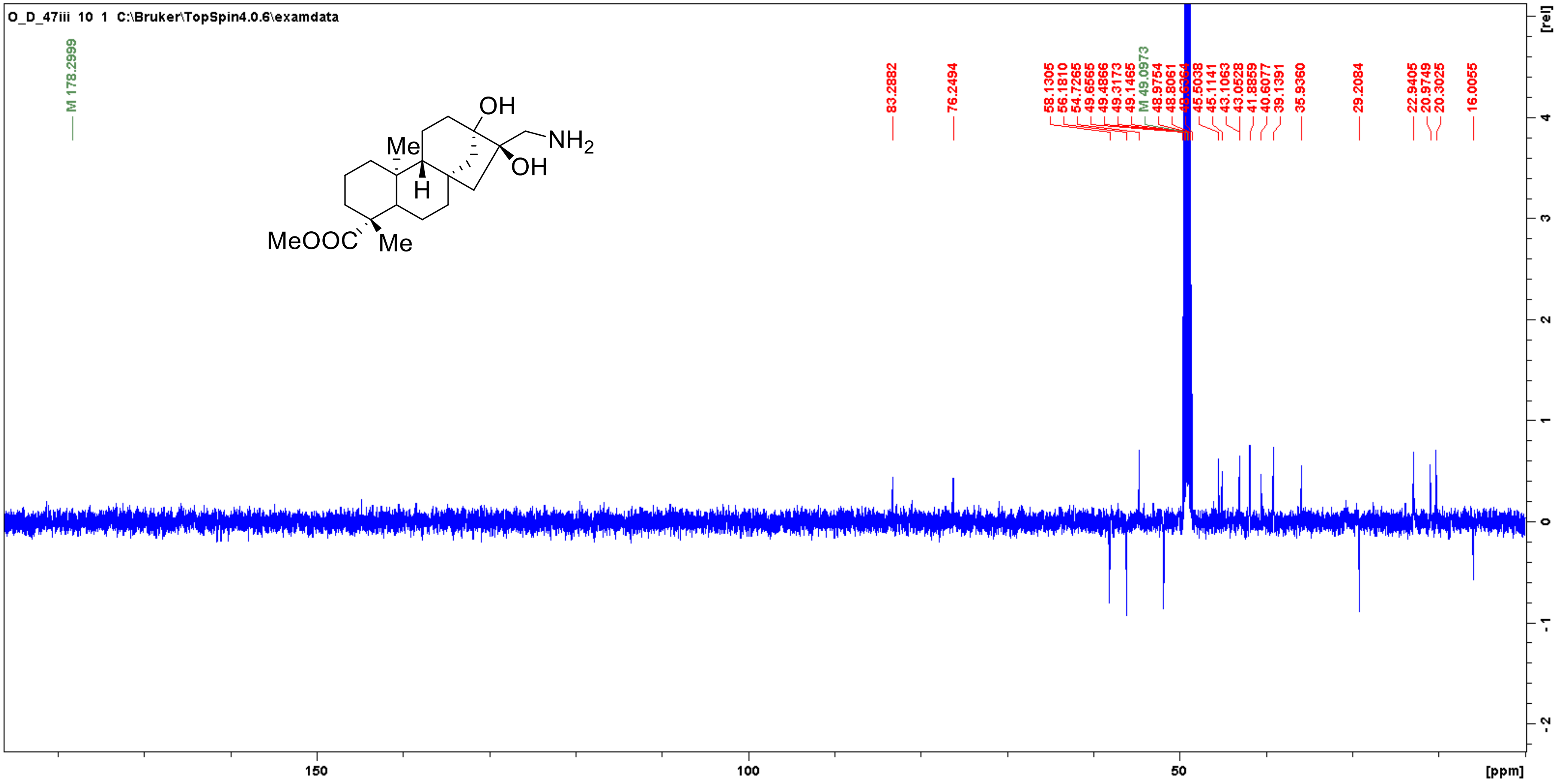

HSQC-NMR of compound **23**

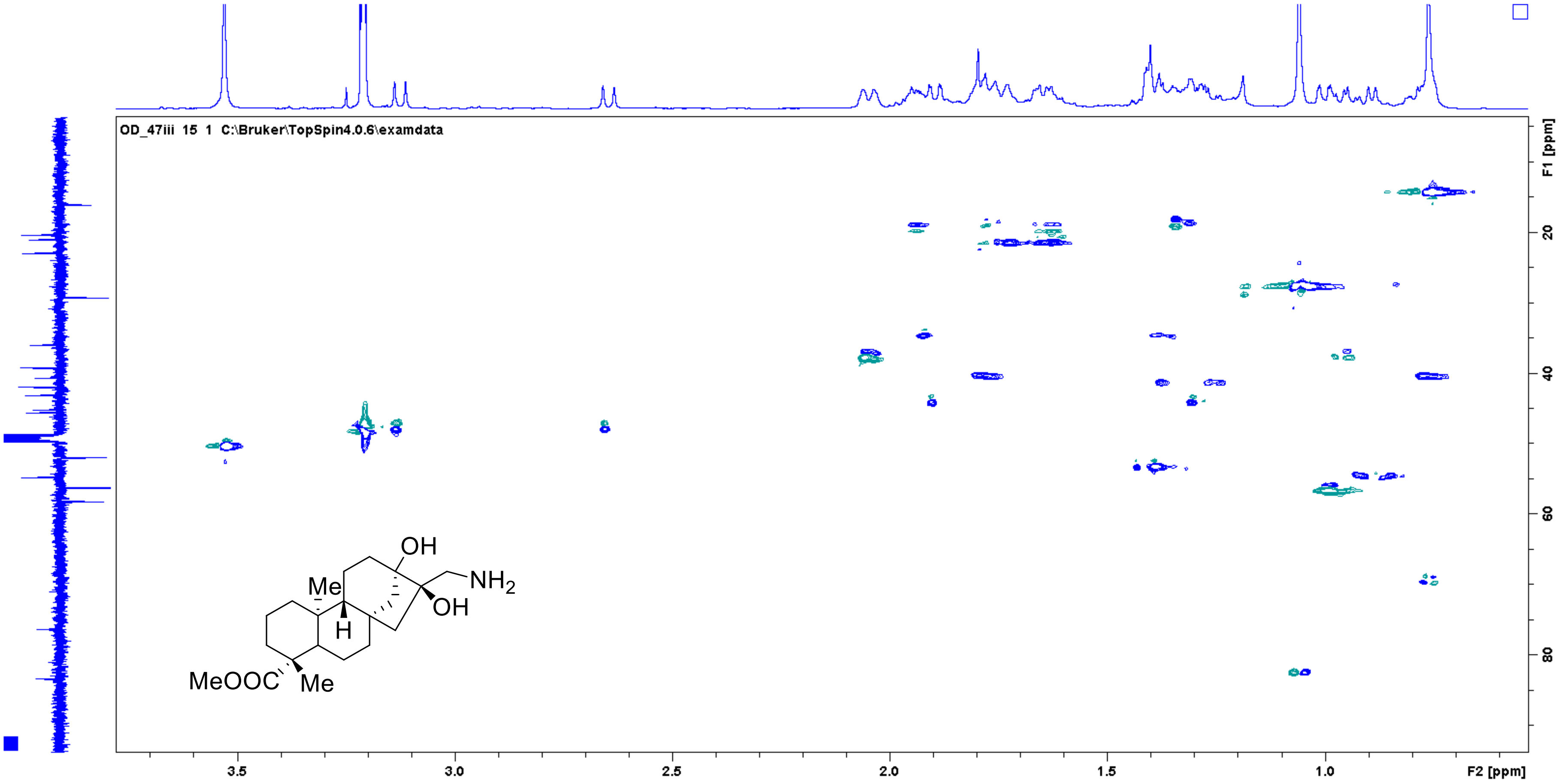

HMBC-NMR of compound **23**

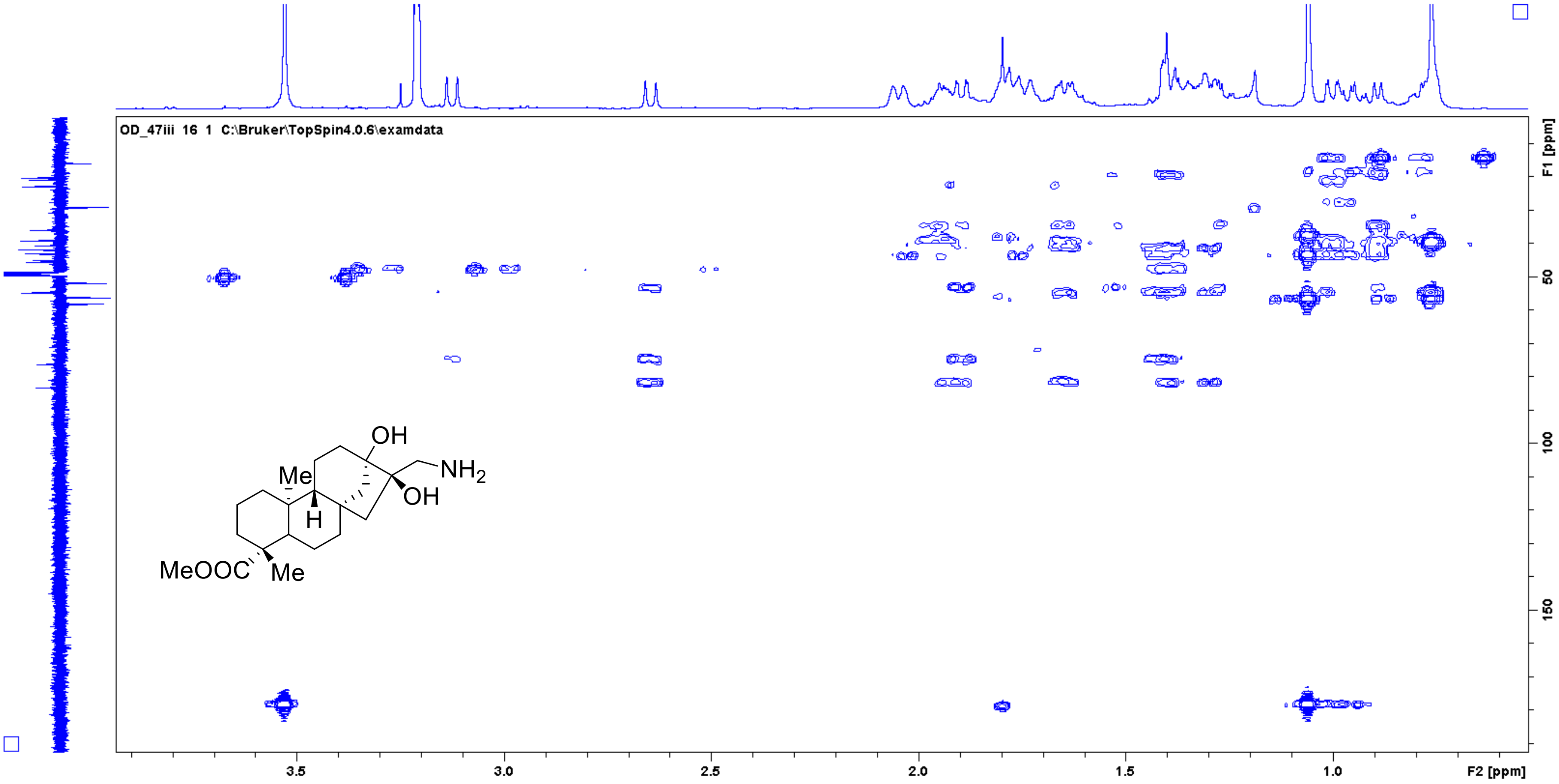

^{1}H-NMR of compound **24A and 24B**

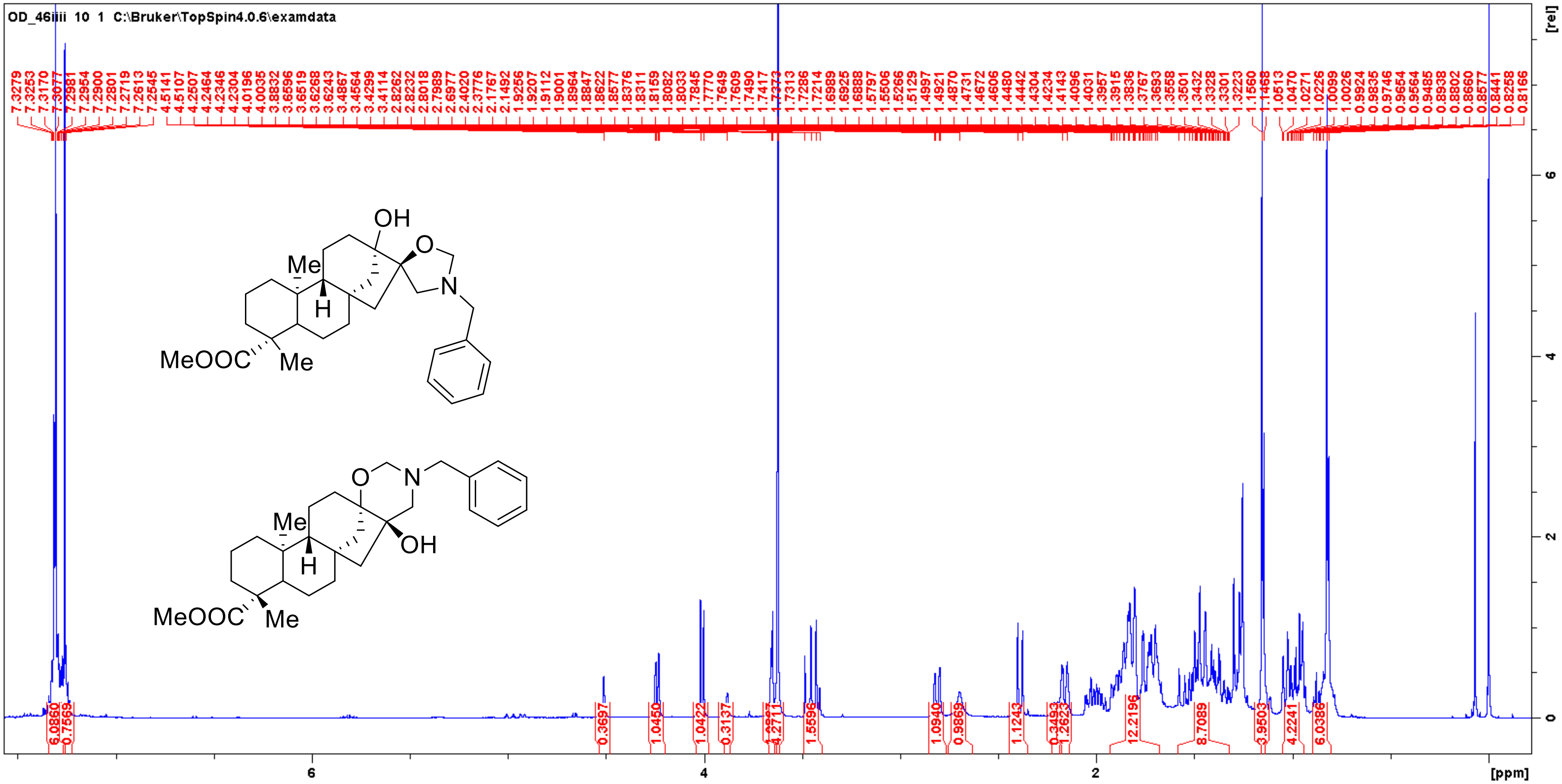

^{13}C-NMR of compound **24A and 24B**

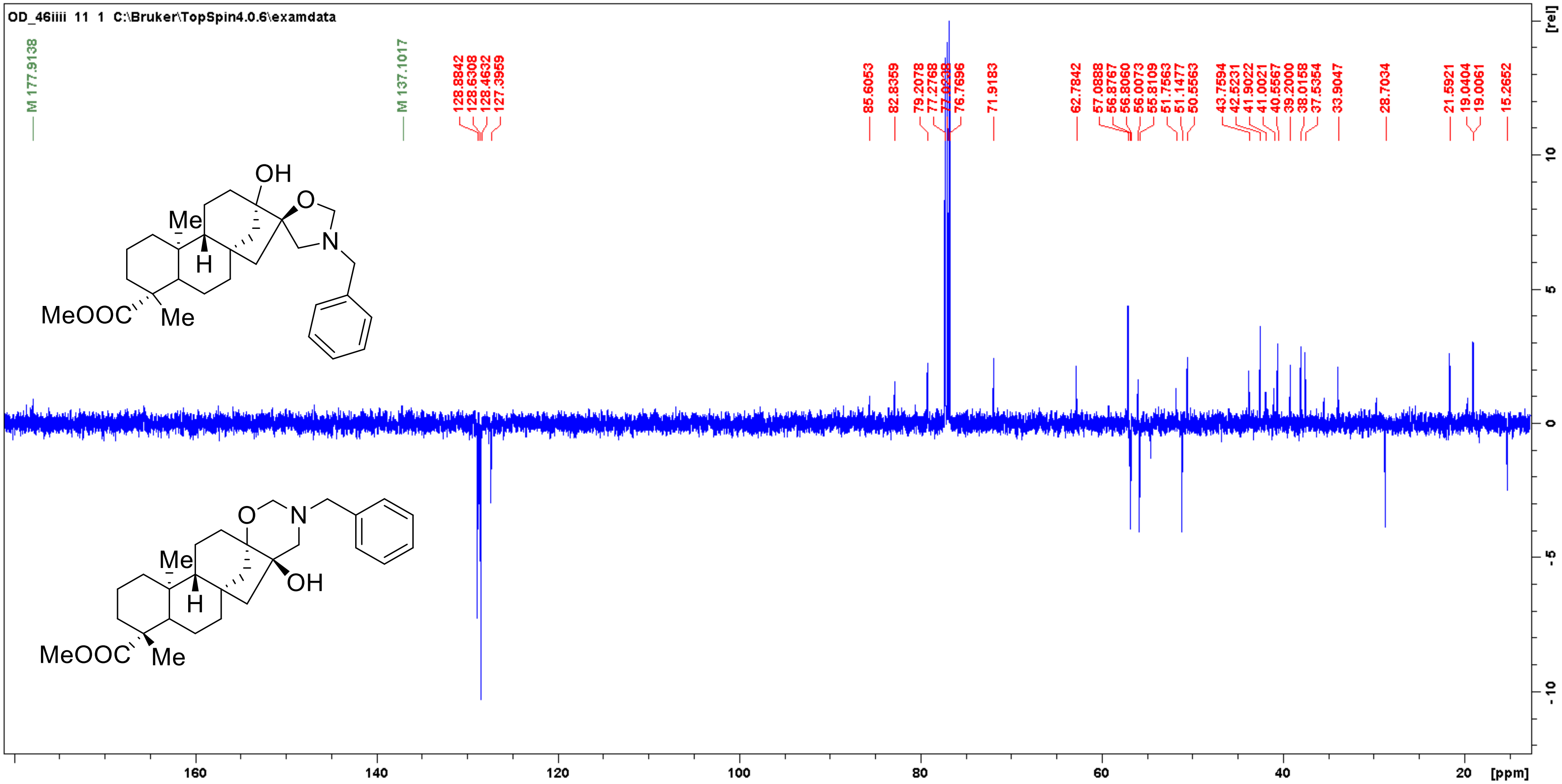

HSQC of compound **24A and 24B**

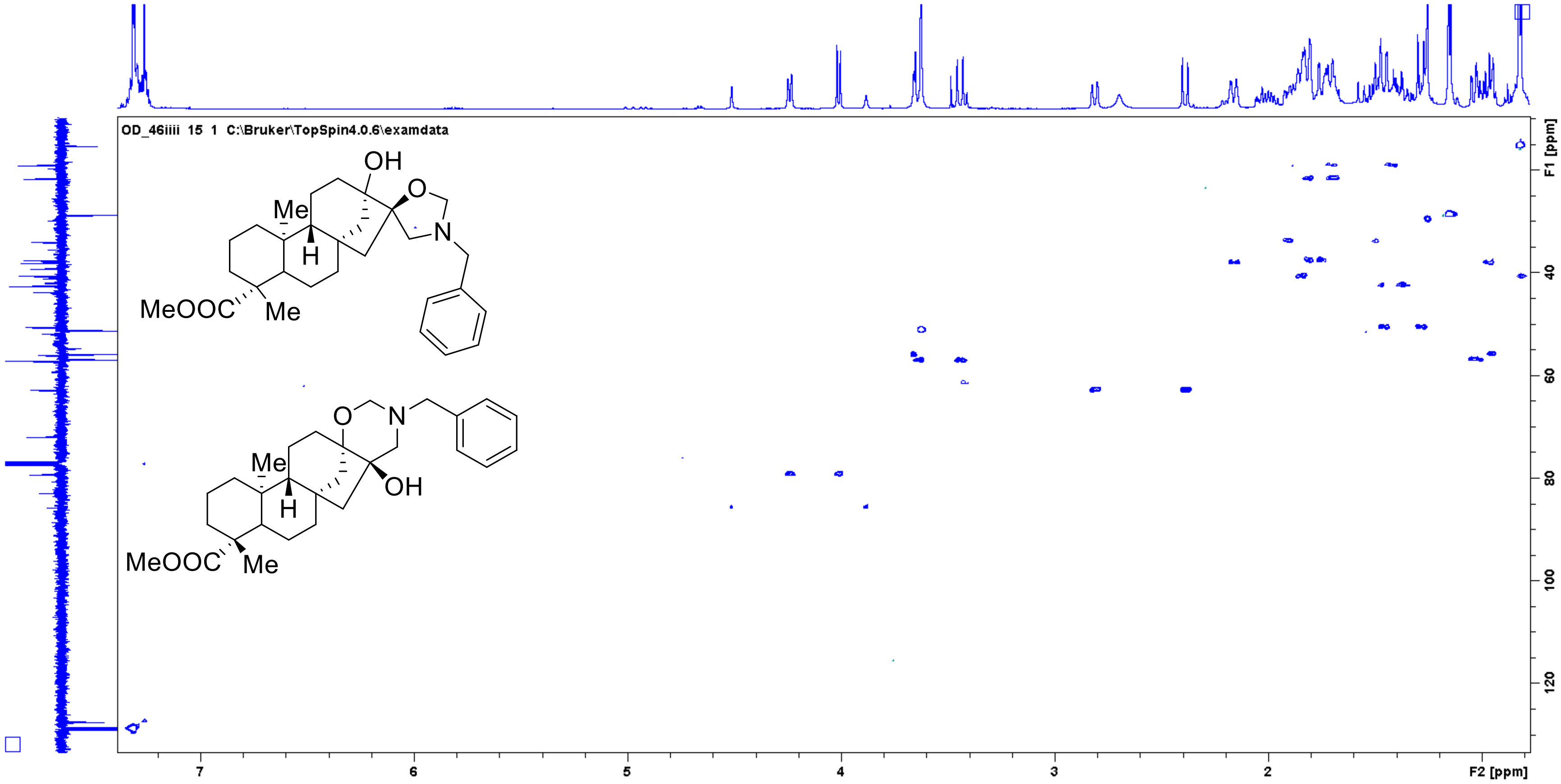

HMBC of compound **24A and 24B**

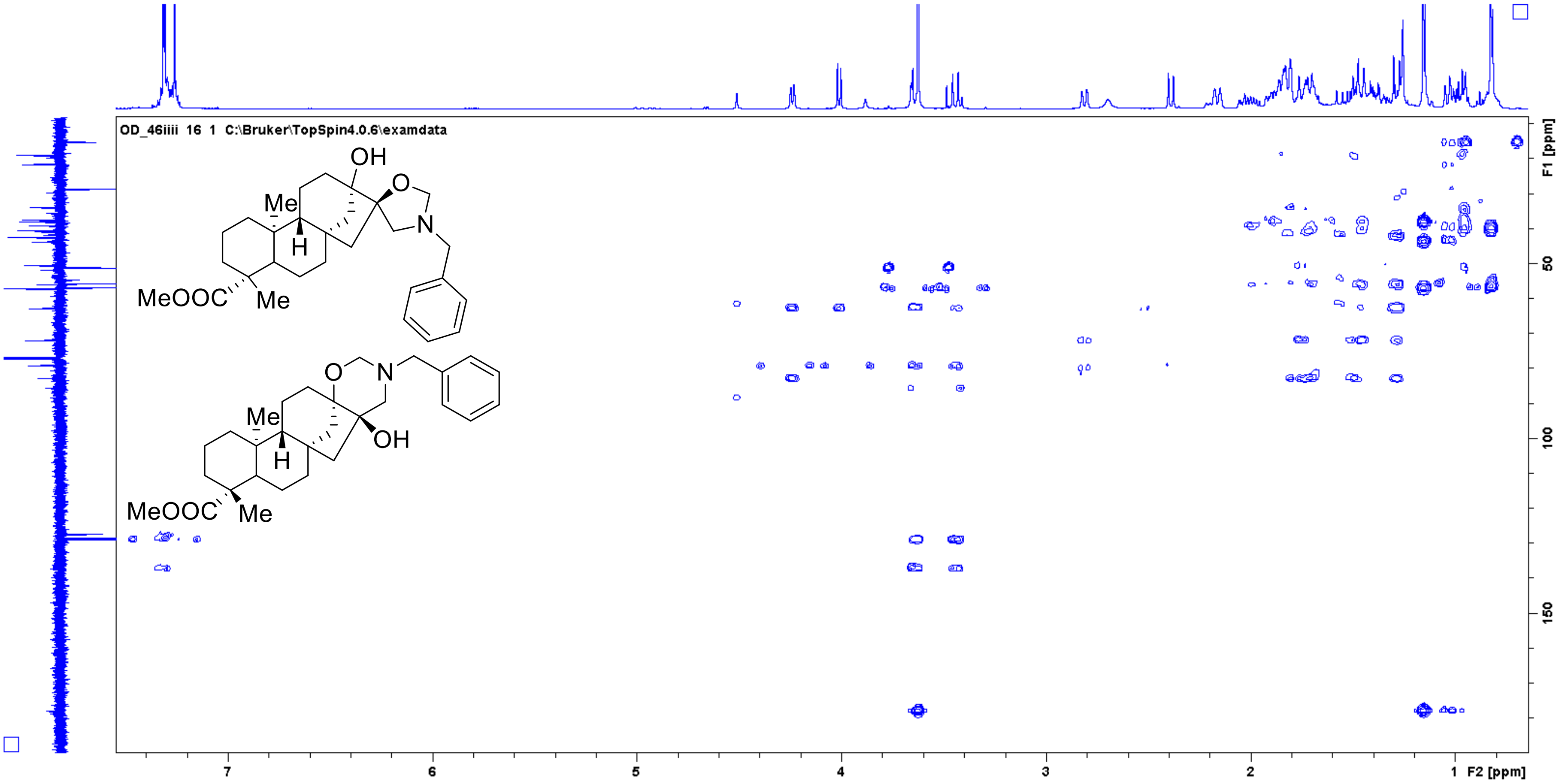

Supplement: Supplementary file 1 [file ijms-21-00184-s001.pdf]
